# Supplementary material for: Recognition of Protein Pupylation Sites by Adopting Resampling Approach
Source: Molecules. 2018 Nov 27;23(12):3097. doi: 10.3390/molecules23123097 (PMC6321382; doi:10.3390/molecules23123097)
Supplement: Supplementary file 1 [file molecules-23-03097-s001.zip › molecules-379758 proof supple/Supplement/PredictorPup/libsvm-3.11/FAQ.html]

LIBSVM FAQ

**# LIBSVM FAQ**
**last modified :** 
Tue, 11 Oct 2011 13:35:12 GMT
- All Questions(75)

**- Q1:\_Some\_sample\_uses\_of\_libsvm(2)
- Q2:\_Installation\_and\_running\_the\_program(12)
- Q3:\_Data\_preparation(7)
- Q4:\_Training\_and\_prediction(33)
- Q5:\_Probability\_outputs(3)
- Q6:\_Graphic\_interface(3)
- Q7:\_Java\_version\_of\_libsvm(4)
- Q8:\_Python\_interface(1)
- Q9:\_MATLAB\_interface(10)**


- Some courses which have used libsvm as a tool
- Some applications/tools which have used libsvm
- Where can I find documents/videos of libsvm ?
- Where are change log and earlier versions?
- How to cite LIBSVM?
- I would like to use libsvm in my software. Is there any license problem?
- Is there a repository of additional tools based on libsvm?
- On unix machines, I got "error in loading shared libraries" or "cannot open shared object file." What happened ?
- I have modified the source and would like to build the graphic interface "svm-toy" on MS windows. How should I do it ?
- I am an MS windows user but why only one (svm-toy) of those precompiled .exe actually runs ?
- What is the difference between "." and "\*" outputed during training?
- Why occasionally the program (including MATLAB or other interfaces) crashes and gives a segmentation fault?
- How to build a dynamic library (.dll file) on MS windows?
- On some systems (e.g., Ubuntu), compiling LIBSVM gives many warning messages. Is this a problem and how to disable the warning message?
- Why sometimes not all attributes of a data appear in the training/model files ?
- What if my data are non-numerical ?
- Why do you consider sparse format ? Will the training of dense data be much slower ?
- Why sometimes the last line of my data is not read by svm-train?
- Is there a program to check if my data are in the correct format?
- May I put comments in data files?
- How to convert other data formats to LIBSVM format?
- The output of training C-SVM is like the following. What do they mean?
- Can you explain more about the model file?
- Should I use float or double to store numbers in the cache ?
- How do I choose the kernel?
- Does libsvm have special treatments for linear SVM?
- The number of free support vectors is large. What should I do?
- Should I scale training and testing data in a similar way?
- Does it make a big difference if I scale each attribute to [0,1] instead of [-1,1]?
- The prediction rate is low. How could I improve it?
- My data are unbalanced. Could libsvm handle such problems?
- What is the difference between nu-SVC and C-SVC?
- The program keeps running (without showing any output). What should I do?
- The program keeps running (with output, i.e. many dots). What should I do?
- The training time is too long. What should I do?
- Does shrinking always help?
- How do I get the decision value(s)?
- How do I get the distance between a point and the hyperplane?
- On 32-bit machines, if I use a large cache (i.e. large -m) on a linux machine, why sometimes I get "segmentation fault ?"
- How do I disable screen output of svm-train?
- I would like to use my own kernel. Any example? In svm.cpp, there are two subroutines for kernel evaluations: k\_function() and kernel\_function(). Which one should I modify ?
- What method does libsvm use for multi-class SVM ? Why don't you use the "1-against-the rest" method ?
- After doing cross validation, why there is no model file outputted ?
- Why my cross-validation results are different from those in the Practical Guide?
- On some systems CV accuracy is the same in several runs. How could I use different data partitions? In other words, how do I set random seed in LIBSVM?
- I would like to solve L2-loss SVM (i.e., error term is quadratic). How should I modify the code ?
- How do I choose parameters for one-class svm as training data are in only one class?
- Why the code gives NaN (not a number) results?
- Why on windows sometimes grid.py fails?
- Why grid.py/easy.py sometimes generates the following warning message?
- Why the sign of predicted labels and decision values are sometimes reversed?
- I don't know class labels of test data. What should I put in the first column of the test file?
- How can I use OpenMP to parallelize LIBSVM on a multicore/shared-memory computer?
- How could I know which training instances are support vectors?
- Why training a probability model (i.e., -b 1) takes a longer time?
- Why using the -b option does not give me better accuracy?
- Why using svm-predict -b 0 and -b 1 gives different accuracy values?
- How can I save images drawn by svm-toy?
- I press the "load" button to load data points but why svm-toy does not draw them ?
- I would like svm-toy to handle more than three classes of data, what should I do ?
- What is the difference between Java version and C++ version of libsvm?
- Is the Java version significantly slower than the C++ version?
- While training I get the following error message: java.lang.OutOfMemoryError. What is wrong?
- Why you have the main source file svm.m4 and then transform it to svm.java?
- Except the python-C++ interface provided, could I use Jython to call libsvm ?
- I compile the MATLAB interface without problem, but why errors occur while running it?
- On 64bit Windows I compile the MATLAB interface without problem, but why errors occur while running it?
- Does the MATLAB interface provide a function to do scaling?
- How could I use MATLAB interface for parameter selection?
- I use MATLAB parallel programming toolbox on a multi-core environment for parameter selection. Why the program is even slower?
- How do I use LIBSVM with OpenMP under MATLAB?
- How could I generate the primal variable w of linear SVM?
- Is there an OCTAVE interface for libsvm?
- How to handle the name conflict between svmtrain in the libsvm matlab interface and that in MATLAB bioinformatics toolbox?
- On Windows I got an error message "Invalid MEX-file: Specific module not found" when running the pre-built MATLAB interface in the windows sub-directory. What should I do?

---

**Q: Some courses which have used libsvm as a tool**
  

- Institute for Computer Science,
  Faculty of Applied Science, University of Freiburg, Germany
  - Division of Mathematics and Computer Science.
    Faculteit der Exacte Wetenschappen
    Vrije Universiteit, The Netherlands. - Electrical and Computer Engineering Department,
      University of Wisconsin-Madison
      - Technion (Israel Institute of Technology), Israel.- Computer and Information Sciences Dept., University of Florida- The Institute of Computer Science,
            University of Nairobi, Kenya.- Applied Mathematics and Computer Science, University of Iceland.- SVM tutorial in machine learning
                summer school, University of Chicago, 2005.

[Go Top]


---


**Q: Some applications/tools which have used libsvm** 
  
(and maybe liblinear).

- LIBPMK: A Pyramid Match Toolkit
- Maltparser:
  a system for data-driven dependency parsing
- PyMVPA: python tool for classifying neuroimages
- SOLpro: protein solubility predictor
- BDVal: biomarker discovery in high-throughput datasets.
- Realtime object recognition
- scikits.learn: machine learning in Python

[Go Top]


---


**Q: Where can I find documents/videos of libsvm ?**
  

- Official implementation document:
    
  C.-C. Chang and
  C.-J. Lin.
  LIBSVM
  : a library for support vector machines.
  ACM Transactions on Intelligent
  Systems and Technology, 2:27:1--27:27, 2011.
  pdf, ps.gz,
  ACM digital lib.- Instructions for using LIBSVM are in the README files in the main directory and some sub-directories.
      
    README in the main directory: details all options, data format, and library calls.
      
    tools/README: parameter selection and other tools- A guide for beginners:
        
      C.-W. Hsu, C.-C. Chang, and
      C.-J. Lin.
      A practical guide to support vector classification
      - An introductory video
        for windows users.

[Go Top]


---


**Q: Where are change log and earlier versions?**
