# Supplementary material for: Recognition of Protein Pupylation Sites by Adopting Resampling Approach
Source: Molecules. 2018 Nov 27;23(12):3097. doi: 10.3390/molecules23123097 (PMC6321382; doi:10.3390/molecules23123097)
Supplement: Supplementary file 1 [file molecules-23-03097-s001.zip › molecules-379758 proof supple/Supplement/Supplementary Tables.pdf]

Table S1 The results of 20, 40,...,380,400 of features by the 20 step length by jackknife.

Number of features	SN	SP	ACC	MCC
20	0.929	0.3721	0.6987	0.3738
20+20	0.7596	0.4103	0.5394	0.1722
40	0.623	0.4879	0.5403	0.1087
40+20	0.4699	0.7371	0.6454	0.2085
60	0.5519	0.625	0.5984	0.1715
60+20	0.5847	0.6022	0.5963	0.1775
80	0.388	0.7835	0.6479	0.1775
80+20	0.6011	0.6454	0.6305	0.2348
100	0.4918	0.6835	0.6185	0.1713
100+20	0.6393	0.6464	0.644	0.2713
120	0.5956	0.6354	0.622	0.2196
120+20	0.6011	0.697	0.6648	0.2869
140	0.4153	0.7753	0.6551	0.1985
140+20	0.6175	0.7041	0.6752	0.3092
160	0.5519	0.6877	0.6423	0.2314
160+20	0.4973	0.7479	0.6642	0.2452
180	0.6284	0.7507	0.7099	0.3691
180+20	0.6995	0.6521	0.6679	0.3324
200	0.6011	0.6959	0.6642	0.2855
200+20	0.6011	0.7397	0.6934	0.3322
220	0.6175	0.6603	0.646	0.2644
220+20	0.6503	0.6822	0.6715	0.3168
240	0.6721	0.6712	0.6715	0.326
240+20	0.6776	0.6575	0.6642	0.3174
260	0.7213	0.6603	0.6807	0.3607
260+20	0.7213	0.7096	0.7135	0.41
280	0.6557	0.7616	0.7263	0.406
280+20	0.7268	0.6959	0.7062	0.4011
300	0.6885	0.7096	0.7026	0.38
300+20	0.8142	0.7644	0.781	0.5514
320	0.7049	0.7562	0.7391	0.4443
320+20	0.7322	0.789	0.7701	0.5052
340	0.8033	0.7096	0.7409	0.4852
340+20	0.7541	0.7205	0.7318	0.4512
360	0.7923	0.7205	0.7445	0.4862
360+20	0.7869	0.7534	0.7646	5152
380	0.7978	0.7616	0.7737	0.5337
400	0.7541	0.7562	0.7555	0.4885

Table S2. The prediction results of our method for the training dataset.

Protein Name	Peptide	Class label	Prediction label
A0QNF6	FGEVVDEESQKVVDAIASTPT	1	1
A0QP32	DYFAHWINVGKNADESKLPKV	1	1

A0QP90	AGATRIIGVAKAGLDDAGYRN	1	1
A0QPN2	ANADTVTLWSKLALESARARR	1	1
A0QQ65	NMKYADGTPIKASDFTYAIQR	1	1
A0QQH7	LFDEHGAYLAKTGGEVGVTTG	1	1
A0QQU5	AVEKVTETLLKSAKEVETKEQ	1	1
A0QQU5	VAAGANPLGLKRGIEKAVEKV	1	1
A0QS45	SGEPHKTKVAKVTWDQVREIA	1	1
A0QS98	KALEGDPKWVKSVEELMEAVD	1	1
A0QSE0	KRWRLVEILEKAK________	1	1
A0QSH8	KGTQAEKLSEKLGIPQISTGD	1	1
A0QSL6	SGHVGFKGSRKSTPFAAQLAA	1	1
A0QSU4	KIAQVVEVAAKEPEPSAAIRL	1	0
A0QUV7	GRGVGSAENFKIVEELADSLG	1	1
A0QUX7	QSLAVGATEQKDMSRMTIVVS	1	1
A0QUY3	GQTEAAARVDKAVSEHLATRG	1	1
A0QUY9	RVVADVLRGRKVADGVRMLIV	1	1
A0QUZ0	SFILNLSPFDKGSVLVAGPDF	1	1
A0QV10	ITSIASLETGKRLGPDPRTFN	1	1
A0QVB9	ALVEADGDFDKAVELLRIKGA	1	1
A0QVB9	ANDIETLKAAKTGDTTVEQAI	1	1
A0QVB9	IADLSAKIGEKLELRRAAYFD	1	1
A0QVM0	YSFDVDDDGLKNAYYQHREAY	1	1
A0QW25	DVIDEAATFLKQQEDKGVVVR	1	1
A0QWG8	TFYDDPDVLAKVSRGLGEAMV	1	1
A0QWT3	TFGSETVDPAKIEKAIGEVFD	1	0
A0QWT3	WEQTNKVDDLKSAI_______	1	1
A0QWV9	GAARRLGVSAKAREVRGSDRV	1	1
A0QWW2	SVTDLTAELAKSASVEDINAA	1	1
A0QWX9	KTILQAIELWKIVDRPNLLIK	1	1
A0QWX9	VDTEIDARLEKIGSDEALALR	1	1
A0QX20	ELTDAKNAFRKDIHNYVEQNH	1	1
A0QX81	GGVDVDAVVPKVRPIVDAVAQ	1	1
A0QX93	ATEEEDVLLEKELLADEKERA	1	1
A0QXX7	NSWPDNVSLDKARRLLWPIKQ	1	1
A0QYD4	ARRDDLEVAAKELDIESIVFD	1	1
A0QYS9	DLVARAGVLGKTLSHGIEELG	1	1
A0QZ54	IVYIRTLVTGKSSSASRAIDT	1	1
A0QZA1	SVARTLIDASKSAQMVVVGNR	1	1
A0QZE3	IHIEQGRELEKNNVTIGLVDR	1	1
A0R066	ERKIDVDEWQKKAGAGEITEV	1	1
A0R079	KTSDDIFKLIKDENVEYVDIR	1	1
A0R083	LLAAGLRGVEKGYVLGPQAED	1	1
A0R0B0	DHFFGRDPRTKALVADMSDQE	1	1
A0R0B2	VAAGAVAALDKLAEDPPAKAR	1	1
A0R0B3	AVQTEDKYGVKIPDEDLAGLR	1	1
A0R0B3	TVGDVVAYIQKLEEENPEAAA	1	1
A0R0B4	IRVLEDEFVTKWDLPVRIGGH	1	1
A0R0B5	VELRRLSYLQKMATVLGRRVW	1	1

A0R0W1	RAQVLVPELEKRLDDINAQLD	1	1
A0R0W4	DVADRIAVLNKGRIEQIGSPT	1	1
A0R1B5	AIEVVYGADVKGRGIESAAPL	1	1
A0R1V9	LTAVVGGDLSKVDAKQPDDYF	1	1
A0R1V9	PDDYFTRVTSKDYEGKWRIIF	1	1
A0R1V9	VGGDLSKVDAKQPDDYFTRVT	1	1
A0R1Y7	QSHQKAAAAWKDGVFADEVVP	1	1
A0R218	DVYVSMNMVRKNGLRRGDAVT	1	1
A0R220	NFDDCLELARKLTADFPTIAL	1	1
A0R2G5	NSLSGAVEVVKRDGTGITLNE	1	1
A0R2V7	NAQALADGFVKRDAGLVTGGT	1	1
A0R2W6	IGRQAIEGFYKNMTAATTDIK	1	1
A0R342	HPDIPAAATVKPGSEFRIECK	1	1
A0R3D2	LTANVRTRTGKGASRQARRDG	1	1
A0R3D6	VVVLGPGSIPKTPSGKLRRAN	1	1
A0R4C9	RDFVDAQQFSKLLSERGIAND	1	1
A0R4D0	TGAGIHEVDVKVA________	1	1
A0R4Z5	DEGPRETTLEKMAALQPLVEG	1	1
A0R518	EDLAETVELVKGLNRRIVAEE	1	1
A0R566	MPNSNPVAAWKALKDGNARFV	1	1
A0R5E1	SGFRTLEENQKVEFEVGQSPK	1	0
A0R5M8	FDLDAFEHVAKWNSDEERIEM	1	1
A0R5R5	LSREQATWAQKAIAQEGLTDL	1	1
A0R5R7	EILEDRLGESKIASWTDPKGG	1	1
A0R635	HNETVVLDFPKRVVREPIPDE	1	1
A0R647	_MTNSGALDTKFHALIQDQIR	1	1
A0R652	HHSKHHATYVKGVNDAIAKLE	1	1
A0R652	WKNLSPNGGDKPTGELAAAID	1	1
A0R6E3	PATEAETAQIKDLIAALGG__	1	1
A0R6E3	RAAVVLSGLRKLQGSLAEAAS	1	1
A0R6Q0	IKTADVDAAAKEIEATGGAII	1	1
A0R7F9	VIRKDGGTVDKVDIWGRRRLA	1	1
A0R7G6	NFVAAFDVDAKKVGFDLSEAI	1	1
A4ZHR8	ALGGTLTKLSKDQAEYIGVDV	1	1
EG10025	APFAGTVKEIKVNVGDKVSTG	1	1
EG10207	FADYDEARFQKEGFRVVPPAA	1	1
EG10251	DVKILDPRVGKEFPLPTYATS	1	1
EG10367	SVVDLTVRLEKAATYEQIKAA	1	0
EG10407	QEIFTREEMIKYFTLNAVSKS	1	1
EG10410	LAMQVVVLRDKLEPYFTEGRY	1	1
EG10416	SAEQVDPRDEKVANLEAQLAE	1	1
EG10416	TRERDGILRVKAEMENLRRRT	1	1
EG10489	_______MESKVVVPAQGKKI	1	1
EG10489	WLKVKNPNTGKEIVIKDVIAD	1	1
EG10492	MNDAFWDELLKVRGEVNKVIE	1	1
EG10762	YLRWAESRGFKTEIIEESEGE	1	1
EG10770	XXXDIVEYLVKQIKG______	1	1
EG10770	KSFRVQELAEKLYSELRAQGI	1	1

EG10770	QLGTKYSEALKASVQGEDGRN	1	1
EG10803	EGKVFLNIGDKFLLDANLGKG	1	1
EG10803	RADKVREIAAKLGRHVAILGD	1	1
EG10876	ARAAIEAAGGKIEE_______	1	1
EG10903	AALELAEQREKPTWLEVDAGK	1	1
EG10903	REKAKKQSRVKAALELAEQRE	1	1
EG10905	YTAAITGAEGKIHRLEDWGRR	1	1
EG10954	TYVTNLNNLIKGTAFEGKSLE	1	1
EG11192	GELILNEKLAKQLVTAANWVK	1	1
EG11223	HSWVEGKLIDKVGQLGKKLHT	1	1
EG11415	TQVINSKTPLKSYPLDIHNVQ	1	1
EG11427	AWNEKFAAYAKAYPQEAAEFT	1	1
EG11427	FDAKAKEFIAKLQANPAKIAS	1	1
EG11518	LQLGKDLDRFKNLVLVHAARY	1	1
EG11615	EYALLSRLMLKAGSPVHREIL	1	1
EG11615	LHFLARIRQKKYTLPVLILTA	1	1
EG12204	__MAKIKGQVKWFNESKGFGF	1	1
EG12411	RKAGIPLALNKLNAVPTTAYP	1	0
EG12503	EGVKYLEENAKKEGVNSTESG	1	0
EG12545	PNYLHKEPVIKAAKNKKHVFC	1	1
EG12669	GEIAADFAQDKKSDFDLTPFR	1	1
EG12712	INSNEELALPKEKLQELHI__	1	1
EG12982	________MQKVVLATGNVGK	1	1
EG13014	LITYGGGSVKKTGVLDQVLDA	1	1
EG13952	AGGIYLFTDEKSALAYLEKHT	1	1
EG13952	AYLEKHTARLKNLGVEEVVAK	1	1
O05598	VVVLGPGTIPKTPSGKLRRAN	1	1
O05814	YSFDIDAAGLKAAYHAHREAY	1	1
O06188	SAVEAAGKLVKIETDIPRGPA	1	1
O06391	IAARMTTAVDKARVRGAGHEV	1	0
O33294	LHSRGFSEDPKALEPAVRKVE	1	1
O33341	EKSLDDLRLFKALVDAASGYA	1	1
O53176	FMRNSIDLYHKQAADTGARIV	1	1
O53204	DKGYDGRALIKDLSFSLPRNG	1	1
O53226	TKTTRLTPGDKAPAFTLPDAD	1	1
O53226	YNVKATGHVAKLRRDLSV___	1	1
O53442	QHVMKGYRAEKYTQVETLVYM	1	1
O53618	GILRPKSGAIKFDEVDITTLQ	1	1
O53665	TALVYLSPDAKPAATGLESTM	1	1
O53665	TQALAPGLAAKGITINAVAPG	1	1
O53871	AEAWSGGYFAKSVVPVRDQNG	1	1
O69687	IGATLSAEQAKWGQKAVEDEG	1	1
O86352	VGLSRGWSLIKSVRLGNAAGA	1	1
P09621	SARDVLAVVSK__________	1	1
P0A508	EWQLRVGAGEKLGFAQNDIEL	1	1
P0A520	AVEKVTETLLKGAKEVETKEQ	1	1
P0A556	PAKEDEEVVRKATTDEPFAAL	1	1
P0A5B7	LPVGADEDDIKATYDKGILTV	1	0

P0A5B7	LTIKAERTEQKDFDGRSEFAY	1	1
P0A5B7	LTVSVAVSEGKPTEKHIQIRS	1	1
P0A5B7	RAELPGVDPDKDVDIMVRDGQ	1	1
P0A5F7	QAHQIVEQAHKVALEASSSGR	1	1
P0A5H3	LDDATIAKFQKELAAMGFKFQ	1	1
P0A5L2	VARITAPDEPKYDADFAAVRQ	1	1
P0A5U4	ENADVADEIEKKIKEKLGIGA	1	1
P0A5Z4	HVQRLRAKVEKDPENPTVVLT	1	1
P0CG95	SGAGIHEVDVKIT________	1	1
P0CG99	IPDITFELAKKNEDMYLFSPY	1	1
P0CH36	ADVTVLSQSLKKMEDGLRLGA	1	0
P17670	SRYAAATSQTKGLIFG_____	1	1
P60176	ALGGHLTKLTKEQAEYLGVDV	1	1
P60796	TFFDDPDVLAKVSRGLGEAMV	1	1
P63458	VAAGRLTALEKLAEDPPAKAR	1	1
P63523	KPEVVYFEPDKLSTGSANRCE	1	1
P63568	GLVRRAEVLGKSLRHGIEALG	1	1
P63673	SQKLTEEAADKLTEVIKNFKK	1	1
P65161	ESLAVGATECKDRSRMTIVVS	1	1
P65232	AHPELAEQVAKELDVHVTSQD	1	1
P65277	ITAATVVLPFKIDDHSAWRLL	1	0
P65573	DVLARLEVDAKEIIGRYPDRR	1	1
P65880	LFDEHGEYLSKTGREFGVTTG	1	1
P66056	SAEPHKTKVAKVTWDQVREIA	1	1
P66902	NFDDCLELARKMAADFPTISL	1	1
P69440	DGYPRSVEQAKALHEMLERRG	1	1
P71703	KFVAAFDVDAKKVGFDLSDAI	1	1
P71724	QAVAAAEVLGKRQPLLIVSSD	1	1
P71973	AIIDDASTFFKQQEERGVVVR	1	1
P77899	TFGTETEDPVKIEKAIGEVFD	1	1
P96382	DGKLGAFVEVKNSTIGTGTKV	1	1
P96825	WAEITDLSGAKIAGFKL____	1	1
Q10504	DHFFGRDPRTKALVENMSDQD	1	1
Q10530	RARIVKEQADKILAKLGGDDS	1	1
Q10682	VVAVGFTFDPKTHIARVITTG	1	1
Q50685	DTVVPRASMPKFLSTARGLAA	1	1
Q7D8W0	GAGELVSAAGKALRDWDALMV	1	1
A0QNF6	ATLHTNRGDIKIALFGNHAPK	0	0
A0QNF6	DEFHPELQFDKPYLLAMANAG	0	0
A0QNF6	KIALFGNHAPKTVNNFVGLAQ	0	0
A0QNF6	NGSQFFITVGKTPHLNRRHTI	0	0
A0QNF6	NNFVGLAQGTKDYSTENASGG	0	0
A0QNF6	TGTGRGGPGYKFEDEFHPELQ	0	0
A0QP32	AGTFQKLNPEKQPNSYLALSD	0	0
A0QP32	AHLVEAGTFQKLNPEKQPNSY	0	0
A0QP32	DKLGDDGFFVKALHSIGAPLE	0	0
A0QP32	EGDPQHLIDWKGNDWTPESGE	0	0
A0QP32	FVGEKLPTGLKDEFDALKHRL	0	0

A0QP32	GDDIAWMRFGKDGRLYATNPE	0	0
A0QP32	GKNADESKLPKVFFVNWFRRG	0	0
A0QP32	GSEQTAAAEGKVGTVRRDPMA	0	0
A0QP32	GWLAEHMLILKLISPENKAYF	0	0
A0QP32	GYGGNALLGKKCYSLRIASAM	0	0
A0QP32	IAAAFPSACGKTNLAMLQPTI	0	0
A0QP32	INVGKNADESKLPKVFFVNWF	0	0
A0QP32	ISAILFGGRRKTTVPLITEAR	0	0
A0QP32	KDVPWPCNDTKYITHFPETRE	0	0
A0QP32	LILKLISPENKAYFIAAAFPS	0	0
A0QP32	LKWAVERIEHKADGKSTPIGI	0	0
A0QP32	MTRMGRAALDKLGDDGFFVKA	0	0
A0QP32	PADVDEALAVKPEEWRAELPL	0	0
A0QP32	PGFGENSRVLKWAVERIEHKA	0	0
A0QP32	SGYGGNALLGKKCYSLRIASA	0	0
A0QP32	TGLKDEFDALKHRLSEEG___	0	0
A0QP32	TNWSSNPNAMKTIAAGNTVFT	0	0
A0QP32	VERIEHKADGKSTPIGIVPTA	0	0
A0QP32	AGNTVFTNVAKTDDGDVWWEG	0	0
A0QP32	CMGPLDAEDPKLGVEITDSEY	0	0
A0QP32	GNDWTPESGEKAAHPNSRYCT	0	0
A0QP32	IEEWFEFVGEKLPTGLKDEFD	0	0
A0QP32	SIGAPLEPGQKDVPWPCNDTK	0	0
A0QP32	TIPGLDTAPTKHQGLLAWVQE	0	0
A0QP90	AGVPFYLRTGKRMSQRFSEIV	0	0
A0QP90	DEAIRLEMTAKEPGSGGRLRP	0	0
A0QP90	HVNRESVRDEKRKVLEALEPL	0	0
A0QP90	NRESVRDEKRKVLEALEPLTA	0	0
A0QP90	QIFRIDHYLGKESVQQLLVTR	0	0
A0QP90	QRFSEIVVQFKPVPLPMFPGI	0	0
A0QP90	VTESSRVVLEKPIGRDLASAQ	0	0
A0QP90	FGGTGDLAARKLLPALYLRDR	0	0
A0QP90	HSRTETFVAVKAHIRNWRWAG	0	0
A0QPN2	APDVAWDSQAKTAVSSRSLNL	0	0
A0QPN2	ARAGGKFSAHKDATSTGAGLN	0	0
A0QPN2	ARARRSAEGAKARARAASKAK	0	0
A0QPN2	ASRMRDLGLAKNEEGPEPQDF	0	0
A0QPN2	DATFQAAFPLKGKPPNVYGFP	0	0
A0QPN2	DPTTGKNGIVKTLGTARAGGK	0	0
A0QPN2	EAPFTRNDTQKLRGVSNRKPD	0	0
A0QPN2	GAKARARAASKAKGLGTNLSL	0	0
A0QPN2	GLGTNLSLPPKLLPSRESGRG	0	0
A0QPN2	GRGYPGVFEGKEFDPEAPFTR	0	0
A0QPN2	IRVRPDESEKKLHHTLFGGPP	0	0
A0QPN2	KARARAASKAKGLGTNLSLPP	0	0
A0QPN2	KTLGTARAGGKFSAHKDATST	0	0
A0QPN2	LPVDTDPTTGKNGIVKTLGTA	0	0
A0QPN2	PPNVYGFPLNKARAKDEFDAI	0	0

A0QPN2	RTDVTVRRDGKTFLQSFGRGY	0	0
A0QPN2	SNRRVEVQRNKGLGEMDADDF	0	0
A0QPN2	TFQAAFPLKGKPPNVYGFPLN	0	0
A0QPN2	TQKLRGVSNRKPDLHGTEVRI	0	0
A0QPN2	VIRVRPDESEKKLHHTLFGGP	0	0
A0QPN2	YVTMPPLFVVKAGDERIYCQD	0	0
A0QPN2	CEGDSALGTIKAARDATFQAA	0	0
A0QPN2	GGGSHLTAAIKGLSEALAERA	0	0
A0QPN2	SERDAAVAQLKASSNRRVEVQ	0	0
A0QPN2	YGFPLNKARAKDEFDAIERIL	0	0
A0QQ65	ADEVADAHVDKIIVTQNKSNS	0	0
A0QQ65	DDFFRPLINGKSILPTNGNNF	0	0
A0QQ65	EDMPEVSPDGKTYKLKLRQNM	0	0
A0QQ65	FNQDFTSFALK__________	0	0
A0QQ65	FTLYPGPDMDKAKQLIAEANP	0	0
A0QQ65	HVDKIIVTQNKSNSAQVTDIE	0	0
A0QQ65	IVGANDYAEGKADTITGIQTN	0	0
A0QQ65	LTQLGFNATLKVIAGDVYWTT	0	0
A0QQ65	LYPGPDMDKAKQLIAEANPAD	0	0
A0QQ65	MERNPQFQTVKDAGADEVADA	0	0
A0QQ65	_____MIAGRKCLKIASVAAV	0	0
A0QQ65	__MIAGRKCLKIASVAAVAVL	0	0
A0QQ65	PEVSPDGKTYKLKLRQNMKYA	0	0
A0QQ65	SAQVTDIEQNKTDFMHDPPDA	0	0
A0QQ65	TYKLKLRQNMKYADGTPIKAS	0	0
A0QQ65	VSPDGKTYKLKLRQNMKYADG	0	0
A0QQ65	YNTYVPLLTYKHAKGEEGAEV	0	0
A0QQ65	YVPLLTYKHAKGEEGAEVAPG	0	0
A0QQ65	DFTYAIQRLFKADSGGSVFFG	0	0
A0QQ65	LLTQQLTDETKKGYADLDRAY	0	0
A0QQ65	LTQQLTDETKKGYADLDRAYM	0	0
A0QQ65	PPDADRLPEVKARFGDRFRLE	0	0
A0QQ65	SRVAIPELDAKMNELLTQQLT	0	0
A0QQ65	TVWTDDEPDRKRIGEYYHDVL	0	0
A0QQH7	AEKIEAALEFKNQVLVKIYNR	0	0
A0QQH7	ALEFKNQVLVKIYNRKALEPA	0	0
A0QQH7	DKVVERWAGSKKIGTTGRGIG	0	0
A0QQH7	KVVERWAGSKKIGTTGRGIGP	0	0
A0QQH7	LLMPYHVAIDKVVERWAGSKK	0	0
A0QQH7	NQVLVKIYNRKALEPAEVLEN	0	0
A0QQH7	PVCVGYTVDGKRVDEMPMTQS	0	0
A0QQH7	TRITTVLGILKAYTTRVGSGP	0	0
A0QQH7	VDPGVLLTELKGLEDRGVDTS	0	0
A0QQH7	VNGITDYFLTKLDVLSSLETV	0	0
A0QQH7	DVLDEQVLAEKIEAALEFKNQ	0	0
A0QQH7	ENLLEQAEGFKHRIADARLLL	0	0
A0QQH7	GAREFEDLPAKARDYVLRLEE	0	0
A0QQU5	AATEVELKERKHRIEDAVRNA	0	0

A0QQU5	AKLAGGVAVIKAGAATEVELK	0	0
A0QQU5	EDPYILLVSSKVSTVKDLLPL	0	0
A0QQU5	ENSDSDYDREKLQERLAKLAG	0	0
A0QQU5	FLTTEAVVADKPEKAAAPAGD	0	0
A0QQU5	IRGTFKSVAVKAPGFGDRRKA	0	0
A0QQU5	IVRVALSAPLKQIALNGGLEP	0	0
A0QQU5	KAGAATEVELKERKHRIEDAV	0	0
A0QQU5	KEIELEDPYEKIGAELVKEVA	0	0
A0QQU5	LELTEGMRFDKGYISGYFVTD	0	0
A0QQU5	LKRGIEKAVEKVTETLLKSAK	0	0
A0QQU5	LLAAGVADPVKVTRSALQNAA	0	0
A0QQU5	________MAKTIAYDEEARR	0	0
A0QQU5	NPLGLKRGIEKAVEKVTETLL	0	0
A0QQU5	PLLEKVIQSGKPLLIIAEDVE	0	0
A0QQU5	TEAVVADKPEKAAAPAGDPTG	0	0
A0QQU5	TITNDGVSIAKEIELEDPYEK	0	0
A0QQU5	TVKDLLPLLEKVIQSGKPLLI	0	0
A0QQU5	VKAPGFGDRRKAMLQDMAILT	0	0
A0QQU5	ADAVKVTLGPKGRNVVLEKKW	0	0
A0QQU5	ADVSLLGKARKVVVTKDETTI	0	0
A0QQU5	DREKLQERLAKLAGGVAVIKA	0	0
A0QQU5	GAELVKEVAKKTDDVAGDGTT	0	0
A0QQU5	GEALSTLVVNKIRGTFKSVAV	0	0
A0QQU5	GGLEPGVVAEKVSNLPAGHGL	0	0
A0QQU5	GPKGRNVVLEKKWGAPTITND	0	0
A0QQU5	HRIEDAVRNAKAAVEEGIVAG	0	0
A0QQU5	IGAELVKEVAKKTDDVAGDGT	0	0
A0QQU5	IGDLIAEAMDKVGNEGVITVE	0	0
A0QQU5	KVTETLLKSAKEVETKEQIAA	0	0
A0QQU5	LETADVSLLGKARKVVVTKDE	0	0
A0QQU5	LGKARKVVVTKDETTIVEGAG	0	0
A0QQU5	LLKSAKEVETKEQIAATAGIS	0	0
A0QQU5	LLVSSKVSTVKDLLPLLEKVI	0	0
A0QQU5	LVVNKIRGTFKSVAVKAPGFG	0	0
A0QQU5	PKGRNVVLEKKWGAPTITNDG	0	0
A0QQU5	PYEKIGAELVKEVAKKTDDVA	0	0
A0QQU5	RGLNSLADAVKVTLGPKGRNV	0	0
A0QS45	APKKKVAGLIKLQIQAGQANP	0	0
A0QS45	DQVREIAETKKADLNANDIDA	0	0
A0QS45	GVQKGSGEPHKTKVAKVTWDQ	0	0
A0QS45	KLLLKAAGVQKGSGEPHKTKV	0	0
A0QS45	KTPPAAKLLLKAAGVQKGSGE	0	0
A0QS45	LNANDIDAAAKIIAGTARSMG	0	0
A0QS45	_____MAPKKKVAGLIKLQIQ	0	0
A0QS45	QHGVNIMEFCKAYNAATESQR	0	0
A0QS45	TFALKTPPAAKLLLKAAGVQK	0	0
A0QS45	WDQVREIAETKKADLNANDID	0	0
A0QS45	YEDRSFTFALKTPPAAKLLLK	0	0

A0QS45	QKGSGEPHKTKVAKVTWDQVR	0	0
A0QS98	EAPVVRVSALKALEGDPKWVK	0	0
A0QS98	EDVERGQVVVKPGTTTPHTEF	0	0
A0QS98	EFEGQVYILSKDEGGRHTPFF	0	0
A0QS98	EIVGIRPETTKTTVTGVEMFR	0	0
A0QS98	GKTTLTAAITKVLHDKFPDLN	0	0
A0QS98	IGTIGHVDHGKTTLTAAITKV	0	0
A0QS98	________MAKAKFERTKPHV	0	0
A0QS98	______MAKAKFERTKPHVNI	0	0
A0QS98	_MAKAKFERTKPHVNIGTIGH	0	0
A0QS98	MPGDNTDISVKLIQPVAMDEG	0	0
A0QS98	NISHVEYQTDKRHYAHVDAPG	0	0
A0QS98	SALKALEGDPKWVKSVEELME	0	0
A0QS98	SIPDPVRETDKPFLMPVEDVF	0	0
A0QS98	TAAITKVLHDKFPDLNESRAF	0	0
A0QS98	TTVTGVEMFRKLLDQGQAGDN	0	0
A0QS98	VDAPGHADYIKNMITGAAQMD	0	0
A0QS98	DNVGLLLRGIKREDVERGQVV	0	0
A0QS98	GRTVGAGRVTKIIK_______	0	0
A0QS98	GVPYILVALNKSDAVDDEELI	0	0
A0QS98	VGAGRVTKIIK__________	0	0
A0QSE0	EDRKSHPLYGKIIRTTKKVKA	0	0
A0QSE0	GKIIRTTKKVKAHDENGEAGI	0	0
A0QSE0	KGPKYTPAAEKPRGRRKTAIG	0	0
A0QSE0	KTIVVELEDRKSHPLYGKIIR	0	0
A0QSE0	LMETRPLSATKRWRLVEILEK	0	0
A0QSE0	LYGKIIRTTKKVKAHDENGEA	0	0
A0QSE0	______MADQKGPKYTPAAEK	0	0
A0QSE0	___MADQKGPKYTPAAEKPRG	0	0
A0QSE0	PAAEKPRGRRKTAIGYVVSDK	0	0
A0QSE0	PLYGKIIRTTKKVKAHDENGE	0	0
A0QSE0	IGYVVSDKMQKTIVVELEDRK	0	0
A0QSE0	KTAIGYVVSDKMQKTIVVELE	0	0
A0QSE0	WRLVEILEKAK__________	0	0
A0QSH8	DTDEVIRNRMKVYREETEPLL	0	0
A0QSH8	GDGTPLGLEAKRYLDAGDLVP	0	0
A0QSH8	LKDMLAARNTKLDAVLEFQVS	0	0
A0QSH8	PLLEYYRDDLKTVNAVGALDE	0	0
A0QSH8	PQISTGDLFRKNIGDGTPLGL	0	0
A0QSH8	PRSVEQAGALKDMLAARNTKL	0	0
A0QSH8	VSEDELLTRLKGRGRADDTDE	0	0
A0QSH8	VVLLGPPGAGKGTQAEKLSEK	0	0
A0QSH8	PGAGKGTQAEKLSEKLGIPQI	0	0
A0QSL6	AQAKKGGTAAKKGQKTRRREK	0	0
A0QSL6	ARKAQEHGVKKVDVFVKGPGS	0	0
A0QSL6	HGVKKVDVFVKGPGSGRETAI	0	0
A0QSL6	KGGTAAKKGQKTRRREKKNVP	0	0
A0QSL6	KGQKTRRREKKNVPHGAAHIK	0	0

A0QSL6	KKGQKTRRREKKNVPHGAAHI	0	0
A0QSL6	______MAQAKKGGTAAKKGQ	0	0
A0QSL6	_____MAQAKKGGTAAKKGQK	0	0
A0QSL6	QAKKGGTAAKKGQKTRRREKK	0	0
A0QSL6	AARKAQEHGVKKVDVFVKGPG	0	0
A0QSL6	AQLAAENAARKAQEHGVKKVD	0	0
A0QSL6	AWASSGHVGFKGSRKSTPFAA	0	0
A0QSL6	KNVPHGAAHIKSTFNNTIVSI	0	0
A0QSL6	PQPHNGCRPPKRRRV______	0	0
A0QSU4	AGYCDLKEFQKVGLTVGS___	0	0
A0QSU4	GDIHSSGDLAKAIACGADAVV	0	0
A0QSU4	IVPSRRTRSSKDVSTAWQLDA	0	0
A0QSU4	LVGGLRRSMAKAGYCDLKEFQ	0	0
A0QSU4	SMAKAGYCDLKEFQKVGLTVG	0	0
A0QSU4	VASDGEPLNLKTFISELDVPV	0	0
A0QSU4	LIGRHADVEEKIAQVVEVAAK	0	0
A0QUV7	AQAENATRITKREPAVAGDRP	0	0
A0QUV7	GTAAGLTDGLKAAGAAKIYVA	0	0
A0QUV7	GVIIAANADGKEIAGRLAARI	0	0
A0QUV7	IQHRAGMQTSKTIVAVNKDEE	0	0
A0QUV7	LGEPSAVVVGKPGTAAGLTDG	0	0
A0QUV7	MLVEHADGALKKVSAELLTAA	0	0
A0QUV7	PGQFQVGQTGKTVSPQLYIAL	0	0
A0QUV7	QTSKTIVAVNKDEEAPIFEIA	0	0
A0QUV7	ADLGIVGDLFKVSPQLTEAVK	0	0
A0QUV7	KVSPQLTEAVKARKG______	0	0
A0QUV7	LVEHADGALKKVSAELLTAAR	0	0
A0QUV7	PQLTEAVKARKG_________	0	0
A0QUV7	SGLLVDVIEVKEGGVGVHSIF	0	0
A0QUV7	TDGLKAAGAAKIYVAESDDVE	0	0
A0QUX7	KQLNKLINVIKIVEQEEDNSV	0	0
A0QUX7	LTIEATGTPEKLEALLRVLEP	0	0
A0QUX7	NSVSRELALIKVRADATTRGQ	0	0
A0QUX7	THTLSVLVEDKPGVLARVSSL	0	0
A0QUX7	IIEAVNLFRAKVVDVSTESLT	0	0
A0QUX7	PLEQITKQLNKLINVIKIVEQ	0	0
A0QUX7	SRGPRGIGAAK__________	0	0
A0QUX7	VEDSPLEQITKQLNKLINVIK	0	0
A0QUY3	DAFARAQDRRKHLTLVHKTNV	0	0
A0QUY3	DRRKHLTLVHKTNVLAFAGSL	0	0
A0QUY3	_____MSSNLKLAVIAGDGIG	0	0
A0QUY3	SEHLATRGDEKLSTSAVGERI	0	0
A0QUY3	SGVLERGMLLKLRFALDHHVN	0	0
A0QUY3	VLDAVLPGVEKTEYDLGARRY	0	0
A0QUY9	APDETTFEFLKGRPHAPKGAD	0	0
A0QUY9	ATQTLSLKPFKTMAVNVDGEL	0	0
A0QUY9	AVAAATAVRGKLASPADLPAA	0	0
A0QUY9	DGELPPGVSAKDIILAVIAKI	0	0

A0QUY9	DHNVPTIDIDKPIADPVSRTQ	0	0
A0QUY9	EFLKGRPHAPKGADWDAAVAA	0	0
A0QUY9	HVMATQTLSLKPFKTMAVNVD	0	0
A0QUY9	_MDQPTQTSVKPRTLAEKVWD	0	0
A0QUY9	SNRNFEGRQGKGGRTHLVSPA	0	0
A0QUY9	TSVKPRTLAEKVWDDHVVARG	0	0
A0QUY9	AKDIILAVIAKIGTGGGQGHV	0	0
A0QUY9	VARGEGEGTAKEPDLIYIDLH	0	0
A0QUY9	VDEGARQAAEKALAYMDLRPG	0	0
A0QUZ0	EAYEAARPAWKPRTLPA____	0	0
A0QUZ0	GLDDIGLTLRKLDQIEAYEAA	0	0
A0QUZ0	TDQIIPAVYLKRVTRTGFEDG	0	0
A0QUZ0	FADIFRGNAGKAGLLAAEVAQ	0	0
A0QUZ0	VAQDDVELLWKVIEQNPGLEI	0	0
A0QV10	HIQLGNIVIPKSVNPERIASN	0	0
A0QV10	LIHWPVPENNKFVDTFKAFAH	0	0
A0QV10	PENNKFVDTFKAFAHLRDQGR	0	0
A0QV10	PQQELRDVHAKLGIATEAWSP	0	0
A0QV10	VITGIAEQHGKTPAQVLIRWH	0	0
A0QV10	VPREDIFLVTKLWNSDQGYDA	0	0
A0QVB9	AAKANDIETLKAAKTGDTTVE	0	0
A0QVB9	AVELLRIKGAKDVGKRAERAT	0	0
A0QVB9	DQIVAAAVAAKANDIETLKAA	0	0
A0QVB9	ELNSETDFVAKNAEFQALADQ	0	0
A0QVB9	ELTGAGMLDSKNALVEADGDF	0	0
A0QVB9	EYQAGDADKGKEAAHAVALQI	0	0
A0QVB9	IAEETARNEGKPEQALPKIVE	0	0
A0QVB9	IVEGRVTGFYKDVVLLDQPSV	0	0
A0QVB9	LDQPSVSDNKKTVKALLDEAG	0	0
A0QVB9	LLDQPSVSDNKKTVKALLDEA	0	0
A0QVB9	LRIKGAKDVGKRAERATAEGL	0	0
A0QVB9	LVEYQAGDADKGKEAAHAVAL	0	0
A0QVB9	_MANYTAADVKRLRELTGAGM	0	0
A0QVB9	NEGKPEQALPKIVEGRVTGFY	0	0
A0QVB9	PSVSDNKKTVKALLDEAGVTV	0	0
A0QVB9	FDGTVEAYLHKRAADLPPAVG	0	0
A0QVB9	FDKAVELLRIKGAKDVGKRAE	0	0
A0QVB9	HAVALQIAALKAKYLTREDVP	0	0
A0QVB9	RATAEGLVAAKDGALIELNSE	0	0
A0QVB9	VALQIAALKAKYLTREDVPED	0	0
A0QVB9	VEQAIADLSAKIGEKLELRRA	0	0
A0QVM0	AADTLKNVLLKTREPGGEWEL	0	0
A0QVM0	ADFAANPFLVKGYVGPKALQD	0	0
A0QVM0	DPADAEVPSHKLLIRAGYVRA	0	0
A0QVM0	EIGHIFQLGRKYTDAFSADVL	0	0
A0QVM0	FDDRQASPGVKFKDAELLGMP	0	0
A0QVM0	FPVILYQIQTKYRDEARPRAG	0	0
A0QVM0	FSADVLGEDGKPLRLTMGSYG	0	0

A0QVM0	GILRGREFVMKDSYSFDVDDD	0	0
A0QVM0	GRTVTAADTLKNVLLKTREPG	0	0
A0QVM0	GVPGDREVDEKRLGAALEPAE	0	0
A0QVM0	LTVKGEYSSYKDFPVILYQIQ	0	0
A0QVM0	PFLVKGYVGPKALQDNGVRYL	0	0
A0QVM0	AQPTEGLPEAKVYDTPDTPTI	0	0
A0QVM0	PFDVHVVVANKDAGARAGAAE	0	0
A0QVM0	WLPLGLRVLRKIENVVRSEMN	0	0
A0QW25	________MAKLDFDALNSTI	0	0
A0QW25	SEHGMAARGYKDVRANTVPAF	0	0
A0QW25	VALHRPAEFNKSHIPAFLAGE	0	0
A0QW25	AATFLKQQEDKGVVVRGLYDV	0	0
A0QW25	PRISVENLIAKLP________	0	0
A0QWG8	ADYTNHIDKWKFTVPFVCGAT	0	0
A0QWG8	DVSNATTHMRKIGGEIRRLTS	0	0
A0QWG8	EGVFVGSGIFKSGNPEQRAAA	0	0
A0QWG8	IDAVTIPVMAKARIGHFVEAQ	0	0
A0QWG8	ITEGAAMIRSKGEAGTGDVSN	0	0
A0QWG8	MSEDELYVAAKELQAPYELVV	0	0
A0QWG8	NPEQRAAAIVKATTFYDDPDV	0	0
A0QWG8	RVKRGMAEMLKGGVIMDVVTP	0	0
A0QWG8	SSNQTGTARVKRGMAEMLKGG	0	0
A0QWG8	TPADYTNHIDKWKFTVPFVCG	0	0
A0QWG8	ELVVEVARAGKLPVTLFTAGG	0	0
A0QWT3	ARHGGGAFSGKDPSKVDRSAA	0	0
A0QWT3	DIELPWEQTNKVDDLKSAI__	0	0
A0QWT3	DTAHETRVEGKADPLDLQGAG	0	0
A0QWT3	DYRLLVNPTGKFVLGGPMGDA	0	0
A0QWT3	GGAFSGKDPSKVDRSAAYAMR	0	0
A0QWT3	GVLDYLRPDGKTQVTIQYDGT	0	0
A0QWT3	ILDIGYDSSTKGFDGASCGVN	0	0
A0QWT3	PMGDAGLTGRKIIVDTYGGWA	0	0
A0QWT3	RLARRLTEVRKNGVLDYLRPD	0	0
A0QWT3	SAAYAMRWVAKNVVAAGLAER	0	0
A0QWT3	SESVTEGHPDKICDAISDSVL	0	0
A0QWT3	VEVQVAYAIGKAAPVGLFVET	0	0
A0QWT3	EGTLTPDIREKVVNTVLADLG	0	0
A0QWT3	________MSKGRLFTSESVT	0	0
A0QWT3	SETVDPAKIEKAIGEVFDLRP	0	0
A0QWT3	VLDALLEQDPKSRVAVETLVT	0	0
A0QWT3	VTTTAYADIPKIVRDRILDIG	0	0
A0QWV9	DLGIIARRLRKDIYDLYGYNA	0	0
A0QWV9	IRRLLSMADRKAKQEGIPDTE	0	0
A0QWV9	LGRLADPPMTKDAVAGRIRRL	0	0
A0QWV9	_____MTAEVKDELSRLVVNS	0	0
A0QWV9	RLLSMADRKAKQEGIPDTESA	0	0
A0QWV9	SVPDHLAAAGKLRVEHRQASL	0	0
A0QWV9	VHVLSASGIRKNTRYVVRVAN	0	0

A0QWW2	AAEGPLKGILKYYDAPIVSSD	0	0
A0QWW2	AKGHLDAGAKKVIISAPATDE	0	0
A0QWW2	AMKAAAEGPLKGILKYYDAPI	0	0
A0QWW2	ASVEDINAAMKAAAEGPLKGI	0	0
A0QWW2	CTTNCLGPLAKVLNDEFGIVK	0	0
A0QWW2	DTIVIGDTKIKALEVKEGPAA	0	0
A0QWW2	ESTGIFTNAAKAKGHLDAGAK	0	0
A0QWW2	GDDTIVIGDTKIKALEVKEGP	0	0
A0QWW2	GDTKIKALEVKEGPAALPWGD	0	0
A0QWW2	GLTKVIDNQAKVVSWYDNEWG	0	0
A0QWW2	IGLVLPELKGKLDGYALRVPI	0	0
A0QWW2	ITIVLGVNDDKYDGSQNIISN	0	0
A0QWW2	KAIGLVLPELKGKLDGYALRV	0	0
A0QWW2	KAKGHLDAGAKKVIISAPATD	0	0
A0QWW2	KVLNDEFGIVKGLMTTIHAYT	0	0
A0QWW2	NIVPTSTGAAKAIGLVLPELK	0	0
A0QWW2	RALATQKAEGKNTDIEIVAVN	0	0
A0QWW2	RLADLVALVGKSL________	0	0
A0QWW2	RNFYRALATQKAEGKNTDIEI	0	0
A0QWW2	TDNATLAHLLKFDSILGRLPQ	0	0
A0QWW2	TGIFTNAAKAKGHLDAGAKKV	0	0
A0QWW2	HSSLYDAGLTKVIDNQAKVVS	0	0
A0QWW2	QDQNLQDGPHKDLRRARAAAL	0	0
A0QWX9	APNTVNTMPEKTLEAVADHGE	0	0
A0QWX9	DAYLEGLEKAKEAGHDLSKIH	0	0
A0QWX9	DVRNACDLLAKEYEASDGVDG	0	0
A0QWX9	EEVFGSDRFAKLKADGARVQR	0	0
A0QWX9	GSDEALALRGKAGVANARLAY	0	0
A0QWX9	KAKEAGHDLSKIHSVASFFVS	0	0
A0QWX9	KIVDRPNLLIKIPATMAGLPA	0	0
A0QWX9	NPSIFQAALSKGTAYDAQVNE	0	0
A0QWX9	VDPRLAHDTDKTILQAIELWK	0	0
A0QWX9	VFGSDRFAKLKADGARVQRPL	0	0
A0QWX9	VMDAYLEGLEKAKEAGHDLSK	0	0
A0QWX9	ATQGQLDAAKK__________	0	0
A0QWX9	DATQGQLDAAKK_________	0	0
A0QWX9	FRVLEDEGVEKFEKSWQELLD	0	0
A0QWX9	LEDEGVEKFEKSWQELLDATQ	0	0
A0QWX9	QRPLWASTGVKNPEYSDTLYV	0	0
A0QWX9	TAASSQETFDKLAAIGIDLPD	0	0
A0QX20	AALLAKKAVEKGLTTKPWVKT	0	0
A0QX20	AGIPLVVLGGKEYGSGSSRDW	0	0
A0QX20	AVAALGGDPNKVNPLAPAELV	0	0
A0QX20	DMLRRHGVVGKFVEFYGKGVA	0	0
A0QX20	EKGLTTKPWVKTNMAPGSQVV	0	0
A0QX20	EQLALVEAYAKAQGMWHDPER	0	0
A0QX20	EWDPASTYVRKAPYFDGMPAE	0	0
A0QX20	FIYDASENYKKAGIPLVVLGG	0	0

A0QX20	FPAGESAASLKLDGTETYDIE	0	0
A0QX20	GSGSSRDWAAKGTVLLGVKAV	0	0
A0QX20	GSQVVTDYYNKAGLWPYLEKL	0	0
A0QX20	HVTATKEDGSKVEFDAVVRID	0	0
A0QX20	IEELNSGKTPKTVHVTATKED	0	0
A0QX20	IEGIEELNSGKTPKTVHVTAT	0	0
A0QX20	ILQYVLRNMLKSSK_______	0	0
A0QX20	KKAVEKGLTTKPWVKTNMAPG	0	0
A0QX20	LRTEDGANITKDHIEAIANWD	0	0
A0QX20	MFTESYADVFKGDDRWRSLPT	0	0
A0QX20	___MSSENTGKSSLNSFGARD	0	0
A0QX20	NFEGRISPDVKMNYLASPPLV	0	0
A0QX20	NKAGLWPYLEKLGYYLGGYGC	0	0
A0QX20	PAEPEPVSDIKGARVLALLGD	0	0
A0QX20	PGTEKLPYSLKVLAENLLRTE	0	0
A0QX20	QYLDANGVERKDYNSLGSRRG	0	0
A0QX20	RWGQGAFDDFKVVPPGTGIVH	0	0
A0QX20	SMLIPRVVGFKLSGEIKPGVT	0	0
A0QX20	STVVPSISGPKRPQDRIELTD	0	0
A0QX20	SVMLGAALLAKKAVEKGLTTK	0	0
A0QX20	TDHISPAGAIKPGTPAAQYLD	0	0
A0QX20	TPKTVHVTATKEDGSKVEFDA	0	0
A0QX20	VMLGAALLAKKAVEKGLTTKP	0	0
A0QX20	VVGFKLSGEIKPGVTATDVVL	0	0
A0QX20	VVGKFVEFYGKGVAEVPLANR	0	0
A0QX20	YRLNAVPGTEKLPYSLKVLAE	0	0
A0QX20	YVLRNMLKSSK__________	0	0
A0QX20	AAKGTVLLGVKAVITESFERI	0	0
A0QX20	AFIYDASENYKKAGIPLVVLG	0	0
A0QX20	NTGPLPEEISKAINDNDLAVT	0	0
A0QX20	PQDRIELTDAKNAFRKDIHNY	0	0
A0QX20	VEQNHPTPETKLDEAVEESFP	0	0
A0QX81	_____MNMMAKFQMSRIDLRN	0	0
A0QX81	DVSGHVITLSKAEDLPAHGEA	0	0
A0QX81	GPGNIYVTAAKRICRSQVGID	0	0
A0QX81	HVVEYDEAALKDVSGHVITLS	0	0
A0QX81	RELANQLATTKHVERVTAALS	0	0
A0QX81	VERVTAALSGKQSAIVLVDDI	0	0
A0QX93	DALRATLDLLKTEAMAGLPPL	0	0
A0QX93	DAVARLDVMTKALGQPLTSAV	0	0
A0QX93	DSETPLSAYRKLAANRPGTFL	0	0
A0QX93	FPAGTLSGAPKVRAMELIEEV	0	0
A0QX93	RAMELIEEVEKTRRGLYGGVV	0	0
A0QX93	TMEEYTEIVDKLVGDIEAGEA	0	0
A0QX93	GPYEYTEAANKARAVLNAIAA	0	0
A0QX93	GSSPEALVTVKDGRATTHPIA	0	0
A0QX93	LLEKELLADEKERAEHLMLVD	0	0
A0QX93	STVTGELAEDKTALDAVTACF	0	0

A0QX93	TEHRVVPVIRKVLADSETPLS	0	0
A0QXX7	ALIVGGHTFGKTHGAGDASLV	0	0
A0QXX7	APIEQQGLGWKSSYGTGKGPD	0	0
A0QXX7	ARRLLWPIKQKYGNKISWADL	0	0
A0QXX7	ASAASYRNTDKRGGANGARVR	0	0
A0QXX7	DFNASASGGKKVSLADLIVLA	0	0
A0QXX7	DKARRLLWPIKQKYGNKISWA	0	0
A0QXX7	DSGLSTGQLIKTAWASAASYR	0	0
A0QXX7	EDFDYAEEFAKLDVNALKADL	0	0
A0QXX7	EFAKLDVNALKADLTALMTQS	0	0
A0QXX7	EGVDRKTGQLKWTATANDLVF	0	0
A0QXX7	ENPAIPSPKPKSGAPLRNQDW	0	0
A0QXX7	ESENPAIPSPKPKSGAPLRNQ	0	0
A0QXX7	FVNDFVKAWVKVMNNDRFDLK	0	0
A0QXX7	GEEDEWLGTDKRYGGGEQRQL	0	0
A0QXX7	GFRNYVRPGEKVQLEKMLLER	0	0
A0QXX7	GNVALESMGFKTFGFGFGRED	0	0
A0QXX7	ILYGYEWELTKSPAGAWQFTA	0	0
A0QXX7	ISMRVDPIYGKITRRWLDHPE	0	0
A0QXX7	KVMNNDRFDLK__________	0	0
A0QXX7	LEVVWTNTPTKWDNSFLEILY	0	0
A0QXX7	LGWKSSYGTGKGPDTITSGLE	0	0
A0QXX7	LVDDQDIAALKSTVLDSGLST	0	0
A0QXX7	LWPIKQKYGNKISWADLLVFT	0	0
A0QXX7	NGARVRLEPQKNWDVNEPAEL	0	0
A0QXX7	QDFNASASGGKKVSLADLIVL	0	0
A0QXX7	RALDVNHGGTKHGVFTDRPGA	0	0
A0QXX7	SPAGAWQFTAKDGAGAGTIPD	0	0
A0QXX7	TENVYEGVDRKTGQLKWTATA	0	0
A0QXX7	VNLLDMGTEWKTSETTENVYE	0	0
A0QXX7	VRPGEKVQLEKMLLERAYFLG	0	0
A0QXX7	AGSAAIEKAAKDGGYNVTVPF	0	0
A0QXX7	HPEELSEAFAKAWYKLLHRDM	0	0
A0QXX7	IVLAGSAAIEKAAKDGGYNVT	0	0
A0QXX7	LSEAFAKAWYKLLHRDMGPIS	0	0
A0QXX7	NGERFVNDFVKAWVKVMNNDR	0	0
A0QXX7	SDSRPPNPDTKTASTSESENP	0	0
A0QYD4	SIVNVLPESAKEGSVEAAIKA	0	0
A0QYD4	AKEGSVEAAIKAALSDWTAGQ	0	0
A0QYD4	VNVPAPRWEAKDPRTYTLADL	0	0
A0QYS9	FKITRLTGKTKIVAAEGAFHG	0	0
A0QYS9	GHPLVDKVRGKGLLQGIVLTV	0	0
A0QYS9	GIVPDVVTMAKGLGGGLPIGA	0	0
A0QYS9	GSLALTGQPSKQAPFEPLPGN	0	0
A0QYS9	IEELGHPLVDKVRGKGLLQGI	0	0
A0QYS9	SGTEANEVAFKITRLTGKTKI	0	0
A0QYS9	VAFKITRLTGKTKIVAAEGAF	0	0
A0QYS9	VCTAAGLAVLKTLAAEDLVAR	0	0

A0QYS9	QGIVLTVPSAKAVETAARDAG	0	0
A0QYS9	YLVAAREITSKHGALLVLDEV	0	0
A0QZ54	AIKSVLETGQKGLRIQHLLDS	0	0
A0QZ54	AILRPGRLDVKIKIERPDAEA	0	0
A0QZ54	AKAVANSLAKKMAEVRGDDAR	0	0
A0QZ54	ANGDKEVMYFKDFNSGAMIQN	0	0
A0QZ54	ARNAKLMDTLKEARQQLLALR	0	0
A0QZ54	CSPNIEVKELKQGQTVRLNEA	0	0
A0QZ54	DTVDVFTSGRKMRLTCSPNIE	0	0
A0QZ54	EFNGDRALCIKAMIEKVVDRM	0	0
A0QZ54	ELPFLHKDLYKEYSLRPPKGV	0	0
A0QZ54	EVRGDDAREAKSYFLNIKGPE	0	0
A0QZ54	FLEVTYANGDKEVMYFKDFNS	0	0
A0QZ54	IAKAVANSLAKKMAEVRGDDA	0	0
A0QZ54	LNIKGPELLNKFVGETERHIR	0	0
A0QZ54	LYKEYSLRPPKGVLLYGPPGC	0	0
A0QZ54	NPDDWARISGKKGERIVYIRT	0	0
A0QZ54	PDDWARISGKKGERIVYIRTL	0	0
A0QZ54	PPGCGKTLIAKAVANSLAKKM	0	0
A0QZ54	RALCIKAMIEKVVDRMYAEID	0	0
A0QZ54	RDAVELPFLHKDLYKEYSLRP	0	0
A0QZ54	REAKSYFLNIKGPELLNKFVG	0	0
A0QZ54	RIDSLAARNAKLMDTLKEARQ	0	0
A0QZ54	RLTCSPNIEVKELKQGQTVRL	0	0
A0QZ54	VLLYGPPGCGKTLIAKAVANS	0	0
A0QZ54	AMIQNVVDRAKKYAIKSVLET	0	0
A0QZ54	DAEAAQDIFSKYLTEDLPVHA	0	0
A0QZ54	IRLIFQRAREKASEGTPVIVF	0	0
A0QZ54	KAGYAFERIPKAEVEDLVLEE	0	0
A0QZ54	LRPGRLDVKIKIERPDAEAAQ	0	0
A0QZ54	MIQNVVDRAKKYAIKSVLETG	0	0
A0QZ54	RPGDSLLVDTKAGYAFERIPK	0	0
A0QZ54	TDYFDDSRPRKLRPGDSLLVD	0	0
A0QZ54	VVDRAKKYAIKSVLETGQKGL	0	0
A0QZ54	VWLAEPLVAAKDLPDEPTDYF	0	0
A0QZA1	AGWQERYPDVKVHRRVERAQP	0	0
A0QZA1	QPAYWLLEEAKQAQLVVVGSH	0	0
A0QZA1	HGLTVQTEIRKESVARTLIDA	0	0
A0QZA1	NARHAIEQAQKVVADCLGETH	0	0
A0QZE3	DWFNEEGSRFKPSMMGSAVFT	0	0
A0QZE3	GLVDRTWAANKYELNVVGIQG	0	0
A0QZE3	IAEAEIRAGVKVEHRKAHVWP	0	0
A0QZE3	IRAGVKVEHRKAHVWPGHHYQ	0	0
A0QZE3	TRAGHDSTNMKEIVPSVMLFV	0	0
A0R066	AAVITPVSHVKHHDGEFTIAD	0	0
A0R066	ESILANPGFGKYYTDHMVSID	0	0
A0R066	HYGQEIFEGLKAYRWADGSIV	0	0
A0R066	LIASPAGAYFKGGIKPVSVWL	0	0

A0R066	PAGAYFKGGIKPVSVWLSHEY	0	0
A0R066	SLRQLIAVDEKWVPPAGGEES	0	0
A0R066	TDAGFAVEERKIDVDEWQKKA	0	0
A0R066	RASPGGTGAAKFGGNYAASLL	0	0
A0R066	RKIDVDEWQKKAGAGEITEVF	0	0
A0R079	AFTNPTVNSYKRLVPGYEAPI	0	0
A0R079	AMLMAGIDGIKKKIEPLQPVD	0	0
A0R079	EDLIETWISYKRENEIMPIQI	0	0
A0R079	ESDGSANRGYKVRPKGGYFPV	0	0
A0R079	GTAGQAEINYKFNTLLAAADD	0	0
A0R079	IIKNTAWQAGKTVTFMPKPLF	0	0
A0R079	IPITGNNPKAKRLEFRCPDSS	0	0
A0R079	KKIEPLQPVDKDLYELPPDEA	0	0
A0R079	LMAGIDGIKKKIEPLQPVDKD	0	0
A0R079	_______MAEKTSDDIFKLIK	0	0
A0R079	MAEKTSDDIFKLIKDENVEYV	0	0
A0R079	MLMAGIDGIKKKIEPLQPVDK	0	0
A0R079	QAGKTVTFMPKPLFGDNGSGM	0	0
A0R079	SANRGYKVRPKGGYFPVAPYD	0	0
A0R079	SGMHAHQSLWKDGQPLFHDES	0	0
A0R079	TARIDPFRAAKTLNMNFFVHD	0	0
A0R079	VRIPITGNNPKAKRLEFRCPD	0	0
A0R079	YIFDSVSFDSKINGTFYEVDS	0	0
A0R079	APTSLSAVIDKLEEDHEYLTE	0	0
A0R079	DDVLLFKYIIKNTAWQAGKTV	0	0
A0R079	LAAADDVLLFKYIIKNTAWQA	0	0
A0R079	YSRDPRNVARKAENYLASTGI	0	0
A0R083	ADGVRASFMPKPFAEHPGSAM	0	0
A0R083	AVTNQWVNSYKRLVHGGEAPT	0	0
A0R083	DDPLQLSDVAKSFIAGILEHA	0	0
A0R083	DNVMTFRYLVKEVALADGVRA	0	0
A0R083	DPSTFQVLPWKTSDGNHYSAR	0	0
A0R083	EHVFDYFLRNKRSEWENYRSH	0	0
A0R083	LVRVPMYTPHKVSSRRVEVRS	0	0
A0R083	LWFTDVLGYLKSVAIAPAELE	0	0
A0R083	______MDRQKEFVLRTLEER	0	0
A0R083	VHPEIEFFLLKPGPNDGTPPE	0	0
A0R083	YRSHVTPYELKNYLSL_____	0	0
A0R083	SRHVLRRQLAKASDLGFTCYV	0	0
A0R0B0	ATTMATVRTFKELLRDKNIGP	0	0
A0R0B0	DMSDQEIWNLKRGGHDYRKVY	0	0
A0R0B0	EGRNATHQMKKLALEDLKNFR	0	0
A0R0B0	ELNREGVAIEKHRLRHPDEPA	0	0
A0R0B0	EVGFNHFFRGKSHPGGGDHVF	0	0
A0R0B0	FEGRNATHQMKKLALEDLKNF	0	0
A0R0B0	FFRGAGWNVIKVVWGREWDVL	0	0
A0R0B0	FGMDSWFPSLKIYNRNGQLYT	0	0
A0R0B0	FNHYLHDRGIKDTSDQHVWAF	0	0

A0R0B0	GFVPSRRTKSKPLALPGSDTY	0	0
A0R0B0	GLYLYRPAPEKRAKSAQILAS	0	0
A0R0B0	HKGQPTVILAKTIKGYTLGQH	0	0
A0R0B0	LGGFVPSRRTKSKPLALPGSD	0	0
A0R0B0	LPGSDTYKALKKGSGSQAVAT	0	0
A0R0B0	LYRPAPEKRAKSAQILASGVA	0	0
A0R0B0	NLKRGGHDYRKVYAAYRAAME	0	0
A0R0B0	PENLDVEALLKGLYLYRPAPE	0	0
A0R0B0	PGSDTYKALKKGSGSQAVATT	0	0
A0R0B0	PLALPGSDTYKALKKGSGSQA	0	0
A0R0B0	QMKKLALEDLKNFRDVTRVPV	0	0
A0R0B0	QPTVILAKTIKGYTLGQHFEG	0	0
A0R0B0	RLDGPVRGNGKIIQELESFFR	0	0
A0R0B0	STPDGDYQTYKANDGAYVRDH	0	0
A0R0B0	SVDSELMLAYKESEVGQILHE	0	0
A0R0B0	VRTFKELLRDKNIGPRIVPII	0	0
A0R0B0	YAAYRAAMEHKGQPTVILAKT	0	0
A0R0B2	AGVISADDAVKLAATRGAEMA	0	0
A0R0B2	AMEKLVAQLTKPVRWDLCTAT	0	0
A0R0B2	ATLLAHEELTKRGHSAAETIV	0	0
A0R0B2	ELKGTPTRAIKSPEDLDGLDQ	0	0
A0R0B2	LAATRGAEMAKACAVEPTGMA	0	0
A0R0B2	PPAGTLVGIAKRELKGTPTRA	0	0
A0R0B2	PVASAADAMEKLVAQLTKPVR	0	0
A0R0B2	TLVGIAKRELKGTPTRAIKSP	0	0
A0R0B2	EDPPAKARVRKLATAGAFHTH	0	0
A0R0B2	LDKLAEDPPAKARVRKLATAG	0	0
A0R0B3	GIEPSEVTPEKSFVDDLDIDS	0	0
A0R0B3	MVEIAVQTEDKYGVKIPDEDL	0	0
A0R0B3	NPEAAAALREKFAADQ_____	0	0
A0R0B4	ADGLRAGQAMKRAMETAGLDP	0	0
A0R0B4	DPQGASRPFDKDRDGFVFGEA	0	0
A0R0B4	GVEHAAVYAPKSALGHSIGAV	0	0
A0R0B4	MMIIETEEHAKARGAKPLARL	0	0
A0R0B4	RAMETAGLDPKDIDHVNAHAT	0	0
A0R0B4	RRMSYVQRMSKFLSKQLWENA	0	0
A0R0B4	SIAPDIESTWKGLLAGESGIR	0	0
A0R0B4	TEEHAKARGAKPLARLMGAGI	0	0
A0R0B4	VAGEPRYGEYKYAINNSFGFG	0	0
A0R0B4	VIGTGLGGGEKIVETYDAMNE	0	0
A0R0B4	YDAMNEGGPRKVSPLAVQMIM	0	0
A0R0B4	YVQRMSKFLSKQLWENAGAPE	0	0
A0R0B5	AAAIGLERQAKAGVCTSISAC	0	0
A0R0B5	ALATDAESTWKKLLDGQSGIR	0	0
A0R0B5	DIAICGGVETKIEAVPIAGFA	0	0
A0R0B5	DPEGACKPFDKDRNGFVFGEA	0	0
A0R0B5	GGHKPAVYAPKGALGHSVGAV	0	0
A0R0B5	GTNVGDVAESKAINNAMGGHK	0	0

A0R0B5	KAINNAMGGHKPAVYAPKGAL	0	0
A0R0B5	LATDAESTWKKLLDGQSGIRT	0	0
A0R0B5	LMVIETEEHAKARGANILGRL	0	0
A0R0B5	MTRAIELAGLKPTDIDHVNAH	0	0
A0R0B5	NSNDDPEGACKPFDKDRNGFV	0	0
A0R0B5	VAGEPRQGDYKYAINNSFGFG	0	0
A0R0B5	GGHNVALAFGKY_________	0	0
A0R0B5	LVFAYDGMRSKGLRAVSPLVV	0	0
A0R0W1	ARIQVADIGFKGQMVDDGNGP	0	0
A0R0W1	EKPAPKTRPAKRPRGEGQWAL	0	0
A0R0W1	FCKLSFAETRKRAQVLVPELE	0	0
A0R0W1	FREVLETEYLKRPLIDGPAPE	0	0
A0R0W1	FRWWGLYTQRKPGYDGTWTGD	0	0
A0R0W1	GKPEYSNLPRKFKTAISGLQD	0	0
A0R0W1	IDEIVRRYIGKPEYSNLPRKF	0	0
A0R0W1	IQVENMPEIWKRLDAVGLQTT	0	0
A0R0W1	KFLIKDWGVEKFREVLETEYL	0	0
A0R0W1	KLIILDVPDDKIDELRAGLDA	0	0
A0R0W1	LMACTGIEFCKLSFAETRKRA	0	0
A0R0W1	__MTSSEAPAKTAEKPAPKTR	0	0
A0R0W1	PAKTAEKPAPKTRPAKRPRGE	0	0
A0R0W1	PEYSNLPRKFKTAISGLQDVV	0	0
A0R0W1	PIDHVGVQKLKNGLNAVGVAP	0	0
A0R0W1	RDYGYRRLRSKARLKFLIKDW	0	0
A0R0W1	RSKARLKFLIKDWGVEKFREV	0	0
A0R0W1	SGFGRKLRQHKVLSSELGDYI	0	0
A0R0W1	SLGLDSGFGRKLRQHKVLSSE	0	0
A0R0W1	SSEAPAKTAEKPAPKTRPAKR	0	0
A0R0W1	TRPIDHVGVQKLKNGLNAVGV	0	0
A0R0W1	YAKNGFESIDKGDLRGRFRWW	0	0
A0R0W1	YRRLRSKARLKFLIKDWGVEK	0	0
A0R0W1	AGRVSGTILTKVADLAEAAGS	0	0
A0R0W1	EPLNANEQAKKDDNPLNVRAR	0	0
A0R0W1	REPLNANEQAKKDDNPLNVRA	0	0
A0R0W1	SDRIRFTPYQKLIILDVPDDK	0	0
A0R0W1	VRARIENIYAKNGFESIDKGD	0	0
A0R0W1	YIERVVRNFVKQREDGERFAQ	0	0
A0R0W4	AFGLKIRKRPKAEIAERVDNL	0	0
A0R0W4	DNVAFGLKIRKRPKAEIAERV	0	0
A0R0W4	GFVFQHYAAFKHLSVRDNVAF	0	0
A0R0W4	SVRDNVAFGLKIRKRPKAEIA	0	0
A0R0W4	TALLGPSGSGKSTLLRAIAGL	0	0
A0R0W4	LDEPFGALDAKVRDDLRTWLR	0	0
A0R0W4	TSAIVVRGANKHYGDFAALDN	0	0
A0R1B5	DALKYGSRAAKARFAVYIGPL	0	0
A0R1B5	LGVSAAAASFKEGNLIDGLIS	0	0
A0R1B5	MDLVVLDDALKYGSRAAKARF	0	0
A0R1V9	FQWRAQHEDLKTLPFPMVSDL	0	0

A0R1V9	GKWRIIFFWPKDFTFVCPTEI	0	0
A0R1V9	QSDELCACNWKKGDPTINAGE	0	0
A0R1V9	SDELCACNWKKGDPTINAGEL	0	0
A0R1V9	TLPFPMVSDLKRELTAACGVL	0	0
A0R1V9	TRVTSKDYEGKWRIIFFWPKD	0	0
A0R1V9	VCPTEIAAFGKLNEDFEDRDA	0	0
A0R1V9	LNEDFEDRDAKVLGVSVDNEF	0	0
A0R1Y7	AAAVIVMNKAKAEELGLTWLA	0	0
A0R1Y7	DGAAAVIVMNKAKAEELGLTW	0	0
A0R1Y7	EVVPVSIPQRKGDPIEFAEDE	0	0
A0R1Y7	GAIAIRAALEKANVPASMVEY	0	0
A0R1Y7	HLLPKSREGYKYGDATLVDHL	0	0
A0R1Y7	IVAGARTPVGKLMGSLKDFSG	0	0
A0R1Y7	NTTAESLAGLKPAFRKDGTIT	0	0
A0R1Y7	PWDVAALSINKMCLSGIDAIA	0	0
A0R1Y7	QDEYAAQSHQKAAAAWKDGVF	0	0
A0R1Y7	SLAGLKPAFRKDGTITAGSAS	0	0
A0R1Y7	SMSQAPHLLPKSREGYKYGDA	0	0
A0R1Y7	STKELGVDPAKVNVNGGAIAI	0	0
A0R1Y7	TPVGKLMGSLKDFSGTDLGAI	0	0
A0R1Y7	AFAAVALASTKELGVDPAKVN	0	0
A0R1Y7	ALTEQRNDVDKFTRAEQDEYA	0	0
A0R1Y7	LQSQPANAIKKAITREGITVD	0	0
A0R1Y7	TLQSQPANAIKKAITREGITV	0	0
A0R218	ADKPAKEAQTKAAEAPEAGES	0	0
A0R218	AIDLLISQLRKTKTNYEFLVQ	0	0
A0R218	AKTERAEKADKPAKEAQTKAA	0	0
A0R218	AVDVNPSGTRKDELLLSPDEF	0	0
A0R218	DGQDGKQDQGKQDRSERDQDQ	0	0
A0R218	DLLISQLRKTKTNYEFLVQVS	0	0
A0R218	DRGEKDGQDGKQDQGKQDRSE	0	0
A0R218	EAKAEAPAEAKTERAEKADKP	0	0
A0R218	EAKTEAQPEAKAEAPAEAKTE	0	0
A0R218	EEVTDMQRSVKGEVIASTFDR	0	0
A0R218	EFKGTGNAELKLDRKIAERRV	0	0
A0R218	ERAEKADKPAKEAQTKAAEAP	0	0
A0R218	GDSGGQNQRQKFNPLVRLDTV	0	0
A0R218	GEKTEAQPEAKTEAQPEAKAE	0	0
A0R218	GVDSTALYPPKRFLGAARNIE	0	0
A0R218	IGVEGASGLRKSELIAAIRAH	0	0
A0R218	KGEAGEKAGEKTEAQPEAKTE	0	0
A0R218	LSPDEFAIVHKLRRVLSGLDS	0	0
A0R218	MVLPELRALAKEIGVEGASGL	0	0
A0R218	PAEAKTERAEKADKPAKEAQT	0	0
A0R218	PVAEKGEAGEKAGEKTEAQPE	0	0
A0R218	RVIDLIMPIGKGQRALIVSPP	0	0
A0R218	SDTGDLSGAVKSNTAPRARSA	0	0
A0R218	TGDTVIFEEFKGTGNAELKLD	0	0

A0R218	TGNAELKLDRKIAERRVFPAV	0	0
A0R218	TNYEFLVQVSKTAPGSMDVD_	0	0
A0R218	TRQAGAPVAEKGEAGEKAGEK	0	0
A0R218	ALIVSPPKAGKTTILQDIANA	0	0
A0R218	EDAKKRPEFGKLTPLYPNERL	0	0
A0R218	ERAKRLVEQGKDVVVLLDSIT	0	0
A0R218	GQRALIVSPPKAGKTTILQDI	0	0
A0R218	GRQDRADRGEKDGQDGKQDQG	0	0
A0R218	QAAELAIERAKRLVEQGKDVV	0	0
A0R218	TVNGGSVEDAKKRPEFGKLTP	0	0
A0R218	VNGGSVEDAKKRPEFGKLTPL	0	0
A0R220	ASAASIAGLLKSVEDGWVKRG	0	0
A0R220	GNAGNITAYWKGYSEYHRDGV	0	0
A0R220	ITCAVLIPQGKIAMGKLAQAV	0	0
A0R220	KLAQAVMHGAKIIQVDGNFDD	0	0
A0R220	LEGGTPLIHAKRISELTGCTV	0	0
A0R220	LIPQGKIAMGKLAQAVMHGAK	0	0
A0R220	LLKSVEDGWVKRGSTVVCTVT	0	0
A0R220	______MSAAKAAVHQPWPGL	0	0
A0R220	NGLKDPDTALKGMPQVTPVPV	0	0
A0R220	SELTGCTVHLKVEGLNPTGSF	0	0
A0R220	SVNPYRIEGQKTAAFEIVDAL	0	0
A0R220	VEGLNPTGSFKDRGMTVAVTE	0	0
A0R220	VVCTVTGNGLKDPDTALKGMP	0	0
A0R220	AAPLVTGAPVKDPETIATAIR	0	0
A0R2G5	GFETTRARLRKRGAVAVYGID	0	0
A0R2G5	GGGKEVIKVGKRCLLGANSGL	0	0
A0R2G5	RGAVAVYGIDKFPRMVDYVTP	0	0
A0R2G5	RLHLLSHRLTKPHEANLDGIF	0	0
A0R2G5	SIMGTLSGGGKEVIKVGKRCL	0	0
A0R2G5	TLSGGGKEVIKVGKRCLLGAN	0	0
A0R2G5	VPADLAALTGKDEDRDVEVVA	0	0
A0R2G5	VRTTIADLNDKPADTHDVWLR	0	0
A0R2G5	EAGLYVTGGTKVTTADGQVTK	0	0
A0R2G5	KVTTADGQVTKAIELSGASNL	0	0
A0R2V7	ATEEYAPAVDKGCPMVLGGPL	0	0
A0R2V7	DAVAAAAREFKPLVLVAGYSA	0	0
A0R2V7	EAPELANFGAKHINDLSEADW	0	0
A0R2V7	GGPLSHVMAAKAVALAEARQP	0	0
A0R2V7	HAHVTTTTTHKSLRGPRGGMV	0	0
A0R2V7	KELADQRDSLKLIASENYASP	0	0
A0R2V7	LANTQPEGTSKAKYKLADGTA	0	0
A0R2V7	LLTMGTWFSDKYAEGTIGHRF	0	0
A0R2V7	MAHFAGLVAGKVFTGDEDPVP	0	0
A0R2V7	NTQPEGTSKAKYKLADGTAER	0	0
A0R2V7	QPEGTSKAKYKLADGTAERVH	0	0
A0R2V7	SAYPRRVNFAKMREIADEVGA	0	0
A0R2V7	SEADWETLRNKLGNQRLLGMS	0	0

A0R2V7	THGFRPNISGKMFHQRSYGTN	0	0
A0R2V7	VEPRVAAATRKELADQRDSLK	0	0
A0R2W6	FDGDGRITSMKAYWSPADIKQ	0	0
A0R2W6	HDAAALESFVKRYLDTVAGGS	0	0
A0R2W6	MKAYWSPADIKQL________	0	0
A0R2W6	KNMTAATTDIKTELLSFRPGG	0	0
A0R342	ACLNAIEYLKKWGYTGEQAYL	0	0
A0R342	APCHMLSGPIKVEGAEPGDLL	0	0
A0R342	DIRPGGKGPQKVDRGQVAVTS	0	0
A0R342	EGWGYSGIFAKVNGGGFLTDY	0	0
A0R342	FLTDYYPDAYKAIWDFHGQQC	0	0
A0R342	GFIDMHVDLIKGGMETYGVTT	0	0
A0R342	IFDFDIRPGGKGPQKVDRGQV	0	0
A0R342	KPGSEFRIECKEWTDGQIGNN	0	0
A0R342	NACLNAIEYLKKWGYTGEQAY	0	0
A0R342	PEVVFSVDHSKSMRDQAVPGH	0	0
A0R342	PRENGGNHDIKNFTRGSRIFY	0	0
A0R342	GTAPSPDLLAKWNERERALIA	0	0
A0R3D2	GASRQARRDGKVPAVLYGHGT	0	0
A0R3D2	________MAKTANIPNKLTA	0	0
A0R3D2	_MAKTANIPNKLTANVRTRTG	0	0
A0R3D2	ADLLVVQRGEKVTVEVTVLVE	0	0
A0R3D2	IEGTEQLALTKALDVHPIRRN	0	0
A0R3D6	AALRRAVPATKGNTKRLASLG	0	0
A0R3D6	ADVEDFLDAGKPFGLRPEAIL	0	0
A0R3D6	DFLRDTLLWAKLIDKYKGTMT	0	0
A0R3D6	DTLLWAKLIDKYKGTMTAAPN	0	0
A0R3D6	EGNIVVCGRVKDVIIMAGRNI	0	0
A0R3D6	IPMYFGAELVKVTPMDFLRDT	0	0
A0R3D6	LLWAKLIDKYKGTMTAAPNFA	0	0
A0R3D6	MFVGAKYDVEKDVMVSWLPCF	0	0
A0R3D6	____MSRFTEKMYRNARTVTT	0	0
A0R3D6	QLTSGSTGSPKAVQITHRNIH	0	0
A0R3D6	RAVPATKGNTKRLASLGPLLN	0	0
A0R3D6	SNAEAMFVGAKYDVEKDVMVS	0	0
A0R3D6	TMNVIGMIELKAVIISEPFLV	0	0
A0R3D6	FLVATPVLEEKGILVLKVADL	0	0
A0R3D6	PGSIPKTPSGKLRRANSVSLV	0	0
A0R3D6	VLEEKGILVLKVADLLAADPI	0	0
A0R4C9	DVKLLDGGRKKWELDARPLSA	0	0
A0R4C9	DVRSPDEFSGKILAPAHLPQE	0	0
A0R4C9	DWAESNLKAPKTVFVEVDEDT	0	0
A0R4C9	GTFKSDEELAKLYAEAGLDGE	0	0
A0R4C9	IPGAINVPWSKAANEDGTFKS	0	0
A0R4C9	LQELLGHKNVKNYDGSWTEYG	0	0
A0R4C9	LYAEAGLDGEKETIAYCRIGE	0	0
A0R4C9	QDVKLLDGGRKKWELDARPLS	0	0
A0R4C9	VSTDWAESNLKAPKTVFVEVD	0	0

A0R4C9	WFAAYAYWYFKLYGHQDVKLL	0	0
A0R4C9	WFVLQELLGHKNVKNYDGSWT	0	0
A0R4C9	YDTGHIEGAVKLDWKTDLQDP	0	0
A0R4C9	YFKLYGHQDVKLLDGGRKKWE	0	0
A0R4C9	ELDARPLSAEKVERPQTSYTA	0	0
A0R4C9	HIEGAVKLDWKTDLQDPIRRD	0	0
A0R4C9	RDEVIAAIGTKNLVDVRSPDE	0	0
A0R4C9	SKAANEDGTFKSDEELAKLYA	0	0
A0R4C9	VERPQTSYTAKEPDNSIRAFR	0	0
A0R4D0	__MPAGVDLEKETVITGRVVD	0	0
A0R4Z5	AFAPVVLAWIKDLKADPEKVN	0	0
A0R4Z5	AVLLASEQAVKEHNLKPRARI	0	0
A0R4Z5	GPIPATEYALKKTGLSIDDID	0	0
A0R4Z5	IDAVRTPVGKKNGSLAQVHPI	0	0
A0R4Z5	IIDAVRTPVGKKNGSLAQVHP	0	0
A0R4Z5	LGHPLGATGAKLFATMLNTLE	0	0
A0R4Z5	PIPATEYALKKTGLSIDDIDT	0	0
A0R4Z5	PVVLAWIKDLKADPEKVNPNG	0	0
A0R4Z5	SEQAVKEHNLKPRARIHHISA	0	0
A0R4Z5	VPISSAMIVGKELGFSTPTAE	0	0
A0R4Z5	WIKDLKADPEKVNPNGGAIAL	0	0
A0R518	EVDVRDYDALKAVVDSGVEQL	0	0
A0R518	GIGNGGATLDKTSEADWDDMI	0	0
A0R518	IILTSSVGGLKAYPHTGHYIA	0	0
A0R518	___MAGRVEGKVAFITGAARG	0	0
A0R518	MIGVNLSGVWKTVKAAVPHLI	0	0
A0R518	PMTVDAGSMLK__________	0	0
A0R518	TPLFMNEGTMKLFRPDLENPG	0	0
A0R518	VNLSGVWKTVKAAVPHLISGG	0	0
A0R518	YPHTGHYIAAKHGVVGLMRTF	0	0
A0R566	GSIEYAVTVLKVPLIVVLGHD	0	0
A0R566	RVTPSILLGRKAGLSRVDEFE	0	0
A0R566	VLGHDSCGAVKATLSALDEGE	0	0
A0R566	SNPVAAWKALKDGNARFVAGQ	0	0
A0R5E1	KVEFEVGQSPKGPQATGVRTI	0	0
A0R5E1	____MPQGTVKWFNAEKGFGF	0	0
A0R5E1	QGTVKWFNAEKGFGFIAPEDG	0	0
A0R5M8	DSLLLGTDLVKDTGRLVRAYD	0	0
A0R5M8	DVRAGLTAAPKSLPPKWFYDA	0	0
A0R5M8	GEEMLTEVSCKFRPENVVAEL	0	0
A0R5M8	LTAAPKSLPPKWFYDAVGSDL	0	0
A0R5M8	LVELGSGTSEKTRMLLDAMRD	0	0
A0R5M8	VCGDFEEHLGKIPHVGRRLVV	0	0
A0R5R5	AEILEIFAAGKEPLKFTAYDG	0	0
A0R5R5	AVEEVGLPTAKVWGLYMAGSR	0	0
A0R5R5	EIFAAGKEPLKFTAYDGSSAG	0	0
A0R5R5	HNYPAYFNFLKSKLRTGGLLL	0	0
A0R5R5	IVRSIGIEHLKPIAPPPQEAL	0	0

A0R5R5	LRALAERMEFKRPPARVLANI	0	0
A0R5R5	______MTTFKERETSTADRK	0	0
A0R5R5	MVRYAARHGVKALGVTLSREQ	0	0
A0R5R5	PEDATMGLDLKTPRGTTYLAT	0	0
A0R5R5	QDNKYRLVFEKLRLKPGDRLL	0	0
A0R5R5	RRIMEGLRHSKTRDAEAIHHH	0	0
A0R5R5	VLAVKLDDQGKDGGLPLRPWW	0	0
A0R5R5	YPAYFNFLKSKLRTGGLLLNH	0	0
A0R5R5	YRLVFEKLRLKPGDRLLDVGC	0	0
A0R5R5	DATLEEAQDNKYRLVFEKLRL	0	0
A0R5R5	KERETSTADRKLTLAEILEIF	0	0
A0R5R5	VVQLHQVLAVKLDDQGKDGGL	0	0
A0R5R7	AFPYRKDPEDKNIRIAPTFPS	0	0
A0R5R7	ASADNIAWYLKHAGKKSIGPD	0	0
A0R5R7	EQLVASDPTIKGIWCVPVYSN	0	0
A0R5R7	ESKIASWTDPKGGYFVSLDVW	0	0
A0R5R7	GTAKRTVALAKDAGIAVTEAG	0	0
A0R5R7	HAGKKSIGPDKVNQLRHLRFF	0	0
A0R5R7	IAWYLKHAGKKSIGPDKVNQL	0	0
A0R5R7	KQLKLDLTRGKPSPEQLDLSN	0	0
A0R5R7	LAATEALLGDK__________	0	0
A0R5R7	MQRHRELIAPKFALVAEILED	0	0
A0R5R7	NIAWYLKHAGKKSIGPDKVNQ	0	0
A0R5R7	NRPLVFASTSKITFAGAGVSF	0	0
A0R5R7	NYAELQAKQLKLDLTRGKPSP	0	0
A0R5R7	PRPWSAEPTVKFLCPAPGYDR	0	0
A0R5R7	QQRNYAELQAKQLKLDLTRGK	0	0
A0R5R7	RRLVQMPTAAKDFRLMWDNAY	0	0
A0R5R7	TEAGSAFPYRKDPEDKNIRIA	0	0
A0R5R7	VSLDVWPGTAKRTVALAKDAG	0	0
A0R635	ARAKEAFGIQKRQRQMDRCRQ	0	0
A0R635	DDPESIFTTGKAAYIEDYAQR	0	0
A0R635	DSVKHTVSGPKGDLDVMIIAL	0	0
A0R635	FQIQRRCPHLKADLSKFGVVE	0	0
A0R635	GYNEYLYTFFKCLTDERIAYA	0	0
A0R635	HNFFETTDSVKHTVSGPKGDL	0	0
A0R635	NLENGRCLTTKGHELRCQKL_	0	0
A0R635	PMVYDMPARAKEAFGIQKRQR	0	0
A0R635	PVPDLRRELEKLGFHNFFETT	0	0
A0R635	RCPHLKADLSKFGVVEGNTLT	0	0
A0R635	TTKGHELRCQKL_________	0	0
A0R635	DPEHLRRYVNKDAVVLLPDYP	0	0
A0R635	QRMAPVLAAEKARWAPSAGEP	0	0
A0R635	RVVREPIPDEKFRYGFEIPAA	0	0
A0R635	VDYLYVSHLHKDHFDPEHLRR	0	0
A0R647	FDGADLPQLAKHFYAQALEER	0	0
A0R647	LGEQFMQWFLKEQVEEVAAMT	0	0
A0R652	AIDDQFGSFDKFQAQFTAAAN	0	0

A0R652	GDHAAIFLNEKNLAFHLGGHI	0	0
A0R652	GGHINHSIWWKNLSPNGGDKP	0	0
A0R652	HAFYLQYKNVKADYVKAFWNV	0	0
A0R652	MWEHAFYLQYKNVKADYVKAF	0	0
A0R652	QYKNVKADYVKAFWNVVNWDD	0	0
A0R652	YVKGVNDAIAKLEEARANGDH	0	0
A0R652	SGQINELHHSKHHATYVKGVN	0	0
A0R652	VQNRFAAATSKTSGLIFG___	0	0
A0R6E3	AEAANGIGVRKDLIEAVEAEE	0	0
A0R6E3	QRLYVARLRAKLTTAEAANGI	0	0
A0R6Q0	ATPVDHPIPGKVVVGIKTADV	0	0
A0R6Q0	EIEATGGAIIKGPYDDAHERR	0	0
A0R6Q0	GNGLVFYSPLKR_________	0	0
A0R6Q0	KFYETLGFSLKFRDGAHFAAL	0	0
A0R6Q0	LSTENLDESIKFYETLGFSLK	0	0
A0R6Q0	____MSDHEVKMVVLSTENLD	0	0
A0R6Q0	PIPGKVVVGIKTADVDAAAKE	0	0
A0R7F9	GRRRLAYEIAKHAEGIYAVID	0	0
A0R7F9	VLRTKVLRTDKH_________	0	0
A0R7F9	AEGIYAVIDVKAEPATVSELD	0	0
A0R7F9	LNLNESVLRTKVLRTDKH___	0	0
A0R7F9	SLETFLNVIRKDGGTVDKVDI	0	0
A0R7G6	AGVPIVGDDIKSQVGATITHR	0	0
A0R7G6	AIFASENNTIKIADVPPTDVI	0	0
A0R7G6	ASAYLMKSPPKQLADDVARAE	0	0
A0R7G6	FIASDPVWAKKFEDAGVPIVG	0	0
A0R7G6	GATITHRVMAKLFEDRGVTLD	0	0
A0R7G6	MLERSRLESKKVSKTQAVTSN	0	0
A0R7G6	NMLERSRLESKKVSKTQAVTS	0	0
A0R7G6	NTTVPGLMHVKFGPYHVRDVN	0	0
A0R7G6	PIEAASAYLMKSPPKQLADDV	0	0
A0R7G6	QRGPTLDGIGKYYADTIEVSD	0	0
A0R7G6	RSRLESKKVSKTQAVTSNLSG	0	0
A0R7G6	SDHVAWLDDRKWAYVRLEGRA	0	0
A0R7G6	SGALAGKVEDKNVHIGPSDHV	0	0
A0R7G6	TSNLSGALAGKVEDKNVHIGP	0	0
A0R7G6	VFIASDPVWAKKFEDAGVPIV	0	0
A0R7G6	VSDAEPVDVVKVLKEAEVDVL	0	0
A0R7G6	AEPVDVVKVLKEAEVDVLVSY	0	0
A0R7G6	FVAAFDVDAKKVGFDLSEAIF	0	0
A0R7G6	IDAVRAAKIAKDRGIGGPIEA	0	0
A4ZHR8	ADIVITATGNKDIITLDHMKA	0	0
A4ZHR8	ADLSLADFGRKEIRLAEHEMP	0	0
A4ZHR8	DMAALERSGAKKINIKPQVDQ	0	0
A4ZHR8	DSVTKSKFDNKYGTRHSLIDG	0	0
A4ZHR8	EPKGVSVFAWKGETLEEYWWA	0	0
A4ZHR8	ERSGAKKINIKPQVDQWIFDD	0	0
A4ZHR8	EYDNEVYRLAKHLDEKVARIH	0	0

A4ZHR8	GVLRERFEQDKTKWTKIAESV	0	0
A4ZHR8	IITLDHMKAMKNQAILGNIGH	0	0
A4ZHR8	INVNDSVTKSKFDNKYGTRHS	0	0
A4ZHR8	LRERFEQDKTKWTKIAESVKG	0	0
A4ZHR8	MAALERSGAKKINIKPQVDQW	0	0
A4ZHR8	MMVLRGAQYEKAGVVPPAEDD	0	0
A4ZHR8	______MTELKADVRNGIDYK	0	0
A4ZHR8	NKDIITLDHMKAMKNQAILGN	0	0
A4ZHR8	NRGTDVLIGGKKVLICGYGDV	0	0
A4ZHR8	PAEDDDPAEWKVFLGVLRERF	0	0
A4ZHR8	PAINVNDSVTKSKFDNKYGTR	0	0
A4ZHR8	QVDQWIFDDGKSIIVLSEGRL	0	0
A4ZHR8	RFEQDKTKWTKIAESVKGVTE	0	0
A4ZHR8	RGTDVLIGGKKVLICGYGDVG	0	0
A4ZHR8	RREYHDVQPLKGARISGSLHM	0	0
A4ZHR8	TKWTKIAESVKGVTEETTTGV	0	0
A4ZHR8	VGPNGTPEEPKGVSVFAWKGE	0	0
A4ZHR8	VLICGYGDVGKGCAESLAGQG	0	0
A4ZHR8	VYRLAKHLDEKVARIHVEALG	0	0
A4ZHR8	YIGVDVEGPYKPEHYRY____	0	0
A4ZHR8	HVEALGGTLTKLSKDQAEYIG	0	0
A4ZHR8	KADVRNGIDYKVADLSLADFG	0	0
A4ZHR8	QVIAQIELWTKNDEYDNEVYR	0	0
EG10025	AAAPAPAAGVKEVNVPDIGGD	0	0
EG10025	ADAAPAQAEEKKEAAPAAAPA	0	0
EG10025	APFAGVVKELKVNVGDKVKTG	0	0
EG10025	DAAPAQAEEKKEAAPAAAPAA	0	0
EG10025	DVQAYVKEAIKRAEAAPAATG	0	0
EG10025	EEAAKRKLDVKITPVVFIMKA	0	0
EG10025	EGAAPAAAPAKQEAAAPAPAA	0	0
EG10025	ELKVNVGDKVKTGSLIMIFEV	0	0
EG10025	ELSRELMTISKKARDGKLTAG	0	0
EG10025	EQSLITVEGDKASMEVPAPFA	0	0
EG10025	EQSLITVEGDKASMEVPSPQA	0	0
EG10025	EVPAPFAGTVKEIKVNVGDKV	0	0
EG10025	EVPAPFAGVVKELKVNVGDKV	0	0
EG10025	EVPSPQAGIVKEIKVSVGDKT	0	0
EG10025	FRKQQNEEAAKRKLDVKITPV	0	0
EG10025	GEAGAAAPAAKQEAAPAAAPA	0	0
EG10025	GGIPGMLPWPKVDFSKFGEIE	0	0
EG10025	IEEVELGRIQKISGANLSRNW	0	0
EG10025	ILREDVQAYVKEAIKRAEAAP	0	0
EG10025	KQQNEEAAKRKLDVKITPVVF	0	0
EG10025	KSAMEPVWNGKEFVPRLMLPI	0	0
EG10025	LAREFGVNLAKVKGTGRKGRI	0	0
EG10025	LSEDGQRLTLKKYINIGVAVD	0	0
EG10025	LSRELMTISKKARDGKLTAGE	0	0
EG10025	LVVPVFKDVNKKGIIELSREL	0	0

EG10025	_____MAIEIKVPDIGADEVE	0	0
EG10025	MLPWPKVDFSKFGEIEEVELG	0	0
EG10025	MTISKKARDGKLTAGEMQGGC	0	0
EG10025	NLAKVKGTGRKGRILREDVQA	0	0
EG10025	PAAAPAAAAAKDVNVPDIGSD	0	0
EG10025	QEAAAPAPAAKAEAPAAAPAA	0	0
EG10025	REFGVNLAKVKGTGRKGRILR	0	0
EG10025	SEDGQRLTLKKYINIGVAVDT	0	0
EG10025	TPNGLVVPVFKDVNKKGIIEL	0	0
EG10025	VKEIKVNVGDKVSTGSLIMVF	0	0
EG10025	VKEIKVSVGDKTQTGALIMIF	0	0
EG10025	VKELKVNVGDKVKTGSLIMIF	0	0
EG10025	VKITPVVFIMKAVAAALEQMP	0	0
EG10025	VMIPHVTHFDKTDITELEAFR	0	0
EG10025	VVPVFKDVNKKGIIELSRELM	0	0
EG10025	AAAPAAKAEGKSEFAENDAYV	0	0
EG10025	AEAPAAAPAAKAEGKSEFAEN	0	0
EG10025	APEVAILGVSKSAMEPVWNGK	0	0
EG10025	DEVEITEILVKVGDKVEAEQS	0	0
EG10025	DEVEVTEILVKVGDKVEAEQS	0	0
EG10025	DEVEVTEVMVKVGDKVAAEQS	0	0
EG10025	ITEILVKVGDKVEAEQSLITV	0	0
EG10025	SPQAGIVKEIKVSVGDKTQTG	0	0
EG10025	TDITELEAFRKQQNEEAAKRK	0	0
EG10025	VTEILVKVGDKVEAEQSLITV	0	0
EG10025	VTEVMVKVGDKVAAEQSLITV	0	0
EG10207	ATVGSCAQIGKNVHLSGGVGI	0	0
EG10207	DGQWVTHQWLKKAVLLSFRIN	0	0
EG10207	ESRYFDKVPMKFADYDEARFQ	0	0
EG10207	GQWVTHQWLKKAVLLSFRIND	0	0
EG10207	IEGAESRYFDKVPMKFADYDE	0	0
EG10207	LDSGALRVAEKIDGQWVTHQW	0	0
EG10207	SVVVSGNLPSKDGKYSLYCAV	0	0
EG10207	VSGNLPSKDGKYSLYCAVIVK	0	0
EG10207	CAVIVKKVDAKTRGKVGINEL	0	0
EG10207	KYSLYCAVIVKKVDAKTRGKV	0	0
EG10207	VKKVDAKTRGKVGINELLRTI	0	0
EG10207	YSLYCAVIVKKVDAKTRGKVG	0	0
EG10251	MMLPRSGLGHKHGIVLGNLVG	0	0
EG10251	_________MKKIDVKILDPR	0	0
EG10251	________MKKIDVKILDPRV	0	0
EG10251	____MKKIDVKILDPRVGKEF	0	0
EG10367	AGIALNDNFVKLVSWYDNETG	0	0
EG10367	AKKVVMTGPSKDNTPMFVKGA	0	0
EG10367	ARKHITAGAKKVVMTGPSKDN	0	0
EG10367	ATYEQIKAAVKAAAEGEMKGV	0	0
EG10367	AVKAAAEGEMKGVLGYTEDDV	0	0
EG10367	CTTNCLAPLAKVINDNFGIIE	0	0

EG10367	GEVCTSVFDAKAGIALNDNFV	0	0
EG10367	GLFLTDETARKHITAGAKKVV	0	0
EG10367	HGRFDGTVEVKDGHLIVNGKK	0	0
EG10367	IGRIVFRAAQKRSDIEIVAIN	0	0
EG10367	KDGHLIVNGKKIRVTAERDPA	0	0
EG10367	KVLDLIAHISK__________	0	0
EG10367	LDADYMAYMLKYDSTHGRFDG	0	0
EG10367	LEKAATYEQIKAAVKAAAEGE	0	0
EG10367	_______MTIKVGINGFGRIG	0	0
EG10367	NIIPSSTGAAKAVGKVLPELN	0	0
EG10367	PMFVKGANFDKYAGQDIVSNA	0	0
EG10367	PSKDNTPMFVKGANFDKYAGQ	0	0
EG10367	TARKHITAGAKKVVMTGPSKD	0	0
EG10367	TQKTVDGPSHKDWRGGRGASQ	0	0
EG10367	TTVHATTATQKTVDGPSHKDW	0	0
EG10367	VGKVLPELNGKLTGMAFRVPT	0	0
EG10367	WYDNETGYSNKVLDLIAHISK	0	0
EG10367	SSTGAAKAVGKVLPELNGKLT	0	0
EG10367	VKDGHLIVNGKKIRVTAERDP	0	0
EG10367	VTAERDPANLKWDEVGVDVVA	0	0
EG10407	DFAEFDADAAKKHLRPVARQP	0	0
EG10407	DLVKLLGERCKTLKEMAQSCR	0	0
EG10407	DQMLEEGTAYKCYCSKERLEA	0	0
EG10407	EGTAYKCYCSKERLEALREEQ	0	0
EG10407	EWDEGPYYQTKRFDRYNAVID	0	0
EG10407	FAEFDADAAKKHLRPVARQPL	0	0
EG10407	HVSMINGDDGKKLSKRHGAVS	0	0
EG10407	INGDDGKKLSKRHGAVSVMQY	0	0
EG10407	KLLGERCKTLKEMAQSCRYFY	0	0
EG10407	LEALREEQMAKGEKPRYDGRC	0	0
EG10407	LREEQMAKGEKPRYDGRCRHS	0	0
EG10407	NNTPRQINILKALKAPVPVYA	0	0
EG10407	PRQINILKALKAPVPVYAHVS	0	0
EG10407	RNGPQLADLVKLLGERCKTLK	0	0
EG10407	SKSASAFNTDKLLWLNHHYIN	0	0
EG10407	TADELEVGMGKVGMPLRVAVT	0	0
EG10407	VSMINGDDGKKLSKRHGAVSV	0	0
EG10407	ALDVTVHAIGKTRSIERINKA	0	0
EG10407	ARQPLEVVRDKLAAITDWTAE	0	0
EG10407	GKTRSIERINKALDFIAEREN	0	0
EG10407	IKYFTLNAVSKSASAFNTDKL	0	0
EG10407	_________MKIKTRFAPSPT	0	0
EG10407	_______MKIKTRFAPSPTGY	0	0
EG10410	ADYEERKAKIKADAEEAARKI	0	0
EG10410	AEAQPDREIEKRGPAIAQAFD	0	0
EG10410	AEFFFNTDRKKRLEDNLPRLQ	0	0
EG10410	AIAQAFDAEGKPSKAAEGWAR	0	0
EG10410	ALVYTMKGDQKYFPVYANDGK	0	0

EG10410	ANKRVSNILAKSDEVLSDRVN	0	0
EG10410	ASTLKEPEEIKLAMQVVVLRD	0	0
EG10410	ATSLAKLPIPKLMRWGASDVH	0	0
EG10410	DAAAALAAANKRVSNILAKSD	0	0
EG10410	DAEFFFNTDRKKRLEDNLPRL	0	0
EG10410	FDKVMVMVDDKELRINRLTML	0	0
EG10410	FQQQLGTLRDKTDRIQALAGW	0	0
EG10410	GEWLLYRAHVKGESTEALLPN	0	0
EG10410	GKVIADYEERKAKIKADAEEA	0	0
EG10410	IKADAEEAARKIGGNADLSES	0	0
EG10410	LPNMVATSLAKLPIPKLMRWG	0	0
EG10410	LREPVDAFFDKVMVMVDDKEL	0	0
EG10410	LSDRVNASTLKEPEEIKLAMQ	0	0
EG10410	LVEWPVVLTAKFEEKFLAVPA	0	0
EG10410	NFIFVANIESKDPQQIISGNE	0	0
EG10410	NHATRAGLLSKCDLMTNMVFE	0	0
EG10410	PVACALAIADKMDTLAGIFGI	0	0
EG10410	PVVLTAKFEEKFLAVPAEALV	0	0
EG10410	QAFDAEGKPSKAAEGWARGCG	0	0
EG10410	QYPEILRERGKVIADYEERKA	0	0
EG10410	TEEAVRLYGDKLTNANVVDDV	0	0
EG10410	TRPADFDARMKAVSHFRTLDA	0	0
EG10410	VDQAERLTTDKGEWLLYRAHV	0	0
EG10410	VHTVTLLLGDKVIPATILGIQ	0	0
EG10410	VIADYEERKAKIKADAEEAAR	0	0
EG10410	VPAEALVYTMKGDQKYFPVYA	0	0
EG10410	WFAAPRRLALKVANLAEAQPD	0	0
EG10410	AGIFGIGQHPKGDKDPFALRR	0	0
EG10410	ALGVLRIIVEKNLNLDLQTLT	0	0
EG10410	DPQQIISGNEKVVRPRLADAE	0	0
EG10410	FGIGQHPKGDKDPFALRRAAL	0	0
EG10410	KYFPVYANDGKLLPNFIFVAN	0	0
EG10410	LRINRLTMLEKLRELFLRVAD	0	0
EG10410	_______MSEKTFLVEIGTEE	0	0
EG10410	VEIGTEELPPKALRSLAESFA	0	0
EG10416	AMVEGIELTLKSMLDVVRKFG	0	0
EG10416	APGNVLGIMQKGYTLNGRTIR	0	0
EG10416	_______MSSKEQKTPEGQAP	0	0
EG10416	____MSSKEQKTPEGQAPEEI	0	0
EG10416	RAAMVTVAKAKA_________	0	0
EG10416	SLDRALEVADKANPDMSAMVE	0	0
EG10416	TIRAAMVTVAKAKA_______	0	0
EG10416	TLKSMLDVVRKFGVEVIAETN	0	0
EG10416	IEKAHKFALEKFINELLPVID	0	0
EG10416	LRRRTELDIEKAHKFALEKFI	0	0
EG10416	RTELDIEKAHKFALEKFINEL	0	0
EG10489	AVEKAYKGERKISWMEIYTGE	0	0
EG10489	DLIREYRVAIKGPLTTPVGGG	0	0

EG10489	ERLMDGAKLLKCSEFGDAIIE	0	0
EG10489	GELIDGGPWLKVKNPNTGKEI	0	0
EG10489	GIKPCSEEGTKRLVRAAIEYA	0	0
EG10489	GTAPKYAGQDKVNPGSIILSA	0	0
EG10489	GWTEAADLIVKGMEGAINAKT	0	0
EG10489	IGVDVTPAMLKVVDAAVEKAY	0	0
EG10489	IKFLREEMGVKKIRFPEHCGI	0	0
EG10489	ISWMEIYTGEKSTQVYGQDVW	0	0
EG10489	KFLREEMGVKKIRFPEHCGIG	0	0
EG10489	LFEATHGTAPKYAGQDKVNPG	0	0
EG10489	LIDGGPWLKVKNPNTGKEIVI	0	0
EG10489	MLKVVDAAVEKAYKGERKISW	0	0
EG10489	NDRDSVTLVHKGNIMKFTEGA	0	0
EG10489	NIMKFTEGAFKDWGYQLAREE	0	0
EG10489	QGKKITLQNGKLNVPENPIIP	0	0
EG10489	RFPEHCGIGIKPCSEEGTKRL	0	0
EG10489	RYYQGTPSPVKHPELTDMVIF	0	0
EG10489	VKGMEGAINAKTVTYDFERLM	0	0
EG10489	VTLVHKGNIMKFTEGAFKDWG	0	0
EG10489	VVDAAVEKAYKGERKISWMEI	0	0
EG10489	YDFERLMDGAKLLKCSEFGDA	0	0
EG10489	ADSADAEKVIKFLREEMGVKK	0	0
EG10489	ESKVVVPAQGKKITLQNGKLN	0	0
EG10489	EWKADSADAEKVIKFLREEMG	0	0
EG10489	NPNTGKEIVIKDVIADAFLQQ	0	0
EG10489	SEDIYAGIEWKADSADAEKVI	0	0
EG10489	SKVVVPAQGKKITLQNGKLNV	0	0
EG10492	AFQAVDQDALKAKFAVSNVNG	0	0
EG10492	AQQSEVLKGLKVALSKAEGEK	0	0
EG10492	CAKAAQEDILKAYEAYDFHEV	0	0
EG10492	CVSNVAGDGEKRKFA______	0	0
EG10492	DDLYGIIRAAKKGKKTFILHD	0	0
EG10492	DIIKDRQYTAKADSVARRSCQ	0	0
EG10492	DLYGIIRAAKKGKKTFILHDG	0	0
EG10492	DQKGLRAQSLKEIKGVQWIPD	0	0
EG10492	EKFTAAEFRAKCREYAATQVD	0	0
EG10492	ELLKVRGEVNKVIEQARADKK	0	0
EG10492	EMGSFYLDIIKDRQYTAKADS	0	0
EG10492	GIIRAAKKGKKTFILHDGPPY	0	0
EG10492	GIQAWWDLDAKEILGDEADQY	0	0
EG10492	GKIIGNGHLHKGAKPVHWCVD	0	0
EG10492	GLRAQSLKEIKGVQWIPDWGQ	0	0
EG10492	GSIHIGHSVNKILKDIIVKSK	0	0
EG10492	HGFTVDGQGRKMSKSIGNTVS	0	0
EG10492	HGPDDYVIGQKYGLETANPVG	0	0
EG10492	HIGHSVNKILKDIIVKSKGLS	0	0
EG10492	IELKVEQEYGKPGEKFTAAEF	0	0
EG10492	IGNGHLHKGAKPVHWCVDCRS	0	0

EG10492	ILGDEADQYVKVPDTLDVWFD	0	0
EG10492	KILKDIIVKSKGLSGYDSPYV	0	0
EG10492	KVALSKAEGEKCPRCWHYTQD	0	0
EG10492	KVIEQARADKKVGGSLEAAVT	0	0
EG10492	LAEAEVEYYDKTSPSIDVAFQ	0	0
EG10492	LANLNGFDPAKDMVKPEEMVV	0	0
EG10492	______MSDYKSTLNLPETGF	0	0
EG10492	MSSLMISTAMKGKAPYRQVLT	0	0
EG10492	NGFDPAKDMVKPEEMVVLDRW	0	0
EG10492	NKVIEQARADKKVGGSLEAAV	0	0
EG10492	NTVSPQDVMNKLGADILRLWV	0	0
EG10492	PADAQQSEVLKGLKVALSKAE	0	0
EG10492	QAVDQDALKAKFAVSNVNGPI	0	0
EG10492	QEKGALLHVEKMQHSYPCCWR	0	0
EG10492	QHSYPCCWRHKTPIIFRATPQ	0	0
EG10492	RCWHYTQDVGKVAEHAEICGR	0	0
EG10492	RTLELMEEVAKRVEVDGIQAW	0	0
EG10492	SLMISTAMKGKAPYRQVLTHG	0	0
EG10492	SNVAGDGEKRKFA________	0	0
EG10492	TEANIIRALGKIIGNGHLHKG	0	0
EG10492	TGFPMRGDLAKREPGMLARWT	0	0
EG10492	TPQWFVSMDQKGLRAQSLKEI	0	0
EG10492	TVDGQGRKMSKSIGNTVSPQD	0	0
EG10492	VEQEYGKPGEKFTAAEFRAKC	0	0
EG10492	VLDRWAVGCAKAAQEDILKAY	0	0
EG10492	VLKGLKVALSKAEGEKCPRCW	0	0
EG10492	VNKILKDIIVKSKGLSGYDSP	0	0
EG10492	VTDYTILGTVKGAELELLRFT	0	0
EG10492	VWGYLPGEREKYVFTGEWYEG	0	0
EG10492	WDCHGLPIELKVEQEYGKPGE	0	0
EG10492	WGVPMSLFVHKDTEELHPRTL	0	0
EG10492	WSHPYLTMDFKTEANIIRALG	0	0
EG10492	YGIIRAAKKGKKTFILHDGPP	0	0
EG10492	YPTLDGVNVFKANDIVVALLQ	0	0
EG10492	GEMAVSDEILKRAADSYRRIR	0	0
EG10492	NDIVVALLQEKGALLHVEKMQ	0	0
EG10492	QIDGQAVILAKDLVESVMQRI	0	0
EG10492	TLYAEPELSAKLTALGDELRF	0	0
EG10492	YAATQVDGQRKDFIRLGVLGD	0	0
EG10762	AKLYELEMQKKNAEKQAMEDN	0	0
EG10762	AVAELDALEEKLAQLEFRRMF	0	0
EG10762	CQNDRSQHKNKDQAMKQMKAK	0	0
EG10762	EESEGEVAGIKSVTIKISGDY	0	0
EG10762	ELEMQKKNAEKQAMEDNKSDI	0	0
EG10762	EVAGIKSVTIKISGDYAYGWL	0	0
EG10762	KAKLYELEMQKKNAEKQAMED	0	0
EG10762	KDQAMKQMKAKLYELEMQKKN	0	0
EG10762	KNKDQAMKQMKAKLYELEMQK	0	0

EG10762	LRGYLDYDAKKERLEEVNAEL	0	0
EG10762	NAEKQAMEDNKSDIGWGSQIR	0	0
EG10762	RSYVLDDSRIKDLRTGVETRN	0	0
EG10762	SLDQFIEASLKAGL_______	0	0
EG10762	SQHKNKDQAMKQMKAKLYELE	0	0
EG10762	TETGVHRLVRKSPFDSGGRRH	0	0
EG10762	TQCQNDRSQHKNKDQAMKQMK	0	0
EG10762	VLRGYLDYDAKKERLEEVNAE	0	0
EG10762	EAVVDTLDQMKQGLEDVSGLL	0	0
EG10762	NEPERAQALGKERSSLEAVVD	0	0
EG10770	DFAAGANIDGKHYFGINWDRD	0	0
EG10770	DHELNEVKAEKLPQVASPLTF	0	0
EG10770	DLIRNELSSYKQLPLNFYQIQ	0	0
EG10770	GVMRSREFLMKDAYSFHTSQE	0	0
EG10770	IEYKYRRNGEKQLIKTGDIVE	0	0
EG10770	LPLNFYQIQTKFRDEVRPRFG	0	0
EG10770	NIELAEAIAPKEPRAAATQEM	0	0
EG10770	QGIEVLLDDRKERPGVMFADM	0	0
EG10770	QVAILPMNMHKSFRVQELAEK	0	0
EG10770	RNLDNDDIEYKYRRNGEKQLI	0	0
EG10770	RTSQYLLSTLKETPADAEVIS	0	0
EG10770	TWLPTGVRVLKKVENIVREEM	0	0
EG10770	VRGDHELNEVKAEKLPQVASP	0	0
EG10770	WLPTGVRVLKKVENIVREEMN	0	0
EG10770	YRRNGEKQLIKTGDIVEYLVK	0	0
EG10770	ATEEEIRAVVKAGPGSLGPVN	0	0
EG10770	EMTLVDTPNAKTIAELVEQFN	0	0
EG10770	EVGHIFQLGTKYSEALKASVQ	0	0
EG10770	IEKTVKTLLVKAVEGSSFPQV	0	0
EG10770	KTGDIVEYLVKQIKG______	0	0
EG10770	LVEQFNLPIEKTVKTLLVKAV	0	0
EG10770	QFNLPIEKTVKTLLVKAVEGS	0	0
EG10770	QLMLRAGMIRKLASGLYTWLP	0	0
EG10770	SPDGQGRLLIKRGIEVGHIFQ	0	0
EG10770	TYDAMYAAYSKIFSRMGLDFR	0	0
EG10803	AEKIPSINVSKHRLDVQFDNV	0	0
EG10803	ALTEKDKADIKTAALIGVDYL	0	0
EG10803	AMARVCLGAEKIPSINVSKHR	0	0
EG10803	ASEAVNLLRDKGYLMSGDLVI	0	0
EG10803	DAGCDAKIVAKVERAEAVCSQ	0	0
EG10803	DANLGKGEGDKEKVGIDYKGL	0	0
EG10803	GDKEKVGIDYKGLPADVVPGD	0	0
EG10803	GPLSNNKGINKLGGGLSAEAL	0	0
EG10803	IGDPELVGIQKALIRRARQLN	0	0
EG10803	KIRVSTFKEGKVFLNIGDKFL	0	0
EG10803	LLLDDGRVQLKVLEVQGMKVF	0	0
EG10803	LSAEALTEKDKADIKTAALIG	0	0
EG10803	MSAMYAANHLKGVTAIITMTE	0	0

EG10803	NLGKGEGDKEKVGIDYKGLPA	0	0
EG10803	PATDRDNNLEKVIAAGANVVR	0	0
EG10803	QGPKIRVSTFKEGKVFLNIGD	0	0
EG10803	QLKVLEVQGMKVFTEVTVGGP	0	0
EG10803	RLARDAGCDAKIVAKVERAEA	0	0
EG10803	VAILGDLQGPKIRVSTFKEGK	0	0
EG10803	VTVGGPLSNNKGINKLGGGLS	0	0
EG10803	DKFLLDANLGKGEGDKEKVGI	0	0
EG10803	GGLSAEALTEKDKADIKTAAL	0	0
EG10803	__MSRRLRRTKIVTTLGPATD	0	0
EG10803	NFSHGSPEDHKMRADKVREIA	0	0
EG10803	SPEDHKMRADKVREIAAKLGR	0	0
EG10876	EGGVVDLNTLKAANIIGIQIE	0	0
EG10876	GLGKTGGRGHKGQKSRSGGGV	0	0
EG10876	GQMPLYRRLPKFGFTSRKAAI	0	0
EG10876	KTGGRGHKGQKSRSGGGVRRG	0	0
EG10876	LGRGIGSGLGKTGGRGHKGQK	0	0
EG10876	LNTLSPAEGSKKAGKRLGRGI	0	0
EG10876	NIIGIQIEFAKVILAGEVTTP	0	0
EG10876	NTLSPAEGSKKAGKRLGRGIG	0	0
EG10876	PVTVRGLRVTKGARAAIEAAG	0	0
EG10876	RLPKFGFTSRKAAITAEIRLS	0	0
EG10876	SPAEGSKKAGKRLGRGIGSGL	0	0
EG10876	TAEIRLSDLAKVEGGVVDLNT	0	0
EG10903	AGKMEGTFKRKPERSDLSADI	0	0
EG10903	DYGVQLREKQKVRRIYGVLER	0	0
EG10903	EQAPGQHGARKPRLSDYGVQL	0	0
EG10903	KPTWLEVDAGKMEGTFKRKPE	0	0
EG10903	LKSGVRAIDTKCKIEQAPGQH	0	0
EG10903	LSDYGVQLREKQKVRRIYGVL	0	0
EG10903	___MARYLGPKLKLSRREGTD	0	0
EG10903	_MARYLGPKLKLSRREGTDLF	0	0
EG10903	RAEARQLVSHKAIMVNGRVVN	0	0
EG10903	RNYYKEAARLKGNTGENLLAL	0	0
EG10903	SGVRAIDTKCKIEQAPGQHGA	0	0
EG10903	SRREGTDLFLKSGVRAIDTKC	0	0
EG10903	VDAGKMEGTFKRKPERSDLSA	0	0
EG10903	VLERQFRNYYKEAARLKGNTG	0	0
EG10903	DVVSIREKAKKQSRVKAALEL	0	0
EG10903	NDVVSIREKAKKQSRVKAALE	0	0
EG10903	NEHLIVELYSK__________	0	0
EG10903	SPNDVVSIREKAKKQSRVKAA	0	0
EG10905	AVIRSMVMRTKHAVTEASPMV	0	0
EG10905	GRRQLAYPINKLHKAHYVLMN	0	0
EG10905	HAVTEASPMVKAKDERRERRD	0	0
EG10905	QLAYPINKLHKAHYVLMNVEA	0	0
EG10905	VTEASPMVKAKDERRERRDDF	0	0
EG10954	AASFGSFADFKAQFTDAAIKN	0	0

EG10954	FKAQFTDAAIKNFGSGWTWLV	0	0
EG10954	NFGSGWTWLVKNSDGKLAIVS	0	0
EG10954	NLIKGTAFEGKSLEEIIRSSE	0	0
EG10954	SAETIEYHYGKHHQTYVTNLN	0	0
EG10954	SFELPALPYAKDALAPHISAE	0	0
EG10954	WALVNWEFVAKNLAA______	0	0
EG10954	APNAGGEPTGKVAEAIAASFG	0	0
EG10954	WTWLVKNSDGKLAIVSTSNAG	0	0
EG11192	AEVTNSAIELKVLIEQMREQI	0	0
EG11192	AHVKETYNILKKKEAVGRRLD	0	0
EG11192	EELDRLEAHVKETYNILKKKE	0	0
EG11192	ERIRSRLTRGKVECTLRYEPD	0	0
EG11192	FNRESNTLASKSINAEVTNSA	0	0
EG11192	HVKETYNILKKKEAVGRRLDF	0	0
EG11192	KQLVTAANWVKMQSDEGEINP	0	0
EG11192	RLEGVTAEVVKVRSHMPEILQ	0	0
EG11192	SMTAYARREIKGEWGSATWEM	0	0
EG11192	VARETEGQALKALIEQRLEGV	0	0
EG11192	VKETYNILKKKEAVGRRLDFM	0	0
EG11192	LQWQRERLVAKLEDAQVQLEN	0	0
EG11192	SAQGELILNEKLAKQLVTAAN	0	0
EG11223	AALVLDGIQVKRPRCQEAAQQ	0	0
EG11223	ATELADYLVAKGVPFREAHHI	0	0
EG11223	EAVVEAIRQGKPLEDLPLSEL	0	0
EG11223	GALTGMMMTLKGLPLAYNKDM	0	0
EG11223	KLIDKVGQLGKKLHTGRSRND	0	0
EG11223	LAYNKDMQEDKEGLFDALDTW	0	0
EG11223	LIDKVGQLGKKLHTGRSRNDQ	0	0
EG11223	LQSCLDKRAAKGGVSPQQVAQ	0	0
EG11223	NPDALELIRGKCGRVQGALTG	0	0
EG11223	PILSLQSCLDKRAAKGGVSPQ	0	0
EG11223	QDIVGSVAWSKALVTVGVLTA	0	0
EG11223	QVATDLKLWCKDTVSELLTAN	0	0
EG11223	RDESRLQDALKRLDVSPLGCG	0	0
EG11223	RFTQAADQRFKQFNDSLRFDY	0	0
EG11223	SRNDQVATDLKLWCKDTVSEL	0	0
EG11223	TLKGLPLAYNKDMQEDKEGLF	0	0
EG11223	TSGSSLMPQKKNPDALELIRG	0	0
EG11223	VTSGSSLMPQKKNPDALELIR	0	0
EG11223	AEDIHSWVEGKLIDKVGQLGK	0	0
EG11223	LEDLPLSELQKFSQVIDEDVY	0	0
EG11415	ILTAASRDLDKFLWFIESNIE	0	0
EG11415	IQFIDLSLITKQAHWNMRGAN	0	0
EG11415	LDIHNVQDHLKELADRYAIVA	0	0
EG11415	______MSTAKLVKSKATNLL	0	0
EG11415	___MSTAKLVKSKATNLLYTR	0	0
EG11415	_MSTAKLVKSKATNLLYTRND	0	0
EG11415	NDVRKAIGEAKDDDTADILTA	0	0

EG11415	RYAIVANDVRKAIGEAKDDDT	0	0
EG11415	ALGTTQVINSKTPLKSYPLDI	0	0
EG11415	TRNDVSDSEKKATVELLNRQV	0	0
EG11415	YTRNDVSDSEKKATVELLNRQ	0	0
EG11427	ALSMDAVQKAKSGHPGAPMGM	0	0
EG11427	ANAVGMAIAEKTLAAQFNRPG	0	0
EG11427	ANIARGGYVLKDCAGQPELIF	0	0
EG11427	AVEEARAVTDKPSLLMCKTII	0	0
EG11427	CDQVESAVAWKYGVERQDGPT	0	0
EG11427	CSLAGTLKLGKLIAFYDDNGI	0	0
EG11427	DIDGHDAASIKRAVEEARAVT	0	0
EG11427	ELKNFRQLHSKTPGHPEVGYT	0	0
EG11427	EMPSDFDAKAKEFIAKLQANP	0	0
EG11427	FGFTVDNVVAKAKELL_____	0	0
EG11427	FIAKLQANPAKIASRKASQNA	0	0
EG11427	FTVDNVVAKAKELL_______	0	0
EG11427	HEVCSLAGTLKLGKLIAFYDD	0	0
EG11427	IAEVLWRDFLKHNPQNPSWAD	0	0
EG11427	IALTREQLGWKYAPFEIPSEI	0	0
EG11427	IRALSMDAVQKAKSGHPGAPM	0	0
EG11427	KGEMPSDFDAKAKEFIAKLQA	0	0
EG11427	KTIIGFGSPNKAGTHDSHGAP	0	0
EG11427	______MSSRKELANAIRALS	0	0
EG11427	PSEIYAQWDAKEAGQAKESAW	0	0
EG11427	QEAAEFTRRMKGEMPSDFDAK	0	0
EG11427	RNAVRMAALMKQRQVMVYTHD	0	0
EG11427	TGYDLPMEELKNFRQLHSKTP	0	0
EG11427	VEAGIADYWYKYVGLNGAIVG	0	0
EG11427	VTDKPSLLMCKTIIGFGSPNK	0	0
EG11427	AYEKLTAEGVKARVVSMPSTD	0	0
EG11427	DAAYRESVLPKAVTARVAVEA	0	0
EG11427	EVELAVAAYEKLTAEGVKARV	0	0
EG11427	GQAKESAWNEKFAAYAKAYPQ	0	0
EG11427	PSNLTLWSGSKAINEDAAGNY	0	0
EG11427	QANPAKIASRKASQNAIEAFG	0	0
EG11427	QWDAKEAGQAKESAWNEKFAA	0	0
EG11427	VSMPSTDAFDKQDAAYRESVL	0	0
EG11518	EFYLVTVPDGKLSPRLAALKP	0	0
EG11518	GKLSPRLAALKPGDEVQVVSE	0	0
EG11518	GPYLSILQLGKDLDRFKNLVL	0	0
EG11518	LESTIGLPMNKETSHVMLCGN	0	0
EG11518	___MADWVTGKVTKVQNWTDA	0	0
EG11518	MADWVTGKVTKVQNWTDALFS	0	0
EG11518	MQELEKRYEGKLRIQTVVSRE	0	0
EG11518	QLLKETRQMTKHLRRRPGHMT	0	0
EG11518	QMVRDTQQLLKETRQMTKHLR	0	0
EG11518	SYLPLMQELEKRYEGKLRIQT	0	0
EG11518	VLPFTAGQFTKLGLEIDGERV	0	0

EG11615	HIHNLRDKVGKARIRTVRGFG	0	0
EG11615	ILTARDTLTDKIAGLDVGADD	0	0
EG11615	LDVGADDYLVKPFALEELHAR	0	0
EG11615	LEVHIHNLRDKVGKARIRTVR	0	0
EG11615	MGGEELILTPKEYALLSRLML	0	0
EG11615	GLHFLARIRQKKYTLPVLILT	0	0
EG11615	_________MKILIVEDDTLL	0	0
EG12204	FGFITPADGSKDVFVHFSAIQ	0	0
EG12204	HFSAIQGNGFKTLAEGQNVEF	0	0
EG12204	KGQVKWFNESKGFGFITPADG	0	0
EG12204	________MAKIKGQVKWFNE	0	0
EG12204	______MAKIKGQVKWFNESK	0	0
EG12204	NVEFEIQDGQKGPAAVNVTAI	0	0
EG12411	GEKALQEYCAKHLIFRTSWVY	0	0
EG12411	IFRTSWVYAGKGNNFAKTMLR	0	0
EG12411	LVLPDWQVGVKRMLNELFTTT	0	0
EG12411	___MNILLFGKTGQVGWELQR	0	0
EG12411	NFAKTMLRLAKEREELAVIND	0	0
EG12411	RPHNSRLNTEKFQQNFALVLP	0	0
EG12411	TAHAIRVALNKPDVAGLYHLV	0	0
EG12411	TAPLNVYGETKLAGEKALQEY	0	0
EG12411	VNAAAHTAVDKAESEPEFAQL	0	0
EG12411	VYAGKGNNFAKTMLRLAKERE	0	0
EG12411	VYGETKLAGEKALQEYCAKHL	0	0
EG12411	INATSVEAIAKAANEVGAWVI	0	0
EG12411	YAALVFEEARKAGIPLALNKL	0	0
EG12503	EALTLMPVGSKWELTIPQELA	0	0
EG12503	TDRVRVHYTGKLIDGTVFDSS	0	0
EG12503	VAGIADALEGKHPAVPVDVVH	0	0
EG12503	GVKYLEENAKKEGVNSTESGL	0	0
EG12503	RFQAMAAEGVKYLEENAKKEG	0	0
EG12545	AATLSRSQDRKVKISEIIKHT	0	0
EG12545	AGSLRIGGQTKHFLVHETQEE	0	0
EG12545	AKNKKHVFCEKPIALSYEDCV	0	0
EG12545	ARFMNMHDNAKITCVYDPENG	0	0
EG12545	CHTKRNGWENKQERLSWKKMK	0	0
EG12545	DGAIAYGHPGKKTPLWLASLI	0	0
EG12545	DRKVKISEIIKHTSVM_____	0	0
EG12545	EDCVDMVKACKEAGVTFMAGH	0	0
EG12545	ENKQERLSWKKMKEQSGGHLY	0	0
EG12545	EPVIKAAKNKKHVFCEKPIAL	0	0
EG12545	ETQEEDDDRRKGNMTSEMDGA	0	0
EG12545	GAIAYGHPGKKTPLWLASLIR	0	0
EG12545	KEPVIKAAKNKKHVFCEKPIA	0	0
EG12545	KQERLSWKKMKEQSGGHLYHH	0	0
EG12545	LFLHNILCGAKPEEDYIDLLN	0	0
EG12545	LFMTLEFPSGKLATLEWGSAF	0	0
EG12545	LHKEPVIKAAKNKKHVFCEKP	0	0

EG12545	LSYEDCVDMVKACKEAGVTFM	0	0
EG12545	MSSLDALVSSKLVDCVIVATP	0	0
EG12545	NFFNGVQYARKLIKEGVIGEI	0	0
EG12545	NGVQYARKLIKEGVIGEILSC	0	0
EG12545	PEHYVIINGTKGSIKIDMQET	0	0
EG12545	TLSRSQDRKVKISEIIKHTSV	0	0
EG12545	TPLWLASLIRKETLFLHNILC	0	0
EG12545	VIGEILSCHTKRNGWENKQER	0	0
EG12545	VIINGTKGSIKIDMQETAGSL	0	0
EG12545	WENKQERLSWKKMKEQSGGHL	0	0
EG12545	VIVATPNYLHKEPVIKAAKNK	0	0
EG12669	ETADGEYQAKKAIVCAGTWVK	0	0
EG12669	IETADGEYQAKKAIVCAGTWV	0	0
EG12669	KAIVCAGTWVKDLLPELPVQP	0	0
EG12669	LAIKTWIQLAKEAGCAQLFNC	0	0
EG12669	LIRHAYGEGEKYVPLVLRAQT	0	0
EG12669	LPELPVQPVRKVFAWYQADGR	0	0
EG12669	__________MKYDLIIIGSGS	0	0
EG12669	PAENDALKIGKHNGGQVIHSA	0	0
EG12669	QADGRYSVKNKFPAFTGELPN	0	0
EG12669	SAEQWQLNVEKLDAQGIMARW	0	0
EG12669	SGFLRSELAIKTWIQLAKEAG	0	0
EG12669	WYQADGRYSVKNKFPAFTGEL	0	0
EG12669	YGFPAENDALKIGKHNGGQVI	0	0
EG12669	EIAADFAQDKKSDFDLTPFRL	0	0
EG12669	LITGLSGHGFKFASVLGEIAA	0	0
EG12712	PDEQRVADAWKAAMEDVLKVQ	0	0
EG12712	RMEAPAVRVAKTMNTPHGDAI	0	0
EG12712	VFDLRFCVPNKEVMPERGIHT	0	0
EG12712	AWKAAMEDVLKVQDQNQIPEL	0	0
EG12712	SNEELALPKEKLQELHI____	0	0
EG12982	DPIFFVPSEGKTAAELTREEK	0	0
EG12982	EDATDQKNLQKLLETMKDVPD	0	0
EG12982	KNLQKLLETMKDVPDDQRQAR	0	0
EG12982	KTAAELTREEKSAISHRGQAL	0	0
EG12982	RYSGEDATDQKNLQKLLETMK	0	0
EG12982	SAISHRGQALKLLLDALRNG_	0	0
EG12982	GLTFIENAILKARHAAKVTAL	0	0
EG12982	KVVLATGNVGKVRELASLLSD	0	0
EG12982	NAILKARHAAKVTALPAIADD	0	0
EG13014	AIVLPALWNEKRDTKRAKLLQ	0	0
EG13014	AVISRKTTGDKQAFHSAHVQP	0	0
EG13014	DGSSIPALLKKLEEHGMTQLG	0	0
EG13014	DPWHILQTGGKEIKSAIPMGC	0	0
EG13014	EQYVTKPVDAKIQDRFAEGIL	0	0
EG13014	ESNAGAVISRKTTGDKQAFHS	0	0
EG13014	FVHTVEQYVTKPVDAKIQDRF	0	0
EG13014	GVLDQVLDALKGMDVLEFGGI	0	0

EG13014	HILQTGGKEIKSAIPMGCVLT	0	0
EG13014	ILLTLIEDGPKALKEPENYDV	0	0
EG13014	LDGSSIPALLKKLEEHGMTQL	0	0
EG13014	LHTPTRILFGKGAIAGLREQI	0	0
EG13014	PALWNEKRDTKRAKLLQYAER	0	0
EG13014	TLIEDGPKALKEPENYDVRAN	0	0
EG13014	VGGGSVLDGTKFIAAAANYPE	0	0
EG13014	WNEKRDTKRAKLLQYAERVWN	0	0
EG13014	MNAVKLVREQKVTFLLAVGGG	0	0
EG13014	PAYETLMNAVKLVREQKVTFL	0	0
EG13014	VLITYGGGSVKKTGVLDQVLD	0	0
EG13952	DEKSALAYLEKHTARLKNLGV	0	0
EG13952	FLWKVWTESEKNHEAGGIYLF	0	0
EG13952	KNLGVEEVVAKVFDVNEPLSQ	0	0
EG13952	PFGDAMAEQLKPLAESINQEP	0	0
EG13952	SINQEPGFLWKVWTESEKNHE	0	0
EG13952	NEPLSQINQAKLA________	0	0
O05598	ADVEDLLDAGKPFGLRPSAIL	0	0
O05598	APNFAYALLAKRLRRQAKPGD	0	0
O05598	EGHVVVCGRVKDVIIMAGRNI	0	0
O05598	IPMFFGAELVKVTPMDFLRDT	0	0
O05598	LLAKRLRRQAKPGDFDLSTLR	0	0
O05598	MFVGAQYDVDKDVMVSWLPCF	0	0
O05598	____MSRFTEKMFHNARTATT	0	0
O05598	QLTSGSTGSPKAVQITHRNIY	0	0
O05598	TMTVIGMIEAKAVIVSEPFLV	0	0
O05598	AALRRAVPATKGNTRRLATLG	0	0
O05598	DFLRDTLLWAKLIDKYQGTMT	0	0
O05598	DTLLWAKLIDKYQGTMTAAPN	0	0
O05598	FLVAIPILEQKGMQVLTVADL	0	0
O05598	PGTIPKTPSGKLRRANSVTLV	0	0
O05814	AADTLKNVLIKVRQPGGDTEL	0	0
O05814	DDFAKHPFLVKGYIGPKALRE	0	0
O05814	DPADAEVASHKLLIRAGYIRP	0	0
O05814	DRQASPGVKFKDAELLGMPWI	0	0
O05814	EIGHIFQLGSKYTDAFTADVL	0	0
O05814	FPLTLYQIQTKYRDEARPRAG	0	0
O05814	FTADVLGEDGKPVRLTMGSYG	0	0
O05814	GILRAREFVMKDSYSFDIDAA	0	0
O05814	GRTVTAADTLKNVLIKVRQPG	0	0
O05814	GVPGDREVDDKRLGAALEPAD	0	0
O05814	LDDRQASPGVKFKDAELLGMP	0	0
O05814	LTVKGEYSSYKDFPLTLYQIQ	0	0
O05814	PFLVKGYIGPKALRENNVRYL	0	0
O05814	THEELFTLTVKGEYSSYKDFP	0	0
O05814	TQYGDSVFRLKDRRGNDYLLG	0	0
O05814	YALLDDDDFAKHPFLVKGYIG	0	0
O05814	PFDVHLVIANKDAQARAGATA	0	0

O06188	AHCPVAVMRSKVDQPASDINW	0	0
O06188	AVPLRLVSVIKPTHPSPDDYD	0	0
O06188	_MSGRGEPTMKTIIVGIDGSH	0	0
O06188	AVLEYAAREAKLRQAPILALG	0	0
O06188	EAQSAVEAAGKLVKIETDIPR	0	0
O06188	LGSTATELAEKAHCPVAVMRS	0	0
O06391	VWTLRLYLTGKRLWHDPRRRD	0	0
O33294	AGTILHTGDIKFDQLPPDGRP	0	0
O33294	ASISGAVPVGKMFVDGLIAGD	0	0
O33294	DHIGAIPFLLKLRPDIPVVGS	0	0
O33294	DPKALEPAVRKVEAELESLVA	0	0
O33294	GTWRMLRANAKLAASTGVPQE	0	0
O33294	IAQGVRRTVGKWVGETYRRQP	0	0
O33294	LRPDIPVVGSKFTLALVAEKC	0	0
O33294	NGVSVDLVAGKASISGAVPVG	0	0
O33294	SKFTLALVAEKCREYRITPVF	0	0
O33294	AVFGVIDALSKIGARVVTNAQ	0	0
O33341	FVLAVQWHPEKSLDDLRLFKA	0	0
O53176	CGLALALDRDKLSDMRGVLTP	0	0
O53176	RMAQDGDPGYKATSVLLGECG	0	0
O53176	TDTNCVVRSFKGGFSGGTIAS	0	0
O53176	VLYGATGFVGKLTAEYLARAG	0	0
O53176	YIRLLPRGLVKRVVPKPGTGP	0	0
O53176	PRGLVKRVVPKPGTGPSAAAR	0	0
O53204	AARPHRVTHRKLTRG______	0	0
O53204	AFGFKGPDQQKPAGVLSGGER	0	0
O53204	AWEGDDDNEAKWFWFEGNFGA	0	0
O53204	DQARAGIDPRKTVWEVVSDGL	0	0
O53204	DVTLSFYPGAKIGVVGPNGAG	0	0
O53204	EFIYTMKKVRKAHGDKVILDD	0	0
O53204	EGNFGAYEENKVERLGVDAAR	0	0
O53204	EGNYSTYLEKKAERLAVQGRK	0	0
O53204	EMAAEAEKTRKLDFEEIQIPV	0	0
O53204	GGERRRVALCKLLLSKPDLLL	0	0
O53204	GNVEEGMGDIKIKLDRFNEVA	0	0
O53204	IGVVGPNGAGKSSVLRIMAGL	0	0
O53204	LQQEPPLNEDKTVRGNVEEGM	0	0
O53204	__MAEFIYTMKKVRKAHGDKV	0	0
O53204	_MAEFIYTMKKVRKAHGDKVI	0	0
O53204	MKKVRKAHGDKVILDDVTLSF	0	0
O53204	PNGVGKTTLFKTIVGLETPDS	0	0
O53204	RNRLNLALTLKQGGNLILLDE	0	0
O53204	RVALCKLLLSKPDLLLLDEPT	0	0
O53204	RYEEMAAEAEKTRKLDFEEIQ	0	0
O53204	SGAKARQAKSKARLQRYEEMA	0	0
O53204	SRAYVSAFGFKGPDQQKPAGV	0	0
O53204	SVLRIMAGLDKPNNGDAFLAT	0	0
O53204	VEEGMGDIKIKLDRFNEVAEL	0	0

O53204	VGVIGPNGVGKTTLFKTIVGL	0	0
O53204	VQGRKDAKLQKRLTEELAWVR	0	0
O53204	YEGNYSTYLEKKAERLAVQGR	0	0
O53204	EELAWVRSGAKARQAKSKARL	0	0
O53204	GLETPDSGSVKVGETVKLSYV	0	0
O53204	KAERLAVQGRKDAKLQKRLTE	0	0
O53204	NVVVEVDHLDKGYDGRALIKD	0	0
O53204	RLAVQGRKDAKLQKRLTEELA	0	0
O53204	SGSVKVGETVKLSYVDQARAG	0	0
O53204	VRSGAKARQAKSKARLQRYEE	0	0
O53226	GAYGEKQMYGKTVQGVIRSTF	0	0
O53226	GKIVVAQYNVKATGHVAKLRR	0	0
O53226	GLNVVGISPDKPEKLATFRDA	0	0
O53226	________MTKTTRLTPGDKA	0	0
O53226	RSTFVVDEDGKIVVAQYNVKA	0	0
O53226	VLTAWGAYGEKQMYGKTVQGV	0	0
O53226	VVGISPDKPEKLATFRDAQGL	0	0
O53226	YPAASTPGCTKQACDFRDNLG	0	0
O53442	AWGLAGEPSLKQFVTG_____	0	0
O53442	LGADIEAYRDKLQNVADAGIF	0	0
O53442	_______MAQKPVADALTLEL	0	0
O53442	NEDVRVQHVMKGYRAEKYTQV	0	0
O53442	QNVADAGIFGKPQLRQLISDR	0	0
O53442	TDACEILLILKDNLAGHHREL	0	0
O53618	GAELANYRRNKVGIVFQAFNL	0	0
O53618	LSCLGGILRPKSGAIKFDEVD	0	0
O53618	VMLLGPSGCGKTTLLSCLGGI	0	0
O53618	ELADGGEELVKVAGPGDYFGE	0	0
O53665	ALEGFTRSLGKELRRGATTAL	0	0
O53665	ATGITEPAGLKGLHEFFTPVL	0	0
O53665	DDSTPPADWEKPLDGKVAIVT	0	0
O53665	ESTMRFLLSAKSAYVDGQVFS	0	0
O53665	LDVTADDAVDKISEHLRDHHG	0	0
O53665	LVNNAGITRDKLLANMDDARW	0	0
O53665	_______MAPKRSSDLFSQVV	0	0
O53665	NRGQTNYATTKAGMIGITQAL	0	0
O53665	PADWEKPLDGKVAIVTGAARG	0	0
O53665	SEHLRDHHGGKADILVNNAGI	0	0
O53665	AAENLAETASKVGGTALWLDV	0	0
O53665	VVEPLRAALEKDYDLVGNNLG	0	0
O53871	AFASVVLKFQKDLNIPDEKLN	0	0
O53871	DTTKEGLAKLKPAFEGLAALG	0	0
O53871	EAIRTPRGKQKNGSLHEVKPL	0	0
O53871	FQKDLNIPDEKLNVNGGAIAM	0	0
O53871	HDEHMRPDTTKEGLAKLKPAF	0	0
O53871	IYEAIRTPRGKQKNGSLHEVK	0	0
O53871	KQKNGSLHEVKPLSLVVGLID	0	0
O53871	LGGFDDVALQKYHWVEKINHV	0	0

O53871	LNEAFASVVLKFQKDLNIPDE	0	0
O53871	LVMIGSAAAGKLQGLTPRARI	0	0
O53871	LVVGLIDELRKRHPDLDENLI	0	0
O53871	MLTGPTPATRKVLDRAGLTVD	0	0
O53871	RPDTTKEGLAKLKPAFEGLAA	0	0
O53871	VALQKYHWVEKINHVHTGGNS	0	0
O53871	GLEAVNTAAQKVRSGWDDLVL	0	0
O53871	VDAYALRSQQKAAEAWSGGYF	0	0
O69687	IFTATGQHPLKFTAYDGSTAG	0	0
O69687	KNYPFYFGFLKSKLRTGGLLL	0	0
O69687	LKTLTERVDFKRPSARVLANV	0	0
O69687	NLQLHHVLATKVDPRGDDSLP	0	0
O69687	RRMANGLLHSKTRDAEAIHHH	0	0
O69687	SIGLTEHIGVKNYPFYFGFLK	0	0
O69687	VHPGDPYELLKTLTERVDFKR	0	0
O69687	YPFYFGFLKSKLRTGGLLLNH	0	0
O69687	AVAEVGLPTAKVWGLYMAASR	0	0
O69687	EASLEQAQENKYRLIFEKLRL	0	0
O69687	LSAEQAKWGQKAVEDEGLSDL	0	0
O69687	QENKYRLIFEKLRLEPGDRLL	0	0
O86352	MTEPAAWDEGKPRIITLTMNP	0	0
O86352	QHISSGVFLLKASVRELRECV	0	0
O86352	SFTVNESRTAKQYRFVLPGPS	0	0
O86352	TSVDVVRPTEKMRCGAPRYDP	0	0
P09621	AKVNIKPLEDKILVQANEAET	0	0
P09621	ASGLVIPDTAKEKPQEGTVVA	0	0
P09621	GLVIPDTAKEKPQEGTVVAVG	0	0
P09621	________MAKVNIKPLEDKI	0	0
P09621	____MAKVNIKPLEDKILVQA	0	0
P09621	VAEGDTVIYSKYGGTEIKYNG	0	0
P09621	GPGRWDEDGEKRIPLDVAEGD	0	0
P09621	IYSKYGGTEIKYNGEEYLILS	0	0
P0A4X0	AAMSATEPLTKTTITRRDPGP	0	0
P0A4X0	GANFTYNSIGKDGQPTQGGYS	0	0
P0A4X0	GICHSDIHTVKAEWGQPNYPV	0	0
P0A4X0	GLGGLGHMGVKLGAAMGADVT	0	0
P0A4X0	PGPHDVAIDIKFAGICHSDIH	0	0
P0A4X0	SCTRGIEQYCKPGANFTYNSI	0	0
P0A4X0	YATADPDTFRKLRGGFDLILN	0	0
P0A4X0	DVTVLSQSLKKMEDGLRLGAK	0	0
P0A4X0	KMEDGLRLGAKSYYATADPDT	0	0
P0A4X0	VVTAVGSEVTKYRQGDRVGVG	0	0
P0A508	AAEVGYPVLIKPSAGGGGKGM	0	0
P0A508	GDVVVVVEAMKMEHSLEAPVS	0	0
P0A508	IEVMGDKIAAKNAVAAFDVPV	0	0
P0A508	LIKPSAGGGGKGMRLVQDPAR	0	0
P0A508	PARAIEVMGDKIAAKNAVAAF	0	0
P0A508	RECSLQRRHQKVIEEAPSPLL	0	0

P0A508	TWHLREAEEHKIHRAVGARPA	0	0
P0A508	VGSDYDPLLTKVIAHGADREE	0	0
P0A508	PARESYLDIGKVLDAAARTGA	0	0
P0A508	QVLVSVGDQVKVEQVLARIKD	0	0
P0A508	VKVEQVLARIKD_________	0	0
P0A520	AVVADKPEKEKASVPGGGDMG	0	0
P0A520	FLTTEAVVADKPEKEKASVPG	0	0
P0A520	GDEATGANIVKVALEAPLKQI	0	0
P0A520	IVKVALEAPLKQIAFNSGLEP	0	0
P0A520	KVTETLLKGAKEVETKEQIAA	0	0
P0A520	LKRGIEKAVEKVTETLLKGAK	0	0
P0A520	LLKGAKEVETKEQIAATAAIS	0	0
P0A520	LQAAPTLDELKLEGDEATGAN	0	0
P0A520	PLLEKVIGAGKPLLIIAEDVE	0	0
P0A520	TEAVVADKPEKEKASVPGGGD	0	0
P0A520	TVKDLLPLLEKVIGAGKPLLI	0	0
P0A520	ADLSLLGKARKVVVTKDETTI	0	0
P0A520	LENADLSLLGKARKVVVTKDE	0	0
P0A520	RGLNALADAVKVTLGPKGRNV	0	0
P0A520	SGLEPGVVAEKVRNLPAGHGL	0	0
P0A556	AFKIAGSQVLKKAAALAQPVI	0	0
P0A556	AGHIYAVIGLKDTTTGDTLSD	0	0
P0A556	ANVGKPQVAYKETIKRLVQNV	0	0
P0A556	ATKGKKERLGKLFQMHSNKEN	0	0
P0A556	DPVIEVAIEPKTKSDQEKLSL	0	0
P0A556	DQEKLSLSIQKLAEEDPTFKV	0	0
P0A556	EEGATYEFESKVTGGRIPREY	0	0
P0A556	FGIMAHIDAGKTTTTERILYY	0	0
P0A556	FKIAGSQVLKKAAALAQPVIL	0	0
P0A556	FKIATHPFFGKLTYIRVYSGT	0	0
P0A556	HEVDSSEMAFKIAGSQVLKKA	0	0
P0A556	IEPKTKSDQEKLSLSIQKLAE	0	0
P0A556	ILVDRMRREFKVEANVGKPQV	0	0
P0A556	ITSAATTTFWKDNQLNIIDTP	0	0
P0A556	IYPVLCGSAFKNKGVQPMLDA	0	0
P0A556	KPQVAYKETIKRLVQNVEYTH	0	0
P0A556	LDGAVAVFDGKEGVEPQSEQV	0	0
P0A556	LGKLFQMHSNKENPVDRASAG	0	0
P0A556	LVQNVEYTHKKQTGGSGQFAK	0	0
P0A556	MFGYVGDLRSKTQGRANYSMV	0	0
P0A556	PRICFVNKMDKIGADFYFSVR	0	0
P0A556	PVLCGSAFKNKGVQPMLDAVV	0	0
P0A556	QKLAEEDPTFKVHLDSETGQT	0	0
P0A556	QSEQVWRQADKYDVPRICFVN	0	0
P0A556	REFKVEANVGKPQVAYKETIK	0	0
P0A556	RILYYTGINYKIGEVHDGAAT	0	0
P0A556	RLVQNVEYTHKKQTGGSGQFA	0	0
P0A556	TVDEIKGAIRKLTIASEIYPV	0	0

P0A556	VAESDEHLLEKYLGGEELTVD	0	0
P0A556	VIEVAIEPKTKSDQEKLSLSI	0	0
P0A556	VLAGYPLVNLKVTLLDGAYHE	0	0
P0A556	VPPAIGHAPAKEDEEVVRKAT	0	0
P0A556	YDVPRICFVNKMDKIGADFYF	0	0
P0A556	ESGSQVINATKGKKERLGKLF	0	0
P0A556	GGEELTVDEIKGAIRKLTIAS	0	0
P0A556	GSQVINATKGKKERLGKLFQM	0	0
P0A556	GVVDLVEMNAKVWRGETKLGE	0	0
P0A556	KQTGGSGQFAKVIINLEPFTG	0	0
P0A556	LAEQAEEYRTKLLEVVAESDE	0	0
P0A556	_______MAQKDVLTDLSRVR	0	0
P0A556	MNAKVWRGETKLGETYDTVEI	0	0
P0A556	PANVSKEIIAKATGE______	0	0
P0A556	SQVINATKGKKERLGKLFQMH	0	0
P0A556	SYSEVPANVSKEIIAKATGE_	0	0
P0A556	TDEPFAALAFKIATHPFFGKL	0	0
P0A5B7	IMVRDGQLTIKAERTEQKDFD	0	0
P0A5B7	TRLMRLEDEMKEGRYEVRAEL	0	0
P0A5B7	VAVSEGKPTEKHIQIRSTN__	0	0
P0A5B7	DEDDIKATYDKGILTVSVAVS	0	0
P0A5F7	EWRNLEDEQEKMLARRLSGWQ	0	0
P0A5F7	FEYSRFQEAQKREIVEHSYLV	0	0
P0A5F7	QDRYPDVVVHKVVVCDRPAPR	0	0
P0A5H3	AAEERGYTATKHQREVGAGYF	0	0
P0A5H3	AYNCSPSFNWKKHLDDATIAK	0	0
P0A5H3	ERTREGFYRTKNGIEPCIARA	0	0
P0A5H3	GGALNVYELQKALIAAGVAGS	0	0
P0A5H3	HWEDQLASEKKCGHLGGKVLI	0	0
P0A5H3	MAVQQVRAGLKAIYLSGWQVA	0	0
P0A5H3	NALQRADQIAKIEGDTSVENW	0	0
P0A5H3	NGIEPCIARAKAYAPFADLIW	0	0
P0A5H3	SEKKCGHLGGKVLIPTQQHIR	0	0
P0A5H3	SHWEDQLASEKKCGHLGGKVL	0	0
P0A5H3	YNCSPSFNWKKHLDDATIAKF	0	0
P0A5H3	EAARQFSEAVKAEYPDQMLAY	0	0
P0A5H3	FQKELAAMGFKFQFITLAGFH	0	0
P0A5H3	KKHLDDATIAKFQKELAAMGF	0	0
P0A5H3	___MSVVGTPKSAEQIQQEWD	0	0
P0A5H3	QQEWDTNPRWKDVTRTYSAED	0	0
P0A5L2	AVGGAKNSVLKLMAATLLAEG	0	0
P0A5L2	CVLGPLVGRCKRARVALPGGD	0	0
P0A5L2	LSGEVAVGGAKNSVLKLMAAT	0	0
P0A5L2	SFRVTQYERPKAVNVATLPFP	0	0
P0A5L2	DPAHLQLVLHKLHDAGATVTQ	0	0
P0A5U4	AGIYGKLAWEKTIPNWFFEPD	0	0
P0A5U4	ALELAVAQIEKSYGKGSVMRL	0	0
P0A5U4	AVAQIEKSYGKGSVMRLGDEA	0	0

P0A5U4	FGSPETTTGGKALKFYASVRM	0	0
P0A5U4	GAIVWATPDHKVLTEYGWRAA	0	0
P0A5U4	GDGRDGWVGGKTPINFINVQR	0	0
P0A5U4	KQAEFDILYGKGISREGSLID	0	0
P0A5U4	__MTQTPDREKALELAVAQIE	0	0
P0A5U4	NRTRVKVVKNKCLAEGTRIFD	0	0
P0A5U4	PETTTGGKALKFYASVRMDVR	0	0
P0A5U4	QARLMSQALRKMTGALNNSGT	0	0
P0A5U4	RMDVRRVETLKDGTNAVGNRT	0	0
P0A5U4	SIVNGRRIQSKRQVFEVRISG	0	0
P0A5U4	STVRDYDPTQKRPSIVNGRRI	0	0
P0A5U4	TAIFINQLRDKIGVMFGSPET	0	0
P0A5U4	TNAVGNRTRVKVVKNKCLAEG	0	0
P0A5U4	TYEGEQLGQGKENARNFLVEN	0	0
P0A5U4	VASGDPRGGMKQVLGASRLRR	0	0
P0A5U4	VGNRTRVKVVKNKCLAEGTRI	0	0
P0A5U4	VQALADALDDKFLHDMLAEEL	0	0
P0A5U4	VVVHNCSPPFKQAEFDILYGK	0	0
P0A5U4	YGWRAAGELRKGDRVAQPRRF	0	0
P0A5U4	ADEIEKKIKEKLGIGAVVTDD	0	0
P0A5U4	AEHALDPDYAKKLGVDTDSLL	0	0
P0A5U4	DLCQQAGIYGKLAWEKTIPNW	0	0
P0A5U4	DMGVDQGLIRKSGAWFTYEGE	0	0
P0A5U4	DVADEIEKKIKEKLGIGAVVT	0	0
P0A5U4	EHALDPDYAKKLGVDTDSLLV	0	0
P0A5U4	HRIEDVVDGRKPIHVVAAAKD	0	0
P0A5U4	IEIYGPESSGKTTVALHAVAN	0	0
P0A5U4	NADVADEIEKKIKEKLGIGAV	0	0
P0A5U4	RKPIHVVAAAKDGTLHARPVV	0	0
P0A5Z4	ADVEIDVPAHKVTRNGEQISL	0	0
P0A5Z4	DDYIMKPFKPKELVARVRARL	0	0
P0A5Z4	EFDLLVALARKPRQVFTRDVL	0	0
P0A5Z4	GADDYIMKPFKPKELVARVRA	0	0
P0A5Z4	LESGADDYIMKPFKPKELVAR	0	0
P0A5Z4	SGVPIVMLTAKTDTVDVVLGL	0	0
P0A5Z4	VVLTVRGVGYKAGPP______	0	0
P0A5Z4	VNVHVQRLRAKVEKDPENPTV	0	0
P0CG95	LTLPASVDLEKETVITGRVVD	0	0
P0CG95	_____MCSGPKQGLTLPASVD	0	0
P0CG99	AFKYYTSYTLKTFDGKRYLER	0	0
P0CG99	AQIYAWRKGIKTLYYIRLRQM	0	0
P0CG99	ASKIEIRKEGKIGRVYYPAPY	0	0
P0CG99	ATPTFLNSGKKQRGEPVSCFL	0	0
P0CG99	DIHIPTQDDWKQLKESVQKHG	0	0
P0CG99	DQGLSLTLFFKDTATTRDVNK	0	0
P0CG99	DWKQLKESVQKHGIYNQNLQA	0	0
P0CG99	ERGTHFKGFEKSKYASGEFFD	0	0
P0CG99	FRFPTFLGAFKYYTSYTLKTF	0	0

P0CG99	GTHFKGFEKSKYASGEFFDKY	0	0
P0CG99	HPDIYRFLDTKRENADEKIRI	0	0
P0CG99	HPVASKIEIRKEGKIGRVYYP	0	0
P0CG99	IPTQDDWKQLKESVQKHGIYN	0	0
P0CG99	IQFEKDREAAKQYFLQHVNQN	0	0
P0CG99	KDTATTRDVNKAQIYAWRKGI	0	0
P0CG99	KIAIERGTHFKGFEKSKYASG	0	0
P0CG99	LDTKRENADEKIRIKTLSLGV	0	0
P0CG99	MVDDARIRKTKINAREFFQTL	0	0
P0CG99	NDDLSYAKVGKDISCNLGSLN	0	0
P0CG99	NQSSGVIPIMKLLEDSFSYAN	0	0
P0CG99	NTVFFHSQDEKLDYLIENEYY	0	0
P0CG99	RSINSALQLSKRGGGVALLLS	0	0
P0CG99	SCNLGSLNIAKAMDSPDFAQT	0	0
P0CG99	SEFNDDLSYAKVGKDISCNLG	0	0
P0CG99	SKYASGEFFDKYTDQVWEPKT	0	0
P0CG99	SNIREHGAPIKNIENQSSGVI	0	0
P0CG99	STSSIHPVASKIEIRKEGKIG	0	0
P0CG99	TDQVWEPKTDKVRRLFADADI	0	0
P0CG99	TSYTLKTFDGKRYLERFEDRV	0	0
P0CG99	TVNRSNPIAGKITHSNLCSEI	0	0
P0CG99	VNKAQIYAWRKGIKTLYYIRL	0	0
P0CG99	VPFADVSVTEKYYEMVDDARI	0	0
P0CG99	YDADGKIQFEKDREAAKQYFL	0	0
P0CG99	YEMVDDARIRKTKINAREFFQ	0	0
P0CG99	AMLNLYDADGKIQFEKDREAA	0	0
P0CG99	DKYTDQVWEPKTDKVRRLFAD	0	0
P0CG99	IKSLLDRAYAKKFRFPTFLGA	0	0
P0CG99	KSLLDRAYAKKFRFPTFLGAF	0	0
P0CG99	LAAGDTELAEKLVDEIIDGRF	0	0
P0CG99	PATPTFLNSGKKQRGEPVSCF	0	0
P0CG99	RENADEKIRIKTLSLGVVIPD	0	0
P0CG99	VIPDITFELAKKNEDMYLFSP	0	0
P0CG99	VLDQYSRDFIKSLLDRAYAKK	0	0
P0CG99	VLFHALRASNKIAIERGTHFK	0	0
P0CG99	YQDAYEIGYEKIIDTYAAATQ	0	0
P0CH36	GICHSDIHTVKAEWGVPNYPV	0	0
P0CH36	GLGGLGHVAVKLAKAMGADVT	0	0
P0CH36	LDLGAYLGLLKLDGALVELGL	0	0
P0CH36	LRHWNAGPGKKVAVIGLGGLG	0	0
P0CH36	PLRHWNAGPGKKVAVIGLGGL	0	0
P0CH36	TEVGSEVTKYKVGDRVGVGCF	0	0
P0CH36	VDSCRECDNCKAGLEQYCTGT	0	0
P0CH36	VVTEVGSEVTKYKVGDRVGVG	0	0
P0CH36	YATSDPETFDKLAGSFDLILN	0	0
P0CH36	AATSATEPLTKTTITRRAVGP	0	0
P0CH36	DVTVLSQSLKKMEDGLRLGAS	0	0
P0CH36	GLGHVAVKLAKAMGADVTVLS	0	0

P17670	AGHVNHTIWWKNLSPNGGDKP	0	0
P17670	AIADAFGSFDKFRAQFHAAAT	0	0
P17670	AVAKLEEARAKEDHSAILLNE	0	0
P17670	EDHSAILLNEKNLAFNLAGHV	0	0
P17670	HAFYLQYKNVKVDFAKAFWNV	0	0
P17670	MWEHAFYLQYKNVKVDFAKAF	0	0
P17670	QYKNVKVDFAKAFWNVVNWAD	0	0
P17670	YVKGANDAVAKLEEARAKEDH	0	0
P17670	AALGWDTLGNKLLIFQVYDHQ	0	0
P17670	HHSKHHATYVKGANDAVAKLE	0	0
P17670	SGQINELHHSKHHATYVKGAN	0	0
P17670	WKNLSPNGGDKPTGELAAAIA	0	0
P60176	ADIVVTATGNKDIIMLEHIKA	0	0
P60176	ADLSLADFGRKELRIAEHEMP	0	0
P60176	DKWTKIAESVKGVTEETTTGV	0	0
P60176	DVGKGCAEAMKGQGARVSVTE	0	0
P60176	EPKGVPVFAWKGETLEEYWWA	0	0
P60176	EQMLTWPDPDKPANMILDDGG	0	0
P60176	ERSGATRVNVKPQVDLWTFGD	0	0
P60176	EYDNEVYRLPKHLDEKVARIH	0	0
P60176	HVEALGGHLTKLTKEQAEYLG	0	0
P60176	IIMLEHIKAMKDHAILGNIGH	0	0
P60176	LRTRFETDKDKWTKIAESVKG	0	0
P60176	MLVLRGMQYEKAGVVPPAEED	0	0
P60176	___MTGNLVTKNSLTPDVRNG	0	0
P60176	NKDIIMLEHIKAMKDHAILGN	0	0
P60176	NLLRTRFETDKDKWTKIAESV	0	0
P60176	NRGTDALIGGKKVLICGYGDV	0	0
P60176	PAEEDDPAEWKVFLNLLRTRF	0	0
P60176	RFETDKDKWTKIAESVKGVTE	0	0
P60176	RGTDALIGGKKVLICGYGDVG	0	0
P60176	RREYAEVQPLKGARISGSLHM	0	0
P60176	VGPHGTPDEPKGVPVFAWKGE	0	0
P60176	VLICGYGDVGKGCAEAMKGQG	0	0
P60176	VYRLPKHLDEKVARIHVEALG	0	0
P60176	YLGVDVEGPYKPDHYRY____	0	0
P60176	QTIAQIELWTKNDEYDNEVYR	0	0
P60176	TPDVRNGIDFKIADLSLADFG	0	0
P60796	EGVFVGSGIFKSGAPEHRAAA	0	0
P60796	GNPATGTARVKRGMAEMLKGG	0	0
P60796	IAAVTIPVMAKVRIGHFVEAQ	0	0
P60796	ISEGAAMIRSKGEAGTGDVSN	0	0
P60796	MSEDELFVAAKELQAPYELVA	0	0
P60796	TPADYAHHIDKWNFTVPFVCG	0	0
P60796	APEHRAAAIVKATTFFDDPDV	0	0
P60796	ELVAEVARAGKLPVTLFTAGG	0	0
P63345	AKAVANSLAKKMAEVRGDDAH	0	0
P63345	ARNSKLMETLKEARQQLLALR	0	0

P63345	CSPNIDAASLKKGQTVRLNEA	0	0
P63345	DAEAAQDIYSKYLTEFLPVHA	0	0
P63345	DTVDVFTSGRKMRLTCSPNID	0	0
P63345	EFDGDRSACIKAMIEKVVDRM	0	0
P63345	EVRGDDAHEAKSYFLNIKGPE	0	0
P63345	HEAKSYFLNIKGPELLNKFVG	0	0
P63345	LYREYSLRPPKGVLLYGPPGC	0	0
P63345	RDAVELPFLHKELYREYSLRP	0	0
P63345	RIDSLAARNSKLMETLKEARQ	0	0
P63345	RSACIKAMIEKVVDRMYAEID	0	0
P63345	SPNIDAASLKKGQTVRLNEAL	0	0
P63345	AMIQNVVDRAKKNAIKSVLET	0	0
P63345	EALNDDTRPRKLRPGDSLLVD	0	0
P63345	MIQNVVDRAKKNAIKSVLETG	0	0
P63345	VVDRAKKNAIKSVLETGQPGL	0	0
P63458	ELARRCVLAGKDVIVAGHSVG	0	0
P63458	ELRGVPARAVKSPADLDELAN	0	0
P63458	LAATRGAEMAKACATEPTGMS	0	0
P63458	PPAGTLSGIAKRELRGVPARA	0	0
P63458	TATLLSNRDGKPVTSAAAAMD	0	0
P63458	GAADQIAAWSKAADLDLARLG	0	0
P63458	LEKLAEDPPAKARVRALGVAG	0	0
P63523	AISVPVDHTEKLVTVIELKLL	0	0
P63523	EDEMFIVGRMKDMLIVYGRNH	0	0
P63523	GDNVAEGYWNKPDETRHTFGA	0	0
P63523	GEAMDELDVIKNNVTAAISRS	0	0
P63523	LTGNSPSFRVKDLPSAAYLQY	0	0
P63523	PAGTIGEIWVKGDNVAEGYWN	0	0
P63523	PGSIPTTTSGKIRRAACVEQY	0	0
P63523	TEKLVTVIELKLLGDSAGEAM	0	0
P63523	LYVASRNSGDKPEVVYFEPDK	0	0
P63523	PNFAFELAVRKTTDADIEGLD	0	0
P63523	STGSANRCEPKTGTPLLSYGM	0	0
P63568	FKLSRLTGRTKLVAAHDAFHG	0	0
P63568	GITPDVVTLAKGLGGGLPIGA	0	0
P63568	GSLALTGQPAKQTPFAPLPGD	0	0
P63568	LGIALTAPHAKDAEATARDAG	0	0
P63568	SGAEANEAAFKLSRLTGRTKL	0	0
P63673	AIAQHWMYEGKHVLIIFDDLT	0	0
P63673	AVKVKKPAPKKKK________	0	0
P63673	DEDKLAKEAVKVKKPAPKKKK	0	0
P63673	DEHVEALDEDKLAKEAVKVKK	0	0
P63673	DKLAKEAVKVKKPAPKKKK__	0	0
P63673	EAVKVKKPAPKKKK_______	0	0
P63673	ERGARLVELLKQPQSQPMPVE	0	0
P63673	FENIEEGQQVKRTGEVLSVPV	0	0
P63673	GQRQLIIGDRKTGKTAVCVDT	0	0
P63673	HVLIIFDDLTKQAEAYRAISL	0	0

P63673	KLAKEAVKVKKPAPKKKK___	0	0
P63673	KVKKPAPKKKK__________	0	0
P63673	LHSRLLERCAKLSDDLGGGSL	0	0
P63673	QLIIGDRKTGKTAVCVDTILN	0	0
P63673	QRQNWESGDPKKQVRCVYVAI	0	0
P63673	RQNWESGDPKKQVRCVYVAIG	0	0
P63673	SLTGLPIIETKANDISAYIPT	0	0
P63673	VEALDEDKLAKEAVKVKKPAP	0	0
P63673	VGGAAQIKAMKEVAGSLRLDL	0	0
P63673	VKVKKPAPKKKK_________	0	0
P63673	VSRVGGAAQIKAMKEVAGSLR	0	0
P63673	AAAASESAGFKWLAPYTGSAI	0	0
P63673	AFASDLDAASKAQLERGARLV	0	0
P63673	APSVVHRQGVKEPLQTGIKAI	0	0
P63673	DKLTEVIKNFKKGFAATGGGS	0	0
P63673	EAADKLTEVIKNFKKGFAATG	0	0
P63673	EILTEIRDSQKLTEEAADKLT	0	0
P63673	GVKEPLQTGIKAIDAMTPIGR	0	0
P63673	KLTEVIKNFKKGFAATGGGSV	0	0
P63673	VRCVYVAIGQKGTTIAAVRRT	0	0
P64245	AVTNQWVNSYKRLVQGGEAPT	0	0
P64245	DDPLQLSEVGKSFIAGILEHA	0	0
P64245	DNVMTFRYVIKEVALEEGARA	0	0
P64245	EEGARASFMPKPFGQHPGSAM	0	0
P64245	EHVFDFFLRNKRTEWANYRSH	0	0
P64245	LVRVPMYTPHKTSSRRVEVRS	0	0
P64245	LWFTDVLGFLKSVAIAPAELE	0	0
P64245	VHPEIEFFLLKPGPEDGSVPV	0	0
P64245	PRHVLRRQLTKAGELGFSCYV	0	0
P65161	KQLNKLINVIKIVEQDDEHSV	0	0
P65161	LTVEATGNRGKLEALLRVLEP	0	0
P65161	SRGPRGIGTAK__________	0	0
P65161	THTLSVLVEDKPGVLARVAAL	0	0
P65161	AEDTPLEQITKQLNKLINVIK	0	0
P65161	HSVSRELALIKVQADAGSRSQ	0	0
P65161	_______MSPKTHTLSVLVED	0	0
P65232	ISARLIADLLKTAGADRIVTV	0	0
P65232	LGGVPLAFIHKTRDPRVPNQV	0	0
P65232	MIDALKRGSAKRITAVMPFYP	0	0
P65232	MPFYPYARQDKKHRGREPISA	0	0
P65232	_MSHDWTDNRKNLMLFAGRAH	0	0
P65232	PDSGRVRIAEKWADALGGVPL	0	0
P65232	PFYPYARQDKKHRGREPISAR	0	0
P65232	MEQLIMIDALKRGSAKRITAV	0	0
P65232	VTNTLPIGEDKRFPQLTVLSI	0	0
P65277	EAFEGACAYWKPRTLPAP___	0	0
P65277	GLDDIALTLRKLDEIEAFEGA	0	0
P65277	TDQIIPAVFLKRVTRTGFEDG	0	0

P65277	FGDIFRGNAGKAGLLAAEVAQ	0	0
P65277	VAQDDVELLWKLIEQSPGLEI	0	0
P65573	LVDSLRSDTPKAPTRGAPLCG	0	0
P65573	MGGDAIFDRLKEHLGVGHDET	0	0
P65573	PTLAGLQVARKNDMQAPPTPG	0	0
P65880	ACEFKNQVLVKIYNRKALDPA	0	0
P65880	DKVTERYMGSKKIGTTGRGIG	0	0
P65880	DVLDPEQLTHKVEAACEFKNQ	0	0
P65880	GAQWGDEGKGKATDLLGGRVQ	0	0
P65880	GLQDRGVDTAKLLISADAHLL	0	0
P65880	GRGIGPCYQDKIARIGIRVAD	0	0
P65880	KVTERYMGSKKIGTTGRGIGP	0	0
P65880	LIGAQWGDEGKGKATDLLGGR	0	0
P65880	LLMPYHIAIDKVTERYMGSKK	0	0
P65880	PMTQRDLCRAKPVYEELPGWW	0	0
P65880	THKVEAACEFKNQVLVKIYNR	0	0
P65880	TRIGTVLGILKAYTTRVGSGP	0	0
P65880	VNGITDYFLTKLDVLSSLESV	0	0
P65880	DALLEQAEGFKHRIADTRLLL	0	0
P65880	GAREFDDLPAKARDYVLRLEQ	0	0
P65880	NQVLVKIYNRKALDPAQVVDA	0	0
P66056	APKKKVAGLIKLQIVAGQANP	0	0
P66056	DQVREIAETKKTDLNANDVDA	0	0
P66056	GVAKGSAEPHKTKVAKVTWDQ	0	0
P66056	KLLLKAAGVAKGSAEPHKTKV	0	0
P66056	KTPPAAKLLLKAAGVAKGSAE	0	0
P66056	LNANDVDAAAKIIAGTARSMG	0	0
P66056	_______MAPKKKVAGLIKLQ	0	0
P66056	______MAPKKKVAGLIKLQI	0	0
P66056	_____MAPKKKVAGLIKLQIV	0	0
P66056	QHGVNIMEFCKAYNAATENQR	0	0
P66056	TFTLKTPPAAKLLLKAAGVAK	0	0
P66056	WDQVREIAETKKTDLNANDVD	0	0
P66056	YEDRSFTFTLKTPPAAKLLLK	0	0
P66056	AKGSAEPHKTKVAKVTWDQVR	0	0
P66902	GNAGNITAYWKGYTEYHQLGL	0	0
P66902	KLAQAVMHGAKIIQIDGNFDD	0	0
P66902	NGLKDPDTALKDMPSVSPVPV	0	0
P66902	SKQTGCTIHLKVEGLNPTGSF	0	0
P66902	SVNPVRIEGQKTAAFEIVDVL	0	0
P66902	TSAVEAQQQSKGRFLAASDEE	0	0
P66902	VEGLNPTGSFKDRGMTMAVTD	0	0
P66902	VVCTVTGNGLKDPDTALKDMP	0	0
P66902	ASAASIAGLLKAIDDGWVARG	0	0
P66902	TEYHQLGLIDKLPRMLGTQAA	0	0
P66902	TPLIAATNLSKQTGCTIHLKV	0	0
P66902	VPVDPVAVVEKLGLA______	0	0
P69440	DTDDVILNRMKVYRDETAPLL	0	0

P69440	EEGTKLGVEAKRYLDAGDLVP	0	0
P69440	LFRRNIEEGTKLGVEAKRYLD	0	0
P69440	PLLEYYRDQLKTVDAVGTMDE	0	0
P69440	VLLLGPPGAGKGTQAVKLAEK	0	0
P69440	VSEEVLLERLKGRGRADDTDD	0	0
P69440	KGTQAVKLAEKLGIPQISTGE	0	0
P69440	PGAGKGTQAVKLAEKLGIPQI	0	0
P69440	VFARALRALGK__________	0	0
P71703	AIFASENNTIKIADVAPTNVI	0	0
P71703	ARVPIVGDDIKSQVGATITHR	0	0
P71703	AVTSNLKREFKTKDVHIGPSD	0	0
P71703	FGDVPLNLEYKLEVWDSPNSA	0	0
P71703	FIASDPVWAKKFTDARVPIVG	0	0
P71703	GATITHRVLAKLFEDRGVQLD	0	0
P71703	IDAVRAAKIAKDRGIGGPVIP	0	0
P71703	MLERERLESKKISKTQAVTSN	0	0
P71703	NMLERERLESKKISKTQAVTS	0	0
P71703	PVIPASAYLMKSPPEQLPDDI	0	0
P71703	QRGPTLDGIGKYYADTIELSD	0	0
P71703	RERLESKKISKTQAVTSNLKR	0	0
P71703	RFGPYHVRDVKFVAAFDVDAK	0	0
P71703	SDHVGWLDDRKWAYVRLEGRA	0	0
P71703	SKTQAVTSNLKREFKTKDVHI	0	0
P71703	TSNLKREFKTKDVHIGPSDHV	0	0
P71703	VDVVQALKEAKVDVLVSYLPV	0	0
P71703	VFIASDPVWAKKFTDARVPIV	0	0
P71703	AEPVDVVQALKEAKVDVLVSY	0	0
P71703	FVAAFDVDAKKVGFDLSDAIF	0	0
P71703	GVIIDAVRAAKIAKDRGIGGP	0	0
P71703	YLPVGSEEADKFYAQCAIDAG	0	0
P71724	LIAALSAALLKLPVANWPALG	0	0
P71724	DLRRAYDTAVKLGERTGLVVR	0	0
P71973	AEHGMAARGYKDVRANTVPAF	0	0
P71973	VGLHRPAEFNKSHIPAFLAGE	0	0
P77899	GVLPYLRPDGKTQVTIAYEDN	0	0
P77899	ILEIGYDSSDKGFDGATCGVN	0	0
P77899	_______MSEKGRLFTSESVT	0	0
P77899	RLSRRLTEVRKNGVLPYLRPD	0	0
P77899	TQHAADIDLEKTLDPDIREKV	0	0
P77899	TVRVLVNPTGKFVLGGPMGDA	0	0
P77899	DVELPWEQLDKVDDLKRAI__	0	0
P77899	EKTLDPDIREKVLNTVLDDLA	0	0
P77899	HVVGEVTTSAKEAFADITNTV	0	0
P77899	TETEDPVKIEKAIGEVFDLRP	0	0
P77899	WEQLDKVDDLKRAI_______	0	0
P96382	PGTRMRSDTPKVLHTLAGRSM	0	0
P96382	QRNIENWVQRKRPGSPAAQAS	0	0
P96382	RPGSPAAQASKRASEMACQQP	0	0

P96382	SVFVNYDGTSKRRTTVGSHVR	0	0
P96382	MLSHVLHAIAKLAPQRLIVVL	0	0
P96382	RPGTALGADGKLGAFVEVKNS	0	0
P96382	VKNSTIGTGTKVPHLTYVGDA	0	0
P96825	AGILRDGTFHKMSFENWDAVL	0	0
P96825	ATEDGAANIIKTALDEFGAVH	0	0
P96825	LFGNDGANFDKPPSVQDVAAR	0	0
P96825	LINTLALEGAKYNIHANALAP	0	0
P96825	MSFENWDAVLKVHLYGGYHVL	0	0
P96825	NASVYVVGGGKVQRVALFGND	0	0
P96825	NFGQTNYGAAKLGLVGLINTL	0	0
P96825	QDILPPEVLEKLTPEFVAPVV	0	0
P96825	ADEVVAEIRDKGGRAVANYDS	0	0
P96825	DLSGAKIAGFKL_________	0	0
Q10504	AKTIKGYALGKHFEGRNATHQ	0	0
Q10504	ATTMATVRTFKEVLRDKQIGP	0	0
Q10504	AVDADLMLAYKESEVGQILHE	0	0
Q10504	EGRNATHQMKKLTLEDLKEFR	0	0
Q10504	EVGFNHFFRGKSHPGGGDQVF	0	0
Q10504	FEGRNATHQMKKLTLEDLKEF	0	0
Q10504	FNHYLHDRGIKDTSDQHVWCF	0	0
Q10504	GFVPERRTKSKALTLPGRDIY	0	0
Q10504	HKGQPTVILAKTIKGYALGKH	0	0
Q10504	LGGFVPERRTKSKALTLPGRD	0	0
Q10504	LPGRDIYAPLKKGSGHQEVAT	0	0
Q10504	NLKRGGHDYRKVYAAYRAAVD	0	0
Q10504	NMSDQDIWNLKRGGHDYRKVY	0	0
Q10504	NRDGVAIETEKLRHPDRPAGV	0	0
Q10504	PGRDIYAPLKKGSGHQEVATT	0	0
Q10504	QMKKLTLEDLKEFRDTQRIPV	0	0
Q10504	QPTVILAKTIKGYALGKHFEG	0	0
Q10504	TTPDGDYQTYKANDGGYVRDH	0	0
Q10504	VRTFKEVLRDKQIGPRIVPII	0	0
Q10504	YAAYRAAVDHKGQPTVILAKT	0	0
Q10504	YHAATEQRTNKAQILASGVAM	0	0
Q10504	FFRGAGWNVIKVVWGREWDAL	0	0
Q10530	ALTDDYFIERKLYPNVDFYTG	0	0
Q10530	DDVSEFVRKVKNREAGVKLMG	0	0
Q10530	GGDDSLLGIAKELEEAALTDD	0	0
Q10530	KLMGFGHRVYKNYDPRARIVK	0	0
Q10530	KLPTIAAYAYKKSVGQPFLYP	0	0
Q10530	LPTIAAYAYKKSVGQPFLYPD	0	0
Q10530	QRHTMLHEDLKRFFDGFPRNA	0	0
Q10530	RGYPIDQLAEKSTFIEVCYLL	0	0
Q10530	RKVKNREAGVKLMGFGHRVYK	0	0
Q10530	VELSTIRLLAKLPTIAAYAYK	0	0
Q10530	VKEQADKILAKLGGDDSLLGI	0	0
Q10530	WREMHDEGDSKIGRPRQIYTG	0	0

Q10530	DGIALGPLLAKTGHTTFDVGF	0	0
Q10530	DVGFANTAAAKSSITYIDGDA	0	0
Q10530	KNYDPRARIVKEQADKILAKL	0	0
Q10530	SGDDVSEFVRKVKNREAGVKL	0	0
Q10682	ARVITTGGSQKAVNADRSGLA	0	0
Q50685	AIACKDPEKKKKLMTDIFALA	0	0
Q50685	AYVLEGGSARKLIEAREKAFW	0	0
Q50685	DFDQVMAAVPKILASGLAPDI	0	0
Q50685	DGNVHMAIACKDPEKKKKLMT	0	0
Q50685	EAREKAFWAAKALGADDIIDT	0	0
Q50685	GLLISFDRMNKVLEVDTANQV	0	0
Q50685	GPPQKPAYAAKPATPEEVAQL	0	0
Q50685	GTNAGGMRAVKYGVARHNVLG	0	0
Q50685	GYFLELEDPVKISLMRRIKQS	0	0
Q50685	HDEELTGPPQKPAYAAKPATP	0	0
Q50685	HMAIACKDPEKKKKLMTDIFA	0	0
Q50685	IACKDPEKKKKLMTDIFALAM	0	0
Q50685	ISGEHGVGRAKTGYFLELEDP	0	0
Q50685	MAIACKDPEKKKKLMTDIFAL	0	0
Q50685	PVKISLMRRIKQSFDPAGILN	0	0
Q50685	ATPEEVAQLLKAASENGVPVT	0	0
Q50685	EIIRTGGRMAKVSTGYDLTQL	0	0
Q50685	SARKLIEAREKAFWAAKALGA	0	0
Q50685	TLALVTEVIVKLHPRLDHNAS	0	0
Q7D8W0	GGSSKDRIAVKMIEAAEASGQ	0	0
Q7D8W0	GPEIWADTEGKVTHFVAGIGT	0	0
Q7D8W0	GTITGAGRYLKEVSGGRVRIV	0	0
Q7D8W0	GYKCVFVCPDKVSEDKRNVLI	0	0
Q7D8W0	IEAAEASGQLKPGGTIVEPTS	0	0
Q7D8W0	LALVAQRRGYKCVFVCPDKVS	0	0
Q7D8W0	LPDGGRGYMSKIFNDAWMSSY	0	0
Q7D8W0	RLVRDIDGAWKPDQYANPEGP	0	0
Q7D8W0	SCGMAVVAALKVAEEAGPDAL	0	0
Q7D8W0	VEYLNPGGSSKDRIAVKMIEA	0	0
Q7D8W0	WDALMVVEEGKPVGVITRYDL	0	0
Q7D8W0	FVCPDKVSEDKRNVLIAYGAE	0	0
Q7D8W0	LLSAVFEGRAKLADAVSAHMS	0	0
Q7D8W0	QSTVGDVLRRKSGALPALVHT	0	0
Q7D8W0	VPDGAGTVAAKVEYLNPGGSS	0	0

Table S3. The prediction results of PUL-PUP for the training dataset.

Protein Name	Peptide	Class label	Prediction label
A0QNF6	FGEVVDEESQKVVDAIASTPT	1	1
A0QP32	DYFAHWINVGKNADESKLPKV	1	1
A0QP90	AGATRIIGVAKAGLDDAGYRN	1	1

A0QPN2	ANADTVTLWSKLALESARARR	1	1
A0QQ65	NMKYADGTPIKASDFTYAIQR	1	1
A0QQH7	LFDEHGAYLAKTGGEVGVTTG	1	1
A0QQU5	AVEKVTETLLKSAKEVETKEQ	1	1
A0QQU5	VAAGANPLGLKRGIEKAVEKV	1	1
A0QS45	SGEPHKTKVAKVTWDQVREIA	1	0
A0QS98	KALEGDPKWVKSVEELMEAVD	1	1
A0QSE0	KRWRLVEILEKAK________	1	0
A0QSH8	KGTQAEKLSEKLGIPQISTGD	1	1
A0QSL6	SGHVGFKGSRKSTPFAAQLAA	1	1
A0QSU4	KIAQVVEVAAKEPEPSAAIRL	1	0
A0QUV7	GRGVGSAENFKIVEELADSLG	1	0
A0QUX7	QSLAVGATEQKDMSRMTIVVS	1	1
A0QUY3	GQTEAAARVDKAVSEHLATRG	1	1
A0QUY9	RVVADVLRGRKVADGVRMLIV	1	1
A0QUZ0	SFILNLSPFDKGSVLVAGPDF	1	1
A0QV10	ITSIASLETGKRLGPDPRTFN	1	1
A0QVB9	ALVEADGDFDKAVELLRIKGA	1	1
A0QVB9	ANDIETLKAAKTGDTTVEQAI	1	1
A0QVB9	IADLSAKIGEKLELRRAAYFD	1	1
A0QVM0	YSFDVDDDGLKNAYYQHREAY	1	1
A0QW25	DVIDEAATFLKQQEDKGVVVR	1	0
A0QWG8	TFYDDPDVLAKVSRGLGEAMV	1	0
A0QWT3	TFGSETVDPAKIEKAIGEVFD	1	0
A0QWT3	WEQTNKVDDLKSAI_______	1	1
A0QWV9	GAARRLGVSAKAREVRGSDRV	1	1
A0QWW2	SVTDLTAELAKSASVEDINAA	1	0
A0QWX9	KTILQAIELWKIVDRPNLLIK	1	1
A0QWX9	VDTEIDARLEKIGSDEALALR	1	1
A0QX20	ELTDAKNAFRKDIHNYVEQNH	1	1
A0QX81	GGVDVDAVVPKVRPIVDAVAQ	1	1
A0QX93	ATEEEDVLLEKELLADEKERA	1	1
A0QXX7	NSWPDNVSLDKARRLLWPIKQ	1	1
A0QYD4	ARRDDLEVAAKELDIESIVFD	1	1
A0QYS9	DLVARAGVLGKTLSHGIEELG	1	1
A0QZ54	IVYIRTLVTGKSSSASRAIDT	1	1
A0QZA1	SVARTLIDASKSAQMVVVGNR	1	1
A0QZE3	IHIEQGRELEKNNVTIGLVDR	1	1
A0R066	ERKIDVDEWQKKAGAGEITEV	1	1
A0R079	KTSDDIFKLIKDENVEYVDIR	1	1
A0R083	LLAAGLRGVEKGYVLGPQAED	1	0
A0R0B0	DHFFGRDPRTKALVADMSDQE	1	1
A0R0B2	VAAGAVAALDKLAEDPPAKAR	1	1
A0R0B3	AVQTEDKYGVKIPDEDLAGLR	1	1
A0R0B3	TVGDVVAYIQKLEEENPEAAA	1	1
A0R0B4	IRVLEDEFVTKWDLPVRIGGH	1	1
A0R0B5	VELRRLSYLQKMATVLGRRVW	1	1
A0R0W1	RAQVLVPELEKRLDDINAQLD	1	0

A0R0W4	DVADRIAVLNKGRIEQIGSPT	1	1
A0R1B5	AIEVVYGADVKGRGIESAAPL	1	1
A0R1V9	LTAVVGGDLSKVDAKQPDDYF	1	1
A0R1V9	PDDYFTRVTSKDYEGKWRIIF	1	1
A0R1V9	VGGDLSKVDAKQPDDYFTRVT	1	1
A0R1Y7	QSHQKAAAAWKDGVFADEVVP	1	1
A0R218	DVYVSMNMVRKNGLRRGDAVT	1	1
A0R220	NFDDCLELARKLTADFPTIAL	1	1
A0R2G5	NSLSGAVEVVKRDGTGITLNE	1	1
A0R2V7	NAQALADGFVKRDAGLVTGGT	1	1
A0R2W6	IGRQAIEGFYKNMTAATTDIK	1	1
A0R342	HPDIPAAATVKPGSEFRIECK	1	1
A0R3D2	LTANVRTRTGKGASRQARRDG	1	1
A0R3D6	VVVLGPGSIPKTPSGKLRRAN	1	1
A0R4C9	RDFVDAQQFSKLLSERGIAND	1	1
A0R4D0	TGAGIHEVDVKVA________	1	1
A0R4Z5	DEGPRETTLEKMAALQPLVEG	1	1
A0R518	EDLAETVELVKGLNRRIVAEE	1	1
A0R566	MPNSNPVAAWKALKDGNARFV	1	1
A0R5E1	SGFRTLEENQKVEFEVGQSPK	1	0
A0R5M8	FDLDAFEHVAKWNSDEERIEM	1	1
A0R5R5	LSREQATWAQKAIAQEGLTDL	1	1
A0R5R7	EILEDRLGESKIASWTDPKGG	1	1
A0R635	HNETVVLDFPKRVVREPIPDE	1	1
A0R647	_MTNSGALDTKFHALIQDQIR	1	1
A0R652	HHSKHHATYVKGVNDAIAKLE	1	1
A0R652	WKNLSPNGGDKPTGELAAAID	1	1
A0R6E3	PATEAETAQIKDLIAALGG__	1	1
A0R6E3	RAAVVLSGLRKLQGSLAEAAS	1	0
A0R6Q0	IKTADVDAAAKEIEATGGAII	1	1
A0R7F9	VIRKDGGTVDKVDIWGRRRLA	1	1
A0R7G6	NFVAAFDVDAKKVGFDLSEAI	1	1
A4ZHR8	ALGGTLTKLSKDQAEYIGVDV	1	1
EG10025	APFAGTVKEIKVNVGDKVSTG	1	1
EG10207	FADYDEARFQKEGFRVVPPAA	1	0
EG10251	DVKILDPRVGKEFPLPTYATS	1	1
EG10367	SVVDLTVRLEKAATYEQIKAA	1	0
EG10407	QEIFTREEMIKYFTLNAVSKS	1	1
EG10410	LAMQVVVLRDKLEPYFTEGRY	1	1
EG10416	SAEQVDPRDEKVANLEAQLAE	1	1
EG10416	TRERDGILRVKAEMENLRRRT	1	1
EG10489	_______MESKVVVPAQGKKI	1	1
EG10489	WLKVKNPNTGKEIVIKDVIAD	1	1
EG10492	MNDAFWDELLKVRGEVNKVIE	1	1
EG10762	YLRWAESRGFKTEIIEESEGE	1	1
EG10770	XXXDIVEYLVKQIKG______	1	1
EG10770	KSFRVQELAEKLYSELRAQGI	1	1
EG10770	QLGTKYSEALKASVQGEDGRN	1	1

EG10803	EGKVFLNIGDKFLLDANLGKG	1	1
EG10803	RADKVREIAAKLGRHVAILGD	1	1
EG10876	ARAAIEAAGGKIEE_______	1	0
EG10903	AALELAEQREKPTWLEVDAGK	1	1
EG10903	REKAKKQSRVKAALELAEQRE	1	1
EG10905	YTAAITGAEGKIHRLEDWGRR	1	1
EG10954	TYVTNLNNLIKGTAFEGKSLE	1	1
EG11192	GELILNEKLAKQLVTAANWVK	1	1
EG11223	HSWVEGKLIDKVGQLGKKLHT	1	0
EG11415	TQVINSKTPLKSYPLDIHNVQ	1	1
EG11427	AWNEKFAAYAKAYPQEAAEFT	1	1
EG11427	FDAKAKEFIAKLQANPAKIAS	1	1
EG11518	LQLGKDLDRFKNLVLVHAARY	1	1
EG11615	EYALLSRLMLKAGSPVHREIL	1	1
EG11615	LHFLARIRQKKYTLPVLILTA	1	1
EG12204	__MAKIKGQVKWFNESKGFGF	1	1
EG12411	RKAGIPLALNKLNAVPTTAYP	1	0
EG12503	EGVKYLEENAKKEGVNSTESG	1	0
EG12545	PNYLHKEPVIKAAKNKKHVFC	1	0
EG12669	GEIAADFAQDKKSDFDLTPFR	1	1
EG12712	INSNEELALPKEKLQELHI__	1	1
EG12982	________MQKVVLATGNVGK	1	1
EG13014	LITYGGGSVKKTGVLDQVLDA	1	1
EG13952	AGGIYLFTDEKSALAYLEKHT	1	0
EG13952	AYLEKHTARLKNLGVEEVVAK	1	1
O05598	VVVLGPGTIPKTPSGKLRRAN	1	1
O05814	YSFDIDAAGLKAAYHAHREAY	1	1
O06188	SAVEAAGKLVKIETDIPRGPA	1	1
O06391	IAARMTTAVDKARVRGAGHEV	1	0
O33294	LHSRGFSEDPKALEPAVRKVE	1	1
O33341	EKSLDDLRLFKALVDAASGYA	1	1
O53176	FMRNSIDLYHKQAADTGARIV	1	0
O53204	DKGYDGRALIKDLSFSLPRNG	1	1
O53226	TKTTRLTPGDKAPAFTLPDAD	1	1
O53226	YNVKATGHVAKLRRDLSV___	1	1
O53442	QHVMKGYRAEKYTQVETLVYM	1	0
O53618	GILRPKSGAIKFDEVDITTLQ	1	1
O53665	TALVYLSPDAKPAATGLESTM	1	1
O53665	TQALAPGLAAKGITINAVAPG	1	1
O53871	AEAWSGGYFAKSVVPVRDQNG	1	0
O69687	IGATLSAEQAKWGQKAVEDEG	1	1
O86352	VGLSRGWSLIKSVRLGNAAGA	1	1
P09621	SARDVLAVVSK__________	1	1
P0A508	EWQLRVGAGEKLGFAQNDIEL	1	1
P0A520	AVEKVTETLLKGAKEVETKEQ	1	0
P0A556	PAKEDEEVVRKATTDEPFAAL	1	1
P0A5B7	LPVGADEDDIKATYDKGILTV	1	0
P0A5B7	LTIKAERTEQKDFDGRSEFAY	1	1

P0A5B7	LTVSVAVSEGKPTEKHIQIRS	1	1
P0A5B7	RAELPGVDPDKDVDIMVRDGQ	1	1
P0A5F7	QAHQIVEQAHKVALEASSSGR	1	1
P0A5H3	LDDATIAKFQKELAAMGFKFQ	1	1
P0A5L2	VARITAPDEPKYDADFAAVRQ	1	1
P0A5U4	ENADVADEIEKKIKEKLGIGA	1	1
P0A5Z4	HVQRLRAKVEKDPENPTVVLT	1	1
P0CG95	SGAGIHEVDVKIT________	1	1
P0CG99	IPDITFELAKKNEDMYLFSPY	1	1
P0CH36	ADVTVLSQSLKKMEDGLRLGA	1	0
P17670	SRYAAATSQTKGLIFG_____	1	0
P60176	ALGGHLTKLTKEQAEYLGVDV	1	1
P60796	TFFDDPDVLAKVSRGLGEAMV	1	1
P63458	VAAGRLTALEKLAEDPPAKAR	1	1
P63523	KPEVVYFEPDKLSTGSANRCE	1	1
P63568	GLVRRAEVLGKSLRHGIEALG	1	1
P63673	SQKLTEEAADKLTEVIKNFKK	1	1
P65161	ESLAVGATECKDRSRMTIVVS	1	1
P65232	AHPELAEQVAKELDVHVTSQD	1	1
P65277	ITAATVVLPFKIDDHSAWRLL	1	0
P65573	DVLARLEVDAKEIIGRYPDRR	1	1
P65880	LFDEHGEYLSKTGREFGVTTG	1	0
P66056	SAEPHKTKVAKVTWDQVREIA	1	1
P66902	NFDDCLELARKMAADFPTISL	1	1
P69440	DGYPRSVEQAKALHEMLERRG	1	1
P71703	KFVAAFDVDAKKVGFDLSDAI	1	1
P71724	QAVAAAEVLGKRQPLLIVSSD	1	1
P71973	AIIDDASTFFKQQEERGVVVR	1	0
P77899	TFGTETEDPVKIEKAIGEVFD	1	1
P96382	DGKLGAFVEVKNSTIGTGTKV	1	1
P96825	WAEITDLSGAKIAGFKL____	1	1
Q10504	DHFFGRDPRTKALVENMSDQD	1	1
Q10530	RARIVKEQADKILAKLGGDDS	1	1
Q10682	VVAVGFTFDPKTHIARVITTG	1	1
Q50685	DTVVPRASMPKFLSTARGLAA	1	0
Q7D8W0	GAGELVSAAGKALRDWDALMV	1	1
A0QNF6	ATLHTNRGDIKIALFGNHAPK	0	0
A0QNF6	DEFHPELQFDKPYLLAMANAG	0	0
A0QNF6	KIALFGNHAPKTVNNFVGLAQ	0	0
A0QNF6	NGSQFFITVGKTPHLNRRHTI	0	0
A0QNF6	NNFVGLAQGTKDYSTENASGG	0	0
A0QNF6	TGTGRGGPGYKFEDEFHPELQ	0	0
A0QP32	AGTFQKLNPEKQPNSYLALSD	0	0
A0QP32	AHLVEAGTFQKLNPEKQPNSY	0	0
A0QP32	DKLGDDGFFVKALHSIGAPLE	0	0
A0QP32	EGDPQHLIDWKGNDWTPESGE	0	0
A0QP32	FVGEKLPTGLKDEFDALKHRL	0	0
A0QP32	GDDIAWMRFGKDGRLYATNPE	0	0

A0QP32	GKNADESKLPKVFFVNWFRRG	0	0
A0QP32	GSEQTAAAEGKVGTVRRDPMA	0	0
A0QP32	GWLAEHMLILKLISPENKAYF	0	0
A0QP32	GYGGNALLGKKCYSLRIASAM	0	0
A0QP32	IAAAFPSACGKTNLAMLQPTI	0	0
A0QP32	INVGKNADESKLPKVFFVNWF	0	0
A0QP32	ISAILFGGRRKTTVPLITEAR	0	0
A0QP32	KDVPWPCNDTKYITHFPETRE	0	0
A0QP32	LILKLISPENKAYFIAAAFPS	0	0
A0QP32	LKWAVERIEHKADGKSTPIGI	0	0
A0QP32	MTRMGRAALDKLGDDGFFVKA	0	0
A0QP32	PADVDEALAVKPEEWRAELPL	0	0
A0QP32	PGFGENSRVLKWAVERIEHKA	0	0
A0QP32	SGYGGNALLGKKCYSLRIASA	0	0
A0QP32	TGLKDEFDALKHRLSEEG___	0	0
A0QP32	TNWSSNPNAMKTIAAGNTVFT	0	0
A0QP32	VERIEHKADGKSTPIGIVPTA	0	0
A0QP32	AGNTVFTNVAKTDDGDVWWEG	0	0
A0QP32	CMGPLDAEDPKLGVEITDSEY	0	0
A0QP32	GNDWTPESGEKAAHPNSRYCT	0	0
A0QP32	IEEWFEFVGEKLPTGLKDEFD	0	0
A0QP32	SIGAPLEPGQKDVPWPCNDTK	0	0
A0QP32	TIPGLDTAPTKHQGLLAWVQE	0	0
A0QP90	AGVPFYLRTGKRMSQRFSEIV	0	0
A0QP90	DEAIRLEMTAKEPGSGGRLRP	0	0
A0QP90	HVNRESVRDEKRKVLEALEPL	0	0
A0QP90	NRESVRDEKRKVLEALEPLTA	0	0
A0QP90	QIFRIDHYLGKESVQQLLVTR	0	0
A0QP90	QRFSEIVVQFKPVPLPMFPGI	0	0
A0QP90	VTESSRVVLEKPIGRDLASAQ	0	0
A0QP90	FGGTGDLAARKLLPALYLRDR	0	1
A0QP90	HSRTETFVAVKAHIRNWRWAG	0	0
A0QPN2	APDVAWDSQAKTAVSSRSLNL	0	0
A0QPN2	ARAGGKFSAHKDATSTGAGLN	0	0
A0QPN2	ARARRSAEGAKARARAASKAK	0	0
A0QPN2	ASRMRDLGLAKNEEGPEPQDF	0	0
A0QPN2	DATFQAAFPLKGKPPNVYGFP	0	0
A0QPN2	DPTTGKNGIVKTLGTARAGGK	0	0
A0QPN2	EAPFTRNDTQKLRGVSNRKPD	0	0
A0QPN2	GAKARARAASKAKGLGTNLSL	0	0
A0QPN2	GLGTNLSLPPKLLPSRESGRG	0	0
A0QPN2	GRGYPGVFEGKEFDPEAPFTR	0	0
A0QPN2	IRVRPDESEKKLHHTLFGGPP	0	0
A0QPN2	KARARAASKAKGLGTNLSLPP	0	0
A0QPN2	KTLGTARAGGKFSAHKDATST	0	0
A0QPN2	LPVDTDPTTGKNGIVKTLGTA	0	0
A0QPN2	PPNVYGFPLNKARAKDEFDAI	0	0
A0QPN2	RTDVTVRRDGKTFLQSFGRGY	0	0

A0QPN2	SNRRVEVQRNKGLGEMDADDF	0	0
A0QPN2	TFQAAFPLKGKPPNVYGFPLN	0	0
A0QPN2	TQKLRGVSNRKPDLHGTEVRI	0	0
A0QPN2	VIRVRPDESEKKLHHTLFGGP	0	0
A0QPN2	YVTMPPLFVVKAGDERIYCQD	0	0
A0QPN2	CEGDSALGTIKAARDATFQAA	0	0
A0QPN2	GGGSHLTAAIKGLSEALAERA	0	0
A0QPN2	SERDAAVAQLKASSNRRVEVQ	0	0
A0QPN2	YGFPLNKARAKDEFDAIERIL	0	0
A0QQ65	ADEVADAHVDKIIVTQNKSNS	0	0
A0QQ65	DDFFRPLINGKSILPTNGNNF	0	0
A0QQ65	EDMPEVSPDGKTYKLKLRQNM	0	0
A0QQ65	FNQDFTSFALK__________	0	0
A0QQ65	FTLYPGPDMDKAKQLIAEANP	0	0
A0QQ65	HVDKIIVTQNKSNSAQVTDIE	0	0
A0QQ65	IVGANDYAEGKADTITGIQTN	0	0
A0QQ65	LTQLGFNATLKVIAGDVYWTT	0	0
A0QQ65	LYPGPDMDKAKQLIAEANPAD	0	0
A0QQ65	MERNPQFQTVKDAGADEVADA	0	0
A0QQ65	_____MIAGRKCLKIASVAAV	0	0
A0QQ65	__MIAGRKCLKIASVAAVAVL	0	0
A0QQ65	PEVSPDGKTYKLKLRQNMKYA	0	0
A0QQ65	SAQVTDIEQNKTDFMHDPPDA	0	0
A0QQ65	TYKLKLRQNMKYADGTPIKAS	0	0
A0QQ65	VSPDGKTYKLKLRQNMKYADG	0	0
A0QQ65	YNTYVPLLTYKHAKGEEGAEV	0	0
A0QQ65	YVPLLTYKHAKGEEGAEVAPG	0	0
A0QQ65	DFTYAIQRLFKADSGGSVFFG	0	0
A0QQ65	LLTQQLTDETKKGYADLDRAY	0	1
A0QQ65	LTQQLTDETKKGYADLDRAYM	0	1
A0QQ65	PPDADRLPEVKARFGDRFRLE	0	0
A0QQ65	SRVAIPELDAKMNELLTQQLT	0	0
A0QQ65	TVWTDDEPDRKRIGEYYHDVL	0	0
A0QQH7	AEKIEAALEFKNQVLVKIYNR	0	0
A0QQH7	ALEFKNQVLVKIYNRKALEPA	0	0
A0QQH7	DKVVERWAGSKKIGTTGRGIG	0	0
A0QQH7	KVVERWAGSKKIGTTGRGIGP	0	0
A0QQH7	LLMPYHVAIDKVVERWAGSKK	0	0
A0QQH7	NQVLVKIYNRKALEPAEVLEN	0	0
A0QQH7	PVCVGYTVDGKRVDEMPMTQS	0	0
A0QQH7	TRITTVLGILKAYTTRVGSGP	0	0
A0QQH7	VDPGVLLTELKGLEDRGVDTS	0	0
A0QQH7	VNGITDYFLTKLDVLSSLETV	0	0
A0QQH7	DVLDEQVLAEKIEAALEFKNQ	0	1
A0QQH7	ENLLEQAEGFKHRIADARLLL	0	1
A0QQH7	GAREFEDLPAKARDYVLRLEE	0	0
A0QQU5	AATEVELKERKHRIEDAVRNA	0	0
A0QQU5	AKLAGGVAVIKAGAATEVELK	0	0

A0QQU5	EDPYILLVSSKVSTVKDLLPL	0	0
A0QQU5	ENSDSDYDREKLQERLAKLAG	0	0
A0QQU5	FLTTEAVVADKPEKAAAPAGD	0	0
A0QQU5	IRGTFKSVAVKAPGFGDRRKA	0	0
A0QQU5	IVRVALSAPLKQIALNGGLEP	0	0
A0QQU5	KAGAATEVELKERKHRIEDAV	0	0
A0QQU5	KEIELEDPYEKIGAELVKEVA	0	0
A0QQU5	LELTEGMRFDKGYISGYFVTD	0	0
A0QQU5	LKRGIEKAVEKVTETLLKSAK	0	0
A0QQU5	LLAAGVADPVKVTRSALQNAA	0	0
A0QQU5	________MAKTIAYDEEARR	0	0
A0QQU5	NPLGLKRGIEKAVEKVTETLL	0	0
A0QQU5	PLLEKVIQSGKPLLIIAEDVE	0	0
A0QQU5	TEAVVADKPEKAAAPAGDPTG	0	0
A0QQU5	TITNDGVSIAKEIELEDPYEK	0	0
A0QQU5	TVKDLLPLLEKVIQSGKPLLI	0	0
A0QQU5	VKAPGFGDRRKAMLQDMAILT	0	0
A0QQU5	ADAVKVTLGPKGRNVVLEKKW	0	1
A0QQU5	ADVSLLGKARKVVVTKDETTI	0	1
A0QQU5	DREKLQERLAKLAGGVAVIKA	0	1
A0QQU5	GAELVKEVAKKTDDVAGDGTT	0	0
A0QQU5	GEALSTLVVNKIRGTFKSVAV	0	0
A0QQU5	GGLEPGVVAEKVSNLPAGHGL	0	0
A0QQU5	GPKGRNVVLEKKWGAPTITND	0	0
A0QQU5	HRIEDAVRNAKAAVEEGIVAG	0	0
A0QQU5	IGAELVKEVAKKTDDVAGDGT	0	0
A0QQU5	IGDLIAEAMDKVGNEGVITVE	0	0
A0QQU5	KVTETLLKSAKEVETKEQIAA	0	0
A0QQU5	LETADVSLLGKARKVVVTKDE	0	0
A0QQU5	LGKARKVVVTKDETTIVEGAG	0	0
A0QQU5	LLKSAKEVETKEQIAATAGIS	0	0
A0QQU5	LLVSSKVSTVKDLLPLLEKVI	0	0
A0QQU5	LVVNKIRGTFKSVAVKAPGFG	0	0
A0QQU5	PKGRNVVLEKKWGAPTITNDG	0	0
A0QQU5	PYEKIGAELVKEVAKKTDDVA	0	0
A0QQU5	RGLNSLADAVKVTLGPKGRNV	0	0
A0QS45	APKKKVAGLIKLQIQAGQANP	0	0
A0QS45	DQVREIAETKKADLNANDIDA	0	0
A0QS45	GVQKGSGEPHKTKVAKVTWDQ	0	0
A0QS45	KLLLKAAGVQKGSGEPHKTKV	0	0
A0QS45	KTPPAAKLLLKAAGVQKGSGE	0	0
A0QS45	LNANDIDAAAKIIAGTARSMG	0	0
A0QS45	_____MAPKKKVAGLIKLQIQ	0	0
A0QS45	QHGVNIMEFCKAYNAATESQR	0	0
A0QS45	TFALKTPPAAKLLLKAAGVQK	0	0
A0QS45	WDQVREIAETKKADLNANDID	0	0
A0QS45	YEDRSFTFALKTPPAAKLLLK	0	0
A0QS45	QKGSGEPHKTKVAKVTWDQVR	0	0

A0QS98	EAPVVRVSALKALEGDPKWVK	0	0
A0QS98	EDVERGQVVVKPGTTTPHTEF	0	0
A0QS98	EFEGQVYILSKDEGGRHTPFF	0	0
A0QS98	EIVGIRPETTKTTVTGVEMFR	0	0
A0QS98	GKTTLTAAITKVLHDKFPDLN	0	0
A0QS98	IGTIGHVDHGKTTLTAAITKV	0	0
A0QS98	________MAKAKFERTKPHV	0	0
A0QS98	______MAKAKFERTKPHVNI	0	0
A0QS98	_MAKAKFERTKPHVNIGTIGH	0	0
A0QS98	MPGDNTDISVKLIQPVAMDEG	0	0
A0QS98	NISHVEYQTDKRHYAHVDAPG	0	0
A0QS98	SALKALEGDPKWVKSVEELME	0	0
A0QS98	SIPDPVRETDKPFLMPVEDVF	0	0
A0QS98	TAAITKVLHDKFPDLNESRAF	0	0
A0QS98	TTVTGVEMFRKLLDQGQAGDN	0	0
A0QS98	VDAPGHADYIKNMITGAAQMD	0	0
A0QS98	DNVGLLLRGIKREDVERGQVV	0	0
A0QS98	GRTVGAGRVTKIIK_______	0	0
A0QS98	GVPYILVALNKSDAVDDEELI	0	0
A0QS98	VGAGRVTKIIK__________	0	0
A0QSE0	EDRKSHPLYGKIIRTTKKVKA	0	0
A0QSE0	GKIIRTTKKVKAHDENGEAGI	0	0
A0QSE0	KGPKYTPAAEKPRGRRKTAIG	0	0
A0QSE0	KTIVVELEDRKSHPLYGKIIR	0	0
A0QSE0	LMETRPLSATKRWRLVEILEK	0	0
A0QSE0	LYGKIIRTTKKVKAHDENGEA	0	0
A0QSE0	______MADQKGPKYTPAAEK	0	0
A0QSE0	___MADQKGPKYTPAAEKPRG	0	0
A0QSE0	PAAEKPRGRRKTAIGYVVSDK	0	0
A0QSE0	PLYGKIIRTTKKVKAHDENGE	0	0
A0QSE0	IGYVVSDKMQKTIVVELEDRK	0	1
A0QSE0	KTAIGYVVSDKMQKTIVVELE	0	0
A0QSE0	WRLVEILEKAK__________	0	0
A0QSH8	DTDEVIRNRMKVYREETEPLL	0	0
A0QSH8	GDGTPLGLEAKRYLDAGDLVP	0	0
A0QSH8	LKDMLAARNTKLDAVLEFQVS	0	0
A0QSH8	PLLEYYRDDLKTVNAVGALDE	0	0
A0QSH8	PQISTGDLFRKNIGDGTPLGL	0	0
A0QSH8	PRSVEQAGALKDMLAARNTKL	0	0
A0QSH8	VSEDELLTRLKGRGRADDTDE	0	0
A0QSH8	VVLLGPPGAGKGTQAEKLSEK	0	0
A0QSH8	PGAGKGTQAEKLSEKLGIPQI	0	1
A0QSL6	AQAKKGGTAAKKGQKTRRREK	0	0
A0QSL6	ARKAQEHGVKKVDVFVKGPGS	0	0
A0QSL6	HGVKKVDVFVKGPGSGRETAI	0	0
A0QSL6	KGGTAAKKGQKTRRREKKNVP	0	0
A0QSL6	KGQKTRRREKKNVPHGAAHIK	0	0
A0QSL6	KKGQKTRRREKKNVPHGAAHI	0	0

A0QSL6	_____MAQAKKGGTAAKKGQ	0	0
A0QSL6	____MAQAKKGGTAAKKGQK	0	0
A0QSL6	QAKKGGTAAKKGQKTRRREKK	0	0
A0QSL6	AARKAQEHGVKKVDVFVKGPG	0	1
A0QSL6	AQLAAENAARKAQEHGVKKVD	0	1
A0QSL6	AWASSGHVGFKGSRKSTPFAA	0	0
A0QSL6	KNVPHGAAHIKSTFNNTIVSI	0	0
A0QSL6	PQPHNGCRPPKRRRV_____	0	0
A0QSU4	AGYCDLKEFQKVGLTVGS__	0	0
A0QSU4	GDIHSSGDLAKAIACGADAVV	0	0
A0QSU4	IVPSRRTRSSKDVSTAWQLDA	0	0
A0QSU4	LVGGLRRSMAKAGYCDLKEFQ	0	0
A0QSU4	SMAKAGYCDLKEFQKVGLTVG	0	0
A0QSU4	VASDGEPLNLKTFISELDVPV	0	0
A0QSU4	LIGRHADVEEKIAQVVEVAAK	0	0
A0QUV7	AQAENATRITKREPAVAGDRP	0	0
A0QUV7	GTAAGLTDGLKAAGAAKIYVA	0	0
A0QUV7	GVIIAANADGKEIAGRLAARI	0	0
A0QUV7	IQHRAGMQTSKTIVAVNKDEE	0	0
A0QUV7	LGEPSAVVVGKPGTAAGLTDG	0	0
A0QUV7	MLVEHADGALKKVSAELLTAA	0	0
A0QUV7	PGQFQVGQTGKTVSPQLYIAL	0	0
A0QUV7	QTSKTIVAVNKDEEAPIFEIA	0	0
A0QUV7	ADLGIVGDLFKVSPQLTEAVK	0	0
A0QUV7	KVSPQLTEAVKARKG_____	0	0
A0QUV7	LVEHADGALKKVSAELLTAAR	0	0
A0QUV7	PQLTEAVKARKG________	0	0
A0QUV7	SGLLVDVIEVKEGGVGVHSIF	0	0
A0QUV7	TDGLKAAGAAKIYVAESDDVE	0	0
A0QUX7	KQLNKLINVIKIVEQEEDNSV	0	0
A0QUX7	LTIEATGTPEKLEALLRVLEP	0	0
A0QUX7	NSVSRELALIKVRADATTRGQ	0	0
A0QUX7	THTLSVLVEDKPGVLARVSSL	0	0
A0QUX7	IIEAVNLFRAKVVDVSTESLT	0	0
A0QUX7	PLEQITKQLNKLINVIKIVEQ	0	0
A0QUX7	SRGPRGIGAAK_________	0	0
A0QUX7	VEDSPLEQITKQLNKLINVIK	0	0
A0QUY3	DAFARAQDRRKHLTLVHKTNV	0	0
A0QUY3	DRRKHLTLVHKTNVLAFAGSL	0	0
A0QUY3	____MSSNLKLAVIAGDGIG	0	0
A0QUY3	SEHLATRGDEKLSTSAVGERI	0	0
A0QUY3	SGVLERGMLLKLRFALDHHVN	0	0
A0QUY3	VLDAVLPGVEKTEYDLGARRY	0	0
A0QUY9	APDETTFEFLKGRPHAPKGAD	0	0
A0QUY9	ATQTLSLKPFKTMAVNVDGEL	0	0
A0QUY9	AVAAATAVRGKLASPADLPAA	0	0
A0QUY9	DGELPPGVSAKDIILAVIAKI	0	0
A0QUY9	DHNVPTIDIDKPIADPVSRTQ	0	0

A0QUY9	EFLKGRPHAPKGADWDAAVAA	0	0
A0QUY9	HVMATQTLSLKPFKTMAVNVD	0	0
A0QUY9	_MDQPTQTSVKPRTLAEKVWD	0	0
A0QUY9	SNRNFEGRQGKGGRTHLVSPA	0	0
A0QUY9	TSVKPRTLAEKVWDDHVVARG	0	0
A0QUY9	AKDIILAVIAKIGTGGGQGHV	0	1
A0QUY9	VARGEGEGTAKEPDLIYIDLH	0	1
A0QUY9	VDEGARQAAEKALAYMDLRPG	0	1
A0QUZ0	EAYEAARPAWKPRTLPA____	0	0
A0QUZ0	GLDDIGLTLRKLDQIEAYEAA	0	0
A0QUZ0	TDQIIPAVYLKRVTRTGFEDG	0	0
A0QUZ0	FADIFRGNAGKAGLLAAEVAQ	0	1
A0QUZ0	VAQDDVELLWKVIEQNPGLEI	0	1
A0QV10	HIQLGNIVIPKSVNPERIASN	0	0
A0QV10	LIHWPVPENNKFVDTFKAFAH	0	0
A0QV10	PENNKFVDTFKAFAHLRDQGR	0	0
A0QV10	PQQELRDVHAKLGIATEAWSP	0	0
A0QV10	VITGIAEQHGKTPAQVLIRWH	0	0
A0QV10	VPREDIFLVTKLWNSDQGYDA	0	0
A0QVB9	AAKANDIETLKAAKTGDTTVE	0	0
A0QVB9	AVELLRIKGAKDVGKRAERAT	0	0
A0QVB9	DQIVAAAVAAKANDIETLKAA	0	0
A0QVB9	ELNSETDFVAKNAEFQALADQ	0	0
A0QVB9	ELTGAGMLDSKNALVEADGDF	0	0
A0QVB9	EYQAGDADKGKEAAHAVALQI	0	0
A0QVB9	IAEETARNEGKPEQALPKIVE	0	0
A0QVB9	IVEGRVTGFYKDVVLLDQPSV	0	0
A0QVB9	LDQPSVSDNKKTVKALLDEAG	0	0
A0QVB9	LLDQPSVSDNKKTVKALLDEA	0	0
A0QVB9	LRIKGAKDVGKRAERATAEGL	0	0
A0QVB9	LVEYQAGDADKGKEAAHAVAL	0	0
A0QVB9	_MANYTAADVKRLRELTGAGM	0	0
A0QVB9	NEGKPEQALPKIVEGRVTGFY	0	0
A0QVB9	PSVSDNKKTVKALLDEAGVTV	0	0
A0QVB9	FDGTVEAYLHKRAADLPPAVG	0	1
A0QVB9	FDKAVELLRIKGAKDVGKRAE	0	0
A0QVB9	HAVALQIAALKAKYLTREDVP	0	0
A0QVB9	RATAEGLVAAKDGALIELNSE	0	0
A0QVB9	VALQIAALKAKYLTREDVPED	0	0
A0QVB9	VEQAIADLSAKIGEKLELRRA	0	0
A0QVM0	AADTLKNVLLKTREPGGEWEL	0	0
A0QVM0	ADFAANPFLVKGYVGPKALQD	0	0
A0QVM0	DPADAEVPSHKLLIRAGYVRA	0	0
A0QVM0	EIGHIFQLGRKYTDAFSADVL	0	0
A0QVM0	FDDRQASPGVKFKDAELLGMP	0	0
A0QVM0	FPVILYQIQTKYRDEARPRAG	0	0
A0QVM0	FSADVLGEDGKPLRLTMGSYG	0	0
A0QVM0	GILRGREFVMKDSYSFDVDDD	0	0

A0QVM0	GRTVTAADTLKNVLLKTREPG	0	0
A0QVM0	GVPGDREVDEKRLGAALEPAE	0	0
A0QVM0	LTVKGEYSSYKDFPVILYQIQ	0	0
A0QVM0	PFLVKGYVGPKALQDNGVRYL	0	0
A0QVM0	AQPTEGLPEAKVYDTPDTPTI	0	0
A0QVM0	PFDVHVVVANKDAGARAGAAE	0	1
A0QVM0	WLPLGLRVLRKIENVVRSEMN	0	0
A0QW25	________MAKLDFDALNSTI	0	0
A0QW25	SEHGMAARGYKDVRANTVPAF	0	0
A0QW25	VALHRPAEFNKSHIPAFLAGE	0	0
A0QW25	AATFLKQQEDKGVVVRGLYDV	0	1
A0QW25	PRISVENLIAKLP________	0	0
A0QWG8	ADYTNHIDKWKFTVPFVCGAT	0	0
A0QWG8	DVSNATTHMRKIGGEIRRLTS	0	0
A0QWG8	EGVFVGSGIFKSGNPEQRAAA	0	0
A0QWG8	IDAVTIPVMAKARIGHFVEAQ	0	0
A0QWG8	ITEGAAMIRSKGEAGTGDVSN	0	0
A0QWG8	MSEDELYVAAKELQAPYELVV	0	0
A0QWG8	NPEQRAAAIVKATTFYDDPDV	0	0
A0QWG8	RVKRGMAEMLKGGVIMDVVTP	0	0
A0QWG8	SSNQTGTARVKRGMAEMLKGG	0	0
A0QWG8	TPADYTNHIDKWKFTVPFVCG	0	0
A0QWG8	ELVVEVARAGKLPVTLFTAGG	0	0
A0QWT3	ARHGGGAFSGKDPSKVDRSAA	0	0
A0QWT3	DIELPWEQTNKVDDLKSAI__	0	0
A0QWT3	DTAHETRVEGKADPLDLQGAG	0	0
A0QWT3	DYRLLVNPTGKFVLGGPMGDA	0	0
A0QWT3	GGAFSGKDPSKVDRSAAYAMR	0	0
A0QWT3	GVLDYLRPDGKTQVTIQYDGT	0	0
A0QWT3	ILDIGYDSSTKGFDGASCGVN	0	0
A0QWT3	PMGDAGLTGRKIIVDTYGGWA	0	0
A0QWT3	RLARRLTEVRKNGVLDYLRPD	0	0
A0QWT3	SAAYAMRWVAKNVVAAGLAER	0	0
A0QWT3	SESVTEGHPDKICDAISDSVL	0	0
A0QWT3	VEVQVAYAIGKAAPVGLFVET	0	0
A0QWT3	EGTLTPDIREKVVNTVLADLG	0	1
A0QWT3	________MSKGRLFTSESVT	0	0
A0QWT3	SETVDPAKIEKAIGEVFDLRP	0	0
A0QWT3	VLDALLEQDPKSRVAVETLVT	0	1
A0QWT3	VTTTAYADIPKIVRDRILDIG	0	0
A0QWV9	DLGIIARRLRKDIYDLYGYNA	0	0
A0QWV9	IRRLLSMADRKAKQEGIPDTE	0	0
A0QWV9	LGRLADPPMTKDAVAGRIRRL	0	0
A0QWV9	_____MTAEVKDELSRLVVNS	0	0
A0QWV9	RLLSMADRKAKQEGIPDTESA	0	0
A0QWV9	SVPDHLAAAGKLRVEHRQASL	0	0
A0QWV9	VHVLSASGIRKNTRYVVRVAN	0	0
A0QWW2	AAEGPLKGILKYYDAPIVSSD	0	0

A0QWW2	AKGHLDAGAKKVIISAPATDE	0	0
A0QWW2	AMKAAAEGPLKGILKYYDAPI	0	0
A0QWW2	ASVEDINAAMKAAAEGPLKGI	0	0
A0QWW2	CTTNCLGPLAKVLNDEFGIVK	0	0
A0QWW2	DTIVIGDTKIKALEVKEGPAA	0	0
A0QWW2	ESTGIFTNAAKAKGHLDAGAK	0	0
A0QWW2	GDDTIVIGDTKIKALEVKEGP	0	0
A0QWW2	GDTKIKALEVKEGPAALPWGD	0	0
A0QWW2	GLTKVIDNQAKVVSWYDNEWG	0	0
A0QWW2	IGLVLPELKGKLDGYALRVPI	0	0
A0QWW2	ITIVLGVNDDKYDGSQNIISN	0	0
A0QWW2	KAIGLVLPELKGKLDGYALRV	0	0
A0QWW2	KAKGHLDAGAKKVIISAPATD	0	0
A0QWW2	KVLNDEFGIVKGLMTTIHAYT	0	0
A0QWW2	NIVPTSTGAAKAIGLVLPELK	0	0
A0QWW2	RALATQKAEGKNTDIEIVAVN	0	0
A0QWW2	RLADLVALVGKSL________	0	0
A0QWW2	RNFYRALATQKAEGKNTDIEI	0	0
A0QWW2	TDNATLAHLLKFDSILGRLPQ	0	0
A0QWW2	TGIFTNAAKAKGHLDAGAKKV	0	0
A0QWW2	HSSLYDAGLTKVIDNQAKVVS	0	0
A0QWW2	QDQNLQDGPHKDLRRARAAAL	0	0
A0QWX9	APNTVNTMPEKTLEAVADHGE	0	0
A0QWX9	DAYLEGLEKAKEAGHDLSKIH	0	0
A0QWX9	DVRNACDLLAKEYEASDGVDG	0	0
A0QWX9	EEVFGSDRFAKLKADGARVQR	0	0
A0QWX9	GSDEALALRGKAGVANARLAY	0	0
A0QWX9	KAKEAGHDLSKIHSVASFFVS	0	0
A0QWX9	KIVDRPNLLIKIPATMAGLPA	0	0
A0QWX9	NPSIFQAALSKGTAYDAQVNE	0	0
A0QWX9	VDPRLAHDTDKTILQAIELWK	0	0
A0QWX9	VFGSDRFAKLKADGARVQRPL	0	0
A0QWX9	VMDAYLEGLEKAKEAGHDLSK	0	0
A0QWX9	ATQGQLDAAKK__________	0	0
A0QWX9	DATQGQLDAAKK_________	0	1
A0QWX9	FRVLEDEGVEKFEKSWQELLD	0	0
A0QWX9	LEDEGVEKFEKSWQELLDATQ	0	0
A0QWX9	QRPLWASTGVKNPEYSDTLYV	0	0
A0QWX9	TAASSQETFDKLAAIGIDLPD	0	0
A0QX20	AALLAKKAVEKGLTTKPWVKT	0	0
A0QX20	AGIPLVVLGGKEYGSGSSRDW	0	0
A0QX20	AVAALGGDPNKVNPLAPAELV	0	0
A0QX20	DMLRRHGVVGKFVEFYGKGVA	0	0
A0QX20	EKGLTTKPWVKTNMAPGSQVV	0	0
A0QX20	EQLALVEAYAKAQGMWHDPER	0	0
A0QX20	EWDPASTYVRKAPYFDGMPAE	0	0
A0QX20	FIYDASENYKKAGIPLVVLGG	0	0
A0QX20	FPAGESAASLKLDGTETYDIE	0	0

A0QX20	GSGSSRDWAAKGTVLLGVKAV	0	0
A0QX20	GSQVVTDYYNKAGLWPYLEKL	0	0
A0QX20	HVTATKEDGSKVEFDAVVRID	0	0
A0QX20	IEELNSGKTPKTVHVTATKED	0	0
A0QX20	IEGIEELNSGKTPKTVHVTAT	0	0
A0QX20	ILQYVLRNMLKSSK_______	0	0
A0QX20	KKAVEKGLTTKPWVKTNMAPG	0	0
A0QX20	LRTEDGANITKDHIEAIANWD	0	0
A0QX20	MFTESYADVFKGDDRWRSLPT	0	0
A0QX20	___MSSENTGKSSLNSFGARD	0	0
A0QX20	NFEGRISPDVKMNYLASPPLV	0	0
A0QX20	NKAGLWPYLEKLGYYLGGYGC	0	0
A0QX20	PAEPEPVSDIKGARVLALLGD	0	0
A0QX20	PGTEKLPYSLKVLAENLLRTE	0	0
A0QX20	QYLDANGVERKDYNSLGSRRG	0	0
A0QX20	RWGQGAFDDFKVVPPGTGIVH	0	0
A0QX20	SMLIPRVVGFKLSGEIKPGVT	0	0
A0QX20	STVVPSISGPKRPQDRIELTD	0	0
A0QX20	SVMLGAALLAKKAVEKGLTTK	0	0
A0QX20	TDHISPAGAIKPGTPAAQYLD	0	0
A0QX20	TPKTVHVTATKEDGSKVEFDA	0	0
A0QX20	VMLGAALLAKKAVEKGLTTKP	0	0
A0QX20	VVGFKLSGEIKPGVTATDVVL	0	0
A0QX20	VVGKFVEFYGKGVAEVPLANR	0	0
A0QX20	YRLNAVPGTEKLPYSLKVLAE	0	0
A0QX20	YVLRNMLKSSK__________	0	0
A0QX20	AAKGTVLLGVKAVITESFERI	0	1
A0QX20	AFIYDASENYKKAGIPLVVLG	0	1
A0QX20	NTGPLPEEISKAINDNDLAVT	0	0
A0QX20	PQDRIELTDAKNAFRKDIHNY	0	0
A0QX20	VEQNHPTPETKLDEAVEESFP	0	1
A0QX81	_____MNMMAKFQMSRIDLRN	0	0
A0QX81	DVSGHVITLSKAEDLPAHGEA	0	1
A0QX81	GPGNIYVTAAKRICRSQVGID	0	1
A0QX81	HVVEYDEAALKDVSGHVITLS	0	0
A0QX81	RELANQLATTKHVERVTAALS	0	0
A0QX81	VERVTAALSGKQSAIVLVDDI	0	0
A0QX93	DALRATLDLLKTEAMAGLPPL	0	0
A0QX93	DAVARLDVMTKALGQPLTSAV	0	0
A0QX93	DSETPLSAYRKLAANRPGTFL	0	0
A0QX93	FPAGTLSGAPKVRAMELIEEV	0	0
A0QX93	RAMELIEEVEKTRRGLYGGVV	0	0
A0QX93	TMEEYTEIVDKLVGDIEAGEA	0	0
A0QX93	GPYEYTEAANKARAVLNAIAA	0	1
A0QX93	GSSPEALVTVKDGRATTHPIA	0	1
A0QX93	LLEKELLADEKERAEHLMLVD	0	0
A0QX93	STVTGELAEDKTALDAVTACF	0	0
A0QX93	TEHRVVPVIRKVLADSETPLS	0	0

A0QXX7	ALIVGGHTFGKTHGAGDASLV	0	0
A0QXX7	APIEQQGLGWKSSYGTGKGPD	0	0
A0QXX7	ARRLLWPIKQKYGNKISWADL	0	0
A0QXX7	ASAASYRNTDKRGGANGARVR	0	0
A0QXX7	DFNASASGGKKVSLADLIVLA	0	0
A0QXX7	DKARRLLWPIKQKYGNKISWA	0	0
A0QXX7	DSGLSTGQLIKTAWASAASYR	0	0
A0QXX7	EDFDYAEEFAKLDVNALKADL	0	0
A0QXX7	EFAKLDVNALKADLTALMTQS	0	0
A0QXX7	EGVDRKTGQLKWTATANDLVF	0	0
A0QXX7	ENPAIPSPKPKSGAPLRNQDW	0	0
A0QXX7	ESENPAIPSPKPKSGAPLRNQ	0	0
A0QXX7	FVNDFVKAWVKVMNNDRFDLK	0	0
A0QXX7	GEEDEWLGTDKRYGGGEQRQL	0	0
A0QXX7	GFRNYVRPGEKVQLEKMLLER	0	0
A0QXX7	GNVALESMGFKTFGFGFGRED	0	0
A0QXX7	ILYGYEWELTKSPAGAWQFTA	0	0
A0QXX7	ISMRVDPIYGKITRRWLDHPE	0	0
A0QXX7	KVMNNDRFDLK__________	0	0
A0QXX7	LEVVWTNTPTKWDNSFLEILY	0	0
A0QXX7	LGWKSSYGTGKGPDTITSGLE	0	0
A0QXX7	LVDDQDIAALKSTVLDSGLST	0	0
A0QXX7	LWPIKQKYGNKISWADLLVFT	0	0
A0QXX7	NGARVRLEPQKNWDVNEPAEL	0	0
A0QXX7	QDFNASASGGKKVSLADLIVL	0	0
A0QXX7	RALDVNHGGTKHGVFTDRPGA	0	0
A0QXX7	SPAGAWQFTAKDGAGAGTIPD	0	0
A0QXX7	TENVYEGVDRKTGQLKWTATA	0	0
A0QXX7	VNLLDMGTEWKTSETTENVYE	0	0
A0QXX7	VRPGEKVQLEKMLLERAYFLG	0	0
A0QXX7	AGSAAIEKAAKDGGYNVTVPF	0	0
A0QXX7	HPEELSEAFAKAWYKLLHRDM	0	0
A0QXX7	IVLAGSAAIEKAAKDGGYNVT	0	0
A0QXX7	LSEAFAKAWYKLLHRDMGPIS	0	0
A0QXX7	NGERFVNDFVKAWVKVMNNDR	0	1
A0QXX7	SDSRPPNPDTKTASTSESENP	0	0
A0QYD4	SIVNVLPESAKEGSVEAAIKA	0	0
A0QYD4	AKEGSVEAAIKAALSDWTAGQ	0	1
A0QYD4	VNVPAPRWEAKDPRTYTLADL	0	1
A0QYS9	FKITRLTGKTKIVAAEGAFHG	0	0
A0QYS9	GHPLVDKVRGKGLLQGIVLTV	0	0
A0QYS9	GIVPDVVTMAKGLGGGLPIGA	0	0
A0QYS9	GSLALTGQPSKQAPFEPLPGN	0	0
A0QYS9	IEELGHPLVDKVRGKGLLQGI	0	0
A0QYS9	SGTEANEVAFKITRLTGKTKI	0	0
A0QYS9	VAFKITRLTGKTKIVAAEGAF	0	0
A0QYS9	VCTAAGLAVLKTLAAEDLVAR	0	0
A0QYS9	QGIVLTVPSAKAVETAARDAG	0	1

A0QYS9	YLVAAREITSKHGALLVLDEV	0	0
A0QZ54	AIKSVLETGQKGLRIQHLLDS	0	0
A0QZ54	AILRPGRLDVKIKIERPDAEA	0	0
A0QZ54	AKAVANSLAKKMAEVRGDDAR	0	0
A0QZ54	ANGDKEVMYFKDFNSGAMIQN	0	0
A0QZ54	ARNAKLMDTLKEARQQLLALR	0	0
A0QZ54	CSPNIEVKELKQGQTVRLNEA	0	0
A0QZ54	DTVDVFTSGRKMRLTCSPNIE	0	0
A0QZ54	EFNGDRALCIKAMIEKVVDRM	0	0
A0QZ54	ELPFLHKDLYKEYSLRPPKGV	0	0
A0QZ54	EVRGDDAREAKSYFLNIKGPE	0	0
A0QZ54	FLEVTYANGDKEVMYFKDFNS	0	0
A0QZ54	IAKAVANSLAKKMAEVRGDDA	0	0
A0QZ54	LNIKGPELLNKFVGETERHIR	0	0
A0QZ54	LYKEYSLRPPKGVLLYGPPGC	0	0
A0QZ54	NPDDWARISGKKGERIVYIRT	0	0
A0QZ54	PDDWARISGKKGERIVYIRTL	0	0
A0QZ54	PPGCGKTLIAKAVANSLAKKM	0	0
A0QZ54	RALCIKAMIEKVVDRMYAEID	0	0
A0QZ54	RDAVELPFLHKDLYKEYSLRP	0	0
A0QZ54	REAKSYFLNIKGPELLNKFVG	0	0
A0QZ54	RIDSLAARNAKLMDTLKEARQ	0	0
A0QZ54	RLTCSPNIEVKELKQGQTVRL	0	0
A0QZ54	VLLYGPPGCGKTLIAKAVANS	0	0
A0QZ54	AMIQNVVDRAKKYAIKSVLET	0	1
A0QZ54	DAEAAQDIFSKYLTEDLPVHA	0	1
A0QZ54	IRLIFQRAREKASEGTPVIVF	0	1
A0QZ54	KAGYAFERIPKAEVEDLVLEE	0	0
A0QZ54	LRPGRLDVKIKIERPDAEAAQ	0	0
A0QZ54	MIQNVVDRAKKYAIKSVLETG	0	0
A0QZ54	RPGDSLLVDTKAGYAFERIPK	0	0
A0QZ54	TDYFDDSRPRKLRPGDSLLVD	0	0
A0QZ54	VVDRAKKYAIKSVLETGQKGL	0	0
A0QZ54	VWLAEPLVAAKDLPDEPTDYF	0	0
A0QZA1	AGWQERYPDVKVHRRVERAQP	0	0
A0QZA1	QPAYWLLEEAKQAQLVVVGSH	0	0
A0QZA1	HGLTVQTEIRKESVARTLIDA	0	0
A0QZA1	NARHAIEQAQKVVADCLGETH	0	0
A0QZE3	DWFNEEGSRFKPSMMGSAVFT	0	0
A0QZE3	GLVDRTWAANKYELNVVGIQG	0	0
A0QZE3	IAEAEIRAGVKVEHRKAHVWP	0	0
A0QZE3	IRAGVKVEHRKAHVWPGHHYQ	0	0
A0QZE3	TRAGHDSTNMKEIVPSVMLFV	0	0
A0R066	AAVITPVSHVKHHDGEFTIAD	0	1
A0R066	ESILANPGFGKYYTDHMVSID	0	1
A0R066	HYGQEIFEGLKAYRWADGSIV	0	1
A0R066	LIASPAGAYFKGGIKPVSVWL	0	1
A0R066	PAGAYFKGGIKPVSVWLSHEY	0	1

A0R066	SLRQLIAVDEKWVPPAGGEES	0	0
A0R066	TDAGFAVEERKIDVDEWQKKA	0	0
A0R066	RASPGGTGAAKFGGNYAASLL	0	1
A0R066	RKIDVDEWQKKAGAGEITEVF	0	0
A0R079	AFTNPTVNSYKRLVPGYEAPI	0	0
A0R079	AMLMAGIDGIKKKIEPLQPVD	0	0
A0R079	EDLIETWISYKRENEIMPIQI	0	0
A0R079	ESDGSANRGYKVRPKGGYFPV	0	0
A0R079	GTAGQAEINYKFNTLLAAADD	0	0
A0R079	IIKNTAWQAGKTVTFMPKPLF	0	0
A0R079	IPITGNNPKAKRLEFRCPDSS	0	0
A0R079	KKIEPLQPVDKDLYELPPDEA	0	0
A0R079	LMAGIDGIKKKIEPLQPVDKD	0	0
A0R079	_______MAEKTSDDIFKLIK	0	1
A0R079	MAEKTSDDIFKLIKDENVEYV	0	1
A0R079	MLMAGIDGIKKKIEPLQPVDK	0	1
A0R079	QAGKTVTFMPKPLFGDNGSGM	0	0
A0R079	SANRGYKVRPKGGYFPVAPYD	0	0
A0R079	SGMHAHQSLWKDGQPLFHDES	0	0
A0R079	TARIDPFRAAKTLNMNFFVHD	0	0
A0R079	VRIPITGNNPKAKRLEFRCPD	0	0
A0R079	YIFDSVSFDSKINGTFYEVDS	0	0
A0R079	APTSLSAVIDKLEEDHEYLTE	0	1
A0R079	DDVLLFKYIIKNTAWQAGKTV	0	1
A0R079	LAAADDVLLFKYIIKNTAWQA	0	0
A0R079	YSRDPRNVARKAENYLASTGI	0	0
A0R083	ADGVRASFMPKPFAEHPGSAM	0	0
A0R083	AVTNQWVNSYKRLVHGGEAPT	0	0
A0R083	DDPLQLSDVAKSFIAGILEHA	0	0
A0R083	DNVMTFRYLVKEVALADGVRA	0	0
A0R083	DPSTFQVLPWKTSDGNHYSAR	0	0
A0R083	EHVFDYFLRNKRSEWENYRSH	0	0
A0R083	LVRVPMYTPHKVSSRRVEVRS	0	0
A0R083	LWFTDVLGYLKSVAIAPAELE	0	0
A0R083	______MDRQKEFVLRTLEER	0	0
A0R083	VHPEIEFFLLKPGPNDGTPPE	0	0
A0R083	YRSHVTPYELKNYLSL_____	0	0
A0R083	SRHVLRRQLAKASDLGFTCYV	0	1
A0R0B0	ATTMATVRTFKELLRDKNIGP	0	0
A0R0B0	DMSDQEIWNLKRGGHDYRKVY	0	0
A0R0B0	EGRNATHQMKKLALEDLKNFR	0	0
A0R0B0	ELNREGVAIEKHRLRHPDEPA	0	0
A0R0B0	EVGFNHFFRGKSHPGGGDHVF	0	0
A0R0B0	FEGRNATHQMKKLALEDLKNF	0	0
A0R0B0	FFRGAGWNVIKVVWGREWDVL	0	0
A0R0B0	FGMDSWFPSLKIYNRNGQLYT	0	0
A0R0B0	FNHYLHDRGIKDTSDQHVWAF	0	0
A0R0B0	GFVPSRRTKSKPLALPGSDTY	0	0

A0R0B0	GLYLYRPAPEKRAKSAQILAS	0	0
A0R0B0	HKGQPTVILAKTIKGYTLGQH	0	0
A0R0B0	LGGFVPSRRTKSKPLALPGSD	0	0
A0R0B0	LPGSDTYKALKKGSGSQAVAT	0	0
A0R0B0	LYRPAPEKRAKSAQILASGVA	0	0
A0R0B0	NLKRGGHDYRKVYAAYRAAME	0	1
A0R0B0	PENLDVEALLKGLYLYRPAPE	0	1
A0R0B0	PGSDTYKALKKGSGSQAVATT	0	1
A0R0B0	PLALPGSDTYKALKKGSGSQA	0	1
A0R0B0	QMKKLALEDLKNFRDVTRVPV	0	0
A0R0B0	QPTVILAKTIKGYTLGQHFEG	0	0
A0R0B0	RLDGPVRGNGKIIQELESFFR	0	0
A0R0B0	STPDGDYQTYKANDGAYVRDH	0	0
A0R0B0	SVDSELMLAYKESEVGQILHE	0	0
A0R0B0	VRTFKELLRDKNIGPRIVPII	0	0
A0R0B0	YAAYRAAMEHKGQPTVILAKT	0	0
A0R0B2	AGVISADDAVKLAATRGAEMA	0	0
A0R0B2	AMEKLVAQLTKPVRWDLCTAT	0	0
A0R0B2	ATLLAHEELTKRGHSAAETIV	0	0
A0R0B2	ELKGTPTRAIKSPEDLDGLDQ	0	0
A0R0B2	LAATRGAEMAKACAVEPTGMA	0	0
A0R0B2	PPAGTLVGIAKRELKGTPTRA	0	0
A0R0B2	PVASAADAMEKLVAQLTKPVR	0	0
A0R0B2	TLVGIAKRELKGTPTRAIKSP	0	0
A0R0B2	EDPPAKARVRKLATAGAFHTH	0	0
A0R0B2	LDKLAEDPPAKARVRKLATAG	0	1
A0R0B3	GIEPSEVTPEKSFVDDLDIDS	0	0
A0R0B3	MVEIAVQTEDKYGVKIPDEDL	0	0
A0R0B3	NPEAAAALREKFAADQ_____	0	0
A0R0B4	ADGLRAGQAMKRAMETAGLDP	0	0
A0R0B4	DPQGASRPFDKDRDGFVFGEA	0	0
A0R0B4	GVEHAAVYAPKSALGHSIGAV	0	0
A0R0B4	MMIIETEEHAKARGAKPLARL	0	0
A0R0B4	RAMETAGLDPKDIDHVNAHAT	0	0
A0R0B4	RRMSYVQRMSKFLSKQLWENA	0	0
A0R0B4	SIAPDIESTWKGLLAGESGIR	0	0
A0R0B4	TEEHAKARGAKPLARLMGAGI	0	0
A0R0B4	VAGEPRYGEYKYAINNSFGFG	0	0
A0R0B4	VIGTGLGGGEKIVETYDAMNE	0	0
A0R0B4	YDAMNEGGPRKVSPLAVQMIM	0	0
A0R0B4	YVQRMSKFLSKQLWENAGAPE	0	0
A0R0B5	AAAIGLERQAKAGVCTSISAC	0	0
A0R0B5	ALATDAESTWKKLLDGQSGIR	0	0
A0R0B5	DIAICGGVETKIEAVPIAGFA	0	0
A0R0B5	DPEGACKPFDKDRNGFVFGEA	0	0
A0R0B5	GGHKPAVYAPKGALGHSVGAV	0	0
A0R0B5	GTNVGDVAESKAINNAMGGHK	0	0
A0R0B5	KAINNAMGGHKPAVYAPKGAL	0	0

A0R0B5	LATDAESTWKKLLDGQSGIRT	0	0
A0R0B5	LMVIETEEHAKARGANILGRL	0	0
A0R0B5	MTRAIELAGLKPTDIDHVNAH	0	1
A0R0B5	NSNDDPEGACKPFDKDRNGFV	0	1
A0R0B5	VAGEPRQGDYKYAINNSFGFG	0	1
A0R0B5	GGHNVALAFGKY_________	0	0
A0R0B5	LVFAYDGMRSKGLRAVSPLVV	0	0
A0R0W1	ARIQVADIGFKGQMVDDGNGP	0	0
A0R0W1	EKPAPKTRPAKRPRGEGQWAL	0	0
A0R0W1	FCKLSFAETRKRAQVLVPELE	0	0
A0R0W1	FREVLETEYLKRPLIDGPAPE	0	0
A0R0W1	FRWWGLYTQRKPGYDGTWTGD	0	0
A0R0W1	GKPEYSNLPRKFKTAISGLQD	0	0
A0R0W1	IDEIVRRYIGKPEYSNLPRKF	0	0
A0R0W1	IQVENMPEIWKRLDAVGLQTT	0	0
A0R0W1	KFLIKDWGVEKFREVLETEYL	0	0
A0R0W1	KLIILDVPDDKIDELRAGLDA	0	0
A0R0W1	LMACTGIEFCKLSFAETRKRA	0	0
A0R0W1	__MTSSEAPAKTAEKPAPKTR	0	0
A0R0W1	PAKTAEKPAPKTRPAKRPRGE	0	0
A0R0W1	PEYSNLPRKFKTAISGLQDVV	0	0
A0R0W1	PIDHVGVQKLKNGLNAVGVAP	0	0
A0R0W1	RDYGYRRLRSKARLKFLIKDW	0	0
A0R0W1	RSKARLKFLIKDWGVEKFREV	0	0
A0R0W1	SGFGRKLRQHKVLSSELGDYI	0	0
A0R0W1	SLGLDSGFGRKLRQHKVLSSE	0	0
A0R0W1	SSEAPAKTAEKPAPKTRPAKR	0	0
A0R0W1	TRPIDHVGVQKLKNGLNAVGV	0	0
A0R0W1	YAKNGFESIDKGDLRGRFRWW	0	0
A0R0W1	YRRLRSKARLKFLIKDWGVEK	0	0
A0R0W1	AGRVSGTILTKVADLAEAAGS	0	1
A0R0W1	EPLNANEQAKKDDNPLNVRAR	0	1
A0R0W1	REPLNANEQAKKDDNPLNVRA	0	1
A0R0W1	SDRIRFTPYQKLIILDVPDDK	0	0
A0R0W1	VRARIENIYAKNGFESIDKGD	0	0
A0R0W1	YIERVVRNFVKQREDGERFAQ	0	0
A0R0W4	AFGLKIRKRPKAEIAERVDNL	0	0
A0R0W4	DNVAFGLKIRKRPKAEIAERV	0	0
A0R0W4	GFVFQHYAAFKHLSVRDNVAF	0	0
A0R0W4	SVRDNVAFGLKIRKRPKAEIA	0	0
A0R0W4	TALLGPSGSGKSTLLRAIAGL	0	0
A0R0W4	LDEPFGALDAKVRDDLRTWLR	0	1
A0R0W4	TSAIVVRGANKHYGDFAALDN	0	0
A0R1B5	DALKYGSRAAKARFAVYIGPL	0	0
A0R1B5	LGVSAAAASFKEGNLIDGLIS	0	0
A0R1B5	MDLVVLDDALKYGSRAAKARF	0	1
A0R1V9	FQWRAQHEDLKTLPFPMVSDL	0	0
A0R1V9	GKWRIIFFWPKDFTFVCPTEI	0	0

A0R1V9	QSDELCACNWKKGDPTINAGE	0	0
A0R1V9	SDELCACNWKKGDPTINAGEL	0	0
A0R1V9	TLPFPMVSDLKRELTAACGVL	0	0
A0R1V9	TRVTSKDYEGKWRIIFFWPKD	0	0
A0R1V9	VCPTEIAAFGKLNEDFEDRDA	0	0
A0R1V9	LNEDFEDRDAKVLGVSVDNEF	0	0
A0R1Y7	AAAVIVMNKAKAEELGLTWLA	0	0
A0R1Y7	DGAAAVIVMNKAKAEELGLTW	0	0
A0R1Y7	EVVPVSIPQRKGDPIEFAEDE	0	0
A0R1Y7	GAIAIRAALEKANVPASMVEY	0	0
A0R1Y7	HLLPKSREGYKYGDATLVDHL	0	0
A0R1Y7	IVAGARTPVGKLMGSLKDFSG	0	0
A0R1Y7	NTTAESLAGLKPAFRKDGTIT	0	0
A0R1Y7	PWDVAALSINKMCLSGIDAIA	0	0
A0R1Y7	QDEYAAQSHQKAAAAWKDGVF	0	0
A0R1Y7	SLAGLKPAFRKDGTITAGSAS	0	0
A0R1Y7	SMSQAPHLLPKSREGYKYGDA	0	0
A0R1Y7	STKELGVDPAKVNVNGGAIAI	0	0
A0R1Y7	TPVGKLMGSLKDFSGTDLGAI	0	0
A0R1Y7	AFAAVALASTKELGVDPAKVN	0	1
A0R1Y7	ALTEQRNDVDKFTRAEQDEYA	0	1
A0R1Y7	LQSQPANAIKKAITREGITVD	0	1
A0R1Y7	TLQSQPANAIKKAITREGITV	0	1
A0R218	ADKPAKEAQTKAAEAPEAGES	0	0
A0R218	AIDLLISQLRKTKTNYEFLVQ	0	0
A0R218	AKTERAEKADKPAKEAQTKAA	0	0
A0R218	AVDVNPSGTRKDELLLSPDEF	0	0
A0R218	DGQDGKQDQGKQDRSERDQDQ	0	0
A0R218	DLLISQLRKTKTNYEFLVQVS	0	0
A0R218	DRGEKDGQDGKQDQGKQDRSE	0	0
A0R218	EAKAEAPAEAKTERAEKADKP	0	0
A0R218	EAKTEAQPEAKAEAPAEAKTE	0	0
A0R218	EEVTDMQRSVKGEVIASTFDR	0	0
A0R218	EFKGTGNAELKLDRKIAERRV	0	0
A0R218	ERAEKADKPAKEAQTKAAEAP	0	0
A0R218	GDSGGQNQRQKFNPLVRLDTV	0	0
A0R218	GEKTEAQPEAKTEAQPEAKAE	0	0
A0R218	GVDSTALYPPKRFLGAARNIE	0	0
A0R218	IGVEGASGLRKSELIAAIRAH	0	0
A0R218	KGEAGEKAGEKTEAQPEAKTE	0	0
A0R218	LSPDEFAIVHKLRRVLSGLDS	0	0
A0R218	MVLPELRALAKEIGVEGASGL	0	0
A0R218	PAEAKTERAEKADKPAKEAQT	0	0
A0R218	PVAEKGEAGEKAGEKTEAQPE	0	0
A0R218	RVIDLIMPIGKGQRALIVSPP	0	0
A0R218	SDTGDLSGAVKSNTAPRARSA	0	0
A0R218	TGDTVIFEEFKGTGNAELKLD	0	0
A0R218	TGNAELKLDRKIAERRVFPAV	0	0

A0R218	TNYEFLVQVSKTAPGSMDVD_	0	0
A0R218	TRQAGAPVAEKGEAGEKAGEK	0	0
A0R218	ALIVSPPKAGKTTILQDIANA	0	0
A0R218	EDAKKRPEFGKLTPLYPNERL	0	0
A0R218	ERAKRLVEQGKDVVVLLDSIT	0	0
A0R218	GQRALIVSPPKAGKTTILQDI	0	0
A0R218	GRQDRADRGEKDGQDGKQDQG	0	0
A0R218	QAAELAIERAKRLVEQGKDVV	0	0
A0R218	TVNGGSVEDAKKRPEFGKLTP	0	0
A0R218	VNGGSVEDAKKRPEFGKLTPL	0	1
A0R220	ASAASIAGLLKSVEDGWVKRG	0	0
A0R220	GNAGNITAYWKGYSEYHRDGV	0	0
A0R220	ITCAVLIPQGKIAMGKLAQAV	0	0
A0R220	KLAQAVMHGAKIIQVDGNFDD	0	0
A0R220	LEGGTPLIHAKRISELTGCTV	0	0
A0R220	LIPQGKIAMGKLAQAVMHGAK	0	0
A0R220	LLKSVEDGWVKRGSTVVCTVT	0	0
A0R220	______MSAAKAAVHQPWPGL	0	0
A0R220	NGLKDPDTALKGMPQVTPVPV	0	0
A0R220	SELTGCTVHLKVEGLNPTGSF	0	0
A0R220	SVNPYRIEGQKTAAFEIVDAL	0	0
A0R220	VEGLNPTGSFKDRGMTVAVTE	0	0
A0R220	VVCTVTGNGLKDPDTALKGMP	0	0
A0R220	AAPLVTGAPVKDPETIATAIR	0	0
A0R2G5	GFETTRARLRKRGAVAVYGID	0	0
A0R2G5	GGGKEVIKVGKRCLLGANSGL	0	0
A0R2G5	RGAVAVYGIDKFPRMVDYVTP	0	0
A0R2G5	RLHLLSHRLTKPHEANLDGIF	0	0
A0R2G5	SIMGTLSGGGKEVIKVGKRCL	0	0
A0R2G5	TLSGGGKEVIKVGKRCLLGAN	0	0
A0R2G5	VPADLAALTGKDEDRDVEVVA	0	0
A0R2G5	VRTTIADLNDKPADTHDVWLR	0	0
A0R2G5	EAGLYVTGGTKVTTADGQVTK	0	1
A0R2G5	KVTTADGQVTKAIELSGASNL	0	0
A0R2V7	ATEEYAPAVDKGCPMVLGGPL	0	0
A0R2V7	DAVAAAAREFKPLVLVAGYSA	0	0
A0R2V7	EAPELANFGAKHINDLSEADW	0	0
A0R2V7	GGPLSHVMAAKAVALAEARQP	0	0
A0R2V7	HAHVTTTTTHKSLRGPRGGMV	0	0
A0R2V7	KELADQRDSLKLIASENYASP	0	0
A0R2V7	LANTQPEGTSKAKYKLADGTA	0	0
A0R2V7	LLTMGTWFSDKYAEGTIGHRF	0	0
A0R2V7	MAHFAGLVAGKVFTGDEDPVP	0	0
A0R2V7	NTQPEGTSKAKYKLADGTAER	0	0
A0R2V7	QPEGTSKAKYKLADGTAERVH	0	0
A0R2V7	SAYPRRVNFAKMREIADEVGA	0	0
A0R2V7	SEADWETLRNKLGNQRLLGMS	0	0
A0R2V7	THGFRPNISGKMFHQRSYGTN	0	0

A0R2V7	VEPRVAAATRKELADQRDSLK	0	0
A0R2W6	FDGDGRITSMKAYWSPADIKQ	0	0
A0R2W6	HDAAALESFVKRYLDTVAGGS	0	0
A0R2W6	MKAYWSPADIKQL________	0	0
A0R2W6	KNMTAATTDIKTELLSFRPGG	0	1
A0R342	ACLNAIEYLKKWGYTGEQAYL	0	0
A0R342	APCHMLSGPIKVEGAEPGDLL	0	0
A0R342	DIRPGGKGPQKVDRGQVAVTS	0	0
A0R342	EGWGYSGIFAKVNGGGFLTDY	0	0
A0R342	FLTDYYPDAYKAIWDFHGQQC	0	0
A0R342	GFIDMHVDLIKGGMETYGVTT	0	0
A0R342	IFDFDIRPGGKGPQKVDRGQV	0	0
A0R342	KPGSEFRIECKEWTDGQIGNN	0	0
A0R342	NACLNAIEYLKKWGYTGEQAY	0	0
A0R342	PEVVFSVDHSKSMRDQAVPGH	0	0
A0R342	PRENGGNHDIKNFTRGSRIFY	0	0
A0R342	GTAPSPDLLAKWNERERALIA	0	0
A0R3D2	GASRQARRDGKVPAVLYGHGT	0	1
A0R3D2	________MAKTANIPNKLTA	0	1
A0R3D2	_MAKTANIPNKLTANVRTRTG	0	1
A0R3D2	ADLLVVQRGEKVTVEVTVLVE	0	0
A0R3D2	IEGTEQLALTKALDVHPIRRN	0	0
A0R3D6	AALRRAVPATKGNTKRLASLG	0	0
A0R3D6	ADVEDFLDAGKPFGLRPEAIL	0	0
A0R3D6	DFLRDTLLWAKLIDKYKGTMT	0	0
A0R3D6	DTLLWAKLIDKYKGTMTAAPN	0	0
A0R3D6	EGNIVVCGRVKDVIIMAGRNI	0	0
A0R3D6	IPMYFGAELVKVTPMDFLRDT	0	0
A0R3D6	LLWAKLIDKYKGTMTAAPNFA	0	0
A0R3D6	MFVGAKYDVEKDVMVSWLPCF	0	0
A0R3D6	____MSRFTEKMYRNARTVTT	0	0
A0R3D6	QLTSGSTGSPKAVQITHRNIH	0	0
A0R3D6	RAVPATKGNTKRLASLGPLLN	0	0
A0R3D6	SNAEAMFVGAKYDVEKDVMVS	0	0
A0R3D6	TMNVIGMIELKAVIISEPFLV	0	0
A0R3D6	FLVATPVLEEKGILVLKVADL	0	1
A0R3D6	PGSIPKTPSGKLRRANSVSLV	0	0
A0R3D6	VLEEKGILVLKVADLLAADPI	0	0
A0R4C9	DVKLLDGGRKKWELDARPLSA	0	0
A0R4C9	DVRSPDEFSGKILAPAHLPQE	0	0
A0R4C9	DWAESNLKAPKTVFVEVDEDT	0	0
A0R4C9	GTFKSDEELAKLYAEAGLDGE	0	0
A0R4C9	IPGAINVPWSKAANEDGTFKS	0	0
A0R4C9	LQELLGHKNVKNYDGSWTEYG	0	0
A0R4C9	LYAEAGLDGEKETIAYCRIGE	0	0
A0R4C9	QDVKLLDGGRKKWELDARPLS	0	0
A0R4C9	VSTDWAESNLKAPKTVFVEVD	0	0
A0R4C9	WFAAYAYWYFKLYGHQDVKLL	0	0

A0R4C9	WFVLQELLGHKNVKNYDGSWT	0	0
A0R4C9	YDTGHIEGAVKLDWKTDLQDP	0	0
A0R4C9	YFKLYGHQDVKLLDGGRKKWE	0	0
A0R4C9	ELDARPLSAEKVERPQTSYTA	0	1
A0R4C9	HIEGAVKLDWKTDLQDPIRRD	0	1
A0R4C9	RDEVIAAIGTKNLVDVRSPDE	0	0
A0R4C9	SKAANEDGTFKSDEELAKLYA	0	0
A0R4C9	VERPQTSYTAKEPDNSIRAFR	0	0
A0R4D0	__MPAGVDLEKETVITGRVVD	0	0
A0R4Z5	AFAPVVLAWIKDLKADPEKVN	0	0
A0R4Z5	AVLLASEQAVKEHNLKPRARI	0	0
A0R4Z5	GPIPATEYALKKTGLSIDDID	0	0
A0R4Z5	IDAVRTPVGKKNGSLAQVHPI	0	0
A0R4Z5	IIDAVRTPVGKKNGSLAQVHP	0	0
A0R4Z5	LGHPLGATGAKLFATMLNTLE	0	0
A0R4Z5	PIPATEYALKKTGLSIDDIDT	0	0
A0R4Z5	PVVLAWIKDLKADPEKVNPNG	0	0
A0R4Z5	SEQAVKEHNLKPRARIHHISA	0	0
A0R4Z5	VPISSAMIVGKELGFSTPTAE	0	0
A0R4Z5	WIKDLKADPEKVNPNGGAIAL	0	0
A0R518	EVDVRDYDALKAVVDSGVEQL	0	0
A0R518	GIGNGGATLDKTSEADWDDMI	0	0
A0R518	IILTSSVGGLKAYPHTGHYIA	0	0
A0R518	___MAGRVEGKVAFITGAARG	0	0
A0R518	MIGVNLSGVWKTVKAAVPHLI	0	1
A0R518	PMTVDAGSMLK__________	0	1
A0R518	TPLFMNEGTMKLFRPDLENPG	0	0
A0R518	VNLSGVWKTVKAAVPHLISGG	0	0
A0R518	YPHTGHYIAAKHGVVGLMRTF	0	0
A0R566	GSIEYAVTVLKVPLIVVLGHD	0	0
A0R566	RVTPSILLGRKAGLSRVDEFE	0	0
A0R566	VLGHDSCGAVKATLSALDEGE	0	0
A0R566	SNPVAAWKALKDGNARFVAGQ	0	0
A0R5E1	KVEFEVGQSPKGPQATGVRTI	0	0
A0R5E1	____MPQGTVKWFNAEKGFGF	0	0
A0R5E1	QGTVKWFNAEKGFGFIAPEDG	0	0
A0R5M8	DSLLLGTDLVKDTGRLVRAYD	0	0
A0R5M8	DVRAGLTAAPKSLPPKWFYDA	0	0
A0R5M8	GEEMLTEVSCKFRPENVVAEL	0	0
A0R5M8	LTAAPKSLPPKWFYDAVGSDL	0	0
A0R5M8	LVELGSGTSEKTRMLLDAMRD	0	0
A0R5M8	VCGDFEEHLGKIPHVGRRLVV	0	0
A0R5R5	AEILEIFAAGKEPLKFTAYDG	0	0
A0R5R5	AVEEVGLPTAKVWGLYMAGSR	0	0
A0R5R5	EIFAAGKEPLKFTAYDGSSAG	0	0
A0R5R5	HNYPAYFNFLKSKLRTGGLLL	0	0
A0R5R5	IVRSIGIEHLKPIAPPPQEAL	0	0
A0R5R5	LRALAERMEFKRPPARVLANI	0	0

A0R5R5	______MTTFKERETSTADRK	0	0
A0R5R5	MVRYAARHGVKALGVTLSREQ	0	0
A0R5R5	PEDATMGLDLKTPRGTTYLAT	0	0
A0R5R5	QDNKYRLVFEKLRLKPGDRLL	0	0
A0R5R5	RRIMEGLRHSKTRDAEAIHHH	0	0
A0R5R5	VLAVKLDDQGKDGGLPLRPWW	0	0
A0R5R5	YPAYFNFLKSKLRTGGLLLNH	0	0
A0R5R5	YRLVFEKLRLKPGDRLLDVGC	0	0
A0R5R5	DATLEEAQDNKYRLVFEKLRL	0	0
A0R5R5	KERETSTADRKLTLAEILEIF	0	0
A0R5R5	VVQLHQVLAVKLDDQGKDGGL	0	0
A0R5R7	AFPYRKDPEDKNIRIAPTFPS	0	0
A0R5R7	ASADNIAWYLKHAGKKSIGPD	0	0
A0R5R7	EQLVASDPTIKGIWCVPVYSN	0	0
A0R5R7	ESKIASWTDPKGGYFVSLDVW	0	0
A0R5R7	GTAKRTVALAKDAGIAVTEAG	0	0
A0R5R7	HAGKKSIGPDKVNQLRHLRFF	0	0
A0R5R7	IAWYLKHAGKKSIGPDKVNQL	0	0
A0R5R7	KQLKLDLTRGKPSPEQLDLSN	0	0
A0R5R7	LAATEALLGDK__________	0	0
A0R5R7	MQRHRELIAPKFALVAEILED	0	0
A0R5R7	NIAWYLKHAGKKSIGPDKVNQ	0	0
A0R5R7	NRPLVFASTSKITFAGAGVSF	0	0
A0R5R7	NYAELQAKQLKLDLTRGKPSP	0	0
A0R5R7	PRPWSAEPTVKFLCPAPGYDR	0	0
A0R5R7	QQRNYAELQAKQLKLDLTRGK	0	0
A0R5R7	RRLVQMPTAAKDFRLMWDNAY	0	1
A0R5R7	TEAGSAFPYRKDPEDKNIRIA	0	1
A0R5R7	VSLDVWPGTAKRTVALAKDAG	0	1
A0R635	ARAKEAFGIQKRQRQMDRCRQ	0	0
A0R635	DDPESIFTTGKAAYIEDYAQR	0	0
A0R635	DSVKHTVSGPKGDLDVMIIAL	0	0
A0R635	FQIQRRCPHLKADLSKFGVVE	0	0
A0R635	GYNEYLYTFFKCLTDERIAYA	0	0
A0R635	HNFFETTDSVKHTVSGPKGDL	0	0
A0R635	NLENGRCLTTKGHELRCQKL_	0	0
A0R635	PMVYDMPARAKEAFGIQKRQR	0	0
A0R635	PVPDLRRELEKLGFHNFFETT	0	0
A0R635	RCPHLKADLSKFGVVEGNTLT	0	0
A0R635	TTKGHELRCQKL_________	0	0
A0R635	DPEHLRRYVNKDAVVLLPDYP	0	1
A0R635	QRMAPVLAAEKARWAPSAGEP	0	0
A0R635	RVVREPIPDEKFRYGFEIPAA	0	0
A0R635	VDYLYVSHLHKDHFDPEHLRR	0	0
A0R647	FDGADLPQLAKHFYAQALEER	0	0
A0R647	LGEQFMQWFLKEQVEEVAAMT	0	0
A0R652	AIDDQFGSFDKFQAQFTAAAN	0	0
A0R652	GDHAAIFLNEKNLAFHLGGHI	0	0

A0R652	GGHINHSIWWKNLSPNGGDKP	0	0
A0R652	HAFYLQYKNVKADYVKAFWNV	0	0
A0R652	MWEHAFYLQYKNVKADYVKAF	0	0
A0R652	QYKNVKADYVKAFWNVVNWDD	0	0
A0R652	YVKGVNDAIAKLEEARANGDH	0	0
A0R652	SGQINELHHSKHHATYVKGVN	0	1
A0R652	VQNRFAAATSKTSGLIFG___	0	0
A0R6E3	AEAANGIGVRKDLIEAVEAEE	0	1
A0R6E3	QRLYVARLRAKLTTAEAANGI	0	1
A0R6Q0	ATPVDHPIPGKVVVGIKTADV	0	0
A0R6Q0	EIEATGGAIIKGPYDDAHERR	0	0
A0R6Q0	GNGLVFYSPLKR_________	0	0
A0R6Q0	KFYETLGFSLKFRDGAHFAAL	0	0
A0R6Q0	LSTENLDESIKFYETLGFSLK	0	0
A0R6Q0	____MSDHEVKMVVLSTENLD	0	1
A0R6Q0	PIPGKVVVGIKTADVDAAAKE	0	1
A0R7F9	GRRRLAYEIAKHAEGIYAVID	0	0
A0R7F9	VLRTKVLRTDKH_________	0	0
A0R7F9	AEGIYAVIDVKAEPATVSELD	0	0
A0R7F9	LNLNESVLRTKVLRTDKH___	0	0
A0R7F9	SLETFLNVIRKDGGTVDKVDI	0	0
A0R7G6	AGVPIVGDDIKSQVGATITHR	0	0
A0R7G6	AIFASENNTIKIADVPPTDVI	0	0
A0R7G6	ASAYLMKSPPKQLADDVARAE	0	0
A0R7G6	FIASDPVWAKKFEDAGVPIVG	0	0
A0R7G6	GATITHRVMAKLFEDRGVTLD	0	0
A0R7G6	MLERSRLESKKVSKTQAVTSN	0	0
A0R7G6	NMLERSRLESKKVSKTQAVTS	0	0
A0R7G6	NTTVPGLMHVKFGPYHVRDVN	0	0
A0R7G6	PIEAASAYLMKSPPKQLADDV	0	0
A0R7G6	QRGPTLDGIGKYYADTIEVSD	0	0
A0R7G6	RSRLESKKVSKTQAVTSNLSG	0	0
A0R7G6	SDHVAWLDDRKWAYVRLEGRA	0	0
A0R7G6	SGALAGKVEDKNVHIGPSDHV	0	0
A0R7G6	TSNLSGALAGKVEDKNVHIGP	0	0
A0R7G6	VFIASDPVWAKKFEDAGVPIV	0	0
A0R7G6	VSDAEPVDVVKVLKEAEVDVL	0	0
A0R7G6	AEPVDVVKVLKEAEVDVLVSY	0	1
A0R7G6	FVAAFDVDAKKVGFDLSEAIF	0	0
A0R7G6	IDAVRAAKIAKDRGIGGPIEA	0	1
A4ZHR8	ADIVITATGNKDIITLDHMKA	0	0
A4ZHR8	ADLSLADFGRKEIRLAEHEMP	0	0
A4ZHR8	DMAALERSGAKKINIKPQVDQ	0	0
A4ZHR8	DSVTKSKFDNKYGTRHSLIDG	0	0
A4ZHR8	EPKGVSVFAWKGETLEEYWWA	0	0
A4ZHR8	ERSGAKKINIKPQVDQWIFDD	0	0
A4ZHR8	EYDNEVYRLAKHLDEKVARIH	0	0
A4ZHR8	GVLRERFEQDKTKWTKIAESV	0	0

A4ZHR8	IITLDHMKAMKNQAILGNIGH	0	0
A4ZHR8	INVNDSVTKSKFDNKYGTRHS	0	0
A4ZHR8	LRERFEQDKTKWTKIAESVKG	0	0
A4ZHR8	MAALERSGAKKINIKPQVDQW	0	0
A4ZHR8	MMVLRGAQYEKAGVVPPAEDD	0	0
A4ZHR8	______MTELKADVRNGIDYK	0	0
A4ZHR8	NKDIITLDHMKAMKNQAILGN	0	0
A4ZHR8	NRGTDVLIGGKKVLICGYGDV	0	0
A4ZHR8	PAEDDDPAEWKVFLGVLRERF	0	0
A4ZHR8	PAINVNDSVTKSKFDNKYGTR	0	0
A4ZHR8	QVDQWIFDDGKSIIVLSEGRL	0	0
A4ZHR8	RFEQDKTKWTKIAESVKGVTE	0	0
A4ZHR8	RGTDVLIGGKKVLICGYGDVG	0	0
A4ZHR8	RREYHDVQPLKGARISGSLHM	0	0
A4ZHR8	TKWTKIAESVKGVTEETTTGV	0	0
A4ZHR8	VGPNGTPEEPKGVSVFAWKGE	0	0
A4ZHR8	VLICGYGDVGKGCAESLAGQG	0	0
A4ZHR8	VYRLAKHLDEKVARIHVEALG	0	0
A4ZHR8	YIGVDVEGPYKPEHYRY____	0	0
A4ZHR8	HVEALGGTLTKLSKDQAEYIG	0	0
A4ZHR8	KADVRNGIDYKVADLSLADFG	0	0
A4ZHR8	QVIAQIELWTKNDEYDNEVYR	0	0
EG10025	AAAPAPAAGVKEVNVPDIGGD	0	0
EG10025	ADAAPAQAEEKKEAAPAAAPA	0	0
EG10025	APFAGVVKELKVNVGDKVKTG	0	0
EG10025	DAAPAQAEEKKEAAPAAAPAA	0	0
EG10025	DVQAYVKEAIKRAEAAPAATG	0	0
EG10025	EEAAKRKLDVKITPVVFIMKA	0	0
EG10025	EGAAPAAAPAKQEAAAPAPAA	0	0
EG10025	ELKVNVGDKVKTGSLIMIFEV	0	0
EG10025	ELSRELMTISKKARDGKLTAG	0	0
EG10025	EQSLITVEGDKASMEVPAPFA	0	0
EG10025	EQSLITVEGDKASMEVPSPQA	0	0
EG10025	EVPAPFAGTVKEIKVNVGDKV	0	0
EG10025	EVPAPFAGVVKELKVNVGDKV	0	0
EG10025	EVPSPQAGIVKEIKVSVGDKT	0	0
EG10025	FRKQQNEEAAKRKLDVKITPV	0	0
EG10025	GEAGAAAPAAKQEAAPAAAPA	0	0
EG10025	GGIPGMLPWPKVDFSKFGEIE	0	0
EG10025	IEEVELGRIQKISGANLSRNW	0	0
EG10025	ILREDVQAYVKEAIKRAEAAP	0	0
EG10025	KQQNEEAAKRKLDVKITPVVF	0	0
EG10025	KSAMEPVWNGKEFVPRLMLPI	0	0
EG10025	LAREFGVNLAKVKGTGRKGRI	0	0
EG10025	LSEDGQRLTLKKYINIGVAVD	0	0
EG10025	LSRELMTISKKARDGKLTAGE	0	0
EG10025	LVVPVFKDVNKKGIIELSREL	0	0
EG10025	_____MAIEIKVPDIGADEVE	0	0

EG10025	MLPWPKVDFSKFGEIEEVELG	0	0
EG10025	MTISKKARDGKLTAGEMQGGC	0	0
EG10025	NLAKVKGTGRKGRILREDVQA	0	1
EG10025	PAAAPAAAAAKDVNVPDIGSD	0	1
EG10025	QEAAAPAPAAKAEAPAAAPAA	0	1
EG10025	REFGVNLAKVKGTGRKGRILR	0	1
EG10025	SEDGQRLTLKKYINIGVAVDT	0	1
EG10025	TPNGLVVPVFKDVNKKGIIEL	0	1
EG10025	VKEIKVNVGDKVSTGSLIMVF	0	0
EG10025	VKEIKVSVGDKTQTGALIMIF	0	0
EG10025	VKELKVNVGDKVKTGSLIMIF	0	0
EG10025	VKITPVVFIMKAVAAALEQMP	0	0
EG10025	VMIPHVTHFDKTDITELEAFR	0	0
EG10025	VVPVFKDVNKKGIIELSRELM	0	0
EG10025	AAAPAAKAEGKSEFAENDAYV	0	1
EG10025	AEAPAAAPAAKAEGKSEFAEN	0	0
EG10025	APEVAILGVSKSAMEPVWNGK	0	0
EG10025	DEVEITEILVKVGDKVEAEQS	0	0
EG10025	DEVEVTEILVKVGDKVEAEQS	0	0
EG10025	DEVEVTEVMVKVGDKVAAEQS	0	1
EG10025	ITEILVKVGDKVEAEQSLITV	0	1
EG10025	SPQAGIVKEIKVSVGDKTQTG	0	1
EG10025	TDITELEAFRKQQNEEAAKRK	0	0
EG10025	VTEILVKVGDKVEAEQSLITV	0	0
EG10025	VTEVMVKVGDKVAAEQSLITV	0	0
EG10207	ATVGSCAQIGKNVHLSGGVGI	0	0
EG10207	DGQWVTHQWLKKAVLLSFRIN	0	0
EG10207	ESRYFDKVPMKFADYDEARFQ	0	0
EG10207	GQWVTHQWLKKAVLLSFRIND	0	0
EG10207	IEGAESRYFDKVPMKFADYDE	0	0
EG10207	LDSGALRVAEKIDGQWVTHQW	0	0
EG10207	SVVVSGNLPSKDGKYSLYCAV	0	0
EG10207	VSGNLPSKDGKYSLYCAVIVK	0	0
EG10207	CAVIVKKVDAKTRGKVGINEL	0	1
EG10207	KYSLYCAVIVKKVDAKTRGKV	0	1
EG10207	VKKVDAKTRGKVGINELLRTI	0	0
EG10207	YSLYCAVIVKKVDAKTRGKVG	0	0
EG10251	MMLPRSGLGHKHGIVLGNLVG	0	0
EG10251	_________MKKIDVKILDPR	0	1
EG10251	________MKKIDVKILDPRV	0	1
EG10251	____MKKIDVKILDPRVGKEF	0	1
EG10367	AGIALNDNFVKLVSWYDNETG	0	0
EG10367	AKKVVMTGPSKDNTPMFVKGA	0	0
EG10367	ARKHITAGAKKVVMTGPSKDN	0	0
EG10367	ATYEQIKAAVKAAAEGEMKGV	0	0
EG10367	AVKAAAEGEMKGVLGYTEDDV	0	0
EG10367	CTTNCLAPLAKVINDNFGIIE	0	0
EG10367	GEVCTSVFDAKAGIALNDNFV	0	0

EG10367	GLFLTDETARKHITAGAKKVV	0	0
EG10367	HGRFDGTVEVKDGHLIVNGKK	0	0
EG10367	IGRIVFRAAQKRSDIEIVAIN	0	0
EG10367	KDGHLIVNGKKIRVTAERDPA	0	0
EG10367	KVLDLIAHISK__________	0	0
EG10367	LDADYMAYMLKYDSTHGRFDG	0	0
EG10367	LEKAATYEQIKAAVKAAAEGE	0	0
EG10367	_______MTIKVGINGFGRIG	0	0
EG10367	NIIPSSTGAAKAVGKVLPELN	0	0
EG10367	PMFVKGANFDKYAGQDIVSNA	0	0
EG10367	PSKDNTPMFVKGANFDKYAGQ	0	0
EG10367	TARKHITAGAKKVVMTGPSKD	0	0
EG10367	TQKTVDGPSHKDWRGGRGASQ	0	0
EG10367	TTVHATTATQKTVDGPSHKDW	0	0
EG10367	VGKVLPELNGKLTGMAFRVPT	0	0
EG10367	WYDNETGYSNKVLDLIAHISK	0	0
EG10367	SSTGAAKAVGKVLPELNGKLT	0	0
EG10367	VKDGHLIVNGKKIRVTAERDP	0	0
EG10367	VTAERDPANLKWDEVGVDVVA	0	1
EG10407	DFAEFDADAAKKHLRPVARQP	0	0
EG10407	DLVKLLGERCKTLKEMAQSCR	0	0
EG10407	DQMLEEGTAYKCYCSKERLEA	0	0
EG10407	EGTAYKCYCSKERLEALREEQ	0	0
EG10407	EWDEGPYYQTKRFDRYNAVID	0	0
EG10407	FAEFDADAAKKHLRPVARQPL	0	0
EG10407	HVSMINGDDGKKLSKRHGAVS	0	0
EG10407	INGDDGKKLSKRHGAVSVMQY	0	0
EG10407	KLLGERCKTLKEMAQSCRYFY	0	0
EG10407	LEALREEQMAKGEKPRYDGRC	0	0
EG10407	LREEQMAKGEKPRYDGRCRHS	0	0
EG10407	NNTPRQINILKALKAPVPVYA	0	0
EG10407	PRQINILKALKAPVPVYAHVS	0	0
EG10407	RNGPQLADLVKLLGERCKTLK	0	0
EG10407	SKSASAFNTDKLLWLNHHYIN	0	0
EG10407	TADELEVGMGKVGMPLRVAVT	0	0
EG10407	VSMINGDDGKKLSKRHGAVSV	0	0
EG10407	ALDVTVHAIGKTRSIERINKA	0	1
EG10407	ARQPLEVVRDKLAAITDWTAE	0	1
EG10407	GKTRSIERINKALDFIAEREN	0	0
EG10407	IKYFTLNAVSKSASAFNTDKL	0	0
EG10407	_________MKIKTRFAPSPT	0	0
EG10407	_______MKIKTRFAPSPTGY	0	0
EG10410	ADYEERKAKIKADAEEAARKI	0	0
EG10410	AEAQPDREIEKRGPAIAQAFD	0	0
EG10410	AEFFFNTDRKKRLEDNLPRLQ	0	0
EG10410	AIAQAFDAEGKPSKAAEGWAR	0	0
EG10410	ALVYTMKGDQKYFPVYANDGK	0	0
EG10410	ANKRVSNILAKSDEVLSDRVN	0	0

EG10410	ASTLKEPEEIKLAMQVVVLRD	0	0
EG10410	ATSLAKLPIPKLMRWGASDVH	0	0
EG10410	DAAAALAAANKRVSNILAKSD	0	0
EG10410	DAEFFFNTDRKKRLEDNLPRL	0	0
EG10410	FDKVMVMVDDKELRINRLTML	0	0
EG10410	FQQQLGTLRDKTDRIQALAGW	0	0
EG10410	GEWLLYRAHVKGESTEALLPN	0	0
EG10410	GKVIADYEERKAKIKADAEEA	0	0
EG10410	IKADAEEAARKIGGNADLSES	0	0
EG10410	LPNMVATSLAKLPIPKLMRWG	0	0
EG10410	LREPVDAFFDKVMVMVDDKEL	0	0
EG10410	LSDRVNASTLKEPEEIKLAMQ	0	0
EG10410	LVEWPVVLTAKFEEKFLAVPA	0	0
EG10410	NFIFVANIESKDPQQIISGNE	0	0
EG10410	NHATRAGLLSKCDLMTNMVFE	0	0
EG10410	PVACALAIADKMDTLAGIFGI	0	0
EG10410	PVVLTAKFEEKFLAVPAEALV	0	0
EG10410	QAFDAEGKPSKAAEGWARGCG	0	0
EG10410	QYPEILRERGKVIADYEERKA	0	0
EG10410	TEEAVRLYGDKLTNANVVDDV	0	0
EG10410	TRPADFDARMKAVSHFRTLDA	0	0
EG10410	VDQAERLTTDKGEWLLYRAHV	0	0
EG10410	VHTVTLLLGDKVIPATILGIQ	0	0
EG10410	VIADYEERKAKIKADAEEAAR	0	0
EG10410	VPAEALVYTMKGDQKYFPVYA	0	0
EG10410	WFAAPRRLALKVANLAEAQPD	0	0
EG10410	AGIFGIGQHPKGDKDPFALRR	0	0
EG10410	ALGVLRIIVEKNLNLDLQTLT	0	0
EG10410	DPQQIISGNEKVVRPRLADAE	0	0
EG10410	FGIGQHPKGDKDPFALRRAAL	0	0
EG10410	KYFPVYANDGKLLPNFIFVAN	0	0
EG10410	LRINRLTMLEKLRELFLRVAD	0	0
EG10410	_______MSEKTFLVEIGTEE	0	1
EG10410	VEIGTEELPPKALRSLAESFA	0	1
EG10416	AMVEGIELTLKSMLDVVRKFG	0	0
EG10416	APGNVLGIMQKGYTLNGRTIR	0	0
EG10416	_______MSSKEQKTPEGQAP	0	0
EG10416	____MSSKEQKTPEGQAPEEI	0	0
EG10416	RAAMVTVAKAKA_________	0	0
EG10416	SLDRALEVADKANPDMSAMVE	0	0
EG10416	TIRAAMVTVAKAKA_______	0	0
EG10416	TLKSMLDVVRKFGVEVIAETN	0	0
EG10416	IEKAHKFALEKFINELLPVID	0	0
EG10416	LRRRTELDIEKAHKFALEKFI	0	1
EG10416	RTELDIEKAHKFALEKFINEL	0	1
EG10489	AVEKAYKGERKISWMEIYTGE	0	0
EG10489	DLIREYRVAIKGPLTTPVGGG	0	0
EG10489	ERLMDGAKLLKCSEFGDAIIE	0	0

EG10489	GELIDGGPWLKVKNPNTGKEI	0	0
EG10489	GIKPCSEEGTKRLVRAAIEYA	0	0
EG10489	GTAPKYAGQDKVNPGSIILSA	0	0
EG10489	GWTEAADLIVKGMEGAINAKT	0	0
EG10489	IGVDVTPAMLKVVDAAVEKAY	0	0
EG10489	IKFLREEMGVKKIRFPEHCGI	0	0
EG10489	ISWMEIYTGEKSTQVYGQDVW	0	0
EG10489	KFLREEMGVKKIRFPEHCGIG	0	0
EG10489	LFEATHGTAPKYAGQDKVNPG	0	0
EG10489	LIDGGPWLKVKNPNTGKEIVI	0	0
EG10489	MLKVVDAAVEKAYKGERKISW	0	0
EG10489	NDRDSVTLVHKGNIMKFTEGA	0	0
EG10489	NIMKFTEGAFKDWGYQLAREE	0	0
EG10489	QGKKITLQNGKLNVPENPIIP	0	0
EG10489	RFPEHCGIGIKPCSEEGTKRL	0	0
EG10489	RYYQGTPSPVKHPELTDMVIF	0	0
EG10489	VKGMEGAINAKTVTYDFERLM	0	0
EG10489	VTLVHKGNIMKFTEGAFKDWG	0	0
EG10489	VVDAAVEKAYKGERKISWMEI	0	0
EG10489	YDFERLMDGAKLLKCSEFGDA	0	0
EG10489	ADSADAEKVIKFLREEMGVKK	0	1
EG10489	ESKVVVPAQGKKITLQNGKLN	0	0
EG10489	EWKADSADAEKVIKFLREEMG	0	0
EG10489	NPNTGKEIVIKDVIADAFLQQ	0	0
EG10489	SEDIYAGIEWKADSADAEKVI	0	0
EG10489	SKVVVPAQGKKITLQNGKLNV	0	1
EG10492	AFQAVDQDALKAKFAVSNVNG	0	1
EG10492	AQQSEVLKGLKVALSKAEGEK	0	1
EG10492	CAKAAQEDILKAYEAYDFHEV	0	0
EG10492	CVSNVAGDGEKRKFA______	0	0
EG10492	DDLYGIIRAAKKGKKTFILHD	0	0
EG10492	DIIKDRQYTAKADSVARRSCQ	0	0
EG10492	DLYGIIRAAKKGKKTFILHDG	0	0
EG10492	DQKGLRAQSLKEIKGVQWIPD	0	0
EG10492	EKFTAAEFRAKCREYAATQVD	0	0
EG10492	ELLKVRGEVNKVIEQARADKK	0	0
EG10492	EMGSFYLDIIKDRQYTAKADS	0	0
EG10492	GIIRAAKKGKKTFILHDGPPY	0	0
EG10492	GIQAWWDLDAKEILGDEADQY	0	0
EG10492	GKIIGNGHLHKGAKPVHWCVD	0	0
EG10492	GLRAQSLKEIKGVQWIPDWGQ	0	0
EG10492	GSIHIGHSVNKILKDIIVKSK	0	0
EG10492	HGFTVDGQGRKMSKSIGNTVS	0	0
EG10492	HGPDDYVIGQKYGLETANPVG	0	0
EG10492	HIGHSVNKILKDIIVKSKGLS	0	0
EG10492	IELKVEQEYGKPGEKFTAAEF	0	0
EG10492	IGNGHLHKGAKPVHWCVDCRS	0	0
EG10492	ILGDEADQYVKVPDTLDVWFD	0	0

EG10492	KILKDIIVKSKGLSGYDSPYV	0	1
EG10492	KVALSKAEGEKCPRCWHYTQD	0	1
EG10492	KVIEQARADKKVGGSLEAAVT	0	0
EG10492	LAEAEVEYYDKTSPSIDVAFQ	0	0
EG10492	LANLNGFDPAKDMVKPEEMVV	0	0
EG10492	______MSDYKSTLNLPETGF	0	0
EG10492	MSSLMISTAMKGKAPYRQVLT	0	0
EG10492	NGFDPAKDMVKPEEMVVLDRW	0	0
EG10492	NKVIEQARADKKVGGSLEAAV	0	0
EG10492	NTVSPQDVMNKLGADILRLWV	0	0
EG10492	PADAQQSEVLKGLKVALSKAE	0	0
EG10492	QAVDQDALKAKFAVSNVNGPI	0	0
EG10492	QEKGALLHVEKMQHSYPCCWR	0	0
EG10492	QHSYPCCWRHKTPIIFRATPQ	0	0
EG10492	RCWHYTQDVGKVAEHAEICGR	0	0
EG10492	RTLELMEEVAKRVEVDGIQAW	0	0
EG10492	SLMISTAMKGKAPYRQVLTHG	0	0
EG10492	SNVAGDGEKRKFA________	0	0
EG10492	TEANIIRALGKIIGNGHLHKG	0	0
EG10492	TGFPMRGDLAKREPGMLARWT	0	0
EG10492	TPQWFVSMDQKGLRAQSLKEI	0	0
EG10492	TVDGQGRKMSKSIGNTVSPQD	0	0
EG10492	VEQEYGKPGEKFTAAEFRAKC	0	0
EG10492	VLDRWAVGCAKAAQEDILKAY	0	0
EG10492	VLKGLKVALSKAEGEKCPRCW	0	0
EG10492	VNKILKDIIVKSKGLSGYDSP	0	0
EG10492	VTDYTILGTVKGAELELLRFT	0	0
EG10492	VWGYLPGEREKYVFTGEWYEG	0	0
EG10492	WDCHGLPIELKVEQEYGKPGE	0	0
EG10492	WGVPMSLFVHKDTEELHPRTL	0	0
EG10492	WSHPYLTMDFKTEANIIRALG	0	0
EG10492	YGIIRAAKKGKKTFILHDGPP	0	0
EG10492	YPTLDGVNVFKANDIVVALLQ	0	0
EG10492	GEMAVSDEILKRAADSYRRIR	0	1
EG10492	NDIVVALLQEKGALLHVEKMQ	0	0
EG10492	QIDGQAVILAKDLVESVMQRI	0	0
EG10492	TLYAEPELSAKLTALGDELRF	0	0
EG10492	YAATQVDGQRKDFIRLGVLGD	0	0
EG10762	AKLYELEMQKKNAEKQAMEDN	0	0
EG10762	AVAELDALEEKLAQLEFRRMF	0	0
EG10762	CQNDRSQHKNKDQAMKQMKAK	0	0
EG10762	EESEGEVAGIKSVTIKISGDY	0	0
EG10762	ELEMQKKNAEKQAMEDNKSDI	0	0
EG10762	EVAGIKSVTIKISGDYAYGWL	0	0
EG10762	KAKLYELEMQKKNAEKQAMED	0	0
EG10762	KDQAMKQMKAKLYELEMQKKN	0	0
EG10762	KNKDQAMKQMKAKLYELEMQK	0	0
EG10762	LRGYLDYDAKKERLEEVNAEL	0	0

EG10762	NAEKQAMEDNKSDIGWGSQIR	0	0
EG10762	RSYVLDDSRIKDLRTGVETRN	0	0
EG10762	SLDQFIEASLKAGL_______	0	0
EG10762	SQHKNKDQAMKQMKAKLYELE	0	0
EG10762	TETGVHRLVRKSPFDSGGRRH	0	0
EG10762	TQCQNDRSQHKNKDQAMKQMK	0	0
EG10762	VLRGYLDYDAKKERLEEVNAE	0	0
EG10762	EAVVDTLDQMKQGLEDVSGLL	0	0
EG10762	NEPERAQALGKERSSLEAVVD	0	0
EG10770	DFAAGANIDGKHYFGINWDRD	0	0
EG10770	DHELNEVKAEKLPQVASPLTF	0	0
EG10770	DLIRNELSSYKQLPLNFYQIQ	0	0
EG10770	GVMRSREFLMKDAYSFHTSQE	0	0
EG10770	IEYKYRRNGEKQLIKTGDIVE	0	0
EG10770	LPLNFYQIQTKFRDEVRPRFG	0	0
EG10770	NIELAEAIAPKEPRAAATQEM	0	0
EG10770	QGIEVLLDDRKERPGVMFADM	0	0
EG10770	QVAILPMNMHKSFRVQELAEK	0	0
EG10770	RNLDNDDIEYKYRRNGEKQLI	0	0
EG10770	RTSQYLLSTLKETPADAEVIS	0	0
EG10770	TWLPTGVRVLKKVENIVREEM	0	0
EG10770	VRGDHELNEVKAEKLPQVASP	0	0
EG10770	WLPTGVRVLKKVENIVREEMN	0	0
EG10770	YRRNGEKQLIKTGDIVEYLVK	0	0
EG10770	ATEEEIRAVVKAGPGSLGPVN	0	0
EG10770	EMTLVDTPNAKTIAELVEQFN	0	0
EG10770	EVGHIFQLGTKYSEALKASVQ	0	0
EG10770	IEKTVKTLLVKAVEGSSFPQV	0	0
EG10770	KTGDIVEYLVKQIKG______	0	0
EG10770	LVEQFNLPIEKTVKTLLVKAV	0	0
EG10770	QFNLPIEKTVKTLLVKAVEGS	0	0
EG10770	QLMLRAGMIRKLASGLYTWLP	0	0
EG10770	SPDGQGRLLIKRGIEVGHIFQ	0	0
EG10770	TYDAMYAAYSKIFSRMGLDFR	0	0
EG10803	AEKIPSINVSKHRLDVQFDNV	0	0
EG10803	ALTEKDKADIKTAALIGVDYL	0	0
EG10803	AMARVCLGAEKIPSINVSKHR	0	0
EG10803	ASEAVNLLRDKGYLMSGDLVI	0	0
EG10803	DAGCDAKIVAKVERAEAVCSQ	0	0
EG10803	DANLGKGEGDKEKVGIDYKGL	0	0
EG10803	GDKEKVGIDYKGLPADVVPGD	0	0
EG10803	GPLSNNKGINKLGGGLSAEAL	0	0
EG10803	IGDPELVGIQKALIRRARQLN	0	0
EG10803	KIRVSTFKEGKVFLNIGDKFL	0	0
EG10803	LLLDDGRVQLKVLEVQGMKVF	0	0
EG10803	LSAEALTEKDKADIKTAALIG	0	0
EG10803	MSAMYAANHLKGVTAIITMTE	0	0
EG10803	NLGKGEGDKEKVGIDYKGLPA	0	0

EG10803	PATDRDNNLEKVIAAGANVVR	0	0
EG10803	QGPKIRVSTFKEGKVFLNIGD	0	0
EG10803	QLKVLEVQGMKVFTEVTVGGP	0	0
EG10803	RLARDAGCDAKIVAKVERAEA	0	0
EG10803	VAILGDLQGPKIRVSTFKEGK	0	0
EG10803	VTVGGPLSNNKGINKLGGGLS	0	0
EG10803	DKFLLDANLGKGEGDKEKVGI	0	0
EG10803	GGLSAEALTEKDKADIKTAAL	0	0
EG10803	__MSRRLRRTKIVTTLGPATD	0	0
EG10803	NFSHGSPEDHKMRADKVREIA	0	0
EG10803	SPEDHKMRADKVREIAAKLGR	0	0
EG10876	EGGVVDLNTLKAANIIGIQIE	0	0
EG10876	GLGKTGGRGHKGQKSRSGGGV	0	0
EG10876	GQMPLYRRLPKFGFTSRKAAI	0	0
EG10876	KTGGRGHKGQKSRSGGGVRRG	0	0
EG10876	LGRGIGSGLGKTGGRGHKGQK	0	0
EG10876	LNTLSPAEGSKKAGKRLGRGI	0	0
EG10876	NIIGIQIEFAKVILAGEVTTP	0	0
EG10876	NTLSPAEGSKKAGKRLGRGIG	0	0
EG10876	PVTVRGLRVTKGARAAIEAAG	0	0
EG10876	RLPKFGFTSRKAAITAEIRLS	0	0
EG10876	SPAEGSKKAGKRLGRGIGSGL	0	0
EG10876	TAEIRLSDLAKVEGGVVDLNT	0	1
EG10903	AGKMEGTFKRKPERSDLSADI	0	0
EG10903	DYGVQLREKQKVRRIYGVLER	0	0
EG10903	EQAPGQHGARKPRLSDYGVQL	0	0
EG10903	KPTWLEVDAGKMEGTFKRKPE	0	0
EG10903	LKSGVRAIDTKCKIEQAPGQH	0	0
EG10903	LSDYGVQLREKQKVRRIYGVL	0	0
EG10903	___MARYLGPKLKLSRREGTD	0	0
EG10903	_MARYLGPKLKLSRREGTDLF	0	0
EG10903	RAEARQLVSHKAIMVNGRVVN	0	0
EG10903	RNYYKEAARLKGNTGENLLAL	0	0
EG10903	SGVRAIDTKCKIEQAPGQHGA	0	0
EG10903	SRREGTDLFLKSGVRAIDTKC	0	0
EG10903	VDAGKMEGTFKRKPERSDLSA	0	0
EG10903	VLERQFRNYYKEAARLKGNTG	0	0
EG10903	DVVSIREKAKKQSRVKAALEL	0	1
EG10903	NDVVSIREKAKKQSRVKAALE	0	1
EG10903	NEHLIVELYSK__________	0	0
EG10903	SPNDVVSIREKAKKQSRVKAA	0	0
EG10905	AVIRSMVMRTKHAVTEASPMV	0	0
EG10905	GRRQLAYPINKLHKAHYVLMN	0	0
EG10905	HAVTEASPMVKAKDERRERRD	0	0
EG10905	QLAYPINKLHKAHYVLMNVEA	0	0
EG10905	VTEASPMVKAKDERRERRDDF	0	0
EG10954	AASFGSFADFKAQFTDAAIKN	0	0
EG10954	FKAQFTDAAIKNFGSGWTWLV	0	0

EG10954	NFGSGWTWLVKNSDGKLAIVS	0	0
EG10954	NLIKGTAFEGKSLEEIIRSSE	0	0
EG10954	SAETIEYHYGKHHQTYVTNLN	0	0
EG10954	SFELPALPYAKDALAPHISAE	0	0
EG10954	WALVNWEFVAKNLAA______	0	0
EG10954	APNAGGEPTGKVAEAIAASFG	0	1
EG10954	WTWLVKNSDGKLAIVSTSNAG	0	0
EG11192	AEVTNSAIELKVLIEQMREQI	0	0
EG11192	AHVKETYNILKKKEAVGRRLD	0	0
EG11192	EELDRLEAHVKETYNILKKKE	0	0
EG11192	ERIRSRLTRGKVECTLRYEPD	0	0
EG11192	FNRESNTLASKSINAEVTNSA	0	0
EG11192	HVKETYNILKKKEAVGRRLDF	0	0
EG11192	KQLVTAANWVKMQSDEGEINP	0	0
EG11192	RLEGVTAEVVKVRSHMPEILQ	0	0
EG11192	SMTAYARREIKGEWGSATWEM	0	0
EG11192	VARETEGQALKALIEQRLEGV	0	0
EG11192	VKETYNILKKKEAVGRRLDFM	0	0
EG11192	LQWQRERLVAKLEDAQVQLEN	0	0
EG11192	SAQGELILNEKLAKQLVTAAN	0	0
EG11223	AALVLDGIQVKRPRCQEAAQQ	0	0
EG11223	ATELADYLVAKGVPFREAHHI	0	0
EG11223	EAVVEAIRQGKPLEDLPLSEL	0	0
EG11223	GALTGMMMTLKGLPLAYNKDM	0	0
EG11223	KLIDKVGQLGKKLHTGRSRND	0	0
EG11223	LAYNKDMQEDKEGLFDALDTW	0	0
EG11223	LIDKVGQLGKKLHTGRSRNDQ	0	0
EG11223	LQSCLDKRAAKGGVSPQQVAQ	0	0
EG11223	NPDALELIRGKCGRVQGALTG	0	0
EG11223	PILSLQSCLDKRAAKGGVSPQ	0	0
EG11223	QDIVGSVAWSKALVTVGVLTA	0	0
EG11223	QVATDLKLWCKDTVSELLTAN	0	0
EG11223	RDESRLQDALKRLDVSPLGCG	0	0
EG11223	RFTQAADQRFKQFNDSLRFDY	0	0
EG11223	SRNDQVATDLKLWCKDTVSEL	0	0
EG11223	TLKGLPLAYNKDMQEDKEGLF	0	0
EG11223	TSGSSLMPQKKNPDALELIRG	0	0
EG11223	VTSGSSLMPQKKNPDALELIR	0	0
EG11223	AEDIHSWVEGKLIDKVGQLGK	0	1
EG11223	LEDLPLSELQKFSQVIDEDVY	0	1
EG11415	ILTAASRDLDKFLWFIESNIE	0	0
EG11415	IQFIDLSLITKQAHWNMRGAN	0	0
EG11415	LDIHNVQDHLKELADRYAIVA	0	0
EG11415	______MSTAKLVKSKATNLL	0	0
EG11415	___MSTAKLVKSKATNLLYTR	0	0
EG11415	_MSTAKLVKSKATNLLYTRND	0	0
EG11415	NDVRKAIGEAKDDDTADILTA	0	0
EG11415	RYAIVANDVRKAIGEAKDDDT	0	0

EG11415	ALGTTQVINSKTPLKSYPLDI	0	1
EG11415	TRNDVSDSEKKATVELLNRQV	0	1
EG11415	YTRNDVSDSEKKATVELLNRQ	0	0
EG11427	ALSMDAVQKAKSGHPGAPMGM	0	0
EG11427	ANAVGMAIAEKTLAAQFNRPG	0	0
EG11427	ANIARGGYVLKDCAGQPELIF	0	0
EG11427	AVEEARAVTDKPSLLMCKTII	0	0
EG11427	CDQVESAVAWKYGVERQDGPT	0	0
EG11427	CSLAGTLKLGKLIAFYDDNGI	0	0
EG11427	DIDGHDAASIKRAVEEARAVT	0	0
EG11427	ELKNFRQLHSKTPGHPEVGYT	0	0
EG11427	EMPSDFDAKAKEFIAKLQANP	0	0
EG11427	FGFTVDNVVAKAKELL_____	0	0
EG11427	FIAKLQANPAKIASRKASQNA	0	0
EG11427	FTVDNVVAKAKELL_______	0	0
EG11427	HEVCSLAGTLKLGKLIAFYDD	0	0
EG11427	IAEVLWRDFLKHNPQNPSWAD	0	0
EG11427	IALTREQLGWKYAPFEIPSEI	0	0
EG11427	IRALSMDAVQKAKSGHPGAPM	0	0
EG11427	KGEMPSDFDAKAKEFIAKLQA	0	0
EG11427	KTIIGFGSPNKAGTHDSHGAP	0	0
EG11427	______MSSRKELANAIRALS	0	0
EG11427	PSEIYAQWDAKEAGQAKESAW	0	0
EG11427	QEAAEFTRRMKGEMPSDFDAK	0	0
EG11427	RNAVRMAALMKQRQVMVYTHD	0	0
EG11427	TGYDLPMEELKNFRQLHSKTP	0	0
EG11427	VEAGIADYWYKYVGLNGAIVG	0	0
EG11427	VTDKPSLLMCKTIIGFGSPNK	0	0
EG11427	AYEKLTAEGVKARVVSMPSTD	0	1
EG11427	DAAYRESVLPKAVTARVAVEA	0	1
EG11427	EVELAVAAYEKLTAEGVKARV	0	1
EG11427	GQAKESAWNEKFAAYAKAYPQ	0	0
EG11427	PSNLTLWSGSKAINEDAAGNY	0	0
EG11427	QANPAKIASRKASQNAIEAFG	0	0
EG11427	QWDAKEAGQAKESAWNEKFAA	0	1
EG11427	VSMPSTDAFDKQDAAYRESVL	0	1
EG11518	EFYLVTVPDGKLSPRLAALKP	0	0
EG11518	GKLSPRLAALKPGDEVQVVSE	0	0
EG11518	GPYLSILQLGKDLDRFKNLVL	0	0
EG11518	LESTIGLPMNKETSHVMLCGN	0	0
EG11518	___MADWVTGKVTKVQNWTDA	0	0
EG11518	MADWVTGKVTKVQNWTDALFS	0	0
EG11518	MQELEKRYEGKLRIQTVVSRE	0	0
EG11518	QLLKETRQMTKHLRRRPGHMT	0	0
EG11518	QMVRDTQQLLKETRQMTKHLR	0	0
EG11518	SYLPLMQELEKRYEGKLRIQT	0	0
EG11518	VLPFTAGQFTKLGLEIDGERV	0	0
EG11615	HIHNLRDKVGKARIRTVRGFG	0	0

EG11615	ILTARDTLTDKIAGLDVGADD	0	0
EG11615	LDVGADDYLVKPFALEELHAR	0	0
EG11615	LEVHIHNLRDKVGKARIRTVR	0	0
EG11615	MGGEELILTPKEYALLSRLML	0	0
EG11615	GLHFLARIRQKKYTLPVLILT	0	0
EG11615	__________MKILIVEDDTLL	0	0
EG12204	FGFITPADGSKDVFVHFSAIQ	0	0
EG12204	HFSAIQGNGFKTLAEGQNVEF	0	0
EG12204	KGQVKWFNESKGFGFITPADG	0	0
EG12204	________MAKIKGQVKWFNE	0	0
EG12204	______MAKIKGQVKWFNESK	0	0
EG12204	NVEFEIQDGQKGPAAVNVTAI	0	1
EG12411	GEKALQEYCAKHLIFRTSWVY	0	0
EG12411	IFRTSWVYAGKGNNFAKTMLR	0	0
EG12411	LVLPDWQVGVKRMLNELFTTT	0	0
EG12411	___MNILLFGKTGQVGWELQR	0	0
EG12411	NFAKTMLRLAKEREELAVIND	0	0
EG12411	RPHNSRLNTEKFQQNFALVLP	0	0
EG12411	TAHAIRVALNKPDVAGLYHLV	0	0
EG12411	TAPLNVYGETKLAGEKALQEY	0	0
EG12411	VNAAAHTAVDKAESEPEFAQL	0	0
EG12411	VYAGKGNNFAKTMLRLAKERE	0	0
EG12411	VYGETKLAGEKALQEYCAKHL	0	0
EG12411	INATSVEAIAKAANEVGAWVI	0	0
EG12411	YAALVFEEARKAGIPLALNKL	0	0
EG12503	EALTLMPVGSKWELTIPQELA	0	0
EG12503	TDRVRVHYTGKLIDGTVFDSS	0	0
EG12503	VAGIADALEGKHPAVPVDVVH	0	0
EG12503	GVKYLEENAKKEGVNSTESGL	0	0
EG12503	RFQAMAAEGVKYLEENAKKEG	0	0
EG12545	AATLSRSQDRKVKISEIIKHT	0	0
EG12545	AGSLRIGGQTKHFLVHETQEE	0	0
EG12545	AKNKKHVFCEKPIALSYEDCV	0	0
EG12545	ARFMNMHDNAKITCVYDPENG	0	0
EG12545	CHTKRNGWENKQERLSWKKMK	0	0
EG12545	DGAIAYGHPGKKTPLWLASLI	0	0
EG12545	DRKVKISEIIKHTSVM_____	0	0
EG12545	EDCVDMVKACKEAGVTFMAGH	0	0
EG12545	ENKQERLSWKKMKEQSGGHLY	0	0
EG12545	EPVIKAAKNKKHVFCEKPIAL	0	0
EG12545	ETQEEDDDRRKGNMTSEMDGA	0	0
EG12545	GAIAYGHPGKKTPLWLASLIR	0	0
EG12545	KEPVIKAAKNKKHVFCEKPIA	0	0
EG12545	KQERLSWKKMKEQSGGHLYHH	0	0
EG12545	LFLHNILCGAKPEEDYIDLLN	0	0
EG12545	LFMTLEFPSGKLATLEWGSAF	0	0
EG12545	LHKEPVIKAAKNKKHVFCEKP	0	0
EG12545	LSYEDCVDMVKACKEAGVTFM	0	0

EG12545	MSSLDALVSSKLVDCVIVATP	0	0
EG12545	NFFNGVQYARKLIKEGVIGEI	0	0
EG12545	NGVQYARKLIKEGVIGEILSC	0	0
EG12545	PEHYVIINGTKGSIKIDMQET	0	0
EG12545	TLSRSQDRKVKISEIIKHTSV	0	0
EG12545	TPLWLASLIRKETLFLHNILC	0	0
EG12545	VIGEILSCHTKRNGWENKQER	0	0
EG12545	VIINGTKGSIKIDMQETAGSL	0	0
EG12545	WENKQERLSWKKMKEQSGGHL	0	0
EG12545	VIVATPNYLHKEPVIKAAKNK	0	1
EG12669	ETADGEYQAKKAIVCAGTWVK	0	0
EG12669	IETADGEYQAKKAIVCAGTWV	0	0
EG12669	KAIVCAGTWVKDLLPELPVQP	0	0
EG12669	LAIKTWIQLAKEAGCAQLFNC	0	0
EG12669	LIRHAYGEGEKYVPLVLRAQT	0	0
EG12669	LPELPVQPVRKVFAWYQADGR	0	0
EG12669	__________MKYDLIIIGSGS	0	0
EG12669	PAENDALKIGKHNGGQVIHSA	0	0
EG12669	QADGRYSVKNKFPAFTGELPN	0	0
EG12669	SAEQWQLNVEKLDAQGIMARW	0	0
EG12669	SGFLRSELAIKTWIQLAKEAG	0	0
EG12669	WYQADGRYSVKNKFPAFTGEL	0	0
EG12669	YGFPAENDALKIGKHNGGQVI	0	0
EG12669	EIAADFAQDKKSDFDLTPFRL	0	1
EG12669	LITGLSGHGFKFASVLGEIAA	0	0
EG12712	PDEQRVADAWKAAMEDVLKVQ	0	0
EG12712	RMEAPAVRVAKTMNTPHGDAI	0	0
EG12712	VFDLRFCVPNKEVMPERGIHT	0	0
EG12712	AWKAAMEDVLKVQDQNQIPEL	0	1
EG12712	SNEELALPKEKLQELHI____	0	0
EG12982	DPIFFVPSEGKTAAELTREEK	0	0
EG12982	EDATDQKNLQKLLETMKDVPD	0	0
EG12982	KNLQKLLETMKDVPDDQRQAR	0	0
EG12982	KTAAELTREEKSAISHRGQAL	0	0
EG12982	RYSGEDATDQKNLQKLLETMK	0	0
EG12982	SAISHRGQALKLLLDALRNG_	0	0
EG12982	GLTFIENAILKARHAAKVTAL	0	1
EG12982	KVVLATGNVGKVRELASLLSD	0	1
EG12982	NAILKARHAAKVTALPAIADD	0	1
EG13014	AIVLPALWNEKRDTKRAKLLQ	0	0
EG13014	AVISRKTTGDKQAFHSAHVQP	0	0
EG13014	DGSSIPALLKKLEEHGMTQLG	0	0
EG13014	DPWHILQTGGKEIKSAIPMGC	0	0
EG13014	EQYVTKPVDAKIQDRFAEGIL	0	0
EG13014	ESNAGAVISRKTTGDKQAFHS	0	0
EG13014	FVHTVEQYVTKPVDAKIQDRF	0	0
EG13014	GVLDQVLDALKGMDVLEFGGI	0	0
EG13014	HILQTGGKEIKSAIPMGCVLT	0	0

EG13014	ILLTLIEDGPKALKEPENYDV	0	0
EG13014	LDGSSIPALLKKLEEHGMTQL	0	0
EG13014	LHTPTRILFGKGAIAGLREQI	0	0
EG13014	PALWNEKRDTKRAKLLQYAER	0	0
EG13014	TLIEDGPKALKEPENYDVRAN	0	0
EG13014	VGGGSVLDGTKFIAAAANYPE	0	0
EG13014	WNEKRDTKRAKLLQYAERVWN	0	0
EG13014	MNAVKLVREQKVTFLLAVGGG	0	0
EG13014	PAYETLMNAVKLVREQKVTFL	0	0
EG13014	VLITYGGGSVKKTGVLDQVLD	0	1
EG13952	DEKSALAYLEKHTARLKNLGV	0	0
EG13952	FLWKVWTESEKNHEAGGIYLF	0	0
EG13952	KNLGVEEVVAKVFDVNEPLSQ	0	0
EG13952	PFGDAMAEQLKPLAESINQEP	0	0
EG13952	SINQEPGFLWKVWTESEKNHE	0	0
EG13952	NEPLSQINQAKLA________	0	1
O05598	ADVEDLLDAGKPFGLRPSAIL	0	0
O05598	APNFAYALLAKRLRRQAKPGD	0	0
O05598	EGHVVVCGRVKDVIIMAGRNI	0	0
O05598	IPMFFGAELVKVTPMDFLRDT	0	0
O05598	LLAKRLRRQAKPGDFDLSTLR	0	0
O05598	MFVGAQYDVDKDVMVSWLPCF	0	0
O05598	____MSRFTEKMFHNARTATT	0	0
O05598	QLTSGSTGSPKAVQITHRNIY	0	0
O05598	TMTVIGMIEAKAVIVSEPFLV	0	0
O05598	AALRRAVPATKGNTRRLATLG	0	0
O05598	DFLRDTLLWAKLIDKYQGTMT	0	0
O05598	DTLLWAKLIDKYQGTMTAAPN	0	0
O05598	FLVAIPILEQKGMQVLTVADL	0	0
O05598	PGTIPKTPSGKLRRANSVTLV	0	0
O05814	AADTLKNVLIKVRQPGGDTEL	0	0
O05814	DDFAKHPFLVKGYIGPKALRE	0	0
O05814	DPADAEVASHKLLIRAGYIRP	0	0
O05814	DRQASPGVKFKDAELLGMPWI	0	0
O05814	EIGHIFQLGSKYTDAFTADVL	0	0
O05814	FPLTLYQIQTKYRDEARPRAG	0	0
O05814	FTADVLGEDGKPVRLTMGSYG	0	0
O05814	GILRAREFVMKDSYSFDIDAA	0	0
O05814	GRTVTAADTLKNVLIKVRQPG	0	0
O05814	GVPGDREVDDKRLGAALEPAD	0	0
O05814	LDDRQASPGVKFKDAELLGMP	0	0
O05814	LTVKGEYSSYKDFPLTLYQIQ	0	0
O05814	PFLVKGYIGPKALRENNVRYL	0	0
O05814	THEELFTLTVKGEYSSYKDFP	0	0
O05814	TQYGDSVFRLKDRRGNDYLLG	0	0
O05814	YALLDDDDFAKHPFLVKGYIG	0	0
O05814	PFDVHLVIANKDAQARAGATA	0	1
O06188	AHCPVAVMRSKVDQPASDINW	0	0

O06188	AVPLRLVSVIKPTHPSPDDYD	0	0
O06188	_MSGRGEPTMKTIIVGIDGSH	0	0
O06188	AVLEYAAREAKLRQAPILALG	0	0
O06188	EAQSAVEAAGKLVKIETDIPR	0	0
O06188	LGSTATELAEKAHCPVAVMRS	0	0
O06391	VWTLRLYLTGKRLWHDPRRRD	0	0
O33294	AGTILHTGDIKFDQLPPDGRP	0	0
O33294	ASISGAVPVGKMFVDGLIAGD	0	0
O33294	DHIGAIPFLLKLRPDIPVVGS	0	0
O33294	DPKALEPAVRKVEAELESLVA	0	0
O33294	GTWRMLRANAKLAASTGVPQE	0	0
O33294	IAQGVRRTVGKWVGETYRRQP	0	0
O33294	LRPDIPVVGSKFTLALVAEKC	0	0
O33294	NGVSVDLVAGKASISGAVPVG	0	0
O33294	SKFTLALVAEKCREYRITPVF	0	0
O33294	AVFGVIDALSKIGARVVTNAQ	0	1
O33341	FVLAVQWHPEKSLDDLRLFKA	0	1
O53176	CGLALALDRDKLSDMRGVLTP	0	0
O53176	RMAQDGDPGYKATSVLLGECG	0	0
O53176	TDTNCVVRSFKGGFSGGTIAS	0	0
O53176	VLYGATGFVGKLTAEYLARAG	0	0
O53176	YIRLLPRGLVKRVVPKPGTGP	0	0
O53176	PRGLVKRVVPKPGTGPSAAAR	0	1
O53204	AARPHRVTHRKLTRG______	0	0
O53204	AFGFKGPDQQKPAGVLSGGER	0	0
O53204	AWEGDDDNEAKWFWFEGNFGA	0	0
O53204	DQARAGIDPRKTVWEVVSDGL	0	0
O53204	DVTLSFYPGAKIGVVGPNGAG	0	0
O53204	EFIYTMKKVRKAHGDKVILDD	0	0
O53204	EGNFGAYEENKVERLGVDAAR	0	0
O53204	EGNYSTYLEKKAERLAVQGRK	0	0
O53204	EMAAEAEKTRKLDFEEIQIPV	0	0
O53204	GGERRRVALCKLLLSKPDLLL	0	0
O53204	GNVEEGMGDIKIKLDRFNEVA	0	0
O53204	IGVVGPNGAGKSSVLRIMAGL	0	0
O53204	LQQEPPLNEDKTVRGNVEEGM	0	0
O53204	__MAEFIYTMKKVRKAHGDKV	0	0
O53204	_MAEFIYTMKKVRKAHGDKVI	0	0
O53204	MKKVRKAHGDKVILDDVTLSF	0	0
O53204	PNGVGKTTLFKTIVGLETPDS	0	0
O53204	RNRLNLALTLKQGGNLILLDE	0	0
O53204	RVALCKLLLSKPDLLLLDEPT	0	0
O53204	RYEEMAAEAEKTRKLDFEEIQ	0	0
O53204	SGAKARQAKSKARLQRYEEMA	0	0
O53204	SRAYVSAFGFKGPDQQKPAGV	0	0
O53204	SVLRIMAGLDKPNNGDAFLAT	0	0
O53204	VEEGMGDIKIKLDRFNEVAEL	0	0
O53204	VGVIGPNGVGKTTLFKTIVGL	0	0

O53204	VQGRKDAKLQKRLTEELAWVR	0	0
O53204	YEGNYSTYLEKKAERLAVQGR	0	0
O53204	EELAWVRSGAKARQAKSKARL	0	1
O53204	GLETPDSGSVKVGETVKLSYV	0	1
O53204	KAERLAVQGRKDAKLQKRLTE	0	1
O53204	NVVVEVDHLDKGYDGRALIKD	0	1
O53204	RLAVQGRKDAKLQKRLTEELA	0	1
O53204	SGSVKVGETVKLSYVDQARAG	0	0
O53204	VRSGAKARQAKSKARLQRYEE	0	0
O53226	GAYGEKQMYGKTVQGVIRSTF	0	0
O53226	GKIVVAQYNVKATGHVAKLRR	0	0
O53226	GLNVVGISPDKPEKLATFRDA	0	0
O53226	________MTKTTRLTPGDKA	0	0
O53226	RSTFVVDEDGKIVVAQYNVKA	0	0
O53226	VLTAWGAYGEKQMYGKTVQGV	0	0
O53226	VVGISPDKPEKLATFRDAQGL	0	0
O53226	YPAASTPGCTKQACDFRDNLG	0	0
O53442	AWGLAGEPSLKQFVTG_____	0	0
O53442	LGADIEAYRDKLQNVADAGIF	0	0
O53442	_______MAQKPVADALTLEL	0	0
O53442	NEDVRVQHVMKGYRAEKYTQV	0	0
O53442	QNVADAGIFGKPQLRQLISDR	0	0
O53442	TDACEILLILKDNLAGHHREL	0	0
O53618	GAELANYRRNKVGIVFQAFNL	0	0
O53618	LSCLGGILRPKSGAIKFDEVD	0	0
O53618	VMLLGPSGCGKTTLLSCLGGI	0	0
O53618	ELADGGEELVKVAGPGDYFGE	0	0
O53665	ALEGFTRSLGKELRRGATTAL	0	0
O53665	ATGITEPAGLKGLHEFFTPVL	0	0
O53665	DDSTPPADWEKPLDGKVAIVT	0	0
O53665	ESTMRFLLSAKSAYVDGQVFS	0	0
O53665	LDVTADDAVDKISEHLRDHHG	0	0
O53665	LVNNAGITRDKLLANMDDARW	0	0
O53665	_______MAPKRSSDLFSQVV	0	0
O53665	NRGQTNYATTKAGMIGITQAL	0	0
O53665	PADWEKPLDGKVAIVTGAARG	0	0
O53665	SEHLRDHHGGKADILVNNAGI	0	0
O53665	AAENLAETASKVGGTALWLDV	0	1
O53665	VVEPLRAALEKDYDLVGNNLG	0	1
O53871	AFASVVLKFQKDLNIPDEKLN	0	0
O53871	DTTKEGLAKLKPAFEGLAALG	0	0
O53871	EAIRTPRGKQKNGSLHEVKPL	0	0
O53871	FQKDLNIPDEKLNVNGGAIAM	0	0
O53871	HDEHMRPDTTKEGLAKLKPAF	0	0
O53871	IYEAIRTPRGKQKNGSLHEVK	0	0
O53871	KQKNGSLHEVKPLSLVVGLID	0	0
O53871	LGGFDDVALQKYHWVEKINHV	0	0
O53871	LNEAFASVVLKFQKDLNIPDE	0	0

O53871	LVMIGSAAAGKLQGLTPRARI	0	0
O53871	LVVGLIDELRKRHPDLDENLI	0	0
O53871	MLTGPTPATRKVLDRAGLTVD	0	0
O53871	RPDTTKEGLAKLKPAFEGLAA	0	0
O53871	VALQKYHWVEKINHVHTGGNS	0	0
O53871	GLEAVNTAAQKVRSGWDDLVL	0	0
O53871	VDAYALRSQQKAAEAWSGGYF	0	0
O69687	IFTATGQHPLKFTAYDGSTAG	0	0
O69687	KNYPFYFGFLKSKLRTGGLLL	0	0
O69687	LKTLTERVDFKRPSARVLANV	0	0
O69687	NLQLHHVLATKVDPRGDDSLP	0	0
O69687	RRMANGLLHSKTRDAEAIHHH	0	0
O69687	SIGLTEHIGVKNYPFYFGFLK	0	0
O69687	VHPGDPYELLKTLTERVDFKR	0	0
O69687	YPFYFGFLKSKLRTGGLLLNH	0	0
O69687	AVAEVGLPTAKVWGLYMAASR	0	0
O69687	EASLEQAQENKYRLIFEKLRL	0	0
O69687	LSAEQAKWGQKAVEDEGLSDL	0	0
O69687	QENKYRLIFEKLRLEPGDRLL	0	0
O86352	MTEPAAWDEGKPRIITLTMNP	0	0
O86352	QHISSGVFLLKASVRELRECV	0	0
O86352	SFTVNESRTAKQYRFVLPGPS	0	0
O86352	TSVDVVRPTEKMRCGAPRYDP	0	0
P09621	AKVNIKPLEDKILVQANEAET	0	0
P09621	ASGLVIPDTAKEKPQEGTVVA	0	0
P09621	GLVIPDTAKEKPQEGTVVAVG	0	0
P09621	________MAKVNIKPLEDKI	0	0
P09621	____MAKVNIKPLEDKILVQA	0	0
P09621	VAEGDTVIYSKYGGTEIKYNG	0	0
P09621	GPGRWDEDGEKRIPLDVAEGD	0	1
P09621	IYSKYGGTEIKYNGEEYLILS	0	1
P0A4X0	AAMSATEPLTKTTITRRDPGP	0	0
P0A4X0	GANFTYNSIGKDGQPTQGGYS	0	0
P0A4X0	GICHSDIHTVKAEWGQPNYPV	0	0
P0A4X0	GLGGLGHMGVKLGAAMGADVT	0	0
P0A4X0	PGPHDVAIDIKFAGICHSDIH	0	0
P0A4X0	SCTRGIEQYCKPGANFTYNSI	0	0
P0A4X0	YATADPDTFRKLRGGFDLILN	0	0
P0A4X0	DVTVLSQSLKKMEDGLRLGAK	0	0
P0A4X0	KMEDGLRLGAKSYYATADPDT	0	1
P0A4X0	VVTAVGSEVTKYRQGDRVGVG	0	1
P0A508	AAEVGYPVLIKPSAGGGGKGM	0	0
P0A508	GDVVVVVEAMKMEHSLEAPVS	0	0
P0A508	IEVMGDKIAAKNAVAAFDVPV	0	0
P0A508	LIKPSAGGGGKGMRLVQDPAR	0	0
P0A508	PARAIEVMGDKIAAKNAVAAF	0	0
P0A508	RECSLQRRHQKVIEEAPSPLL	0	0
P0A508	TWHLREAEEHKIHRAVGARPA	0	0

P0A508	VGSDYDPLLTKVIAHGADREE	0	0
P0A508	PARESYLDIGKVLDAAARTGA	0	0
P0A508	QVLVSVGDQVKVEQVLARIKD	0	0
P0A508	VKVEQVLARIKD________	0	0
P0A520	AVVADKPEKEKASVPGGGDMG	0	0
P0A520	FLTTEAVVADKPEKEKASVPG	0	0
P0A520	GDEATGANIVKVALEAPLKQI	0	0
P0A520	IVKVALEAPLKQIAFNSGLEP	0	0
P0A520	KVTETLLKGAKEVETKEQIAA	0	0
P0A520	LKRGIEKAVEKVTETLLKGAK	0	0
P0A520	LLKGAKEVETKEQIAATAAIS	0	0
P0A520	LQAAPTLDELKLEGDEATGAN	0	0
P0A520	PLLEKVIGAGKPLLIIAEDVE	0	0
P0A520	TEAVVADKPEKEKASVPGGGD	0	0
P0A520	TVKDLLPLLEKVIGAGKPLLI	0	0
P0A520	ADLSLLGKARKVVVTKDETTI	0	1
P0A520	LENADLSLLGKARKVVVTKDE	0	1
P0A520	RGLNALADAVKVTLGPKGRNV	0	0
P0A520	SGLEPGVVAEKVRNLPAGHGL	0	0
P0A556	AFKIAGSQVLKKAAALAQPVI	0	0
P0A556	AGHIYAVIGLKDTTTGDTLSD	0	0
P0A556	ANVGKPQVAYKETIKRLVQNV	0	0
P0A556	ATKGKKERLGKLFQMHSNKEN	0	0
P0A556	DPVIEVAIEPKTKSDQEKLSL	0	0
P0A556	DQEKLSLSIQKLAEEDPTFKV	0	0
P0A556	EEGATYEFESKVTGGRIPREY	0	0
P0A556	FGIMAHIDAGKTTTTERILYY	0	0
P0A556	FKIAGSQVLKKAAALAQPVIL	0	0
P0A556	FKIATHPFFGKLTYIRVYSGT	0	0
P0A556	HEVDSSEMAFKIAGSQVLKKA	0	0
P0A556	IEPKTKSDQEKLSLSIQKLAE	0	0
P0A556	ILVDRMRREFKVEANVGKPQV	0	0
P0A556	ITSAATTTFWKDNQLNIIDTP	0	0
P0A556	IYPVLCGSAFKNKGVQPMLDA	0	0
P0A556	KPQVAYKETIKRLVQNVEYTH	0	0
P0A556	LDGAVAVFDGKEGVEPQSEQV	0	0
P0A556	LGKLFQMHSNKENPVDRASAG	0	0
P0A556	LVQNVEYTHKKQTGGSGQFAK	0	0
P0A556	MFGYVGDLRSKTQGRANYSMV	0	0
P0A556	PRICFVNKMDKIGADFYFSVR	0	0
P0A556	PVLCGSAFKNKGVQPMLDAVV	0	0
P0A556	QKLAEEDPTFKVHLDSETGQT	0	0
P0A556	QSEQVWRQADKYDVPRICFVN	0	0
P0A556	REFKVEANVGKPQVAYKETIK	0	0
P0A556	RILYYTGINYKIGEVHDGAAT	0	0
P0A556	RLVQNVEYTHKKQTGGSGQFA	0	0
P0A556	TVDEIKGAIRKLTIASEIYPV	0	0
P0A556	VAESDEHLLEKYLGGEELTVD	0	0

P0A556	VIEVAIEPKTKSDQEKLSLSI	0	0
P0A556	VLAGYPLVNLKVTLLDGAYHE	0	0
P0A556	VPPAIGHAPAKEDEEVVRKAT	0	0
P0A556	YDVPRICFVNKMDKIGADFYF	0	0
P0A556	ESGSQVINATKGKKERLGKLF	0	1
P0A556	GGEELTVDEIKGAIRKLTIAS	0	1
P0A556	GSQVINATKGKKERLGKLFQM	0	1
P0A556	GVVDLVEMNAKVWRGETKLGE	0	0
P0A556	KQTGGSGQFAKVIINLEPFTG	0	0
P0A556	LAEQAEEYRTKLLEVVAESDE	0	0
P0A556	_______MAQKDVLTDLSRVR	0	0
P0A556	MNAKVWRGETKLGETYDTVEI	0	0
P0A556	PANVSKEIIAKATGE______	0	0
P0A556	SQVINATKGKKERLGKLFQMH	0	0
P0A556	SYSEVPANVSKEIIAKATGE_	0	0
P0A556	TDEPFAALAFKIATHPFFGKL	0	0
P0A5B7	IMVRDGQLTIKAERTEQKDFD	0	0
P0A5B7	TRLMRLEDEMKEGRYEVRAEL	0	0
P0A5B7	VAVSEGKPTEKHIQIRSTN__	0	0
P0A5B7	DEDDIKATYDKGILTVSVAVS	0	0
P0A5F7	EWRNLEDEQEKMLARRLSGWQ	0	0
P0A5F7	FEYSRFQEAQKREIVEHSYLV	0	0
P0A5F7	QDRYPDVVVHKVVVCDRPAPR	0	0
P0A5H3	AAEERGYTATKHQREVGAGYF	0	0
P0A5H3	AYNCSPSFNWKKHLDDATIAK	0	0
P0A5H3	ERTREGFYRTKNGIEPCIARA	0	0
P0A5H3	GGALNVYELQKALIAAGVAGS	0	0
P0A5H3	HWEDQLASEKKCGHLGGKVLI	0	0
P0A5H3	MAVQQVRAGLKAIYLSGWQVA	0	0
P0A5H3	NALQRADQIAKIEGDTSVENW	0	0
P0A5H3	NGIEPCIARAKAYAPFADLIW	0	0
P0A5H3	SEKKCGHLGGKVLIPTQQHIR	0	0
P0A5H3	SHWEDQLASEKKCGHLGGKVL	0	0
P0A5H3	YNCSPSFNWKKHLDDATIAKF	0	0
P0A5H3	EAARQFSEAVKAEYPDQMLAY	0	1
P0A5H3	FQKELAAMGFKFQFITLAGFH	0	0
P0A5H3	KKHLDDATIAKFQKELAAMGF	0	0
P0A5H3	___MSVVGTPKSAEQIQQEWD	0	0
P0A5H3	QQEWDTNPRWKDVTRTYSAED	0	0
P0A5L2	AVGGAKNSVLKLMAATLLAEG	0	0
P0A5L2	CVLGPLVGRCKRARVALPGGD	0	0
P0A5L2	LSGEVAVGGAKNSVLKLMAAT	0	0
P0A5L2	SFRVTQYERPKAVNVATLPFP	0	0
P0A5L2	DPAHLQLVLHKLHDAGATVTQ	0	0
P0A5U4	AGIYGKLAWEKTIPNWFFEPD	0	0
P0A5U4	ALELAVAQIEKSYGKGSVMRL	0	0
P0A5U4	AVAQIEKSYGKGSVMRLGDEA	0	0
P0A5U4	FGSPETTTGGKALKFYASVRM	0	0

P0A5U4	GAIVWATPDHKVLTEYGWRAA	0	0
P0A5U4	GDGRDGWVGGKTPINFINVQR	0	0
P0A5U4	KQAEFDILYGKGISREGSLID	0	0
P0A5U4	__MTQTPDREKALELAVAQIE	0	0
P0A5U4	NRTRVKVVKNKCLAEGTRIFD	0	0
P0A5U4	PETTTGGKALKFYASVRMDVR	0	0
P0A5U4	QARLMSQALRKMTGALNNSGT	0	0
P0A5U4	RMDVRRVETLKDGTNAVGNRT	0	0
P0A5U4	SIVNGRRIQSKRQVFEVRISG	0	0
P0A5U4	STVRDYDPTQKRPSIVNGRRI	0	0
P0A5U4	TAIFINQLRDKIGVMFGSPET	0	0
P0A5U4	TNAVGNRTRVKVVKNKCLAEG	0	0
P0A5U4	TYEGEQLGQGKENARNFLVEN	0	0
P0A5U4	VASGDPRGGMKQVLGASRLRR	0	0
P0A5U4	VGNRTRVKVVKNKCLAEGTRI	0	0
P0A5U4	VQALADALDDKFLHDMLAEEL	0	0
P0A5U4	VVVHNCSPPFKQAEFDILYGK	0	0
P0A5U4	YGWRAAGELRKGDRVAQPRRF	0	0
P0A5U4	ADEIEKKIKEKLGIGAVVTDD	0	1
P0A5U4	AEHALDPDYAKKLGVDTDSLL	0	1
P0A5U4	DLCQQAGIYGKLAWEKTIPNW	0	1
P0A5U4	DMGVDQGLIRKSGAWFTYEGE	0	0
P0A5U4	DVADEIEKKIKEKLGIGAVVT	0	0
P0A5U4	EHALDPDYAKKLGVDTDSLLV	0	0
P0A5U4	HRIEDVVDGRKPIHVVAAAKD	0	0
P0A5U4	IEIYGPESSGKTTVALHAVAN	0	0
P0A5U4	NADVADEIEKKIKEKLGIGAV	0	0
P0A5U4	RKPIHVVAAAKDGTLHARPVV	0	0
P0A5Z4	ADVEIDVPAHKVTRNGEQISL	0	0
P0A5Z4	DDYIMKPFKPKELVARVRARL	0	0
P0A5Z4	EFDLLVALARKPRQVFTRDVL	0	0
P0A5Z4	GADDYIMKPFKPKELVARVRA	0	0
P0A5Z4	LESGADDYIMKPFKPKELVAR	0	0
P0A5Z4	SGVPIVMLTAKTDTVDVVLGL	0	0
P0A5Z4	VVLTVRGVGYKAGPP______	0	0
P0A5Z4	VNVHVQRLRAKVEKDPENPTV	0	0
P0CG95	LTLPASVDLEKETVITGRVVD	0	0
P0CG95	_____MCSGPKQGLTLPASVD	0	0
P0CG99	AFKYYTSYTLKTFDGKRYLER	0	0
P0CG99	AQIYAWRKGIKTLYYIRLRQM	0	0
P0CG99	ASKIEIRKEGKIGRVYYPAPY	0	0
P0CG99	ATPTFLNSGKKQRGEPVSCFL	0	0
P0CG99	DIHIPTQDDWKQLKESVQKHG	0	0
P0CG99	DQGLSLTLFFKDTATTRDVNK	0	0
P0CG99	DWKQLKESVQKHGIYNQNLQA	0	0
P0CG99	ERGTHFKGFEKSKYASGEFFD	0	0
P0CG99	FRFPTFLGAFKYYTSYTLKTF	0	0
P0CG99	GTHFKGFEKSKYASGEFFDKY	0	0

P0CG99	HPDIYRFLDTKRENADEKIRI	0	0
P0CG99	HPVASKIEIRKEGKIGRVYYP	0	0
P0CG99	IPTQDDWKQLKESVQKHGIYN	0	0
P0CG99	IQFEKDREAAKQYFLQHVNQN	0	0
P0CG99	KDTATTRDVNKAQIYAWRKGI	0	0
P0CG99	KIAIERGTHFKGFEKSKYASG	0	0
P0CG99	LDTKRENADEKIRIKTLSLGV	0	0
P0CG99	MVDDARIRKTKINAREFFQTL	0	0
P0CG99	NDDLSYAKVGKDISCNLGSLN	0	0
P0CG99	NQSSGVIPIMKLLEDSFSYAN	0	0
P0CG99	NTVFFHSQDEKLDYLIENEYY	0	0
P0CG99	RSINSALQLSKRGGGVALLLS	0	0
P0CG99	SCNLGSLNIAKAMDSPDFAQT	0	0
P0CG99	SEFNDDLSYAKVGKDISCNLG	0	0
P0CG99	SKYASGEFFDKYTDQVWEPKT	0	0
P0CG99	SNIREHGAPIKNIENQSSGVI	0	0
P0CG99	STSSIHPVASKIEIRKEGKIG	0	0
P0CG99	TDQVWEPKTDKVRRLFADADI	0	0
P0CG99	TSYTLKTFDGKRYLERFEDRV	0	0
P0CG99	TVNRSNPIAGKITHSNLCSEI	0	0
P0CG99	VNKAQIYAWRKGIKTLYYIRL	0	0
P0CG99	VPFADVSVTEKYYEMVDDARI	0	0
P0CG99	YDADGKIQFEKDREAAKQYFL	0	0
P0CG99	YEMVDDARIRKTKINAREFFQ	0	0
P0CG99	AMLNLYDADGKIQFEKDREAA	0	1
P0CG99	DKYTDQVWEPKTDKVRRLFAD	0	1
P0CG99	IKSLLDRAYAKKFRFPTFLGA	0	1
P0CG99	KSLLDRAYAKKFRFPTFLGAF	0	0
P0CG99	LAAGDTELAEKLVDEIIDGRF	0	0
P0CG99	PATPTFLNSGKKQRGEPVSCF	0	0
P0CG99	RENADEKIRIKTLSLGVVIPD	0	0
P0CG99	VIPDITFELAKKNEDMYLFSP	0	0
P0CG99	VLDQYSRDFIKSLLDRAYAKK	0	0
P0CG99	VLFHALRASNKIAIERGTHFK	0	0
P0CG99	YQDAYEIGYEKIIDTYAAATQ	0	0
P0CH36	GICHSDIHTVKAEWGVPNYPV	0	0
P0CH36	GLGGLGHVAVKLAKAMGADVT	0	0
P0CH36	LDLGAYLGLLKLDGALVELGL	0	0
P0CH36	LRHWNAGPGKKVAVIGLGGLG	0	0
P0CH36	PLRHWNAGPGKKVAVIGLGGL	0	0
P0CH36	TEVGSEVTKYKVGDRVGVGCF	0	0
P0CH36	VDSCRECDNCKAGLEQYCTGT	0	0
P0CH36	VVTEVGSEVTKYKVGDRVGVG	0	0
P0CH36	YATSDPETFDKLAGSFDLILN	0	0
P0CH36	AATSATEPLTKTTITRRAVGP	0	1
P0CH36	DVTVLSQSLKKMEDGLRLGAS	0	0
P0CH36	GLGHVAVKLAKAMGADVTVLS	0	0
P17670	AGHVNHTIWWKNLSPNGGDKP	0	0

P17670	AIADAFGSFDKFRAQFHAAAT	0	0
P17670	AVAKLEEARAKEDHSAILLNE	0	0
P17670	EDHSAILLNEKNLAFNLAGHV	0	0
P17670	HAFYLQYKNVKVDFAKAFWNV	0	0
P17670	MWEHAFYLQYKNVKVDFAKAF	0	0
P17670	QYKNVKVDFAKAFWNVVNWAD	0	0
P17670	YVKGANDAVAKLEEARAKEDH	0	0
P17670	AALGWDTLGNKLLIFQVYDHQ	0	0
P17670	HHSKHHATYVKGANDAVAKLE	0	0
P17670	SGQINELHHSKHHATYVKGAN	0	0
P17670	WKNLSPNGGDKPTGELAAAIA	0	0
P60176	ADIVVTATGNKDIIMLEHIKA	0	0
P60176	ADLSLADFGRKELRIAEHEMP	0	0
P60176	DKWTKIAESVKGVTEETTTGV	0	0
P60176	DVGKGCAEAMKGQGARVSVTE	0	0
P60176	EPKGVPVFAWKGETLEEYWWA	0	0
P60176	EQMLTWPDPDKPANMILDDGG	0	0
P60176	ERSGATRVNVKPQVDLWTFGD	0	0
P60176	EYDNEVYRLPKHLDEKVARIH	0	0
P60176	HVEALGGHLTKLTKEQAEYLG	0	0
P60176	IIMLEHIKAMKDHAILGNIGH	0	0
P60176	LRTRFETDKDKWTKIAESVKG	0	0
P60176	MLVLRGMQYEKAGVVPPAEED	0	0
P60176	___MTGNLVTKNSLTPDVRNG	0	0
P60176	NKDIIMLEHIKAMKDHAILGN	0	0
P60176	NLLRTRFETDKDKWTKIAESV	0	0
P60176	NRGTDALIGGKKVLICGYGDV	0	0
P60176	PAEEDDPAEWKVFLNLLRTRF	0	0
P60176	RFETDKDKWTKIAESVKGVTE	0	0
P60176	RGTDALIGGKKVLICGYGDVG	0	0
P60176	RREYAEVQPLKGARISGSLHM	0	0
P60176	VGPHGTPDEPKGVPVFAWKGE	0	0
P60176	VLICGYGDVGKGCAEAMKGQG	0	0
P60176	VYRLPKHLDEKVARIHVEALG	0	0
P60176	YLGVDVEGPYKPDHYRY____	0	0
P60176	QTIAQIELWTKNDEYDNEVYR	0	1
P60176	TPDVRNGIDFKIADLSLADFG	0	1
P60796	EGVFVGSGIFKSGAPEHRAAA	0	0
P60796	GNPATGTARVKRGMAEMLKGG	0	0
P60796	IAAVTIPVMAKVRIGHFVEAQ	0	0
P60796	ISEGAAMIRSKGEAGTGDVSN	0	0
P60796	MSEDELFVAAKELQAPYELVA	0	0
P60796	TPADYAHHIDKWNFTVPFVCG	0	0
P60796	APEHRAAAIVKATTFFDDPDV	0	1
P60796	ELVAEVARAGKLPVTLFTAGG	0	0
P63345	AKAVANSLAKKMAEVRGDDAH	0	0
P63345	ARNSKLMETLKEARQQLLALR	0	0
P63345	CSPNIDAASLKKGQTVRLNEA	0	0

P63345	DAEAAQDIYSKYLTEFLPVHA	0	0
P63345	DTVDVFTSGRKMRLTCSPNID	0	0
P63345	EFDGDRSACIKAMIEKVVDRM	0	0
P63345	EVRGDDAHEAKSYFLNIKGPE	0	0
P63345	HEAKSYFLNIKGPELLNKFVG	0	0
P63345	LYREYSLRPPKGVLLYGPPGC	0	0
P63345	RDAVELPFLHKELYREYSLRP	0	0
P63345	RIDSLAARNSKLMETLKEARQ	0	0
P63345	RSACIKAMIEKVVDRMYAEID	0	0
P63345	SPNIDAASLKKGQTVRLNEAL	0	0
P63345	AMIQNVVDRAKKNAIKSVLET	0	1
P63345	EALNDDTRPRKLRPGDSLLVD	0	0
P63345	MIQNVVDRAKKNAIKSVLETG	0	0
P63345	VVDRAKKNAIKSVLETGQPGL	0	0
P63458	ELARRCVLAGKDVIVAGHSVG	0	0
P63458	ELRGVPARAVKSPADLDELAN	0	0
P63458	LAATRGAEMAKACATEPTGMS	0	0
P63458	PPAGTLSGIAKRELRGVPARA	0	0
P63458	TATLLSNRDGKPVTSAAAAMD	0	0
P63458	GAADQIAAWSKAADLDLARLG	0	1
P63458	LEKLAEDPPAKARVRALGVAG	0	0
P63523	AISVPVDHTEKLVTVIELKLL	0	0
P63523	EDEMFIVGRMKDMLIVYGRNH	0	0
P63523	GDNVAEGYWNKPDETRHTFGA	0	0
P63523	GEAMDELDVIKNNVTAAISRS	0	0
P63523	LTGNSPSFRVKDLPSAAYLQY	0	0
P63523	PAGTIGEIWVKGDNVAEGYWN	0	0
P63523	PGSIPTTTSGKIRRAACVEQY	0	0
P63523	TEKLVTVIELKLLGDSAGEAM	0	0
P63523	LYVASRNSGDKPEVVYFEPDK	0	0
P63523	PNFAFELAVRKTTDADIEGLD	0	1
P63523	STGSANRCEPKTGTPLLSYGM	0	1
P63568	FKLSRLTGRTKLVAAHDAFHG	0	0
P63568	GITPDVVTLAKGLGGGLPIGA	0	0
P63568	GSLALTGQPAKQTPFAPLPGD	0	0
P63568	LGIALTAPHAKDAEATARDAG	0	0
P63568	SGAEANEAAFKLSRLTGRTKL	0	0
P63673	AIAQHWMYEGKHVLIIFDDLT	0	0
P63673	AVKVKKPAPKKKK________	0	0
P63673	DEDKLAKEAVKVKKPAPKKKK	0	0
P63673	DEHVEALDEDKLAKEAVKVKK	0	0
P63673	DKLAKEAVKVKKPAPKKKK__	0	0
P63673	EAVKVKKPAPKKKK_______	0	0
P63673	ERGARLVELLKQPQSQPMPVE	0	0
P63673	FENIEEGQQVKRTGEVLSVPV	0	0
P63673	GQRQLIIGDRKTGKTAVCVDT	0	0
P63673	HVLIIFDDLTKQAEAYRAISL	0	0
P63673	KLAKEAVKVKKPAPKKKK___	0	0

P63673	KVKKPAPKKKK__________	0	0
P63673	LHSRLLERCAKLSDDLGGGSL	0	0
P63673	QLIIGDRKTGKTAVCVDTILN	0	0
P63673	QRQNWESGDPKKQVRCVYVAI	0	0
P63673	RQNWESGDPKKQVRCVYVAIG	0	0
P63673	SLTGLPIIETKANDISAYIPT	0	0
P63673	VEALDEDKLAKEAVKVKKPAP	0	0
P63673	VGGAAQIKAMKEVAGSLRLDL	0	0
P63673	VKVKKPAPKKKK_________	0	0
P63673	VSRVGGAAQIKAMKEVAGSLR	0	0
P63673	AAAASESAGFKWLAPYTGSAI	0	1
P63673	AFASDLDAASKAQLERGARLV	0	1
P63673	APSVVHRQGVKEPLQTGIKAI	0	0
P63673	DKLTEVIKNFKKGFAATGGGS	0	0
P63673	EAADKLTEVIKNFKKGFAATG	0	0
P63673	EILTEIRDSQKLTEEAADKLT	0	0
P63673	GVKEPLQTGIKAIDAMTPIGR	0	0
P63673	KLTEVIKNFKKGFAATGGGSV	0	0
P63673	VRCVYVAIGQKGTTIAAVRRT	0	0
P64245	AVTNQWVNSYKRLVQGGEAPT	0	0
P64245	DDPLQLSEVGKSFIAGILEHA	0	0
P64245	DNVMTFRYVIKEVALEEGARA	0	0
P64245	EEGARASFMPKPFGQHPGSAM	0	0
P64245	EHVFDFFLRNKRTEWANYRSH	0	0
P64245	LVRVPMYTPHKTSSRRVEVRS	0	0
P64245	LWFTDVLGFLKSVAIAPAELE	0	0
P64245	VHPEIEFFLLKPGPEDGSVPV	0	0
P64245	PRHVLRRQLTKAGELGFSCYV	0	1
P65161	KQLNKLINVIKIVEQDDEHSV	0	0
P65161	LTVEATGNRGKLEALLRVLEP	0	0
P65161	SRGPRGIGTAK__________	0	0
P65161	THTLSVLVEDKPGVLARVAAL	0	0
P65161	AEDTPLEQITKQLNKLINVIK	0	1
P65161	HSVSRELALIKVQADAGSRSQ	0	1
P65161	_______MSPKTHTLSVLVED	0	1
P65232	ISARLIADLLKTAGADRIVTV	0	0
P65232	LGGVPLAFIHKTRDPRVPNQV	0	0
P65232	MIDALKRGSAKRITAVMPFYP	0	0
P65232	MPFYPYARQDKKHRGREPISA	0	0
P65232	_MSHDWTDNRKNLMLFAGRAH	0	0
P65232	PDSGRVRIAEKWADALGGVPL	0	0
P65232	PFYPYARQDKKHRGREPISAR	0	0
P65232	MEQLIMIDALKRGSAKRITAV	0	1
P65232	VTNTLPIGEDKRFPQLTVLSI	0	1
P65277	EAFEGACAYWKPRTLPAP___	0	0
P65277	GLDDIALTLRKLDEIEAFEGA	0	0
P65277	TDQIIPAVFLKRVTRTGFEDG	0	0
P65277	FGDIFRGNAGKAGLLAAEVAQ	0	0

P65277	VAQDDVELLWKLIEQSPGLEI	0	0
P65573	LVDSLRSDTPKAPTRGAPLCG	0	0
P65573	MGGDAIFDRLKEHLGVGHDET	0	0
P65573	PTLAGLQVARKNDMQAPPTPG	0	0
P65880	ACEFKNQVLVKIYNRKALDPA	0	0
P65880	DKVTERYMGSKKIGTTGRGIG	0	0
P65880	DVLDPEQLTHKVEAACEFKNQ	0	0
P65880	GAQWGDEGKGKATDLLGGRVQ	0	0
P65880	GLQDRGVDTAKLLISADAHLL	0	0
P65880	GRGIGPCYQDKIARIGIRVAD	0	0
P65880	KVTERYMGSKKIGTTGRGIGP	0	0
P65880	LIGAQWGDEGKGKATDLLGGR	0	0
P65880	LLMPYHIAIDKVTERYMGSKK	0	0
P65880	PMTQRDLCRAKPVYEELPGWW	0	0
P65880	THKVEAACEFKNQVLVKIYNR	0	0
P65880	TRIGTVLGILKAYTTRVGSGP	0	0
P65880	VNGITDYFLTKLDVLSSLESV	0	0
P65880	DALLEQAEGFKHRIADTRLLL	0	0
P65880	GAREFDDLPAKARDYVLRLEQ	0	0
P65880	NQVLVKIYNRKALDPAQVVDA	0	0
P66056	APKKKVAGLIKLQIVAGQANP	0	0
P66056	DQVREIAETKKTDLNANDVDA	0	0
P66056	GVAKGSAEPHKTKVAKVTWDQ	0	0
P66056	KLLLKAAGVAKGSAEPHKTKV	0	0
P66056	KTPPAAKLLLKAAGVAKGSAE	0	0
P66056	LNANDVDAAAKIIAGTARSMG	0	0
P66056	_______MAPKKKVAGLIKLQ	0	0
P66056	______MAPKKKVAGLIKLQI	0	0
P66056	_____MAPKKKVAGLIKLQIV	0	0
P66056	QHGVNIMEFCKAYNAATENQR	0	0
P66056	TFTLKTPPAAKLLLKAAGVAK	0	0
P66056	WDQVREIAETKKTDLNANDVD	0	0
P66056	YEDRSFTFTLKTPPAAKLLLK	0	0
P66056	AKGSAEPHKTKVAKVTWDQVR	0	0
P66902	GNAGNITAYWKGYTEYHQLGL	0	0
P66902	KLAQAVMHGAKIIQIDGNFDD	0	0
P66902	NGLKDPDTALKDMPSVSPVPV	0	0
P66902	SKQTGCTIHLKVEGLNPTGSF	0	0
P66902	SVNPVRIEGQKTAAFEIVDVL	0	0
P66902	TSAVEAQQQSKGRFLAASDEE	0	0
P66902	VEGLNPTGSFKDRGMTMAVTD	0	0
P66902	VVCTVTGNGLKDPDTALKDMP	0	0
P66902	ASAASIAGLLKAIDDGWVARG	0	0
P66902	TEYHQLGLIDKLPRMLGTQAA	0	0
P66902	TPLIAATNLSKQTGCTIHLKV	0	0
P66902	VPVDPVAVVEKLGLA______	0	0
P69440	DTDDVILNRMKVYRDETAPLL	0	0
P69440	EEGTKLGVEAKRYLDAGDLVP	0	0

P69440	LFRRNIEEGTKLGVEAKRYLD	0	0
P69440	PLLEYYRDQLKTVDAVGTMDE	0	0
P69440	VLLLGPPGAGKGTQAVKLAEK	0	0
P69440	VSEEVLLERLKGRGRADDTDD	0	0
P69440	KGTQAVKLAEKLGIPQISTGE	0	0
P69440	PGAGKGTQAVKLAEKLGIPQI	0	0
P69440	VFARALRALGK__________	0	0
P71703	AIFASENNTIKIADVAPTNVI	0	0
P71703	ARVPIVGDDIKSQVGATITHR	0	0
P71703	AVTSNLKREFKTKDVHIGPSD	0	0
P71703	FGDVPLNLEYKLEVWDSPNSA	0	0
P71703	FIASDPVWAKKFTDARVPIVG	0	0
P71703	GATITHRVLAKLFEDRGVQLD	0	0
P71703	IDAVRAAKIAKDRGIGGPVIP	0	0
P71703	MLERERLESKKISKTQAVTSN	0	0
P71703	NMLERERLESKKISKTQAVTS	0	0
P71703	PVIPASAYLMKSPPEQLPDDI	0	0
P71703	QRGPTLDGIGKYYADTIELSD	0	0
P71703	RERLESKKISKTQAVTSNLKR	0	0
P71703	RFGPYHVRDVKFVAAFDVDAK	0	0
P71703	SDHVGWLDDRKWAYVRLEGRA	0	0
P71703	SKTQAVTSNLKREFKTKDVHI	0	0
P71703	TSNLKREFKTKDVHIGPSDHV	0	0
P71703	VDVVQALKEAKVDVLVSYLPV	0	0
P71703	VFIASDPVWAKKFTDARVPIV	0	0
P71703	AEPVDVVQALKEAKVDVLVSY	0	1
P71703	FVAAFDVDAKKVGFDLSDAIF	0	0
P71703	GVIIDAVRAAKIAKDRGIGGP	0	0
P71703	YLPVGSEEADKFYAQCAIDAG	0	0
P71724	LIAALSAALLKLPVANWPALG	0	0
P71724	DLRRAYDTAVKLGERTGLVVR	0	1
P71973	AEHGMAARGYKDVRANTVPAF	0	0
P71973	VGLHRPAEFNKSHIPAFLAGE	0	0
P77899	GVLPYLRPDGKTQVTIAYEDN	0	0
P77899	ILEIGYDSSDKGFDGATCGVN	0	0
P77899	_______MSEKGRLFTSESVT	0	0
P77899	RLSRRLTEVRKNGVLPYLRPD	0	0
P77899	TQHAADIDLEKTLDPDIREKV	0	0
P77899	TVRVLVNPTGKFVLGGPMGDA	0	0
P77899	DVELPWEQLDKVDDLKRAI__	0	1
P77899	EKTLDPDIREKVLNTVLDDLA	0	0
P77899	HVVGEVTTSAKEAFADITNTV	0	0
P77899	TETEDPVKIEKAIGEVFDLRP	0	1
P77899	WEQLDKVDDLKRAI_______	0	1
P96382	PGTRMRSDTPKVLHTLAGRSM	0	0
P96382	QRNIENWVQRKRPGSPAAQAS	0	0
P96382	RPGSPAAQASKRASEMACQQP	0	0
P96382	SVFVNYDGTSKRRTTVGSHVR	0	0

P96382	MLSHVLHAIAKLAPQRLIVVL	0	1
P96382	RPGTALGADGKLGAFVEVKNS	0	0
P96382	VKNSTIGTGTKVPHLTYVGDA	0	0
P96825	AGILRDGTFHKMSFENWDAVL	0	0
P96825	ATEDGAANIIKTALDEFGAVH	0	0
P96825	LFGNDGANFDKPPSVQDVAAR	0	0
P96825	LINTLALEGAKYNIHANALAP	0	0
P96825	MSFENWDAVLKVHLYGGYHVL	0	0
P96825	NASVYVVGGGKVQRVALFGND	0	0
P96825	NFGQTNYGAAKLGLVGLINTL	0	0
P96825	QDILPPEVLEKLTPEFVAPVV	0	0
P96825	ADEVVAEIRDKGGRAVANYDS	0	0
P96825	DLSGAKIAGFKL_________	0	0
Q10504	AKTIKGYALGKHFEGRNATHQ	0	0
Q10504	ATTMATVRTFKEVLRDKQIGP	0	0
Q10504	AVDADLMLAYKESEVGQILHE	0	0
Q10504	EGRNATHQMKKLTLEDLKEFR	0	0
Q10504	EVGFNHFFRGKSHPGGGDQVF	0	0
Q10504	FEGRNATHQMKKLTLEDLKEF	0	0
Q10504	FNHYLHDRGIKDTSDQHVWCF	0	0
Q10504	GFVPERRTKSKALTLPGRDIY	0	0
Q10504	HKGQPTVILAKTIKGYALGKH	0	0
Q10504	LGGFVPERRTKSKALTLPGRD	0	0
Q10504	LPGRDIYAPLKKGSGHQEVAT	0	0
Q10504	NLKRGGHDYRKVYAAYRAAVD	0	0
Q10504	NMSDQDIWNLKRGGHDYRKVY	0	0
Q10504	NRDGVAIETEKLRHPDRPAGV	0	0
Q10504	PGRDIYAPLKKGSGHQEVATT	0	0
Q10504	QMKKLTLEDLKEFRDTQRIPV	0	0
Q10504	QPTVILAKTIKGYALGKHFEG	0	0
Q10504	TTPDGDYQTYKANDGGYVRDH	0	0
Q10504	VRTFKEVLRDKQIGPRIVPII	0	0
Q10504	YAAYRAAVDHKGQPTVILAKT	0	0
Q10504	YHAATEQRTNKAQILASGVAM	0	0
Q10504	FFRGAGWNVIKVVWGREWDAL	0	1
Q10530	ALTDDYFIERKLYPNVDFYTG	0	0
Q10530	DDVSEFVRKVKNREAGVKLMG	0	0
Q10530	GGDDSLLGIAKELEEAALTDD	0	0
Q10530	KLMGFGHRVYKNYDPRARIVK	0	0
Q10530	KLPTIAAYAYKKSVGQPFLYP	0	0
Q10530	LPTIAAYAYKKSVGQPFLYPD	0	0
Q10530	QRHTMLHEDLKRFFDGFPRNA	0	0
Q10530	RGYPIDQLAEKSTFIEVCYLL	0	0
Q10530	RKVKNREAGVKLMGFGHRVYK	0	0
Q10530	VELSTIRLLAKLPTIAAYAYK	0	0
Q10530	VKEQADKILAKLGGDDSLLGI	0	0
Q10530	WREMHDEGDSKIGRPRQIYTG	0	0
Q10530	DGIALGPLLAKTGHTTFDVGF	0	0

Q10530	DVGFANTAAAKSSITYIDGDA	0	0
Q10530	KNYDPRARIVKEQADKILAKL	0	0
Q10530	SGDDVSEFVRKVKNREAGVKL	0	0
Q10682	ARVITTGGSQKAVNADRSGLA	0	0
Q50685	AIACKDPEKKKKLMTDIFALA	0	0
Q50685	AYVLEGGSARKLIEAREKAFW	0	0
Q50685	DFDQVMAAVPKILASGLAPDI	0	0
Q50685	DGNVHMAIACKDPEKKKKLMT	0	0
Q50685	EAREKAFWAAKALGADDIIDT	0	0
Q50685	GLLISFDRMNKVLEVDTANQV	0	0
Q50685	GPPQKPAYAAKPATPEEVAQL	0	0
Q50685	GTNAGGMRAVKYGVARHNVLG	0	0
Q50685	GYFLELEDPVKISLMRRIKQS	0	0
Q50685	HDEELTGPPQKPAYAAKPATP	0	0
Q50685	HMAIACKDPEKKKKLMTDIFA	0	0
Q50685	IACKDPEKKKKLMTDIFALAM	0	0
Q50685	ISGEHGVGRAKTGYFLELEDP	0	0
Q50685	MAIACKDPEKKKKLMTDIFAL	0	0
Q50685	PVKISLMRRIKQSFDPAGILN	0	0
Q50685	ATPEEVAQLLKAASENGVPVT	0	1
Q50685	EIIRTGGRMAKVSTGYDLTQL	0	0
Q50685	SARKLIEAREKAFWAAKALGA	0	0
Q50685	TLALVTEVIVKLHPRLDHNAS	0	0
Q7D8W0	GGSSKDRIAVKMIEAAEASGQ	0	0
Q7D8W0	GPEIWADTEGKVTHFVAGIGT	0	0
Q7D8W0	GTITGAGRYLKEVSGGRVRIV	0	0
Q7D8W0	GYKCVFVCPDKVSEDKRNVLI	0	0
Q7D8W0	IEAAEASGQLKPGGTIVEPTS	0	0
Q7D8W0	LALVAQRRGYKCVFVCPDKVS	0	0
Q7D8W0	LPDGGRGYMSKIFNDAWMSSY	0	0
Q7D8W0	RLVRDIDGAWKPDQYANPEGP	0	0
Q7D8W0	SCGMAVVAALKVAEEAGPDAL	0	0
Q7D8W0	VEYLNPGGSSKDRIAVKMIEA	0	0
Q7D8W0	WDALMVVEEGKPVGVITRYDL	0	0
Q7D8W0	FVCPDKVSEDKRNVLIAYGAE	0	0
Q7D8W0	LLSAVFEGRAKLADAVSAHMS	0	0
Q7D8W0	QSTVGDVLRRKSGALPALVHT	0	0
Q7D8W0	VPDGAGTVAAKVEYLNPGGSS	0	0

Table S4. The 10-fold cross-validation prediction results of PSoL on the training dataset.

Protein Name	Peptide	Class label	Prediction label
A0QNF6	FGEVVDEESQKVVDAIASTPT	1	0
A0QP32	DYFAHWINVGKNADESKLPKV	1	1
A0QP90	AGATRIIGVAKAGLDDAGYRN	1	0
A0QPN2	ANADTVTLWSKLALESARARR	1	1
A0QQ65	NMKYADGTPIKASDFTYAIQR	1	0
A0QQH7	LFDEHGAYLAKTGGEVGVTTG	1	0

A0QQU5	AVEKVTETLLKSAKEVETKEQ	1	1
A0QQU5	VAAGANPLGLKRGIEKAVEKV	1	0
A0QS45	SGEPHKTKVAKVTWDQVREIA	1	0
A0QS98	KALEGDPKWVKSVEELMEAVD	1	0
A0QSE0	KRWRLVEILEKAK________	1	0
A0QSH8	KGTQAEKLSEKLGIPQISTGD	1	1
A0QSL6	SGHVGFKGSRKSTPFAAQLAA	1	1
A0QSU4	KIAQVVEVAAKEPEPSAAIRL	1	1
A0QUV7	GRGVGSAENFKIVEELADSLG	1	1
A0QUX7	QSLAVGATEQKDMSRMTIVVS	1	0
A0QUY3	GQTEAAARVDKAVSEHLATRG	1	1
A0QUY9	RVVADVLRGRKVADGVRMLIV	1	1
A0QUZ0	SFILNLSPFDKGSVLVAGPDF	1	0
A0QV10	ITSIASLETGKRLGPDPRTFN	1	0
A0QVB9	ALVEADGDFDKAVELLRIKGA	1	0
A0QVB9	ANDIETLKAAKTGDTTVEQAI	1	0
A0QVB9	IADLSAKIGEKLELRRAAYFD	1	0
A0QVM0	YSFDVDDDGLKNAYYQHREAY	1	0
A0QW25	DVIDEAATFLKQQEDKGVVVR	1	0
A0QWG8	TFYDDPDVLAKVSRGLGEAMV	1	0
A0QWT3	TFGSETVDPAKIEKAIGEVFD	1	0
A0QWT3	WEQTNKVDDLKSAI_______	1	0
A0QWV9	GAARRLGVSAKAREVRGSDRV	1	1
A0QWW2	SVTDLTAELAKSASVEDINAA	1	1
A0QWX9	KTILQAIELWKIVDRPNLLIK	1	0
A0QWX9	VDTEIDARLEKIGSDEALALR	1	1
A0QX20	ELTDAKNAFRKDIHNYVEQNH	1	0
A0QX81	GGVDVDAVVPKVRPIVDAVAQ	1	1
A0QX93	ATEEEDVLLEKELLADEKERA	1	0
A0QXX7	NSWPDNVSLDKARRLLWPIKQ	1	1
A0QYD4	ARRDDLEVAAKELDIESIVFD	1	0
A0QYS9	DLVARAGVLGKTLSHGIEELG	1	1
A0QZ54	IVYIRTLVTGKSSSASRAIDT	1	0
A0QZA1	SVARTLIDASKSAQMVVVGNR	1	0
A0QZE3	IHIEQGRELEKNNVTIGLVDR	1	0
A0R066	ERKIDVDEWQKKAGAGEITEV	1	0
A0R079	KTSDDIFKLIKDENVEYVDIR	1	0
A0R083	LLAAGLRGVEKGYVLGPQAED	1	0
A0R0B0	DHFFGRDPRTKALVADMSDQE	1	1
A0R0B2	VAAGAVAALDKLAEDPPAKAR	1	0
A0R0B3	AVQTEDKYGVKIPDEDLAGLR	1	1
A0R0B3	TVGDVVAYIQKLEEENPEAAA	1	0
A0R0B4	IRVLEDEFVTKWDLPVRIGGH	1	1
A0R0B5	VELRRLSYLQKMATVLGRRVW	1	1
A0R0W1	RAQVLVPELEKRLDDINAQLD	1	0
A0R0W4	DVADRIAVLNKGRIEQIGSPT	1	0
A0R1B5	AIEVVYGADVKGRGIESAAPL	1	1
A0R1V9	LTAVVGGDLSKVDAKQPDDYF	1	1

A0R1V9	PDDYFTRVTSKDYEGKWRIIF	1	1
A0R1V9	VGGDLSKVDAKQPDDYFTRVT	1	1
A0R1Y7	QSHQKAAAAWKDGVFADEVVP	1	0
A0R218	DVYVSMNMVRKNGLRRGDAVT	1	0
A0R220	NFDDCLELARKLTADFPTIAL	1	0
A0R2G5	NSLSGAVEVVKRDGTGITLNE	1	0
A0R2V7	NAQALADGFVKRDAGLVTGGT	1	0
A0R2W6	IGRQAIEGFYKNMTAATTDIK	1	0
A0R342	HPDIPAAATVKPGSEFRIECK	1	0
A0R3D2	LTANVRTRTGKGASRQARRDG	1	0
A0R3D6	VVVLGPGSIPKTPSGKLRRAN	1	1
A0R4C9	RDFVDAQQFSKLLSERGIAND	1	1
A0R4D0	TGAGIHEVDVKVA________	1	1
A0R4Z5	DEGPRETTLEKMAALQPLVEG	1	1
A0R518	EDLAETVELVKGLNRRIVAEE	1	1
A0R566	MPNSNPVAAWKALKDGNARFV	1	1
A0R5E1	SGFRTLEENQKVEFEVGQSPK	1	0
A0R5M8	FDLDAFEHVAKWNSDEERIEM	1	0
A0R5R5	LSREQATWAQKAIAQEGLTDL	1	0
A0R5R7	EILEDRLGESKIASWTDPKGG	1	1
A0R635	HNETVVLDFPKRVVREPIPDE	1	0
A0R647	_MTNSGALDTKFHALIQDQIR	1	0
A0R652	HHSKHHATYVKGVNDAIAKLE	1	1
A0R652	WKNLSPNGGDKPTGELAAAID	1	0
A0R6E3	PATEAETAQIKDLIAALGG__	1	1
A0R6E3	RAAVVLSGLRKLQGSLAEAAS	1	1
A0R6Q0	IKTADVDAAAKEIEATGGAII	1	0
A0R7F9	VIRKDGGTVDKVDIWGRRRLA	1	0
A0R7G6	NFVAAFDVDAKKVGFDLSEAI	1	1
A4ZHR8	ALGGTLTKLSKDQAEYIGVDV	1	1
EG10025	APFAGTVKEIKVNVGDKVSTG	1	0
EG10207	FADYDEARFQKEGFRVVPPAA	1	0
EG10251	DVKILDPRVGKEFPLPTYATS	1	0
EG10367	SVVDLTVRLEKAATYEQIKAA	1	0
EG10407	QEIFTREEMIKYFTLNAVSKS	1	1
EG10410	LAMQVVVLRDKLEPYFTEGRY	1	0
EG10416	SAEQVDPRDEKVANLEAQLAE	1	0
EG10416	TRERDGILRVKAEMENLRRRT	1	0
EG10489	_______MESKVVVPAQGKKI	1	1
EG10489	WLKVKNPNTGKEIVIKDVIAD	1	1
EG10492	MNDAFWDELLKVRGEVNKVIE	1	0
EG10762	YLRWAESRGFKTEIIEESEGE	1	0
EG10770	XXXDIVEYLVKQIKG______	1	1
EG10770	KSFRVQELAEKLYSELRAQGI	1	1
EG10770	QLGTKYSEALKASVQGEDGRN	1	1
EG10803	EGKVFLNIGDKFLLDANLGKG	1	1
EG10803	RADKVREIAAKLGRHVAILGD	1	1
EG10876	ARAAIEAAGGKIEE_______	1	1

EG10903	AALELAEQREKPTWLEVDAGK	1	1
EG10903	REKAKKQSRVKAALELAEQRE	1	1
EG10905	YTAAITGAEGKIHRLEDWGRR	1	1
EG10954	TYVTNLNNLIKGTAFEGKSLE	1	1
EG11192	GELILNEKLAKQLVTAANWVK	1	1
EG11223	HSWVEGKLIDKVGQLGKKLHT	1	1
EG11415	TQVINSKTPLKSYPLDIHNVQ	1	1
EG11427	AWNEKFAAYAKAYPQEAAEFT	1	1
EG11427	FDAKAKEFIAKLQANPAKIAS	1	1
EG11518	LQLGKDLDRFKNLVLVHAARY	1	1
EG11615	EYALLSRLMLKAGSPVHREIL	1	1
EG11615	LHFLARIRQKKYTLPVLILTA	1	1
EG12204	__MAKIKGQVKWFNESKGFGF	1	0
EG12411	RKAGIPLALNKLNAVPTTAYP	1	1
EG12503	EGVKYLEENAKKEGVNSTESG	1	1
EG12545	PNYLHKEPVIKAAKNKKHVFC	1	1
EG12669	GEIAADFAQDKKSDFDLTPFR	1	1
EG12712	INSNEELALPKEKLQELHI__	1	1
EG12982	________MQKVVLATGNVGK	1	1
EG13014	LITYGGGSVKKTGVLDQVLDA	1	1
EG13952	AGGIYLFTDEKSALAYLEKHT	1	1
EG13952	AYLEKHTARLKNLGVEEVVAK	1	1
O05598	VVVLGPGTIPKTPSGKLRRAN	1	1
O05814	YSFDIDAAGLKAAYHAHREAY	1	1
O06188	SAVEAAGKLVKIETDIPRGPA	1	1
O06391	IAARMTTAVDKARVRGAGHEV	1	1
O33294	LHSRGFSEDPKALEPAVRKVE	1	1
O33341	EKSLDDLRLFKALVDAASGYA	1	1
O53176	FMRNSIDLYHKQAADTGARIV	1	1
O53204	DKGYDGRALIKDLSFSLPRNG	1	1
O53226	TKTTRLTPGDKAPAFTLPDAD	1	1
O53226	YNVKATGHVAKLRRDLSV___	1	1
O53442	QHVMKGYRAEKYTQVETLVYM	1	1
O53618	GILRPKSGAIKFDEVDITTLQ	1	1
O53665	TALVYLSPDAKPAATGLESTM	1	1
O53665	TQALAPGLAAKGITINAVAPG	1	1
O53871	AEAWSGGYFAKSVVPVRDQNG	1	1
O69687	IGATLSAEQAKWGQKAVEDEG	1	1
O86352	VGLSRGWSLIKSVRLGNAAGA	1	1
P09621	SARDVLAVVSK__________	1	1
P0A508	EWQLRVGAGEKLGFAQNDIEL	1	1
P0A520	AVEKVTETLLKGAKEVETKEQ	1	0
P0A556	PAKEDEEVVRKATTDEPFAAL	1	1
P0A5B7	LPVGADEDDIKATYDKGILTV	1	1
P0A5B7	LTIKAERTEQKDFDGRSEFAY	1	1
P0A5B7	LTVSVAVSEGKPTEKHIQIRS	1	1
P0A5B7	RAELPGVDPDKDVDIMVRDGQ	1	1
P0A5F7	QAHQIVEQAHKVALEASSSGR	1	1

P0A5H3	LDDATIAKFQKELAAMGFKFQ	1	1
P0A5L2	VARITAPDEPKYDADFAAVRQ	1	1
P0A5U4	ENADVADEIEKKIKEKLGIGA	1	1
P0A5Z4	HVQRLRAKVEKDPENPTVVLT	1	1
P0CG95	SGAGIHEVDVKIT________	1	1
P0CG99	IPDITFELAKKNEDMYLFSPY	1	1
P0CH36	ADVTVLSQSLKKMEDGLRLGA	1	1
P17670	SRYAAATSQTKGLIFG_____	1	1
P60176	ALGGHLTKLTKEQAEYLGVDV	1	1
P60796	TFFDDPDVLAKVSRGLGEAMV	1	1
P63458	VAAGRLTALEKLAEDPPAKAR	1	1
P63523	KPEVVYFEPDKLSTGSANRCE	1	1
P63568	GLVRRAEVLGKSLRHGIEALG	1	1
P63673	SQKLTEEAADKLTEVIKNFKK	1	1
P65161	ESLAVGATECKDRSRMTIVVS	1	1
P65232	AHPELAEQVAKELDVHVTSQD	1	1
P65277	ITAATVVLPFKIDDHSAWRLL	1	1
P65573	DVLARLEVDAKEIIGRYPDRR	1	1
P65880	LFDEHGEYLSKTGREFGVTTG	1	0
P66056	SAEPHKTKVAKVTWDQVREIA	1	1
P66902	NFDDCLELARKMAADFPTISL	1	1
P69440	DGYPRSVEQAKALHEMLERRG	1	1
P71703	KFVAAFDVDAKKVGFDLSDAI	1	1
P71724	QAVAAAEVLGKRQPLLIVSSD	1	1
P71973	AIIDDASTFFKQQEERGVVVR	1	1
P77899	TFGTETEDPVKIEKAIGEVFD	1	1
P96382	DGKLGAFVEVKNSTIGTGTKV	1	1
P96825	WAEITDLSGAKIAGFKL____	1	1
Q10504	DHFFGRDPRTKALVENMSDQD	1	1
Q10530	RARIVKEQADKILAKLGGDDS	1	1
Q10682	VVAVGFTFDPKTHIARVITTG	1	1
Q50685	DTVVPRASMPKFLSTARGLAA	1	1
Q7D8W0	GAGELVSAAGKALRDWDALMV	1	1
A0QNF6	ATLHTNRGDIKIALFGNHAPK	0	0
A0QNF6	DEFHPELQFDKPYLLAMANAG	0	0
A0QNF6	KIALFGNHAPKTVNNFVGLAQ	0	0
A0QNF6	NGSQFFITVGKTPHLNRRHTI	0	0
A0QNF6	NNFVGLAQGTKDYSTENASGG	0	1
A0QNF6	TGTGRGGPGYKFEDEFHPELQ	0	0
A0QP32	AGTFQKLNPEKQPNSYLALSD	0	0
A0QP32	AHLVEAGTFQKLNPEKQPNSY	0	0
A0QP32	DKLGDDGFFVKALHSIGAPLE	0	0
A0QP32	EGDPQHLIDWKGNDWTPESGE	0	0
A0QP32	FVGEKLPTGLKDEFDALKHRL	0	0
A0QP32	GDDIAWMRFGKDGRLYATNPE	0	0
A0QP32	GKNADESKLPKVFFVNWFRRG	0	0
A0QP32	GSEQTAAAEGKVGTVRRDPMA	0	0
A0QP32	GWLAEHMLILKLISPENKAYF	0	0

A0QP32	GYGGNALLGKKCYSLRIASAM	0	0
A0QP32	IAAAFPSACGKTNLAMLQPTI	0	0
A0QP32	INVGKNADESKLPKVFFVNWF	0	1
A0QP32	ISAILFGGRRKTTVPLITEAR	0	1
A0QP32	KDVPWPCNDTKYITHFPETRE	0	0
A0QP32	LILKLISPENKAYFIAAAFPS	0	0
A0QP32	LKWAVERIEHKADGKSTPIGI	0	0
A0QP32	MTRMGRAALDKLGDDGFFVKA	0	0
A0QP32	PADVDEALAVKPEEWRAELPL	0	0
A0QP32	PGFGENSRVLKWAVERIEHKA	0	0
A0QP32	SGYGGNALLGKKCYSLRIASA	0	0
A0QP32	TGLKDEFDALKHRLSEEG___	0	0
A0QP32	TNWSSNPNAMKTIAAGNTVFT	0	0
A0QP32	VERIEHKADGKSTPIGIVPTA	0	0
A0QP32	AGNTVFTNVAKTDDGDVWWEG	0	1
A0QP32	CMGPLDAEDPKLGVEITDSEY	0	1
A0QP32	GNDWTPESGEKAAHPNSRYCT	0	0
A0QP32	IEEWFEFVGEKLPTGLKDEFD	0	0
A0QP32	SIGAPLEPGQKDVPWPCNDTK	0	1
A0QP32	TIPGLDTAPTKHQGLLAWVQE	0	0
A0QP90	AGVPFYLRTGKRMSQRFSEIV	0	1
A0QP90	DEAIRLEMTAKEPGSGGRLRP	0	0
A0QP90	HVNRESVRDEKRKVLEALEPL	0	0
A0QP90	NRESVRDEKRKVLEALEPLTA	0	0
A0QP90	QIFRIDHYLGKESVQQLLVTR	0	0
A0QP90	QRFSEIVVQFKPVPLPMFPGI	0	0
A0QP90	VTESSRVVLEKPIGRDLASAQ	0	0
A0QP90	FGGTGDLAARKLLPALYLRDR	0	1
A0QP90	HSRTETFVAVKAHIRNWRWAG	0	1
A0QPN2	APDVAWDSQAKTAVSSRSLNL	0	0
A0QPN2	ARAGGKFSAHKDATSTGAGLN	0	0
A0QPN2	ARARRSAEGAKARARAASKAK	0	0
A0QPN2	ASRMRDLGLAKNEEGPEPQDF	0	0
A0QPN2	DATFQAAFPLKGKPPNVYGFP	0	0
A0QPN2	DPTTGKNGIVKTLGTARAGGK	0	0
A0QPN2	EAPFTRNDTQKLRGVSNRKPD	0	0
A0QPN2	GAKARARAASKAKGLGTNLSL	0	0
A0QPN2	GLGTNLSLPPKLLPSRESGRG	0	0
A0QPN2	GRGYPGVFEGKEFDPEAPFTR	0	0
A0QPN2	IRVRPDESEKKLHHTLFGGPP	0	0
A0QPN2	KARARAASKAKGLGTNLSLPP	0	0
A0QPN2	KTLGTARAGGKFSAHKDATST	0	0
A0QPN2	LPVDTDPTTGKNGIVKTLGTA	0	0
A0QPN2	PPNVYGFPLNKARAKDEFDAI	0	0
A0QPN2	RTDVTVRRDGKTFLQSFGRGY	0	0
A0QPN2	SNRRVEVQRNKGLGEMDADDF	0	0
A0QPN2	TFQAAFPLKGKPPNVYGFPLN	0	0
A0QPN2	TQKLRGVSNRKPDLHGTEVRI	0	0

A0QPN2	VIRVRPDESEKKLHHTLFGGP	0	1
A0QPN2	YVTMPPLFVVKAGDERIYCQD	0	0
A0QPN2	CEGDSALGTIKAARDATFQAA	0	1
A0QPN2	GGGSHLTAAIKGLSEALAERA	0	1
A0QPN2	SERDAAVAQLKASSNRRVEVQ	0	0
A0QPN2	YGFPLNKARAKDEFDAIERIL	0	1
A0QQ65	ADEVADAHVDKIIVTQNKSNS	0	0
A0QQ65	DDFFRPLINGKSILPTNGNNF	0	0
A0QQ65	EDMPEVSPDGKTYKLKLRQNM	0	0
A0QQ65	FNQDFTSFALK__________	0	0
A0QQ65	FTLYPGPDMDKAKQLIAEANP	0	0
A0QQ65	HVDKIIVTQNKSNSAQVTDIE	0	0
A0QQ65	IVGANDYAEGKADTITGIQTN	0	0
A0QQ65	LTQLGFNATLKVIAGDVYWTT	0	0
A0QQ65	LYPGPDMDKAKQLIAEANPAD	0	0
A0QQ65	MERNPQFQTVKDAGADEVADA	0	0
A0QQ65	_____MIAGRKCLKIASVAAV	0	0
A0QQ65	__MIAGRKCLKIASVAAVAVL	0	0
A0QQ65	PEVSPDGKTYKLKLRQNMKYA	0	0
A0QQ65	SAQVTDIEQNKTDFMHDPPDA	0	0
A0QQ65	TYKLKLRQNMKYADGTPIKAS	0	0
A0QQ65	VSPDGKTYKLKLRQNMKYADG	0	0
A0QQ65	YNTYVPLLTYKHAKGEEGAEV	0	0
A0QQ65	YVPLLTYKHAKGEEGAEVAPG	0	0
A0QQ65	DFTYAIQRLFKADSGGSVFFG	0	1
A0QQ65	LLTQQLTDETKKGYADLDRAY	0	0
A0QQ65	LTQQLTDETKKGYADLDRAYM	0	0
A0QQ65	PPDADRLPEVKARFGDRFRLE	0	1
A0QQ65	SRVAIPELDAKMNELLTQQLT	0	0
A0QQ65	TVWTDDEPDRKRIGEYYHDVL	0	0
A0QQH7	AEKIEAALEFKNQVLVKIYNR	0	0
A0QQH7	ALEFKNQVLVKIYNRKALEPA	0	0
A0QQH7	DKVVERWAGSKKIGTTGRGIG	0	0
A0QQH7	KVVERWAGSKKIGTTGRGIGP	0	0
A0QQH7	LLMPYHVAIDKVVERWAGSKK	0	0
A0QQH7	NQVLVKIYNRKALEPAEVLEN	0	0
A0QQH7	PVCVGYTVDGKRVDEMPMTQS	0	0
A0QQH7	TRITTVLGILKAYTTRVGSGP	0	0
A0QQH7	VDPGVLLTELKGLEDRGVDTS	0	0
A0QQH7	VNGITDYFLTKLDVLSSLETV	0	0
A0QQH7	DVLDEQVLAEKIEAALEFKNQ	0	1
A0QQH7	ENLLEQAEGFKHRIADARLLL	0	1
A0QQH7	GAREFEDLPAKARDYVLRLEE	0	1
A0QQU5	AATEVELKERKHRIEDAVRNA	0	0
A0QQU5	AKLAGGVAVIKAGAATEVELK	0	0
A0QQU5	EDPYILLVSSKVSTVKDLLPL	0	0
A0QQU5	ENSDSDYDREKLQERLAKLAG	0	0
A0QQU5	FLTTEAVVADKPEKAAAPAGD	0	0

A0QQU5	IRGTFKSVAVKAPGFGDRRKA	0	0
A0QQU5	IVRVALSAPLKQIALNGGLEP	0	0
A0QQU5	KAGAATEVELKERKHRIEDAV	0	0
A0QQU5	KEIELEDPYEKIGAELVKEVA	0	0
A0QQU5	LELTEGMRFDKGYISGYFVTD	0	0
A0QQU5	LKRGIEKAVEKVTETLLKSAK	0	0
A0QQU5	LLAAGVADPVKVTRSALQNAA	0	0
A0QQU5	________MAKTIAYDEEARR	0	0
A0QQU5	NPLGLKRGIEKAVEKVTETLL	0	0
A0QQU5	PLLEKVIQSGKPLLIIAEDVE	0	0
A0QQU5	TEAVVADKPEKAAAPAGDPTG	0	0
A0QQU5	TITNDGVSIAKEIELEDPYEK	0	0
A0QQU5	TVKDLLPLLEKVIQSGKPLLI	0	0
A0QQU5	VKAPGFGDRRKAMLQDMAILT	0	0
A0QQU5	ADAVKVTLGPKGRNVVLEKKW	0	1
A0QQU5	ADVSLLGKARKVVVTKDETTI	0	0
A0QQU5	DREKLQERLAKLAGGVAVIKA	0	0
A0QQU5	GAELVKEVAKKTDDVAGDGTT	0	0
A0QQU5	GEALSTLVVNKIRGTFKSVAV	0	1
A0QQU5	GGLEPGVVAEKVSNLPAGHGL	0	1
A0QQU5	GPKGRNVVLEKKWGAPTITND	0	0
A0QQU5	HRIEDAVRNAKAAVEEGIVAG	0	0
A0QQU5	IGAELVKEVAKKTDDVAGDGT	0	0
A0QQU5	IGDLIAEAMDKVGNEGVITVE	0	0
A0QQU5	KVTETLLKSAKEVETKEQIAA	0	0
A0QQU5	LETADVSLLGKARKVVVTKDE	0	0
A0QQU5	LGKARKVVVTKDETTIVEGAG	0	1
A0QQU5	LLKSAKEVETKEQIAATAGIS	0	1
A0QQU5	LLVSSKVSTVKDLLPLLEKVI	0	0
A0QQU5	LVVNKIRGTFKSVAVKAPGFG	0	0
A0QQU5	PKGRNVVLEKKWGAPTITNDG	0	0
A0QQU5	PYEKIGAELVKEVAKKTDDVA	0	0
A0QQU5	RGLNSLADAVKVTLGPKGRNV	0	1
A0QS45	APKKKVAGLIKLQIQAGQANP	0	0
A0QS45	DQVREIAETKKADLNANDIDA	0	0
A0QS45	GVQKGSGEPHKTKVAKVTWDQ	0	0
A0QS45	KLLLKAAGVQKGSGEPHKTKV	0	0
A0QS45	KTPPAAKLLLKAAGVQKGSGE	0	0
A0QS45	LNANDIDAAAKIIAGTARSMG	0	0
A0QS45	_____MAPKKKVAGLIKLQIQ	0	0
A0QS45	QHGVNIMEFCKAYNAATESQR	0	0
A0QS45	TFALKTPPAAKLLLKAAGVQK	0	0
A0QS45	WDQVREIAETKKADLNANDID	0	0
A0QS45	YEDRSFTFALKTPPAAKLLLK	0	0
A0QS45	QKGSGEPHKTKVAKVTWDQVR	0	1
A0QS98	EAPVVRVSALKALEGDPKWVK	0	1
A0QS98	EDVERGQVVVKPGTTTPHTEF	0	1
A0QS98	EFEGQVYILSKDEGGRHTPFF	0	0

A0QS98	EIVGIRPETTKTTVTGVEMFR	0	0
A0QS98	GKTTLTAAITKVLHDKFPDLN	0	0
A0QS98	IGTIGHVDHGKTTLTAAITKV	0	0
A0QS98	________MAKAKFERTKPHV	0	0
A0QS98	______MAKAKFERTKPHVNI	0	0
A0QS98	_MAKAKFERTKPHVNIGTIGH	0	0
A0QS98	MPGDNTDISVKLIQPVAMDEG	0	0
A0QS98	NISHVEYQTDKRHYAHVDAPG	0	0
A0QS98	SALKALEGDPKWVKSVEELME	0	0
A0QS98	SIPDPVRETDKPFLMPVEDVF	0	0
A0QS98	TAAITKVLHDKFPDLNESRAF	0	0
A0QS98	TTVTGVEMFRKLLDQGQAGDN	0	0
A0QS98	VDAPGHADYIKNMITGAAQMD	0	0
A0QS98	DNVGLLLRGIKREDVERGQVV	0	1
A0QS98	GRTVGAGRVTKIIK_______	0	0
A0QS98	GVPYILVALNKSDAVDDEELI	0	1
A0QS98	VGAGRVTKIIK__________	0	1
A0QSE0	EDRKSHPLYGKIIRTTKKVKA	0	0
A0QSE0	GKIIRTTKKVKAHDENGEAGI	0	0
A0QSE0	KGPKYTPAAEKPRGRRKTAIG	0	0
A0QSE0	KTIVVELEDRKSHPLYGKIIR	0	0
A0QSE0	LMETRPLSATKRWRLVEILEK	0	0
A0QSE0	LYGKIIRTTKKVKAHDENGEA	0	0
A0QSE0	______MADQKGPKYTPAAEK	0	0
A0QSE0	___MADQKGPKYTPAAEKPRG	0	1
A0QSE0	PAAEKPRGRRKTAIGYVVSDK	0	0
A0QSE0	PLYGKIIRTTKKVKAHDENGE	0	1
A0QSE0	IGYVVSDKMQKTIVVELEDRK	0	0
A0QSE0	KTAIGYVVSDKMQKTIVVELE	0	0
A0QSE0	WRLVEILEKAK__________	0	0
A0QSH8	DTDEVIRNRMKVYREETEPLL	0	0
A0QSH8	GDGTPLGLEAKRYLDAGDLVP	0	0
A0QSH8	LKDMLAARNTKLDAVLEFQVS	0	0
A0QSH8	PLLEYYRDDLKTVNAVGALDE	0	0
A0QSH8	PQISTGDLFRKNIGDGTPLGL	0	0
A0QSH8	PRSVEQAGALKDMLAARNTKL	0	0
A0QSH8	VSEDELLTRLKGRGRADDTDE	0	0
A0QSH8	VVLLGPPGAGKGTQAEKLSEK	0	0
A0QSH8	PGAGKGTQAEKLSEKLGIPQI	0	1
A0QSL6	AQAKKGGTAAKKGQKTRRREK	0	0
A0QSL6	ARKAQEHGVKKVDVFVKGPGS	0	0
A0QSL6	HGVKKVDVFVKGPGSGRETAI	0	0
A0QSL6	KGGTAAKKGQKTRRREKKNVP	0	0
A0QSL6	KGQKTRRREKKNVPHGAAHIK	0	0
A0QSL6	KKGQKTRRREKKNVPHGAAHI	0	0
A0QSL6	______MAQAKKGGTAAKKGQ	0	0
A0QSL6	_____MAQAKKGGTAAKKGQK	0	0
A0QSL6	QAKKGGTAAKKGQKTRRREKK	0	0

A0QSL6	AARKAQEHGVKKVDVFVKGPG	0	1
A0QSL6	AQLAAENAARKAQEHGVKKVD	0	0
A0QSL6	AWASSGHVGFKGSRKSTPFAA	0	0
A0QSL6	KNVPHGAAHIKSTFNNTIVSI	0	0
A0QSL6	PQPHNGCRPPKRRRV______	0	0
A0QSU4	AGYCDLKEFQKVGLTVGS___	0	0
A0QSU4	GDIHSSGDLAKAIACGADAVV	0	0
A0QSU4	IVPSRRTRSSKDVSTAWQLDA	0	0
A0QSU4	LVGGLRRSMAKAGYCDLKEFQ	0	0
A0QSU4	SMAKAGYCDLKEFQKVGLTVG	0	0
A0QSU4	VASDGEPLNLKTFISELDVPV	0	0
A0QSU4	LIGRHADVEEKIAQVVEVAAK	0	1
A0QUV7	AQAENATRITKREPAVAGDRP	0	0
A0QUV7	GTAAGLTDGLKAAGAAKIYVA	0	0
A0QUV7	GVIIAANADGKEIAGRLAARI	0	0
A0QUV7	IQHRAGMQTSKTIVAVNKDEE	0	0
A0QUV7	LGEPSAVVVGKPGTAAGLTDG	0	0
A0QUV7	MLVEHADGALKKVSAELLTAA	0	0
A0QUV7	PGQFQVGQTGKTVSPQLYIAL	0	0
A0QUV7	QTSKTIVAVNKDEEAPIFEIA	0	0
A0QUV7	ADLGIVGDLFKVSPQLTEAVK	0	1
A0QUV7	KVSPQLTEAVKARKG______	0	1
A0QUV7	LVEHADGALKKVSAELLTAAR	0	0
A0QUV7	PQLTEAVKARKG_________	0	0
A0QUV7	SGLLVDVIEVKEGGVGVHSIF	0	0
A0QUV7	TDGLKAAGAAKIYVAESDDVE	0	0
A0QUX7	KQLNKLINVIKIVEQEEDNSV	0	1
A0QUX7	LTIEATGTPEKLEALLRVLEP	0	0
A0QUX7	NSVSRELALIKVRADATTRGQ	0	0
A0QUX7	THTLSVLVEDKPGVLARVSSL	0	1
A0QUX7	IIEAVNLFRAKVVDVSTESLT	0	0
A0QUX7	PLEQITKQLNKLINVIKIVEQ	0	0
A0QUX7	SRGPRGIGAAK__________	0	0
A0QUX7	VEDSPLEQITKQLNKLINVIK	0	1
A0QUY3	DAFARAQDRRKHLTLVHKTNV	0	0
A0QUY3	DRRKHLTLVHKTNVLAFAGSL	0	0
A0QUY3	_____MSSNLKLAVIAGDGIG	0	0
A0QUY3	SEHLATRGDEKLSTSAVGERI	0	0
A0QUY3	SGVLERGMLLKLRFALDHHVN	0	0
A0QUY3	VLDAVLPGVEKTEYDLGARRY	0	0
A0QUY9	APDETTFEFLKGRPHAPKGAD	0	0
A0QUY9	ATQTLSLKPFKTMAVNVDGEL	0	0
A0QUY9	AVAAATAVRGKLASPADLPAA	0	0
A0QUY9	DGELPPGVSAKDIILAVIAKI	0	0
A0QUY9	DHNVPTIDIDKPIADPVSRTQ	0	0
A0QUY9	EFLKGRPHAPKGADWDAAVAA	0	0
A0QUY9	HVMATQTLSLKPFKTMAVNVD	0	0
A0QUY9	_MDQPTQTSVKPRTLAEKVWD	0	0

A0QUY9	SNRNFEGRQGKGGRTHLVSPA	0	0
A0QUY9	TSVKPRTLAEKVWDDHVVARG	0	0
A0QUY9	AKDIILAVIAKIGTGGGQGHV	0	1
A0QUY9	VARGEGEGTAKEPDLIYIDLH	0	1
A0QUY9	VDEGARQAAEKALAYMDLRPG	0	1
A0QUZ0	EAYEAARPAWKPRTLPA____	0	0
A0QUZ0	GLDDIGLTLRKLDQIEAYEAA	0	0
A0QUZ0	TDQIIPAVYLKRVTRTGFEDG	0	1
A0QUZ0	FADIFRGNAGKAGLLAAEVAQ	0	0
A0QUZ0	VAQDDVELLWKVIEQNPGLEI	0	0
A0QV10	HIQLGNIVIPKSVNPERIASN	0	0
A0QV10	LIHWPVPENNKFVDTFKAFAH	0	0
A0QV10	PENNKFVDTFKAFAHLRDQGR	0	0
A0QV10	PQQELRDVHAKLGIATEAWSP	0	0
A0QV10	VITGIAEQHGKTPAQVLIRWH	0	0
A0QV10	VPREDIFLVTKLWNSDQGYDA	0	0
A0QVB9	AAKANDIETLKAAKTGDTTVE	0	0
A0QVB9	AVELLRIKGAKDVGKRAERAT	0	0
A0QVB9	DQIVAAAVAAKANDIETLKAA	0	0
A0QVB9	ELNSETDFVAKNAEFQALADQ	0	0
A0QVB9	ELTGAGMLDSKNALVEADGDF	0	0
A0QVB9	EYQAGDADKGKEAAHAVALQI	0	0
A0QVB9	IAEETARNEGKPEQALPKIVE	0	1
A0QVB9	IVEGRVTGFYKDVVLLDQPSV	0	0
A0QVB9	LDQPSVSDNKKTVKALLDEAG	0	0
A0QVB9	LLDQPSVSDNKKTVKALLDEA	0	0
A0QVB9	LRIKGAKDVGKRAERATAEGL	0	0
A0QVB9	LVEYQAGDADKGKEAAHAVAL	0	0
A0QVB9	_MANYTAADVKRLRELTGAGM	0	0
A0QVB9	NEGKPEQALPKIVEGRVTGFY	0	0
A0QVB9	PSVSDNKKTVKALLDEAGVTV	0	0
A0QVB9	FDGTVEAYLHKRAADLPPAVG	0	1
A0QVB9	FDKAVELLRIKGAKDVGKRAE	0	0
A0QVB9	HAVALQIAALKAKYLTREDVP	0	1
A0QVB9	RATAEGLVAAKDGALIELNSE	0	1
A0QVB9	VALQIAALKAKYLTREDVPED	0	1
A0QVB9	VEQAIADLSAKIGEKLELRRA	0	1
A0QVM0	AADTLKNVLLKTREPGGEWEL	0	0
A0QVM0	ADFAANPFLVKGYVGPKALQD	0	0
A0QVM0	DPADAEVPSHKLLIRAGYVRA	0	0
A0QVM0	EIGHIFQLGRKYTDAFSADVL	0	1
A0QVM0	FDDRQASPGVKFKDAELLGMP	0	0
A0QVM0	FPVILYQIQTKYRDEARPRAG	0	0
A0QVM0	FSADVLGEDGKPLRLTMGSYG	0	0
A0QVM0	GILRGREFVMKDSYSFDVDDD	0	0
A0QVM0	GRTVTAADTLKNVLLKTREPG	0	0
A0QVM0	GVPGDREVDEKRLGAALEPAE	0	0
A0QVM0	LTVKGEYSSYKDFPVILYQIQ	0	0

A0QVM0	PFLVKGYVGPKALQDNGVRYL	0	0
A0QVM0	AQPTEGLPEAKVYDTPDTPTI	0	1
A0QVM0	PFDVHVVVANKDAGARAGAAE	0	1
A0QVM0	WLPLGLRVLRKIENVVRSEMN	0	0
A0QW25	________MAKLDFDALNSTI	0	0
A0QW25	SEHGMAARGYKDVRANTVPAF	0	0
A0QW25	VALHRPAEFNKSHIPAFLAGE	0	0
A0QW25	AATFLKQQEDKGVVVRGLYDV	0	1
A0QW25	PRISVENLIAKLP________	0	1
A0QWG8	ADYTNHIDKWKFTVPFVCGAT	0	0
A0QWG8	DVSNATTHMRKIGGEIRRLTS	0	0
A0QWG8	EGVFVGSGIFKSGNPEQRAAA	0	0
A0QWG8	IDAVTIPVMAKARIGHFVEAQ	0	0
A0QWG8	ITEGAAMIRSKGEAGTGDVSN	0	0
A0QWG8	MSEDELYVAAKELQAPYELVV	0	0
A0QWG8	NPEQRAAAIVKATTFYDDPDV	0	0
A0QWG8	RVKRGMAEMLKGGVIMDVVTP	0	0
A0QWG8	SSNQTGTARVKRGMAEMLKGG	0	0
A0QWG8	TPADYTNHIDKWKFTVPFVCG	0	1
A0QWG8	ELVVEVARAGKLPVTLFTAGG	0	1
A0QWT3	ARHGGGAFSGKDPSKVDRSAA	0	0
A0QWT3	DIELPWEQTNKVDDLKSAI__	0	0
A0QWT3	DTAHETRVEGKADPLDLQGAG	0	0
A0QWT3	DYRLLVNPTGKFVLGGPMGDA	0	1
A0QWT3	GGAFSGKDPSKVDRSAAYAMR	0	0
A0QWT3	GVLDYLRPDGKTQVTIQYDGT	0	0
A0QWT3	ILDIGYDSSTKGFDGASCGVN	0	0
A0QWT3	PMGDAGLTGRKIIVDTYGGWA	0	0
A0QWT3	RLARRLTEVRKNGVLDYLRPD	0	0
A0QWT3	SAAYAMRWVAKNVVAAGLAER	0	0
A0QWT3	SESVTEGHPDKICDAISDSVL	0	0
A0QWT3	VEVQVAYAIGKAAPVGLFVET	0	0
A0QWT3	EGTLTPDIREKVVNTVLADLG	0	1
A0QWT3	________MSKGRLFTSESVT	0	1
A0QWT3	SETVDPAKIEKAIGEVFDLRP	0	1
A0QWT3	VLDALLEQDPKSRVAVETLVT	0	0
A0QWT3	VTTTAYADIPKIVRDRILDIG	0	1
A0QWV9	DLGIIARRLRKDIYDLYGYNA	0	0
A0QWV9	IRRLLSMADRKAKQEGIPDTE	0	0
A0QWV9	LGRLADPPMTKDAVAGRIRRL	0	0
A0QWV9	_____MTAEVKDELSRLVVNS	0	0
A0QWV9	RLLSMADRKAKQEGIPDTESA	0	0
A0QWV9	SVPDHLAAAGKLRVEHRQASL	0	1
A0QWV9	VHVLSASGIRKNTRYVVRVAN	0	1
A0QWW2	AAEGPLKGILKYYDAPIVSSD	0	0
A0QWW2	AKGHLDAGAKKVIISAPATDE	0	0
A0QWW2	AMKAAAEGPLKGILKYYDAPI	0	0
A0QWW2	ASVEDINAAMKAAAEGPLKGI	0	0

A0QWW2	CTTNCLGPLAKVLNDEFGIVK	0	0
A0QWW2	DTIVIGDTKIKALEVKEGPAA	0	0
A0QWW2	ESTGIFTNAAKAKGHLDAGAK	0	0
A0QWW2	GDDTIVIGDTKIKALEVKEGP	0	0
A0QWW2	GDTKIKALEVKEGPAALPWGD	0	0
A0QWW2	GLTKVIDNQAKVVSWYDNEWG	0	0
A0QWW2	IGLVLPELKGKLDGYALRVPI	0	0
A0QWW2	ITIVLGVNDDKYDGSQNIISN	0	0
A0QWW2	KAIGLVLPELKGKLDGYALRV	0	0
A0QWW2	KAKGHLDAGAKKVIISAPATD	0	0
A0QWW2	KVLNDEFGIVKGLMTTIHAYT	0	0
A0QWW2	NIVPTSTGAAKAIGLVLPELK	0	0
A0QWW2	RALATQKAEGKNTDIEIVAVN	0	0
A0QWW2	RLADLVALVGKSL________	0	0
A0QWW2	RNFYRALATQKAEGKNTDIEI	0	1
A0QWW2	TDNATLAHLLKFDSILGRLPQ	0	0
A0QWW2	TGIFTNAAKAKGHLDAGAKKV	0	0
A0QWW2	HSSLYDAGLTKVIDNQAKVVS	0	1
A0QWW2	QDQNLQDGPHKDLRRARAAAL	0	1
A0QWX9	APNTVNTMPEKTLEAVADHGE	0	0
A0QWX9	DAYLEGLEKAKEAGHDLSKIH	0	0
A0QWX9	DVRNACDLLAKEYEASDGVDG	0	0
A0QWX9	EEVFGSDRFAKLKADGARVQR	0	0
A0QWX9	GSDEALALRGKAGVANARLAY	0	0
A0QWX9	KAKEAGHDLSKIHSVASFFVS	0	0
A0QWX9	KIVDRPNLLIKIPATMAGLPA	0	1
A0QWX9	NPSIFQAALSKGTAYDAQVNE	0	0
A0QWX9	VDPRLAHDTDKTILQAIELWK	0	0
A0QWX9	VFGSDRFAKLKADGARVQRPL	0	0
A0QWX9	VMDAYLEGLEKAKEAGHDLSK	0	0
A0QWX9	ATQGQLDAAKK__________	0	1
A0QWX9	DATQGQLDAAKK_________	0	1
A0QWX9	FRVLEDEGVEKFEKSWQELLD	0	1
A0QWX9	LEDEGVEKFEKSWQELLDATQ	0	1
A0QWX9	QRPLWASTGVKNPEYSDTLYV	0	1
A0QWX9	TAASSQETFDKLAAIGIDLPD	0	1
A0QX20	AALLAKKAVEKGLTTKPWVKT	0	0
A0QX20	AGIPLVVLGGKEYGSGSSRDW	0	0
A0QX20	AVAALGGDPNKVNPLAPAELV	0	0
A0QX20	DMLRRHGVVGKFVEFYGKGVA	0	0
A0QX20	EKGLTTKPWVKTNMAPGSQVV	0	0
A0QX20	EQLALVEAYAKAQGMWHDPER	0	0
A0QX20	EWDPASTYVRKAPYFDGMPAE	0	0
A0QX20	FIYDASENYKKAGIPLVVLGG	0	0
A0QX20	FPAGESAASLKLDGTETYDIE	0	0
A0QX20	GSGSSRDWAAKGTVLLGVKAV	0	0
A0QX20	GSQVVTDYYNKAGLWPYLEKL	0	0
A0QX20	HVTATKEDGSKVEFDAVVRID	0	1

A0QX20	IEELNSGKTPKTVHVTATKED	0	0
A0QX20	IEGIEELNSGKTPKTVHVTAT	0	0
A0QX20	ILQYVLRNMLKSSK_______	0	1
A0QX20	KKAVEKGLTTKPWVKTNMAPG	0	1
A0QX20	LRTEDGANITKDHIEAIANWD	0	0
A0QX20	MFTESYADVFKGDDRWRSLPT	0	0
A0QX20	___MSSENTGKSSLNSFGARD	0	0
A0QX20	NFEGRISPDVKMNYLASPPLV	0	0
A0QX20	NKAGLWPYLEKLGYYLGGYGC	0	0
A0QX20	PAEPEPVSDIKGARVLALLGD	0	0
A0QX20	PGTEKLPYSLKVLAENLLRTE	0	0
A0QX20	QYLDANGVERKDYNSLGSRRG	0	1
A0QX20	RWGQGAFDDFKVVPPGTGIVH	0	0
A0QX20	SMLIPRVVGFKLSGEIKPGVT	0	0
A0QX20	STVVPSISGPKRPQDRIELTD	0	0
A0QX20	SVMLGAALLAKKAVEKGLTTK	0	1
A0QX20	TDHISPAGAIKPGTPAAQYLD	0	0
A0QX20	TPKTVHVTATKEDGSKVEFDA	0	0
A0QX20	VMLGAALLAKKAVEKGLTTKP	0	0
A0QX20	VVGFKLSGEIKPGVTATDVVL	0	0
A0QX20	VVGKFVEFYGKGVAEVPLANR	0	0
A0QX20	YRLNAVPGTEKLPYSLKVLAE	0	0
A0QX20	YVLRNMLKSSK__________	0	0
A0QX20	AAKGTVLLGVKAVITESFERI	0	1
A0QX20	AFIYDASENYKKAGIPLVVLG	0	1
A0QX20	NTGPLPEEISKAINDNDLAVT	0	1
A0QX20	PQDRIELTDAKNAFRKDIHNY	0	1
A0QX20	VEQNHPTPETKLDEAVEESFP	0	1
A0QX81	_____MNMMAKFQMSRIDLRN	0	0
A0QX81	DVSGHVITLSKAEDLPAHGEA	0	1
A0QX81	GPGNIYVTAAKRICRSQVGID	0	1
A0QX81	HVVEYDEAALKDVSGHVITLS	0	1
A0QX81	RELANQLATTKHVERVTAALS	0	1
A0QX81	VERVTAALSGKQSAIVLVDDI	0	1
A0QX93	DALRATLDLLKTEAMAGLPPL	0	0
A0QX93	DAVARLDVMTKALGQPLTSAV	0	0
A0QX93	DSETPLSAYRKLAANRPGTFL	0	0
A0QX93	FPAGTLSGAPKVRAMELIEEV	0	0
A0QX93	RAMELIEEVEKTRRGLYGGVV	0	1
A0QX93	TMEEYTEIVDKLVGDIEAGEA	0	0
A0QX93	GPYEYTEAANKARAVLNAIAA	0	1
A0QX93	GSSPEALVTVKDGRATTHPIA	0	1
A0QX93	LLEKELLADEKERAEHLMLVD	0	1
A0QX93	STVTGELAEDKTALDAVTACF	0	0
A0QX93	TEHRVVPVIRKVLADSETPLS	0	0
A0QXX7	ALIVGGHTFGKTHGAGDASLV	0	0
A0QXX7	APIEQQGLGWKSSYGTGKGPD	0	0
A0QXX7	ARRLLWPIKQKYGNKISWADL	0	0

A0QXX7	ASAASYRNTDKRGGANGARVR	0	0
A0QXX7	DFNASASGGKKVSLADLIVLA	0	0
A0QXX7	DKARRLLWPIKQKYGNKISWA	0	0
A0QXX7	DSGLSTGQLIKTAWASAASYR	0	0
A0QXX7	EDFDYAEEFAKLDVNALKADL	0	0
A0QXX7	EFAKLDVNALKADLTALMTQS	0	0
A0QXX7	EGVDRKTGQLKWTATANDLVF	0	0
A0QXX7	ENPAIPSPKPKSGAPLRNQDW	0	0
A0QXX7	ESENPAIPSPKPKSGAPLRNQ	0	0
A0QXX7	FVNDFVKAWVKVMNNDRFDLK	0	0
A0QXX7	GEEDEWLGTDKRYGGGEQRQL	0	1
A0QXX7	GFRNYVRPGEKVQLEKMLLER	0	0
A0QXX7	GNVALESMGFKTFGFGFGRED	0	1
A0QXX7	ILYGYEWELTKSPAGAWQFTA	0	0
A0QXX7	ISMRVDPIYGKITRRWLDHPE	0	0
A0QXX7	KVMNNDRFDLK__________	0	0
A0QXX7	LEVVWTNTPTKWDNSFLEILY	0	0
A0QXX7	LGWKSSYGTGKGPDTITSGLE	0	0
A0QXX7	LVDDQDIAALKSTVLDSGLST	0	0
A0QXX7	LWPIKQKYGNKISWADLLVFT	0	0
A0QXX7	NGARVRLEPQKNWDVNEPAEL	0	0
A0QXX7	QDFNASASGGKKVSLADLIVL	0	0
A0QXX7	RALDVNHGGTKHGVFTDRPGA	0	0
A0QXX7	SPAGAWQFTAKDGAGAGTIPD	0	0
A0QXX7	TENVYEGVDRKTGQLKWTATA	0	0
A0QXX7	VNLLDMGTEWKTSETTENVYE	0	0
A0QXX7	VRPGEKVQLEKMLLERAYFLG	0	0
A0QXX7	AGSAAIEKAAKDGGYNVTVPF	0	1
A0QXX7	HPEELSEAFAKAWYKLLHRDM	0	1
A0QXX7	IVLAGSAAIEKAAKDGGYNVT	0	1
A0QXX7	LSEAFAKAWYKLLHRDMGPIS	0	1
A0QXX7	NGERFVNDFVKAWVKVMNNDR	0	1
A0QXX7	SDSRPPNPDTKTASTSESENP	0	0
A0QYD4	SIVNVLPESAKEGSVEAAIKA	0	0
A0QYD4	AKEGSVEAAIKAALSDWTAGQ	0	1
A0QYD4	VNVPAPRWEAKDPRTYTLADL	0	1
A0QYS9	FKITRLTGKTKIVAAEGAFHG	0	0
A0QYS9	GHPLVDKVRGKGLLQGIVLTV	0	0
A0QYS9	GIVPDVVTMAKGLGGGLPIGA	0	0
A0QYS9	GSLALTGQPSKQAPFEPLPGN	0	0
A0QYS9	IEELGHPLVDKVRGKGLLQGI	0	0
A0QYS9	SGTEANEVAFKITRLTGKTKI	0	0
A0QYS9	VAFKITRLTGKTKIVAAEGAF	0	1
A0QYS9	VCTAAGLAVLKTLAAEDLVAR	0	0
A0QYS9	QGIVLTVPSAKAVETAARDAG	0	1
A0QYS9	YLVAAREITSKHGALLVLDEV	0	1
A0QZ54	AIKSVLETGQKGLRIQHLLDS	0	0
A0QZ54	AILRPGRLDVKIKIERPDAEA	0	0

A0QZ54	AKAVANSLAKKMAEVRGDDAR	0	0
A0QZ54	ANGDKEVMYFKDFNSGAMIQN	0	0
A0QZ54	ARNAKLMDTLKEARQQLLALR	0	0
A0QZ54	CSPNIEVKELKQGQTVRLNEA	0	0
A0QZ54	DTVDVFTSGRKMRLTCSPNIE	0	1
A0QZ54	EFNGDRALCIKAMIEKVVDRM	0	0
A0QZ54	ELPFLHKDLYKEYSLRPPKGV	0	0
A0QZ54	EVRGDDAREAKSYFLNIKGPE	0	0
A0QZ54	FLEVTYANGDKEVMYFKDFNS	0	0
A0QZ54	IAKAVANSLAKKMAEVRGDDA	0	0
A0QZ54	LNIKGPELLNKFVGETERHIR	0	0
A0QZ54	LYKEYSLRPPKGVLLYGPPGC	0	0
A0QZ54	NPDDWARISGKKGERIVYIRT	0	0
A0QZ54	PDDWARISGKKGERIVYIRTL	0	0
A0QZ54	PPGCGKTLIAKAVANSLAKKM	0	0
A0QZ54	RALCIKAMIEKVVDRMYAEID	0	0
A0QZ54	RDAVELPFLHKDLYKEYSLRP	0	0
A0QZ54	REAKSYFLNIKGPELLNKFVG	0	0
A0QZ54	RIDSLAARNAKLMDTLKEARQ	0	0
A0QZ54	RLTCSPNIEVKELKQGQTVRL	0	0
A0QZ54	VLLYGPPGCGKTLIAKAVANS	0	0
A0QZ54	AMIQNVVDRAKKYAIKSVLET	0	1
A0QZ54	DAEAAQDIFSKYLTEDLPVHA	0	0
A0QZ54	IRLIFQRAREKASEGTPVIVF	0	0
A0QZ54	KAGYAFERIPKAEVEDLVLEE	0	1
A0QZ54	LRPGRLDVKIKIERPDAEAAQ	0	1
A0QZ54	MIQNVVDRAKKYAIKSVLETG	0	1
A0QZ54	RPGDSLLVDTKAGYAFERIPK	0	0
A0QZ54	TDYFDDSRPRKLRPGDSLLVD	0	1
A0QZ54	VVDRAKKYAIKSVLETGQKGL	0	1
A0QZ54	VWLAEPLVAAKDLPDEPTDYF	0	1
A0QZA1	AGWQERYPDVKVHRRVERAQP	0	0
A0QZA1	QPAYWLLEEAKQAQLVVVGSH	0	0
A0QZA1	HGLTVQTEIRKESVARTLIDA	0	1
A0QZA1	NARHAIEQAQKVVADCLGETH	0	1
A0QZE3	DWFNEEGSRFKPSMMGSAVFT	0	0
A0QZE3	GLVDRTWAANKYELNVVGIQG	0	0
A0QZE3	IAEAEIRAGVKVEHRKAHVWP	0	0
A0QZE3	IRAGVKVEHRKAHVWPGHHYQ	0	0
A0QZE3	TRAGHDSTNMKEIVPSVMLFV	0	0
A0R066	AAVITPVSHVKHHDGEFTIAD	0	0
A0R066	ESILANPGFGKYYTDHMVSID	0	0
A0R066	HYGQEIFEGLKAYRWADGSIV	0	0
A0R066	LIASPAGAYFKGGIKPVSVWL	0	0
A0R066	PAGAYFKGGIKPVSVWLSHEY	0	1
A0R066	SLRQLIAVDEKWVPPAGGEES	0	1
A0R066	TDAGFAVEERKIDVDEWQKKA	0	1
A0R066	RASPGGTGAAKFGGNYAASLL	0	1

A0R066	RKIDVDEWQKKAGAGEITEVF	0	1
A0R079	AFTNPTVNSYKRLVPGYEAPI	0	0
A0R079	AMLMAGIDGIKKKIEPLQPVD	0	0
A0R079	EDLIETWISYKRENEIMPIQI	0	0
A0R079	ESDGSANRGYKVRPKGGYFPV	0	0
A0R079	GTAGQAEINYKFNTLLAAADD	0	0
A0R079	IIKNTAWQAGKTVTFMPKPLF	0	0
A0R079	IPITGNNPKAKRLEFRCPDSS	0	0
A0R079	KKIEPLQPVDKDLYELPPDEA	0	0
A0R079	LMAGIDGIKKKIEPLQPVDKD	0	0
A0R079	_______MAEKTSDDIFKLIK	0	0
A0R079	MAEKTSDDIFKLIKDENVEYV	0	0
A0R079	MLMAGIDGIKKKIEPLQPVDK	0	1
A0R079	QAGKTVTFMPKPLFGDNGSGM	0	1
A0R079	SANRGYKVRPKGGYFPVAPYD	0	0
A0R079	SGMHAHQSLWKDGQPLFHDES	0	0
A0R079	TARIDPFRAAKTLNMNFFVHD	0	0
A0R079	VRIPITGNNPKAKRLEFRCPD	0	0
A0R079	YIFDSVSFDSKINGTFYEVDS	0	1
A0R079	APTSLSAVIDKLEEDHEYLTE	0	1
A0R079	DDVLLFKYIIKNTAWQAGKTV	0	0
A0R079	LAAADDVLLFKYIIKNTAWQA	0	0
A0R079	YSRDPRNVARKAENYLASTGI	0	1
A0R083	ADGVRASFMPKPFAEHPGSAM	0	0
A0R083	AVTNQWVNSYKRLVHGGEAPT	0	0
A0R083	DDPLQLSDVAKSFIAGILEHA	0	0
A0R083	DNVMTFRYLVKEVALADGVRA	0	0
A0R083	DPSTFQVLPWKTSDGNHYSAR	0	0
A0R083	EHVFDYFLRNKRSEWENYRSH	0	0
A0R083	LVRVPMYTPHKVSSRRVEVRS	0	0
A0R083	LWFTDVLGYLKSVAIAPAELE	0	0
A0R083	______MDRQKEFVLRTLEER	0	0
A0R083	VHPEIEFFLLKPGPNDGTPPE	0	0
A0R083	YRSHVTPYELKNYLSL_____	0	0
A0R083	SRHVLRRQLAKASDLGFTCYV	0	1
A0R0B0	ATTMATVRTFKELLRDKNIGP	0	1
A0R0B0	DMSDQEIWNLKRGGHDYRKVY	0	1
A0R0B0	EGRNATHQMKKLALEDLKNFR	0	0
A0R0B0	ELNREGVAIEKHRLRHPDEPA	0	0
A0R0B0	EVGFNHFFRGKSHPGGGDHVF	0	0
A0R0B0	FEGRNATHQMKKLALEDLKNF	0	1
A0R0B0	FFRGAGWNVIKVVWGREWDVL	0	0
A0R0B0	FGMDSWFPSLKIYNRNGQLYT	0	0
A0R0B0	FNHYLHDRGIKDTSDQHVWAF	0	0
A0R0B0	GFVPSRRTKSKPLALPGSDTY	0	0
A0R0B0	GLYLYRPAPEKRAKSAQILAS	0	0
A0R0B0	HKGQPTVILAKTIKGYTLGQH	0	0
A0R0B0	LGGFVPSRRTKSKPLALPGSD	0	0

A0R0B0	LPGSDTYKALKKGSGSQAVAT	0	0
A0R0B0	LYRPAPEKRAKSAQILASGVA	0	0
A0R0B0	NLKRGGHDYRKVYAAYRAAME	0	0
A0R0B0	PENLDVEALLKGLYLYRPAPE	0	1
A0R0B0	PGSDTYKALKKGSGSQAVATT	0	0
A0R0B0	PLALPGSDTYKALKKGSGSQA	0	1
A0R0B0	QMKKLALEDLKNFRDVTRVPV	0	0
A0R0B0	QPTVILAKTIKGYTLGQHFEG	0	0
A0R0B0	RLDGPVRGNGKIIQELESFFR	0	0
A0R0B0	STPDGDYQTYKANDGAYVRDH	0	0
A0R0B0	SVDSELMLAYKESEVGQILHE	0	0
A0R0B0	VRTFKELLRDKNIGPRIVPII	0	0
A0R0B0	YAAYRAAMEHKGQPTVILAKT	0	0
A0R0B2	AGVISADDAVKLAATRGAEMA	0	1
A0R0B2	AMEKLVAQLTKPVRWDLCTAT	0	0
A0R0B2	ATLLAHEELTKRGHSAAETIV	0	0
A0R0B2	ELKGTPTRAIKSPEDLDGLDQ	0	0
A0R0B2	LAATRGAEMAKACAVEPTGMA	0	0
A0R0B2	PPAGTLVGIAKRELKGTPTRA	0	0
A0R0B2	PVASAADAMEKLVAQLTKPVR	0	0
A0R0B2	TLVGIAKRELKGTPTRAIKSP	0	1
A0R0B2	EDPPAKARVRKLATAGAFHTH	0	1
A0R0B2	LDKLAEDPPAKARVRKLATAG	0	1
A0R0B3	GIEPSEVTPEKSFVDDLDIDS	0	1
A0R0B3	MVEIAVQTEDKYGVKIPDEDL	0	0
A0R0B3	NPEAAAALREKFAADQ_____	0	0
A0R0B4	ADGLRAGQAMKRAMETAGLDP	0	0
A0R0B4	DPQGASRPFDKDRDGFVFGEA	0	0
A0R0B4	GVEHAAVYAPKSALGHSIGAV	0	1
A0R0B4	MMIIETEEHAKARGAKPLARL	0	0
A0R0B4	RAMETAGLDPKDIDHVNAHAT	0	1
A0R0B4	RRMSYVQRMSKFLSKQLWENA	0	0
A0R0B4	SIAPDIESTWKGLLAGESGIR	0	1
A0R0B4	TEEHAKARGAKPLARLMGAGI	0	1
A0R0B4	VAGEPRYGEYKYAINNSFGFG	0	1
A0R0B4	VIGTGLGGGEKIVETYDAMNE	0	0
A0R0B4	YDAMNEGGPRKVSPLAVQMIM	0	0
A0R0B4	YVQRMSKFLSKQLWENAGAPE	0	0
A0R0B5	AAAIGLERQAKAGVCTSISAC	0	0
A0R0B5	ALATDAESTWKKLLDGQSGIR	0	0
A0R0B5	DIAICGGVETKIEAVPIAGFA	0	0
A0R0B5	DPEGACKPFDKDRNGFVFGEA	0	0
A0R0B5	GGHKPAVYAPKGALGHSVGAV	0	0
A0R0B5	GTNVGDVAESKAINNAMGGHK	0	0
A0R0B5	KAINNAMGGHKPAVYAPKGAL	0	1
A0R0B5	LATDAESTWKKLLDGQSGIRT	0	1
A0R0B5	LMVIETEEHAKARGANILGRL	0	1
A0R0B5	MTRAIELAGLKPTDIDHVNAH	0	0

A0R0B5	NSNDDPEGACKPFDKDRNGFV	0	0
A0R0B5	VAGEPRQGDYKYAINNSFGFG	0	0
A0R0B5	GGHNVALAFGKY_________	0	1
A0R0B5	LVFAYDGMRSKGLRAVSPLVV	0	1
A0R0W1	ARIQVADIGFKGQMVDDGNGP	0	0
A0R0W1	EKPAPKTRPAKRPRGEGQWAL	0	0
A0R0W1	FCKLSFAETRKRAQVLVPELE	0	0
A0R0W1	FREVLETEYLKRPLIDGPAPE	0	0
A0R0W1	FRWWGLYTQRKPGYDGTWTGD	0	0
A0R0W1	GKPEYSNLPRKFKTAISGLQD	0	0
A0R0W1	IDEIVRRYIGKPEYSNLPRKF	0	0
A0R0W1	IQVENMPEIWKRLDAVGLQTT	0	1
A0R0W1	KFLIKDWGVEKFREVLETEYL	0	1
A0R0W1	KLIILDVPDDKIDELRAGLDA	0	1
A0R0W1	LMACTGIEFCKLSFAETRKRA	0	1
A0R0W1	__MTSSEAPAKTAEKPAPKTR	0	0
A0R0W1	PAKTAEKPAPKTRPAKRPRGE	0	0
A0R0W1	PEYSNLPRKFKTAISGLQDVV	0	0
A0R0W1	PIDHVGVQKLKNGLNAVGVAP	0	0
A0R0W1	RDYGYRRLRSKARLKFLIKDW	0	0
A0R0W1	RSKARLKFLIKDWGVEKFREV	0	0
A0R0W1	SGFGRKLRQHKVLSSELGDYI	0	0
A0R0W1	SLGLDSGFGRKLRQHKVLSSE	0	0
A0R0W1	SSEAPAKTAEKPAPKTRPAKR	0	0
A0R0W1	TRPIDHVGVQKLKNGLNAVGV	0	0
A0R0W1	YAKNGFESIDKGDLRGRFRWW	0	0
A0R0W1	YRRLRSKARLKFLIKDWGVEK	0	0
A0R0W1	AGRVSGTILTKVADLAEAAGS	0	1
A0R0W1	EPLNANEQAKKDDNPLNVRAR	0	1
A0R0W1	REPLNANEQAKKDDNPLNVRA	0	1
A0R0W1	SDRIRFTPYQKLIILDVPDDK	0	1
A0R0W1	VRARIENIYAKNGFESIDKGD	0	1
A0R0W1	YIERVVRNFVKQREDGERFAQ	0	0
A0R0W4	AFGLKIRKRPKAEIAERVDNL	0	0
A0R0W4	DNVAFGLKIRKRPKAEIAERV	0	0
A0R0W4	GFVFQHYAAFKHLSVRDNVAF	0	0
A0R0W4	SVRDNVAFGLKIRKRPKAEIA	0	0
A0R0W4	TALLGPSGSGKSTLLRAIAGL	0	0
A0R0W4	LDEPFGALDAKVRDDLRTWLR	0	0
A0R0W4	TSAIVVRGANKHYGDFAALDN	0	0
A0R1B5	DALKYGSRAAKARFAVYIGPL	0	0
A0R1B5	LGVSAAAASFKEGNLIDGLIS	0	0
A0R1B5	MDLVVLDDALKYGSRAAKARF	0	0
A0R1V9	FQWRAQHEDLKTLPFPMVSDL	0	0
A0R1V9	GKWRIIFFWPKDFTFVCPTEI	0	0
A0R1V9	QSDELCACNWKKGDPTINAGE	0	0
A0R1V9	SDELCACNWKKGDPTINAGEL	0	0
A0R1V9	TLPFPMVSDLKRELTAACGVL	0	1

A0R1V9	TRVTSKDYEGKWRIIFFWPKD	0	0
A0R1V9	VCPTEIAAFGKLNEDFEDRDA	0	1
A0R1V9	LNEDFEDRDAKVLGVSVDNEF	0	1
A0R1Y7	AAAVIVMNKAKAEELGLTWLA	0	1
A0R1Y7	DGAAAVIVMNKAKAEELGLTW	0	1
A0R1Y7	EVVPVSIPQRKGDPIEFAEDE	0	1
A0R1Y7	GAIAIRAALEKANVPASMVEY	0	1
A0R1Y7	HLLPKSREGYKYGDATLVDHL	0	1
A0R1Y7	IVAGARTPVGKLMGSLKDFSG	0	0
A0R1Y7	NTTAESLAGLKPAFRKDGTIT	0	0
A0R1Y7	PWDVAALSINKMCLSGIDAIA	0	0
A0R1Y7	QDEYAAQSHQKAAAAWKDGVF	0	0
A0R1Y7	SLAGLKPAFRKDGTITAGSAS	0	0
A0R1Y7	SMSQAPHLLPKSREGYKYGDA	0	0
A0R1Y7	STKELGVDPAKVNVNGGAIAI	0	0
A0R1Y7	TPVGKLMGSLKDFSGTDLGAI	0	1
A0R1Y7	AFAAVALASTKELGVDPAKVN	0	1
A0R1Y7	ALTEQRNDVDKFTRAEQDEYA	0	0
A0R1Y7	LQSQPANAIKKAITREGITVD	0	1
A0R1Y7	TLQSQPANAIKKAITREGITV	0	1
A0R218	ADKPAKEAQTKAAEAPEAGES	0	0
A0R218	AIDLLISQLRKTKTNYEFLVQ	0	0
A0R218	AKTERAEKADKPAKEAQTKAA	0	1
A0R218	AVDVNPSGTRKDELLLSPDEF	0	1
A0R218	DGQDGKQDQGKQDRSERDQDQ	0	1
A0R218	DLLISQLRKTKTNYEFLVQVS	0	0
A0R218	DRGEKDGQDGKQDQGKQDRSE	0	0
A0R218	EAKAEAPAEAKTERAEKADKP	0	0
A0R218	EAKTEAQPEAKAEAPAEAKTE	0	0
A0R218	EEVTDMQRSVKGEVIASTFDR	0	0
A0R218	EFKGTGNAELKLDRKIAERRV	0	0
A0R218	ERAEKADKPAKEAQTKAAEAP	0	0
A0R218	GDSGGQNQRQKFNPLVRLDTV	0	0
A0R218	GEKTEAQPEAKTEAQPEAKAE	0	0
A0R218	GVDSTALYPPKRFLGAARNIE	0	0
A0R218	IGVEGASGLRKSELIAAIRAH	0	1
A0R218	KGEAGEKAGEKTEAQPEAKTE	0	1
A0R218	LSPDEFAIVHKLRRVLSGLDS	0	0
A0R218	MVLPELRALAKEIGVEGASGL	0	0
A0R218	PAEAKTERAEKADKPAKEAQT	0	0
A0R218	PVAEKGEAGEKAGEKTEAQPE	0	1
A0R218	RVIDLIMPIGKGQRALIVSPP	0	0
A0R218	SDTGDLSGAVKSNTAPRARSA	0	0
A0R218	TGDTVIFEEFKGTGNAELKLD	0	0
A0R218	TGNAELKLDRKIAERRVFPAV	0	0
A0R218	TNYEFLVQVSKTAPGSMDVD_	0	0
A0R218	TRQAGAPVAEKGEAGEKAGEK	0	0
A0R218	ALIVSPPKAGKTTILQDIANA	0	0

A0R218	EDAKKRPEFGKLTPLYPNERL	0	1
A0R218	ERAKRLVEQGKDVVVLLDSIT	0	1
A0R218	GQRALIVSPPKAGKTTILQDI	0	1
A0R218	GRQDRADRGEKDGQDGKQDQG	0	1
A0R218	QAAELAIERAKRLVEQGKDVV	0	1
A0R218	TVNGGSVEDAKKRPEFGKLTP	0	1
A0R218	VNGGSVEDAKKRPEFGKLTPL	0	1
A0R220	ASAASIAGLLKSVEDGWVKRG	0	1
A0R220	GNAGNITAYWKGYSEYHRDGV	0	1
A0R220	ITCAVLIPQGKIAMGKLAQAV	0	0
A0R220	KLAQAVMHGAKIIQVDGNFDD	0	0
A0R220	LEGGTPLIHAKRISELTGCTV	0	0
A0R220	LIPQGKIAMGKLAQAVMHGAK	0	0
A0R220	LLKSVEDGWVKRGSTVVCTVT	0	0
A0R220	______MSAAKAAVHQPWPGL	0	0
A0R220	NGLKDPDTALKGMPQVTPVPV	0	0
A0R220	SELTGCTVHLKVEGLNPTGSF	0	1
A0R220	SVNPYRIEGQKTAAFEIVDAL	0	1
A0R220	VEGLNPTGSFKDRGMTVAVTE	0	1
A0R220	VVCTVTGNGLKDPDTALKGMP	0	0
A0R220	AAPLVTGAPVKDPETIATAIR	0	1
A0R2G5	GFETTRARLRKRGAVAVYGID	0	0
A0R2G5	GGGKEVIKVGKRCLLGANSGL	0	1
A0R2G5	RGAVAVYGIDKFPRMVDYVTP	0	0
A0R2G5	RLHLLSHRLTKPHEANLDGIF	0	1
A0R2G5	SIMGTLSGGGKEVIKVGKRCL	0	1
A0R2G5	TLSGGGKEVIKVGKRCLLGAN	0	0
A0R2G5	VPADLAALTGKDEDRDVEVVA	0	1
A0R2G5	VRTTIADLNDKPADTHDVWLR	0	1
A0R2G5	EAGLYVTGGTKVTTADGQVTK	0	1
A0R2G5	KVTTADGQVTKAIELSGASNL	0	1
A0R2V7	ATEEYAPAVDKGCPMVLGGPL	0	0
A0R2V7	DAVAAAAREFKPLVLVAGYSA	0	0
A0R2V7	EAPELANFGAKHINDLSEADW	0	0
A0R2V7	GGPLSHVMAAKAVALAEARQP	0	0
A0R2V7	HAHVTTTTTHKSLRGPRGGMV	0	0
A0R2V7	KELADQRDSLKLIASENYASP	0	0
A0R2V7	LANTQPEGTSKAKYKLADGTA	0	0
A0R2V7	LLTMGTWFSDKYAEGTIGHRF	0	0
A0R2V7	MAHFAGLVAGKVFTGDEDPVP	0	0
A0R2V7	NTQPEGTSKAKYKLADGTAER	0	0
A0R2V7	QPEGTSKAKYKLADGTAERVH	0	1
A0R2V7	SAYPRRVNFAKMREIADEVGA	0	1
A0R2V7	SEADWETLRNKLGNQRLLGMS	0	1
A0R2V7	THGFRPNISGKMFHQRSYGTN	0	0
A0R2V7	VEPRVAAATRKELADQRDSLK	0	0
A0R2W6	FDGDGRITSMKAYWSPADIKQ	0	0
A0R2W6	HDAAALESFVKRYLDTVAGGS	0	0

A0R2W6	MKAYWSPADIKQL________	0	0
A0R2W6	KNMTAATTDIKTELLSFRPGG	0	1
A0R342	ACLNAIEYLKKWGYTGEQAYL	0	1
A0R342	APCHMLSGPIKVEGAEPGDLL	0	0
A0R342	DIRPGGKGPQKVDRGQVAVTS	0	0
A0R342	EGWGYSGIFAKVNGGGFLTDY	0	0
A0R342	FLTDYYPDAYKAIWDFHGQQC	0	0
A0R342	GFIDMHVDLIKGGMETYGVTT	0	0
A0R342	IFDFDIRPGGKGPQKVDRGQV	0	0
A0R342	KPGSEFRIECKEWTDGQIGNN	0	0
A0R342	NACLNAIEYLKKWGYTGEQAY	0	0
A0R342	PEVVFSVDHSKSMRDQAVPGH	0	1
A0R342	PRENGGNHDIKNFTRGSRIFY	0	0
A0R342	GTAPSPDLLAKWNERERALIA	0	1
A0R3D2	GASRQARRDGKVPAVLYGHGT	0	0
A0R3D2	________MAKTANIPNKLTA	0	0
A0R3D2	_MAKTANIPNKLTANVRTRTG	0	0
A0R3D2	ADLLVVQRGEKVTVEVTVLVE	0	1
A0R3D2	IEGTEQLALTKALDVHPIRRN	0	1
A0R3D6	AALRRAVPATKGNTKRLASLG	0	0
A0R3D6	ADVEDFLDAGKPFGLRPEAIL	0	0
A0R3D6	DFLRDTLLWAKLIDKYKGTMT	0	0
A0R3D6	DTLLWAKLIDKYKGTMTAAPN	0	0
A0R3D6	EGNIVVCGRVKDVIIMAGRNI	0	0
A0R3D6	IPMYFGAELVKVTPMDFLRDT	0	0
A0R3D6	LLWAKLIDKYKGTMTAAPNFA	0	1
A0R3D6	MFVGAKYDVEKDVMVSWLPCF	0	0
A0R3D6	____MSRFTEKMYRNARTVTT	0	1
A0R3D6	QLTSGSTGSPKAVQITHRNIH	0	0
A0R3D6	RAVPATKGNTKRLASLGPLLN	0	0
A0R3D6	SNAEAMFVGAKYDVEKDVMVS	0	0
A0R3D6	TMNVIGMIELKAVIISEPFLV	0	1
A0R3D6	FLVATPVLEEKGILVLKVADL	0	0
A0R3D6	PGSIPKTPSGKLRRANSVSLV	0	0
A0R3D6	VLEEKGILVLKVADLLAADPI	0	1
A0R4C9	DVKLLDGGRKKWELDARPLSA	0	0
A0R4C9	DVRSPDEFSGKILAPAHLPQE	0	0
A0R4C9	DWAESNLKAPKTVFVEVDEDT	0	0
A0R4C9	GTFKSDEELAKLYAEAGLDGE	0	0
A0R4C9	IPGAINVPWSKAANEDGTFKS	0	0
A0R4C9	LQELLGHKNVKNYDGSWTEYG	0	0
A0R4C9	LYAEAGLDGEKETIAYCRIGE	0	0
A0R4C9	QDVKLLDGGRKKWELDARPLS	0	0
A0R4C9	VSTDWAESNLKAPKTVFVEVD	0	0
A0R4C9	WFAAYAYWYFKLYGHQDVKLL	0	0
A0R4C9	WFVLQELLGHKNVKNYDGSWT	0	0
A0R4C9	YDTGHIEGAVKLDWKTDLQDP	0	0
A0R4C9	YFKLYGHQDVKLLDGGRKKWE	0	0

A0R4C9	ELDARPLSAEKVERPQTSYTA	0	1
A0R4C9	HIEGAVKLDWKTDLQDPIRRD	0	1
A0R4C9	RDEVIAAIGTKNLVDVRSPDE	0	1
A0R4C9	SKAANEDGTFKSDEELAKLYA	0	1
A0R4C9	VERPQTSYTAKEPDNSIRAFR	0	1
A0R4D0	__MPAGVDLEKETVITGRVVD	0	0
A0R4Z5	AFAPVVLAWIKDLKADPEKVN	0	0
A0R4Z5	AVLLASEQAVKEHNLKPRARI	0	0
A0R4Z5	GPIPATEYALKKTGLSIDDID	0	0
A0R4Z5	IDAVRTPVGKKNGSLAQVHPI	0	0
A0R4Z5	IIDAVRTPVGKKNGSLAQVHP	0	0
A0R4Z5	LGHPLGATGAKLFATMLNTLE	0	0
A0R4Z5	PIPATEYALKKTGLSIDDIDT	0	0
A0R4Z5	PVVLAWIKDLKADPEKVNPNG	0	0
A0R4Z5	SEQAVKEHNLKPRARIHHISA	0	0
A0R4Z5	VPISSAMIVGKELGFSTPTAE	0	0
A0R4Z5	WIKDLKADPEKVNPNGGAIAL	0	0
A0R518	EVDVRDYDALKAVVDSGVEQL	0	0
A0R518	GIGNGGATLDKTSEADWDDMI	0	0
A0R518	IILTSSVGGLKAYPHTGHYIA	0	0
A0R518	___MAGRVEGKVAFITGAARG	0	0
A0R518	MIGVNLSGVWKTVKAAVPHLI	0	0
A0R518	PMTVDAGSMLK__________	0	0
A0R518	TPLFMNEGTMKLFRPDLENPG	0	0
A0R518	VNLSGVWKTVKAAVPHLISGG	0	0
A0R518	YPHTGHYIAAKHGVVGLMRTF	0	0
A0R566	GSIEYAVTVLKVPLIVVLGHD	0	0
A0R566	RVTPSILLGRKAGLSRVDEFE	0	0
A0R566	VLGHDSCGAVKATLSALDEGE	0	0
A0R566	SNPVAAWKALKDGNARFVAGQ	0	1
A0R5E1	KVEFEVGQSPKGPQATGVRTI	0	0
A0R5E1	____MPQGTVKWFNAEKGFGF	0	0
A0R5E1	QGTVKWFNAEKGFGFIAPEDG	0	0
A0R5M8	DSLLLGTDLVKDTGRLVRAYD	0	0
A0R5M8	DVRAGLTAAPKSLPPKWFYDA	0	0
A0R5M8	GEEMLTEVSCKFRPENVVAEL	0	0
A0R5M8	LTAAPKSLPPKWFYDAVGSDL	0	0
A0R5M8	LVELGSGTSEKTRMLLDAMRD	0	1
A0R5M8	VCGDFEEHLGKIPHVGRRLVV	0	0
A0R5R5	AEILEIFAAGKEPLKFTAYDG	0	0
A0R5R5	AVEEVGLPTAKVWGLYMAGSR	0	0
A0R5R5	EIFAAGKEPLKFTAYDGSSAG	0	0
A0R5R5	HNYPAYFNFLKSKLRTGGLLL	0	0
A0R5R5	IVRSIGIEHLKPIAPPPQEAL	0	0
A0R5R5	LRALAERMEFKRPPARVLANI	0	0
A0R5R5	______MTTFKERETSTADRK	0	0
A0R5R5	MVRYAARHGVKALGVTLSREQ	0	0
A0R5R5	PEDATMGLDLKTPRGTTYLAT	0	0

A0R5R5	QDNKYRLVFEKLRLKPGDRLL	0	0
A0R5R5	RRIMEGLRHSKTRDAEAIHHH	0	0
A0R5R5	VLAVKLDDQGKDGGLPLRPWW	0	0
A0R5R5	YPAYFNFLKSKLRTGGLLLNH	0	0
A0R5R5	YRLVFEKLRLKPGDRLLDVGC	0	0
A0R5R5	DATLEEAQDNKYRLVFEKLRL	0	1
A0R5R5	KERETSTADRKLTLAEILEIF	0	1
A0R5R5	VVQLHQVLAVKLDDQGKDGGL	0	1
A0R5R7	AFPYRKDPEDKNIRIAPTFPS	0	0
A0R5R7	ASADNIAWYLKHAGKKSIGPD	0	0
A0R5R7	EQLVASDPTIKGIWCVPVYSN	0	0
A0R5R7	ESKIASWTDPKGGYFVSLDVW	0	0
A0R5R7	GTAKRTVALAKDAGIAVTEAG	0	0
A0R5R7	HAGKKSIGPDKVNQLRHLRFF	0	0
A0R5R7	IAWYLKHAGKKSIGPDKVNQL	0	0
A0R5R7	KQLKLDLTRGKPSPEQLDLSN	0	0
A0R5R7	LAATEALLGDK__________	0	0
A0R5R7	MQRHRELIAPKFALVAEILED	0	0
A0R5R7	NIAWYLKHAGKKSIGPDKVNQ	0	0
A0R5R7	NRPLVFASTSKITFAGAGVSF	0	0
A0R5R7	NYAELQAKQLKLDLTRGKPSP	0	0
A0R5R7	PRPWSAEPTVKFLCPAPGYDR	0	0
A0R5R7	QQRNYAELQAKQLKLDLTRGK	0	0
A0R5R7	RRLVQMPTAAKDFRLMWDNAY	0	0
A0R5R7	TEAGSAFPYRKDPEDKNIRIA	0	0
A0R5R7	VSLDVWPGTAKRTVALAKDAG	0	0
A0R635	ARAKEAFGIQKRQRQMDRCRQ	0	0
A0R635	DDPESIFTTGKAAYIEDYAQR	0	0
A0R635	DSVKHTVSGPKGDLDVMIIAL	0	0
A0R635	FQIQRRCPHLKADLSKFGVVE	0	0
A0R635	GYNEYLYTFFKCLTDERIAYA	0	0
A0R635	HNFFETTDSVKHTVSGPKGDL	0	0
A0R635	NLENGRCLTTKGHELRCQKL_	0	0
A0R635	PMVYDMPARAKEAFGIQKRQR	0	0
A0R635	PVPDLRRELEKLGFHNFFETT	0	0
A0R635	RCPHLKADLSKFGVVEGNTLT	0	0
A0R635	TTKGHELRCQKL_________	0	0
A0R635	DPEHLRRYVNKDAVVLLPDYP	0	1
A0R635	QRMAPVLAAEKARWAPSAGEP	0	1
A0R635	RVVREPIPDEKFRYGFEIPAA	0	1
A0R635	VDYLYVSHLHKDHFDPEHLRR	0	1
A0R647	FDGADLPQLAKHFYAQALEER	0	0
A0R647	LGEQFMQWFLKEQVEEVAAMT	0	0
A0R652	AIDDQFGSFDKFQAQFTAAAN	0	0
A0R652	GDHAAIFLNEKNLAFHLGGHI	0	0
A0R652	GGHINHSIWWKNLSPNGGDKP	0	0
A0R652	HAFYLQYKNVKADYVKAFWNV	0	0
A0R652	MWEHAFYLQYKNVKADYVKAF	0	0

A0R652	QYKNVKADYVKAFWNVVNWDD	0	0
A0R652	YVKGVNDAIAKLEEARANGDH	0	0
A0R652	SGQINELHHSKHHATYVKGVN	0	1
A0R652	VQNRFAAATSKTSGLIFG___	0	1
A0R6E3	AEAANGIGVRKDLIEAVEAEE	0	1
A0R6E3	QRLYVARLRAKLTTAEAANGI	0	1
A0R6Q0	ATPVDHPIPGKVVVGIKTADV	0	0
A0R6Q0	EIEATGGAIIKGPYDDAHERR	0	0
A0R6Q0	GNGLVFYSPLKR_________	0	0
A0R6Q0	KFYETLGFSLKFRDGAHFAAL	0	0
A0R6Q0	LSTENLDESIKFYETLGFSLK	0	0
A0R6Q0	____MSDHEVKMVVLSTENLD	0	1
A0R6Q0	PIPGKVVVGIKTADVDAAAKE	0	1
A0R7F9	GRRRLAYEIAKHAEGIYAVID	0	0
A0R7F9	VLRTKVLRTDKH_________	0	0
A0R7F9	AEGIYAVIDVKAEPATVSELD	0	1
A0R7F9	LNLNESVLRTKVLRTDKH___	0	1
A0R7F9	SLETFLNVIRKDGGTVDKVDI	0	1
A0R7G6	AGVPIVGDDIKSQVGATITHR	0	0
A0R7G6	AIFASENNTIKIADVPPTDVI	0	0
A0R7G6	ASAYLMKSPPKQLADDVARAE	0	0
A0R7G6	FIASDPVWAKKFEDAGVPIVG	0	0
A0R7G6	GATITHRVMAKLFEDRGVTLD	0	0
A0R7G6	MLERSRLESKKVSKTQAVTSN	0	0
A0R7G6	NMLERSRLESKKVSKTQAVTS	0	0
A0R7G6	NTTVPGLMHVKFGPYHVRDVN	0	0
A0R7G6	PIEAASAYLMKSPPKQLADDV	0	0
A0R7G6	QRGPTLDGIGKYYADTIEVSD	0	0
A0R7G6	RSRLESKKVSKTQAVTSNLSG	0	0
A0R7G6	SDHVAWLDDRKWAYVRLEGRA	0	0
A0R7G6	SGALAGKVEDKNVHIGPSDHV	0	0
A0R7G6	TSNLSGALAGKVEDKNVHIGP	0	0
A0R7G6	VFIASDPVWAKKFEDAGVPIV	0	0
A0R7G6	VSDAEPVDVVKVLKEAEVDVL	0	0
A0R7G6	AEPVDVVKVLKEAEVDVLVSY	0	1
A0R7G6	FVAAFDVDAKKVGFDLSEAIF	0	1
A0R7G6	IDAVRAAKIAKDRGIGGPIEA	0	1
A4ZHR8	ADIVITATGNKDIITLDHMKA	0	0
A4ZHR8	ADLSLADFGRKEIRLAEHEMP	0	0
A4ZHR8	DMAALERSGAKKINIKPQVDQ	0	0
A4ZHR8	DSVTKSKFDNKYGTRHSLIDG	0	0
A4ZHR8	EPKGVSVFAWKGETLEEYWWA	0	0
A4ZHR8	ERSGAKKINIKPQVDQWIFDD	0	0
A4ZHR8	EYDNEVYRLAKHLDEKVARIH	0	1
A4ZHR8	GVLRERFEQDKTKWTKIAESV	0	0
A4ZHR8	IITLDHMKAMKNQAILGNIGH	0	0
A4ZHR8	INVNDSVTKSKFDNKYGTRHS	0	0
A4ZHR8	LRERFEQDKTKWTKIAESVKG	0	0

A4ZHR8	MAALERSGAKKINIKPQVDQW	0	0
A4ZHR8	MMVLRGAQYEKAGVVPPAEDD	0	0
A4ZHR8	______MTELKADVRNGIDYK	0	0
A4ZHR8	NKDIITLDHMKAMKNQAILGN	0	0
A4ZHR8	NRGTDVLIGGKKVLICGYGDV	0	0
A4ZHR8	PAEDDDPAEWKVFLGVLRERF	0	0
A4ZHR8	PAINVNDSVTKSKFDNKYGTR	0	0
A4ZHR8	QVDQWIFDDGKSIIVLSEGRL	0	0
A4ZHR8	RFEQDKTKWTKIAESVKGVTE	0	0
A4ZHR8	RGTDVLIGGKKVLICGYGDVG	0	0
A4ZHR8	RREYHDVQPLKGARISGSLHM	0	0
A4ZHR8	TKWTKIAESVKGVTEETTTGV	0	0
A4ZHR8	VGPNGTPEEPKGVSVFAWKGE	0	0
A4ZHR8	VLICGYGDVGKGCAESLAGQG	0	0
A4ZHR8	VYRLAKHLDEKVARIHVEALG	0	0
A4ZHR8	YIGVDVEGPYKPEHYRY____	0	0
A4ZHR8	HVEALGGTLTKLSKDQAEYIG	0	1
A4ZHR8	KADVRNGIDYKVADLSLADFG	0	1
A4ZHR8	QVIAQIELWTKNDEYDNEVYR	0	1
EG10025	AAAPAPAAGVKEVNVPDIGGD	0	0
EG10025	ADAAPAQAEEKKEAAPAAAPA	0	0
EG10025	APFAGVVKELKVNVGDKVKTG	0	0
EG10025	DAAPAQAEEKKEAAPAAAPAA	0	0
EG10025	DVQAYVKEAIKRAEAAPAATG	0	0
EG10025	EEAAKRKLDVKITPVVFIMKA	0	1
EG10025	EGAAPAAAPAKQEAAAPAPAA	0	0
EG10025	ELKVNVGDKVKTGSLIMIFEV	0	0
EG10025	ELSRELMTISKKARDGKLTAG	0	0
EG10025	EQSLITVEGDKASMEVPAPFA	0	0
EG10025	EQSLITVEGDKASMEVPSPQA	0	0
EG10025	EVPAPFAGTVKEIKVNVGDKV	0	0
EG10025	EVPAPFAGVVKELKVNVGDKV	0	0
EG10025	EVPSPQAGIVKEIKVSVGDKT	0	0
EG10025	FRKQQNEEAAKRKLDVKITPV	0	0
EG10025	GEAGAAAPAAKQEAAPAAAPA	0	0
EG10025	GGIPGMLPWPKVDFSKFGEIE	0	0
EG10025	IEEVELGRIQKISGANLSRNW	0	0
EG10025	ILREDVQAYVKEAIKRAEAAP	0	0
EG10025	KQQNEEAAKRKLDVKITPVVF	0	0
EG10025	KSAMEPVWNGKEFVPRLMLPI	0	0
EG10025	LAREFGVNLAKVKGTGRKGRI	0	0
EG10025	LSEDGQRLTLKKYINIGVAVD	0	0
EG10025	LSRELMTISKKARDGKLTAGE	0	0
EG10025	LVVPVFKDVNKKGIIELSREL	0	0
EG10025	_____MAIEIKVPDIGADEVE	0	0
EG10025	MLPWPKVDFSKFGEIEEVELG	0	0
EG10025	MTISKKARDGKLTAGEMQGGC	0	0
EG10025	NLAKVKGTGRKGRILREDVQA	0	0

EG10025	PAAAPAAAAAKDVNVPDIGSD	0	0
EG10025	QEAAAPAPAAKAEAPAAAPAA	0	0
EG10025	REFGVNLAKVKGTGRKGRILR	0	1
EG10025	SEDGQRLTLKKYINIGVAVDT	0	0
EG10025	TPNGLVVPVFKDVNKKGIIEL	0	0
EG10025	VKEIKVNVGDKVSTGSLIMVF	0	0
EG10025	VKEIKVSVGDKTQTGALIMIF	0	0
EG10025	VKELKVNVGDKVKTGSLIMIF	0	0
EG10025	VKITPVVFIMKAVAAALEQMP	0	0
EG10025	VMIPHVTHFDKTDITELEAFR	0	0
EG10025	VVPVFKDVNKKGIIELSRELM	0	0
EG10025	AAAPAAKAEGKSEFAENDAYV	0	1
EG10025	AEAPAAAPAAKAEGKSEFAEN	0	1
EG10025	APEVAILGVSKSAMEPVWNGK	0	1
EG10025	DEVEITEILVKVGDKVEAEQS	0	1
EG10025	DEVEVTEILVKVGDKVEAEQS	0	1
EG10025	DEVEVTEVMVKVGDKVAAEQS	0	1
EG10025	ITEILVKVGDKVEAEQSLITV	0	1
EG10025	SPQAGIVKEIKVSVGDKTQTG	0	1
EG10025	TDITELEAFRKQQNEEAAKRK	0	1
EG10025	VTEILVKVGDKVEAEQSLITV	0	1
EG10025	VTEVMVKVGDKVAAEQSLITV	0	1
EG10207	ATVGSCAQIGKNVHLSGGVGI	0	0
EG10207	DGQWVTHQWLKKAVLLSFRIN	0	0
EG10207	ESRYFDKVPMKFADYDEARFQ	0	0
EG10207	GQWVTHQWLKKAVLLSFRIND	0	0
EG10207	IEGAESRYFDKVPMKFADYDE	0	0
EG10207	LDSGALRVAEKIDGQWVTHQW	0	0
EG10207	SVVVSGNLPSKDGKYSLYCAV	0	0
EG10207	VSGNLPSKDGKYSLYCAVIVK	0	0
EG10207	CAVIVKKVDAKTRGKVGINEL	0	1
EG10207	KYSLYCAVIVKKVDAKTRGKV	0	1
EG10207	VKKVDAKTRGKVGINELLRTI	0	1
EG10207	YSLYCAVIVKKVDAKTRGKVG	0	1
EG10251	MMLPRSGLGHKHGIVLGNLVG	0	0
EG10251	_________MKKIDVKILDPR	0	1
EG10251	________MKKIDVKILDPRV	0	1
EG10251	____MKKIDVKILDPRVGKEF	0	1
EG10367	AGIALNDNFVKLVSWYDNETG	0	0
EG10367	AKKVVMTGPSKDNTPMFVKGA	0	1
EG10367	ARKHITAGAKKVVMTGPSKDN	0	1
EG10367	ATYEQIKAAVKAAAEGEMKGV	0	0
EG10367	AVKAAAEGEMKGVLGYTEDDV	0	0
EG10367	CTTNCLAPLAKVINDNFGIIE	0	0
EG10367	GEVCTSVFDAKAGIALNDNFV	0	0
EG10367	GLFLTDETARKHITAGAKKVV	0	0
EG10367	HGRFDGTVEVKDGHLIVNGKK	0	0
EG10367	IGRIVFRAAQKRSDIEIVAIN	0	0

EG10367	KDGHLIVNGKKIRVTAERDPA	0	0
EG10367	KVLDLIAHISK__________	0	0
EG10367	LDADYMAYMLKYDSTHGRFDG	0	0
EG10367	LEKAATYEQIKAAVKAAAEGE	0	0
EG10367	_______MTIKVGINGFGRIG	0	0
EG10367	NIIPSSTGAAKAVGKVLPELN	0	0
EG10367	PMFVKGANFDKYAGQDIVSNA	0	0
EG10367	PSKDNTPMFVKGANFDKYAGQ	0	0
EG10367	TARKHITAGAKKVVMTGPSKD	0	0
EG10367	TQKTVDGPSHKDWRGGRGASQ	0	0
EG10367	TTVHATTATQKTVDGPSHKDW	0	0
EG10367	VGKVLPELNGKLTGMAFRVPT	0	0
EG10367	WYDNETGYSNKVLDLIAHISK	0	0
EG10367	SSTGAAKAVGKVLPELNGKLT	0	1
EG10367	VKDGHLIVNGKKIRVTAERDP	0	1
EG10367	VTAERDPANLKWDEVGVDVVA	0	1
EG10407	DFAEFDADAAKKHLRPVARQP	0	1
EG10407	DLVKLLGERCKTLKEMAQSCR	0	1
EG10407	DQMLEEGTAYKCYCSKERLEA	0	0
EG10407	EGTAYKCYCSKERLEALREEQ	0	0
EG10407	EWDEGPYYQTKRFDRYNAVID	0	0
EG10407	FAEFDADAAKKHLRPVARQPL	0	0
EG10407	HVSMINGDDGKKLSKRHGAVS	0	0
EG10407	INGDDGKKLSKRHGAVSVMQY	0	0
EG10407	KLLGERCKTLKEMAQSCRYFY	0	0
EG10407	LEALREEQMAKGEKPRYDGRC	0	0
EG10407	LREEQMAKGEKPRYDGRCRHS	0	0
EG10407	NNTPRQINILKALKAPVPVYA	0	0
EG10407	PRQINILKALKAPVPVYAHVS	0	0
EG10407	RNGPQLADLVKLLGERCKTLK	0	0
EG10407	SKSASAFNTDKLLWLNHHYIN	0	0
EG10407	TADELEVGMGKVGMPLRVAVT	0	0
EG10407	VSMINGDDGKKLSKRHGAVSV	0	0
EG10407	ALDVTVHAIGKTRSIERINKA	0	1
EG10407	ARQPLEVVRDKLAAITDWTAE	0	1
EG10407	GKTRSIERINKALDFIAEREN	0	1
EG10407	IKYFTLNAVSKSASAFNTDKL	0	1
EG10407	_________MKIKTRFAPSPT	0	1
EG10407	_______MKIKTRFAPSPTGY	0	1
EG10410	ADYEERKAKIKADAEEAARKI	0	0
EG10410	AEAQPDREIEKRGPAIAQAFD	0	0
EG10410	AEFFFNTDRKKRLEDNLPRLQ	0	0
EG10410	AIAQAFDAEGKPSKAAEGWAR	0	0
EG10410	ALVYTMKGDQKYFPVYANDGK	0	0
EG10410	ANKRVSNILAKSDEVLSDRVN	0	0
EG10410	ASTLKEPEEIKLAMQVVVLRD	0	0
EG10410	ATSLAKLPIPKLMRWGASDVH	0	0
EG10410	DAAAALAAANKRVSNILAKSD	0	0

EG10410	DAEFFFNTDRKKRLEDNLPRL	0	0
EG10410	FDKVMVMVDDKELRINRLTML	0	0
EG10410	FQQQLGTLRDKTDRIQALAGW	0	0
EG10410	GEWLLYRAHVKGESTEALLPN	0	0
EG10410	GKVIADYEERKAKIKADAEEA	0	0
EG10410	IKADAEEAARKIGGNADLSES	0	0
EG10410	LPNMVATSLAKLPIPKLMRWG	0	0
EG10410	LREPVDAFFDKVMVMVDDKEL	0	0
EG10410	LSDRVNASTLKEPEEIKLAMQ	0	0
EG10410	LVEWPVVLTAKFEEKFLAVPA	0	0
EG10410	NFIFVANIESKDPQQIISGNE	0	0
EG10410	NHATRAGLLSKCDLMTNMVFE	0	0
EG10410	PVACALAIADKMDTLAGIFGI	0	0
EG10410	PVVLTAKFEEKFLAVPAEALV	0	0
EG10410	QAFDAEGKPSKAAEGWARGCG	0	0
EG10410	QYPEILRERGKVIADYEERKA	0	0
EG10410	TEEAVRLYGDKLTNANVVDDV	0	0
EG10410	TRPADFDARMKAVSHFRTLDA	0	0
EG10410	VDQAERLTTDKGEWLLYRAHV	0	0
EG10410	VHTVTLLLGDKVIPATILGIQ	0	0
EG10410	VIADYEERKAKIKADAEEAAR	0	0
EG10410	VPAEALVYTMKGDQKYFPVYA	0	0
EG10410	WFAAPRRLALKVANLAEAQPD	0	0
EG10410	AGIFGIGQHPKGDKDPFALRR	0	1
EG10410	ALGVLRIIVEKNLNLDLQTLT	0	1
EG10410	DPQQIISGNEKVVRPRLADAE	0	1
EG10410	FGIGQHPKGDKDPFALRRAAL	0	1
EG10410	KYFPVYANDGKLLPNFIFVAN	0	1
EG10410	LRINRLTMLEKLRELFLRVAD	0	1
EG10410	_______MSEKTFLVEIGTEE	0	1
EG10410	VEIGTEELPPKALRSLAESFA	0	1
EG10416	AMVEGIELTLKSMLDVVRKFG	0	0
EG10416	APGNVLGIMQKGYTLNGRTIR	0	0
EG10416	_______MSSKEQKTPEGQAP	0	0
EG10416	____MSSKEQKTPEGQAPEEI	0	0
EG10416	RAAMVTVAKAKA_________	0	0
EG10416	SLDRALEVADKANPDMSAMVE	0	0
EG10416	TIRAAMVTVAKAKA_______	0	0
EG10416	TLKSMLDVVRKFGVEVIAETN	0	0
EG10416	IEKAHKFALEKFINELLPVID	0	1
EG10416	LRRRTELDIEKAHKFALEKFI	0	1
EG10416	RTELDIEKAHKFALEKFINEL	0	1
EG10489	AVEKAYKGERKISWMEIYTGE	0	0
EG10489	DLIREYRVAIKGPLTTPVGGG	0	0
EG10489	ERLMDGAKLLKCSEFGDAIIE	0	0
EG10489	GELIDGGPWLKVKNPNTGKEI	0	0
EG10489	GIKPCSEEGTKRLVRAAIEYA	0	0
EG10489	GTAPKYAGQDKVNPGSIILSA	0	1

EG10489	GWTEAADLIVKGMEGAINAKT	0	0
EG10489	IGVDVTPAMLKVVDAAVEKAY	0	0
EG10489	IKFLREEMGVKKIRFPEHCGI	0	0
EG10489	ISWMEIYTGEKSTQVYGQDVW	0	0
EG10489	KFLREEMGVKKIRFPEHCGIG	0	0
EG10489	LFEATHGTAPKYAGQDKVNPG	0	0
EG10489	LIDGGPWLKVKNPNTGKEIVI	0	0
EG10489	MLKVVDAAVEKAYKGERKISW	0	0
EG10489	NDRDSVTLVHKGNIMKFTEGA	0	0
EG10489	NIMKFTEGAFKDWGYQLAREE	0	0
EG10489	QGKKITLQNGKLNVPENPIIP	0	0
EG10489	RFPEHCGIGIKPCSEEGTKRL	0	0
EG10489	RYYQGTPSPVKHPELTDMVIF	0	0
EG10489	VKGMEGAINAKTVTYDFERLM	0	0
EG10489	VTLVHKGNIMKFTEGAFKDWG	0	0
EG10489	VVDAAVEKAYKGERKISWMEI	0	0
EG10489	YDFERLMDGAKLLKCSEFGDA	0	0
EG10489	ADSADAEKVIKFLREEMGVKK	0	1
EG10489	ESKVVVPAQGKKITLQNGKLN	0	1
EG10489	EWKADSADAEKVIKFLREEMG	0	1
EG10489	NPNTGKEIVIKDVIADAFLQQ	0	1
EG10489	SEDIYAGIEWKADSADAEKVI	0	1
EG10489	SKVVVPAQGKKITLQNGKLNV	0	1
EG10492	AFQAVDQDALKAKFAVSNVNG	0	0
EG10492	AQQSEVLKGLKVALSKAEGEK	0	0
EG10492	CAKAAQEDILKAYEAYDFHEV	0	0
EG10492	CVSNVAGDGEKRKFA______	0	0
EG10492	DDLYGIIRAAKKGKKTFILHD	0	0
EG10492	DIIKDRQYTAKADSVARRSCQ	0	0
EG10492	DLYGIIRAAKKGKKTFILHDG	0	0
EG10492	DQKGLRAQSLKEIKGVQWIPD	0	0
EG10492	EKFTAAEFRAKCREYAATQVD	0	0
EG10492	ELLKVRGEVNKVIEQARADKK	0	0
EG10492	EMGSFYLDIIKDRQYTAKADS	0	0
EG10492	GIIRAAKKGKKTFILHDGPPY	0	0
EG10492	GIQAWWDLDAKEILGDEADQY	0	1
EG10492	GKIIGNGHLHKGAKPVHWCVD	0	0
EG10492	GLRAQSLKEIKGVQWIPDWGQ	0	0
EG10492	GSIHIGHSVNKILKDIIVKSK	0	0
EG10492	HGFTVDGQGRKMSKSIGNTVS	0	0
EG10492	HGPDDYVIGQKYGLETANPVG	0	0
EG10492	HIGHSVNKILKDIIVKSKGLS	0	0
EG10492	IELKVEQEYGKPGEKFTAAEF	0	0
EG10492	IGNGHLHKGAKPVHWCVDCRS	0	0
EG10492	ILGDEADQYVKVPDTLDVWFD	0	0
EG10492	KILKDIIVKSKGLSGYDSPYV	0	0
EG10492	KVALSKAEGEKCPRCWHYTQD	0	0
EG10492	KVIEQARADKKVGGSLEAAVT	0	0

EG10492	LAEAEVEYYDKTSPSIDVAFQ	0	0
EG10492	LANLNGFDPAKDMVKPEEMVV	0	0
EG10492	______MSDYKSTLNLPETGF	0	0
EG10492	MSSLMISTAMKGKAPYRQVLT	0	0
EG10492	NGFDPAKDMVKPEEMVVLDRW	0	0
EG10492	NKVIEQARADKKVGGSLEAAV	0	0
EG10492	NTVSPQDVMNKLGADILRLWV	0	0
EG10492	PADAQQSEVLKGLKVALSKAE	0	0
EG10492	QAVDQDALKAKFAVSNVNGPI	0	0
EG10492	QEKGALLHVEKMQHSYPCCWR	0	1
EG10492	QHSYPCCWRHKTPIIFRATPQ	0	0
EG10492	RCWHYTQDVGKVAEHAEICGR	0	0
EG10492	RTLELMEEVAKRVEVDGIQAW	0	0
EG10492	SLMISTAMKGKAPYRQVLTHG	0	0
EG10492	SNVAGDGEKRKFA________	0	0
EG10492	TEANIIRALGKIIGNGHLHKG	0	0
EG10492	TGFPMRGDLAKREPGMLARWT	0	0
EG10492	TPQWFVSMDQKGLRAQSLKEI	0	0
EG10492	TVDGQGRKMSKSIGNTVSPQD	0	0
EG10492	VEQEYGKPGEKFTAAEFRAKC	0	0
EG10492	VLDRWAVGCAKAAQEDILKAY	0	0
EG10492	VLKGLKVALSKAEGEKCPRCW	0	0
EG10492	VNKILKDIIVKSKGLSGYDSP	0	0
EG10492	VTDYTILGTVKGAELELLRFT	0	0
EG10492	VWGYLPGEREKYVFTGEWYEG	0	0
EG10492	WDCHGLPIELKVEQEYGKPGE	0	0
EG10492	WGVPMSLFVHKDTEELHPRTL	0	0
EG10492	WSHPYLTMDFKTEANIIRALG	0	0
EG10492	YGIIRAAKKGKKTFILHDGPP	0	0
EG10492	YPTLDGVNVFKANDIVVALLQ	0	0
EG10492	GEMAVSDEILKRAADSYRRIR	0	1
EG10492	NDIVVALLQEKGALLHVEKMQ	0	1
EG10492	QIDGQAVILAKDLVESVMQRI	0	1
EG10492	TLYAEPELSAKLTALGDELRF	0	1
EG10492	YAATQVDGQRKDFIRLGVLGD	0	1
EG10762	AKLYELEMQKKNAEKQAMEDN	0	0
EG10762	AVAELDALEEKLAQLEFRRMF	0	0
EG10762	CQNDRSQHKNKDQAMKQMKAK	0	0
EG10762	EESEGEVAGIKSVTIKISGDY	0	0
EG10762	ELEMQKKNAEKQAMEDNKSDI	0	0
EG10762	EVAGIKSVTIKISGDYAYGWL	0	0
EG10762	KAKLYELEMQKKNAEKQAMED	0	0
EG10762	KDQAMKQMKAKLYELEMQKKN	0	0
EG10762	KNKDQAMKQMKAKLYELEMQK	0	0
EG10762	LRGYLDYDAKKERLEEVNAEL	0	0
EG10762	NAEKQAMEDNKSDIGWGSQIR	0	0
EG10762	RSYVLDDSRIKDLRTGVETRN	0	0
EG10762	SLDQFIEASLKAGL_______	0	0

EG10762	SQHKNKDQAMKQMKAKLYELE	0	0
EG10762	TETGVHRLVRKSPFDSGGRRH	0	0
EG10762	TQCQNDRSQHKNKDQAMKQMK	0	0
EG10762	VLRGYLDYDAKKERLEEVNAE	0	0
EG10762	EAVVDTLDQMKQGLEDVSGLL	0	1
EG10762	NEPERAQALGKERSSLEAVVD	0	1
EG10770	DFAAGANIDGKHYFGINWDRD	0	0
EG10770	DHELNEVKAEKLPQVASPLTF	0	0
EG10770	DLIRNELSSYKQLPLNFYQIQ	0	1
EG10770	GVMRSREFLMKDAYSFHTSQE	0	0
EG10770	IEYKYRRNGEKQLIKTGDIVE	0	0
EG10770	LPLNFYQIQTKFRDEVRPRFG	0	0
EG10770	NIELAEAIAPKEPRAAATQEM	0	0
EG10770	QGIEVLLDDRKERPGVMFADM	0	0
EG10770	QVAILPMNMHKSFRVQELAEK	0	0
EG10770	RNLDNDDIEYKYRRNGEKQLI	0	0
EG10770	RTSQYLLSTLKETPADAEVIS	0	0
EG10770	TWLPTGVRVLKKVENIVREEM	0	0
EG10770	VRGDHELNEVKAEKLPQVASP	0	0
EG10770	WLPTGVRVLKKVENIVREEMN	0	0
EG10770	YRRNGEKQLIKTGDIVEYLVK	0	0
EG10770	ATEEEIRAVVKAGPGSLGPVN	0	1
EG10770	EMTLVDTPNAKTIAELVEQFN	0	1
EG10770	EVGHIFQLGTKYSEALKASVQ	0	1
EG10770	IEKTVKTLLVKAVEGSSFPQV	0	1
EG10770	KTGDIVEYLVKQIKG______	0	1
EG10770	LVEQFNLPIEKTVKTLLVKAV	0	1
EG10770	QFNLPIEKTVKTLLVKAVEGS	0	1
EG10770	QLMLRAGMIRKLASGLYTWLP	0	1
EG10770	SPDGQGRLLIKRGIEVGHIFQ	0	1
EG10770	TYDAMYAAYSKIFSRMGLDFR	0	1
EG10803	AEKIPSINVSKHRLDVQFDNV	0	0
EG10803	ALTEKDKADIKTAALIGVDYL	0	0
EG10803	AMARVCLGAEKIPSINVSKHR	0	1
EG10803	ASEAVNLLRDKGYLMSGDLVI	0	0
EG10803	DAGCDAKIVAKVERAEAVCSQ	0	0
EG10803	DANLGKGEGDKEKVGIDYKGL	0	0
EG10803	GDKEKVGIDYKGLPADVVPGD	0	0
EG10803	GPLSNNKGINKLGGGLSAEAL	0	0
EG10803	IGDPELVGIQKALIRRARQLN	0	0
EG10803	KIRVSTFKEGKVFLNIGDKFL	0	0
EG10803	LLLDDGRVQLKVLEVQGMKVF	0	0
EG10803	LSAEALTEKDKADIKTAALIG	0	0
EG10803	MSAMYAANHLKGVTAIITMTE	0	1
EG10803	NLGKGEGDKEKVGIDYKGLPA	0	0
EG10803	PATDRDNNLEKVIAAGANVVR	0	0
EG10803	QGPKIRVSTFKEGKVFLNIGD	0	1
EG10803	QLKVLEVQGMKVFTEVTVGGP	0	0

EG10803	RLARDAGCDAKIVAKVERAEA	0	0
EG10803	VAILGDLQGPKIRVSTFKEGK	0	0
EG10803	VTVGGPLSNNKGINKLGGGLS	0	0
EG10803	DKFLLDANLGKGEGDKEKVGI	0	1
EG10803	GGLSAEALTEKDKADIKTAAL	0	1
EG10803	__MSRRLRRTKIVTTLGPATD	0	1
EG10803	NFSHGSPEDHKMRADKVREIA	0	1
EG10803	SPEDHKMRADKVREIAAKLGR	0	1
EG10876	EGGVVDLNTLKAANIIGIQIE	0	0
EG10876	GLGKTGGRGHKGQKSRSGGGV	0	0
EG10876	GQMPLYRRLPKFGFTSRKAAI	0	0
EG10876	KTGGRGHKGQKSRSGGGVRRG	0	0
EG10876	LGRGIGSGLGKTGGRGHKGQK	0	0
EG10876	LNTLSPAEGSKKAGKRLGRGI	0	0
EG10876	NIIGIQIEFAKVILAGEVTTP	0	0
EG10876	NTLSPAEGSKKAGKRLGRGIG	0	0
EG10876	PVTVRGLRVTKGARAAIEAAG	0	0
EG10876	RLPKFGFTSRKAAITAEIRLS	0	1
EG10876	SPAEGSKKAGKRLGRGIGSGL	0	1
EG10876	TAEIRLSDLAKVEGGVVDLNT	0	1
EG10903	AGKMEGTFKRKPERSDLSADI	0	0
EG10903	DYGVQLREKQKVRRIYGVLER	0	0
EG10903	EQAPGQHGARKPRLSDYGVQL	0	0
EG10903	KPTWLEVDAGKMEGTFKRKPE	0	0
EG10903	LKSGVRAIDTKCKIEQAPGQH	0	0
EG10903	LSDYGVQLREKQKVRRIYGVL	0	0
EG10903	___MARYLGPKLKLSRREGTD	0	0
EG10903	_MARYLGPKLKLSRREGTDLF	0	0
EG10903	RAEARQLVSHKAIMVNGRVVN	0	1
EG10903	RNYYKEAARLKGNTGENLLAL	0	1
EG10903	SGVRAIDTKCKIEQAPGQHGA	0	0
EG10903	SRREGTDLFLKSGVRAIDTKC	0	0
EG10903	VDAGKMEGTFKRKPERSDLSA	0	0
EG10903	VLERQFRNYYKEAARLKGNTG	0	0
EG10903	DVVSIREKAKKQSRVKAALEL	0	1
EG10903	NDVVSIREKAKKQSRVKAALE	0	1
EG10903	NEHLIVELYSK__________	0	1
EG10903	SPNDVVSIREKAKKQSRVKAA	0	1
EG10905	AVIRSMVMRTKHAVTEASPMV	0	1
EG10905	GRRQLAYPINKLHKAHYVLMN	0	0
EG10905	HAVTEASPMVKAKDERRERRD	0	0
EG10905	QLAYPINKLHKAHYVLMNVEA	0	0
EG10905	VTEASPMVKAKDERRERRDDF	0	0
EG10954	AASFGSFADFKAQFTDAAIKN	0	0
EG10954	FKAQFTDAAIKNFGSGWTWLV	0	0
EG10954	NFGSGWTWLVKNSDGKLAIVS	0	0
EG10954	NLIKGTAFEGKSLEEIIRSSE	0	0
EG10954	SAETIEYHYGKHHQTYVTNLN	0	0

EG10954	SFELPALPYAKDALAPHISAE	0	0
EG10954	WALVNWEFVAKNLAA______	0	1
EG10954	APNAGGEPTGKVAEAIAASFG	0	1
EG10954	WTWLVKNSDGKLAIVSTSNAG	0	1
EG11192	AEVTNSAIELKVLIEQMREQI	0	0
EG11192	AHVKETYNILKKKEAVGRRLD	0	0
EG11192	EELDRLEAHVKETYNILKKKE	0	0
EG11192	ERIRSRLTRGKVECTLRYEPD	0	0
EG11192	FNRESNTLASKSINAEVTNSA	0	0
EG11192	HVKETYNILKKKEAVGRRLDF	0	0
EG11192	KQLVTAANWVKMQSDEGEINP	0	0
EG11192	RLEGVTAEVVKVRSHMPEILQ	0	0
EG11192	SMTAYARREIKGEWGSATWEM	0	0
EG11192	VARETEGQALKALIEQRLEGV	0	0
EG11192	VKETYNILKKKEAVGRRLDFM	0	0
EG11192	LQWQRERLVAKLEDAQVQLEN	0	1
EG11192	SAQGELILNEKLAKQLVTAAN	0	1
EG11223	AALVLDGIQVKRPRCQEAAQQ	0	0
EG11223	ATELADYLVAKGVPFREAHHI	0	1
EG11223	EAVVEAIRQGKPLEDLPLSEL	0	0
EG11223	GALTGMMMTLKGLPLAYNKDM	0	0
EG11223	KLIDKVGQLGKKLHTGRSRND	0	0
EG11223	LAYNKDMQEDKEGLFDALDTW	0	0
EG11223	LIDKVGQLGKKLHTGRSRNDQ	0	0
EG11223	LQSCLDKRAAKGGVSPQQVAQ	0	0
EG11223	NPDALELIRGKCGRVQGALTG	0	0
EG11223	PILSLQSCLDKRAAKGGVSPQ	0	0
EG11223	QDIVGSVAWSKALVTVGVLTA	0	0
EG11223	QVATDLKLWCKDTVSELLTAN	0	0
EG11223	RDESRLQDALKRLDVSPLGCG	0	0
EG11223	RFTQAADQRFKQFNDSLRFDY	0	0
EG11223	SRNDQVATDLKLWCKDTVSEL	0	0
EG11223	TLKGLPLAYNKDMQEDKEGLF	0	0
EG11223	TSGSSLMPQKKNPDALELIRG	0	0
EG11223	VTSGSSLMPQKKNPDALELIR	0	0
EG11223	AEDIHSWVEGKLIDKVGQLGK	0	1
EG11223	LEDLPLSELQKFSQVIDEDVY	0	1
EG11415	ILTAASRDLDKFLWFIESNIE	0	1
EG11415	IQFIDLSLITKQAHWNMRGAN	0	1
EG11415	LDIHNVQDHLKELADRYAIVA	0	0
EG11415	______MSTAKLVKSKATNLL	0	1
EG11415	___MSTAKLVKSKATNLLYTR	0	0
EG11415	_MSTAKLVKSKATNLLYTRND	0	0
EG11415	NDVRKAIGEAKDDDTADILTA	0	0
EG11415	RYAIVANDVRKAIGEAKDDDT	0	0
EG11415	ALGTTQVINSKTPLKSYPLDI	0	1
EG11415	TRNDVSDSEKKATVELLNRQV	0	1
EG11415	YTRNDVSDSEKKATVELLNRQ	0	1

EG11427	ALSMDAVQKAKSGHPGAPMGM	0	0
EG11427	ANAVGMAIAEKTLAAQFNRPG	0	0
EG11427	ANIARGGYVLKDCAGQPELIF	0	0
EG11427	AVEEARAVTDKPSLLMCKTII	0	0
EG11427	CDQVESAVAWKYGVERQDGPT	0	0
EG11427	CSLAGTLKLGKLIAFYDDNGI	0	0
EG11427	DIDGHDAASIKRAVEEARAVT	0	0
EG11427	ELKNFRQLHSKTPGHPEVGYT	0	0
EG11427	EMPSDFDAKAKEFIAKLQANP	0	0
EG11427	FGFTVDNVVAKAKELL_____	0	0
EG11427	FIAKLQANPAKIASRKASQNA	0	1
EG11427	FTVDNVVAKAKELL_______	0	0
EG11427	HEVCSLAGTLKLGKLIAFYDD	0	0
EG11427	IAEVLWRDFLKHNPQNPSWAD	0	0
EG11427	IALTREQLGWKYAPFEIPSEI	0	0
EG11427	IRALSMDAVQKAKSGHPGAPM	0	0
EG11427	KGEMPSDFDAKAKEFIAKLQA	0	0
EG11427	KTIIGFGSPNKAGTHDSHGAP	0	0
EG11427	______MSSRKELANAIRALS	0	1
EG11427	PSEIYAQWDAKEAGQAKESAW	0	0
EG11427	QEAAEFTRRMKGEMPSDFDAK	0	1
EG11427	RNAVRMAALMKQRQVMVYTHD	0	1
EG11427	TGYDLPMEELKNFRQLHSKTP	0	0
EG11427	VEAGIADYWYKYVGLNGAIVG	0	0
EG11427	VTDKPSLLMCKTIIGFGSPNK	0	0
EG11427	AYEKLTAEGVKARVVSMPSTD	0	1
EG11427	DAAYRESVLPKAVTARVAVEA	0	1
EG11427	EVELAVAAYEKLTAEGVKARV	0	1
EG11427	GQAKESAWNEKFAAYAKAYPQ	0	1
EG11427	PSNLTLWSGSKAINEDAAGNY	0	1
EG11427	QANPAKIASRKASQNAIEAFG	0	1
EG11427	QWDAKEAGQAKESAWNEKFAA	0	1
EG11427	VSMPSTDAFDKQDAAYRESVL	0	1
EG11518	EFYLVTVPDGKLSPRLAALKP	0	0
EG11518	GKLSPRLAALKPGDEVQVVSE	0	0
EG11518	GPYLSILQLGKDLDRFKNLVL	0	0
EG11518	LESTIGLPMNKETSHVMLCGN	0	1
EG11518	___MADWVTGKVTKVQNWTDA	0	1
EG11518	MADWVTGKVTKVQNWTDALFS	0	0
EG11518	MQELEKRYEGKLRIQTVVSRE	0	0
EG11518	QLLKETRQMTKHLRRRPGHMT	0	0
EG11518	QMVRDTQQLLKETRQMTKHLR	0	0
EG11518	SYLPLMQELEKRYEGKLRIQT	0	0
EG11518	VLPFTAGQFTKLGLEIDGERV	0	1
EG11615	HIHNLRDKVGKARIRTVRGFG	0	0
EG11615	ILTARDTLTDKIAGLDVGADD	0	0
EG11615	LDVGADDYLVKPFALEELHAR	0	0
EG11615	LEVHIHNLRDKVGKARIRTVR	0	0

EG11615	MGGEELILTPKEYALLSRLML	0	0
EG11615	GLHFLARIRQKKYTLPVLILT	0	1
EG11615	_________MKILIVEDDTLL	0	1
EG12204	FGFITPADGSKDVFVHFSAIQ	0	0
EG12204	HFSAIQGNGFKTLAEGQNVEF	0	0
EG12204	KGQVKWFNESKGFGFITPADG	0	0
EG12204	________MAKIKGQVKWFNE	0	0
EG12204	______MAKIKGQVKWFNESK	0	1
EG12204	NVEFEIQDGQKGPAAVNVTAI	0	1
EG12411	GEKALQEYCAKHLIFRTSWVY	0	1
EG12411	IFRTSWVYAGKGNNFAKTMLR	0	0
EG12411	LVLPDWQVGVKRMLNELFTTT	0	0
EG12411	___MNILLFGKTGQVGWELQR	0	0
EG12411	NFAKTMLRLAKEREELAVIND	0	0
EG12411	RPHNSRLNTEKFQQNFALVLP	0	0
EG12411	TAHAIRVALNKPDVAGLYHLV	0	0
EG12411	TAPLNVYGETKLAGEKALQEY	0	0
EG12411	VNAAAHTAVDKAESEPEFAQL	0	0
EG12411	VYAGKGNNFAKTMLRLAKERE	0	0
EG12411	VYGETKLAGEKALQEYCAKHL	0	0
EG12411	INATSVEAIAKAANEVGAWVI	0	1
EG12411	YAALVFEEARKAGIPLALNKL	0	1
EG12503	EALTLMPVGSKWELTIPQELA	0	1
EG12503	TDRVRVHYTGKLIDGTVFDSS	0	0
EG12503	VAGIADALEGKHPAVPVDVVH	0	0
EG12503	GVKYLEENAKKEGVNSTESGL	0	1
EG12503	RFQAMAAEGVKYLEENAKKEG	0	1
EG12545	AATLSRSQDRKVKISEIIKHT	0	0
EG12545	AGSLRIGGQTKHFLVHETQEE	0	0
EG12545	AKNKKHVFCEKPIALSYEDCV	0	0
EG12545	ARFMNMHDNAKITCVYDPENG	0	0
EG12545	CHTKRNGWENKQERLSWKKMK	0	0
EG12545	DGAIAYGHPGKKTPLWLASLI	0	1
EG12545	DRKVKISEIIKHTSVM_____	0	1
EG12545	EDCVDMVKACKEAGVTFMAGH	0	0
EG12545	ENKQERLSWKKMKEQSGGHLY	0	0
EG12545	EPVIKAAKNKKHVFCEKPIAL	0	0
EG12545	ETQEEDDDRRKGNMTSEMDGA	0	0
EG12545	GAIAYGHPGKKTPLWLASLIR	0	0
EG12545	KEPVIKAAKNKKHVFCEKPIA	0	0
EG12545	KQERLSWKKMKEQSGGHLYHH	0	0
EG12545	LFLHNILCGAKPEEDYIDLLN	0	0
EG12545	LFMTLEFPSGKLATLEWGSAF	0	0
EG12545	LHKEPVIKAAKNKKHVFCEKP	0	0
EG12545	LSYEDCVDMVKACKEAGVTFM	0	0
EG12545	MSSLDALVSSKLVDCVIVATP	0	0
EG12545	NFFNGVQYARKLIKEGVIGEI	0	1
EG12545	NGVQYARKLIKEGVIGEILSC	0	1

EG12545	PEHYVIINGTKGSIKIDMQET	0	1
EG12545	TLSRSQDRKVKISEIIKHTSV	0	0
EG12545	TPLWLASLIRKETLFLHNILC	0	0
EG12545	VIGEILSCHTKRNGWENKQER	0	0
EG12545	VIINGTKGSIKIDMQETAGSL	0	0
EG12545	WENKQERLSWKKMKEQSGGHL	0	0
EG12545	VIVATPNYLHKEPVIKAAKNK	0	1
EG12669	ETADGEYQAKKAIVCAGTWVK	0	0
EG12669	IETADGEYQAKKAIVCAGTWV	0	0
EG12669	KAIVCAGTWVKDLLPELPVQP	0	0
EG12669	LAIKTWIQLAKEAGCAQLFNC	0	0
EG12669	LIRHAYGEGEKYVPLVLRAQT	0	0
EG12669	LPELPVQPVRKVFAWYQADGR	0	1
EG12669	_________MKYDLIIIGSGS	0	1
EG12669	PAENDALKIGKHNGGQVIHSA	0	1
EG12669	QADGRYSVKNKFPAFTGELPN	0	0
EG12669	SAEQWQLNVEKLDAQGIMARW	0	0
EG12669	SGFLRSELAIKTWIQLAKEAG	0	0
EG12669	WYQADGRYSVKNKFPAFTGEL	0	0
EG12669	YGFPAENDALKIGKHNGGQVI	0	0
EG12669	EIAADFAQDKKSDFDLTPFRL	0	1
EG12669	LITGLSGHGFKFASVLGEIAA	0	1
EG12712	PDEQRVADAWKAAMEDVLKVQ	0	0
EG12712	RMEAPAVRVAKTMNTPHGDAI	0	0
EG12712	VFDLRFCVPNKEVMPERGIHT	0	0
EG12712	AWKAAMEDVLKVQDQNQIPEL	0	1
EG12712	SNEELALPKEKLQELHI____	0	1
EG12982	DPIFFVPSEGKTAAELTREEK	0	0
EG12982	EDATDQKNLQKLLETMKDVPD	0	0
EG12982	KNLQKLLETMKDVPDDQRQAR	0	0
EG12982	KTAAELTREEKSAISHRGQAL	0	1
EG12982	RYSGEDATDQKNLQKLLETMK	0	1
EG12982	SAISHRGQALKLLLDALRNG_	0	1
EG12982	GLTFIENAILKARHAAKVTAL	0	1
EG12982	KVVLATGNVGKVRELASLLSD	0	1
EG12982	NAILKARHAAKVTALPAIADD	0	1
EG13014	AIVLPALWNEKRDTKRAKLLQ	0	0
EG13014	AVISRKTTGDKQAFHSAHVQP	0	0
EG13014	DGSSIPALLKKLEEHGMTQLG	0	0
EG13014	DPWHILQTGGKEIKSAIPMGC	0	0
EG13014	EQYVTKPVDAKIQDRFAEGIL	0	0
EG13014	ESNAGAVISRKTTGDKQAFHS	0	0
EG13014	FVHTVEQYVTKPVDAKIQDRF	0	0
EG13014	GVLDQVLDALKGMDVLEFGGI	0	0
EG13014	HILQTGGKEIKSAIPMGCVLT	0	0
EG13014	ILLTLIEDGPKALKEPENYDV	0	0
EG13014	LDGSSIPALLKKLEEHGMTQL	0	0
EG13014	LHTPTRILFGKGAIAGLREQI	0	1

EG13014	PALWNEKRDTKRAKLLQYAER	0	1
EG13014	TLIEDGPKALKEPENYDVRAN	0	1
EG13014	VGGGSVLDGTKFIAAAANYPE	0	0
EG13014	WNEKRDTKRAKLLQYAERVWN	0	0
EG13014	MNAVKLVREQKVTFLLAVGGG	0	1
EG13014	PAYETLMNAVKLVREQKVTFL	0	1
EG13014	VLITYGGGSVKKTGVLDQVLD	0	1
EG13952	DEKSALAYLEKHTARLKNLGV	0	0
EG13952	FLWKVWTESEKNHEAGGIYLF	0	0
EG13952	KNLGVEEVVAKVFDVNEPLSQ	0	0
EG13952	PFGDAMAEQLKPLAESINQEP	0	0
EG13952	SINQEPGFLWKVWTESEKNHE	0	0
EG13952	NEPLSQINQAKLA________	0	1
O05598	ADVEDLLDAGKPFGLRPSAIL	0	0
O05598	APNFAYALLAKRLRRQAKPGD	0	0
O05598	EGHVVVCGRVKDVIIMAGRNI	0	0
O05598	IPMFFGAELVKVTPMDFLRDT	0	1
O05598	LLAKRLRRQAKPGDFDLSTLR	0	1
O05598	MFVGAQYDVDKDVMVSWLPCF	0	0
O05598	____MSRFTEKMFHNARTATT	0	0
O05598	QLTSGSTGSPKAVQITHRNIY	0	0
O05598	TMTVIGMIEAKAVIVSEPFLV	0	0
O05598	AALRRAVPATKGNTRRLATLG	0	1
O05598	DFLRDTLLWAKLIDKYQGTMT	0	1
O05598	DTLLWAKLIDKYQGTMTAAPN	0	1
O05598	FLVAIPILEQKGMQVLTVADL	0	1
O05598	PGTIPKTPSGKLRRANSVTLV	0	1
O05814	AADTLKNVLIKVRQPGGDTEL	0	0
O05814	DDFAKHPFLVKGYIGPKALRE	0	0
O05814	DPADAEVASHKLLIRAGYIRP	0	0
O05814	DRQASPGVKFKDAELLGMPWI	0	1
O05814	EIGHIFQLGSKYTDAFTADVL	0	1
O05814	FPLTLYQIQTKYRDEARPRAG	0	0
O05814	FTADVLGEDGKPVRLTMGSYG	0	0
O05814	GILRAREFVMKDSYSFDIDAA	0	0
O05814	GRTVTAADTLKNVLIKVRQPG	0	0
O05814	GVPGDREVDDKRLGAALEPAD	0	0
O05814	LDDRQASPGVKFKDAELLGMP	0	0
O05814	LTVKGEYSSYKDFPLTLYQIQ	0	0
O05814	PFLVKGYIGPKALRENNVRYL	0	0
O05814	THEELFTLTVKGEYSSYKDFP	0	0
O05814	TQYGDSVFRLKDRRGNDYLLG	0	0
O05814	YALLDDDDFAKHPFLVKGYIG	0	0
O05814	PFDVHLVIANKDAQARAGATA	0	1
O06188	AHCPVAVMRSKVDQPASDINW	0	0
O06188	AVPLRLVSVIKPTHPSPDDYD	0	0
O06188	_MSGRGEPTMKTIIVGIDGSH	0	1
O06188	AVLEYAAREAKLRQAPILALG	0	1

O06188	EAQSAVEAAGKLVKIETDIPR	0	1
O06188	LGSTATELAEKAHCPVAVMRS	0	1
O06391	VWTLRLYLTGKRLWHDPRRRD	0	0
O33294	AGTILHTGDIKFDQLPPDGRP	0	0
O33294	ASISGAVPVGKMFVDGLIAGD	0	0
O33294	DHIGAIPFLLKLRPDIPVVGS	0	0
O33294	DPKALEPAVRKVEAELESLVA	0	0
O33294	GTWRMLRANAKLAASTGVPQE	0	1
O33294	IAQGVRRTVGKWVGETYRRQP	0	0
O33294	LRPDIPVVGSKFTLALVAEKC	0	0
O33294	NGVSVDLVAGKASISGAVPVG	0	0
O33294	SKFTLALVAEKCREYRITPVF	0	0
O33294	AVFGVIDALSKIGARVVTNAQ	0	1
O33341	FVLAVQWHPEKSLDDLRLFKA	0	1
O53176	CGLALALDRDKLSDMRGVLTP	0	0
O53176	RMAQDGDPGYKATSVLLGECG	0	1
O53176	TDTNCVVRSFKGGFSGGTIAS	0	0
O53176	VLYGATGFVGKLTAEYLARAG	0	0
O53176	YIRLLPRGLVKRVVPKPGTGP	0	0
O53176	PRGLVKRVVPKPGTGPSAAAR	0	1
O53204	AARPHRVTHRKLTRG______	0	0
O53204	AFGFKGPDQQKPAGVLSGGER	0	0
O53204	AWEGDDDNEAKWFWFEGNFGA	0	0
O53204	DQARAGIDPRKTVWEVVSDGL	0	0
O53204	DVTLSFYPGAKIGVVGPNGAG	0	0
O53204	EFIYTMKKVRKAHGDKVILDD	0	0
O53204	EGNFGAYEENKVERLGVDAAR	0	0
O53204	EGNYSTYLEKKAERLAVQGRK	0	0
O53204	EMAAEAEKTRKLDFEEIQIPV	0	0
O53204	GGERRRVALCKLLLSKPDLLL	0	0
O53204	GNVEEGMGDIKIKLDRFNEVA	0	1
O53204	IGVVGPNGAGKSSVLRIMAGL	0	0
O53204	LQQEPPLNEDKTVRGNVEEGM	0	0
O53204	__MAEFIYTMKKVRKAHGDKV	0	0
O53204	_MAEFIYTMKKVRKAHGDKVI	0	0
O53204	MKKVRKAHGDKVILDDVTLSF	0	0
O53204	PNGVGKTTLFKTIVGLETPDS	0	0
O53204	RNRLNLALTLKQGGNLILLDE	0	0
O53204	RVALCKLLLSKPDLLLLDEPT	0	0
O53204	RYEEMAAEAEKTRKLDFEEIQ	0	0
O53204	SGAKARQAKSKARLQRYEEMA	0	0
O53204	SRAYVSAFGFKGPDQQKPAGV	0	0
O53204	SVLRIMAGLDKPNNGDAFLAT	0	1
O53204	VEEGMGDIKIKLDRFNEVAEL	0	1
O53204	VGVIGPNGVGKTTLFKTIVGL	0	0
O53204	VQGRKDAKLQKRLTEELAWVR	0	0
O53204	YEGNYSTYLEKKAERLAVQGR	0	0
O53204	EELAWVRSGAKARQAKSKARL	0	1

O53204	GLETPDSGSVKVGETVKLSYV	0	1
O53204	KAERLAVQGRKDAKLQKRLTE	0	1
O53204	NVVVEVDHLDKGYDGRALIKD	0	1
O53204	RLAVQGRKDAKLQKRLTEELA	0	1
O53204	SGSVKVGETVKLSYVDQARAG	0	1
O53204	VRSGAKARQAKSKARLQRYEE	0	1
O53226	GAYGEKQMYGKTVQGVIRSTF	0	0
O53226	GKIVVAQYNVKATGHVAKLRR	0	0
O53226	GLNVVGISPDKPEKLATFRDA	0	0
O53226	________MTKTTRLTPGDKA	0	0
O53226	RSTFVVDEDGKIVVAQYNVKA	0	0
O53226	VLTAWGAYGEKQMYGKTVQGV	0	0
O53226	VVGISPDKPEKLATFRDAQGL	0	0
O53226	YPAASTPGCTKQACDFRDNLG	0	0
O53442	AWGLAGEPSLKQFVTG_____	0	0
O53442	LGADIEAYRDKLQNVADAGIF	0	0
O53442	_______MAQKPVADALTLEL	0	0
O53442	NEDVRVQHVMKGYRAEKYTQV	0	0
O53442	QNVADAGIFGKPQLRQLISDR	0	1
O53442	TDACEILLILKDNLAGHHREL	0	0
O53618	GAELANYRRNKVGIVFQAFNL	0	1
O53618	LSCLGGILRPKSGAIKFDEVD	0	0
O53618	VMLLGPSGCGKTTLLSCLGGI	0	0
O53618	ELADGGEELVKVAGPGDYFGE	0	1
O53665	ALEGFTRSLGKELRRGATTAL	0	0
O53665	ATGITEPAGLKGLHEFFTPVL	0	0
O53665	DDSTPPADWEKPLDGKVAIVT	0	0
O53665	ESTMRFLLSAKSAYVDGQVFS	0	0
O53665	LDVTADDAVDKISEHLRDHHG	0	1
O53665	LVNNAGITRDKLLANMDDARW	0	1
O53665	_______MAPKRSSDLFSQVV	0	0
O53665	NRGQTNYATTKAGMIGITQAL	0	0
O53665	PADWEKPLDGKVAIVTGAARG	0	0
O53665	SEHLRDHHGGKADILVNNAGI	0	0
O53665	AAENLAETASKVGGTALWLDV	0	1
O53665	VVEPLRAALEKDYDLVGNNLG	0	1
O53871	AFASVVLKFQKDLNIPDEKLN	0	1
O53871	DTTKEGLAKLKPAFEGLAALG	0	0
O53871	EAIRTPRGKQKNGSLHEVKPL	0	0
O53871	FQKDLNIPDEKLNVNGGAIAM	0	0
O53871	HDEHMRPDTTKEGLAKLKPAF	0	0
O53871	IYEAIRTPRGKQKNGSLHEVK	0	0
O53871	KQKNGSLHEVKPLSLVVGLID	0	0
O53871	LGGFDDVALQKYHWVEKINHV	0	0
O53871	LNEAFASVVLKFQKDLNIPDE	0	0
O53871	LVMIGSAAAGKLQGLTPRARI	0	0
O53871	LVVGLIDELRKRHPDLDENLI	0	0
O53871	MLTGPTPATRKVLDRAGLTVD	0	1

O53871	RPDTTKEGLAKLKPAFEGLAA	0	0
O53871	VALQKYHWVEKINHVHTGGNS	0	0
O53871	GLEAVNTAAQKVRSGWDDLVL	0	1
O53871	VDAYALRSQQKAAEAWSGGYF	0	1
O69687	IFTATGQHPLKFTAYDGSTAG	0	0
O69687	KNYPFYFGFLKSKLRTGGLLL	0	0
O69687	LKTLTERVDFKRPSARVLANV	0	0
O69687	NLQLHHVLATKVDPRGDDSLP	0	0
O69687	RRMANGLLHSKTRDAEAIHHH	0	0
O69687	SIGLTEHIGVKNYPFYFGFLK	0	0
O69687	VHPGDPYELLKTLTERVDFKR	0	0
O69687	YPFYFGFLKSKLRTGGLLLNH	0	0
O69687	AVAEVGLPTAKVWGLYMAASR	0	1
O69687	EASLEQAQENKYRLIFEKLRL	0	1
O69687	LSAEQAKWGQKAVEDEGLSDL	0	1
O69687	QENKYRLIFEKLRLEPGDRLL	0	1
O86352	MTEPAAWDEGKPRIITLTMNP	0	0
O86352	QHISSGVFLLKASVRELRECV	0	0
O86352	SFTVNESRTAKQYRFVLPGPS	0	1
O86352	TSVDVVRPTEKMRCGAPRYDP	0	0
P09621	AKVNIKPLEDKILVQANEAET	0	0
P09621	ASGLVIPDTAKEKPQEGTVVA	0	0
P09621	GLVIPDTAKEKPQEGTVVAVG	0	0
P09621	________MAKVNIKPLEDKI	0	0
P09621	____MAKVNIKPLEDKILVQA	0	0
P09621	VAEGDTVIYSKYGGTEIKYNG	0	0
P09621	GPGRWDEDGEKRIPLDVAEGD	0	1
P09621	IYSKYGGTEIKYNGEEYLILS	0	1
P0A4X0	AAMSATEPLTKTTITRRDPGP	0	0
P0A4X0	GANFTYNSIGKDGQPTQGGYS	0	0
P0A4X0	GICHSDIHTVKAEWGQPNYPV	0	1
P0A4X0	GLGGLGHMGVKLGAAMGADVT	0	0
P0A4X0	PGPHDVAIDIKFAGICHSDIH	0	0
P0A4X0	SCTRGIEQYCKPGANFTYNSI	0	0
P0A4X0	YATADPDTFRKLRGGFDLILN	0	0
P0A4X0	DVTVLSQSLKKMEDGLRLGAK	0	1
P0A4X0	KMEDGLRLGAKSYYATADPDT	0	1
P0A4X0	VVTAVGSEVTKYRQGDRVGVG	0	1
P0A508	AAEVGYPVLIKPSAGGGGKGM	0	0
P0A508	GDVVVVVEAMKMEHSLEAPVS	0	0
P0A508	IEVMGDKIAAKNAVAAFDVPV	0	0
P0A508	LIKPSAGGGGKGMRLVQDPAR	0	0
P0A508	PARAIEVMGDKIAAKNAVAAF	0	0
P0A508	RECSLQRRHQKVIEEAPSPLL	0	0
P0A508	TWHLREAEEHKIHRAVGARPA	0	0
P0A508	VGSDYDPLLTKVIAHGADREE	0	1
P0A508	PARESYLDIGKVLDAAARTGA	0	1
P0A508	QVLVSVGDQVKVEQVLARIKD	0	1

P0A508	VKVEQVLARIKD________	0	1
P0A520	AVVADKPEKEKASVPGGGDMG	0	0
P0A520	FLTTEAVVADKPEKEKASVPG	0	0
P0A520	GDEATGANIVKVALEAPLKQI	0	0
P0A520	IVKVALEAPLKQIAFNSGLEP	0	0
P0A520	KVTETLLKGAKEVETKEQIAA	0	0
P0A520	LKRGIEKAVEKVTETLLKGAK	0	0
P0A520	LLKGAKEVETKEQIAATAAIS	0	0
P0A520	LQAAPTLDELKLEGDEATGAN	0	0
P0A520	PLLEKVIGAGKPLLIIAEDVE	0	0
P0A520	TEAVVADKPEKEKASVPGGGD	0	1
P0A520	TVKDLLPLLEKVIGAGKPLLI	0	0
P0A520	ADLSLLGKARKVVVTKDETTI	0	1
P0A520	LENADLSLLGKARKVVVTKDE	0	1
P0A520	RGLNALADAVKVTLGPKGRNV	0	1
P0A520	SGLEPGVVAEKVRNLPAGHGL	0	1
P0A556	AFKIAGSQVLKKAAALAQPVI	0	0
P0A556	AGHIYAVIGLKDTTTGDTLSD	0	0
P0A556	ANVGKPQVAYKETIKRLVQNV	0	0
P0A556	ATKGKKERLGKLFQMHSNKEN	0	0
P0A556	DPVIEVAIEPKTKSDQEKLSL	0	0
P0A556	DQEKLSLSIQKLAEEDPTFKV	0	0
P0A556	EEGATYEFESKVTGGRIPREY	0	0
P0A556	FGIMAHIDAGKTTTTERILYY	0	0
P0A556	FKIAGSQVLKKAAALAQPVIL	0	0
P0A556	FKIATHPFFGKLTYIRVYSGT	0	1
P0A556	HEVDSSEMAFKIAGSQVLKKA	0	0
P0A556	IEPKTKSDQEKLSLSIQKLAE	0	0
P0A556	ILVDRMRREFKVEANVGKPQV	0	0
P0A556	ITSAATTTFWKDNQLNIIDTP	0	0
P0A556	IYPVLCGSAFKNKGVQPMLDA	0	0
P0A556	KPQVAYKETIKRLVQNVEYTH	0	0
P0A556	LDGAVAVFDGKEGVEPQSEQV	0	0
P0A556	LGKLFQMHSNKENPVDRASAG	0	0
P0A556	LVQNVEYTHKKQTGGSGQFAK	0	0
P0A556	MFGYVGDLRSKTQGRANYSMV	0	0
P0A556	PRICFVNKMDKIGADFYFSVR	0	0
P0A556	PVLCGSAFKNKGVQPMLDAVV	0	0
P0A556	QKLAEEDPTFKVHLDSETGQT	0	0
P0A556	QSEQVWRQADKYDVPRICFVN	0	0
P0A556	REFKVEANVGKPQVAYKETIK	0	0
P0A556	RILYYTGINYKIGEVHDGAAT	0	0
P0A556	RLVQNVEYTHKKQTGGSGQFA	0	1
P0A556	TVDEIKGAIRKLTIASEIYPV	0	0
P0A556	VAESDEHLLEKYLGGEELTVD	0	0
P0A556	VIEVAIEPKTKSDQEKLSLSI	0	0
P0A556	VLAGYPLVNLKVTLLDGAYHE	0	0
P0A556	VPPAIGHAPAKEDEEVVRKAT	0	0

P0A556	YDVPRICFVNKMDKIGADFYF	0	0
P0A556	ESGSQVINATKGKKERLGKLF	0	1
P0A556	GGEELTVDEIKGAIRKLTIAS	0	1
P0A556	GSQVINATKGKKERLGKLFQM	0	1
P0A556	GVVDLVEMNAKVWRGETKLGE	0	1
P0A556	KQTGGSGQFAKVIINLEPFTG	0	1
P0A556	LAEQAEEYRTKLLEVVAESDE	0	1
P0A556	_______MAQKDVLTDLSRVR	0	1
P0A556	MNAKVWRGETKLGETYDTVEI	0	1
P0A556	PANVSKEIIAKATGE______	0	1
P0A556	SQVINATKGKKERLGKLFQMH	0	1
P0A556	SYSEVPANVSKEIIAKATGE_	0	1
P0A556	TDEPFAALAFKIATHPFFGKL	0	1
P0A5B7	IMVRDGQLTIKAERTEQKDFD	0	1
P0A5B7	TRLMRLEDEMKEGRYEVRAEL	0	0
P0A5B7	VAVSEGKPTEKHIQIRSTN__	0	0
P0A5B7	DEDDIKATYDKGILTVSVAVS	0	1
P0A5F7	EWRNLEDEQEKMLARRLSGWQ	0	0
P0A5F7	FEYSRFQEAQKREIVEHSYLV	0	0
P0A5F7	QDRYPDVVVHKVVVCDRPAPR	0	0
P0A5H3	AAEERGYTATKHQREVGAGYF	0	0
P0A5H3	AYNCSPSFNWKKHLDDATIAK	0	0
P0A5H3	ERTREGFYRTKNGIEPCIARA	0	0
P0A5H3	GGALNVYELQKALIAAGVAGS	0	0
P0A5H3	HWEDQLASEKKCGHLGGKVLI	0	0
P0A5H3	MAVQQVRAGLKAIYLSGWQVA	0	0
P0A5H3	NALQRADQIAKIEGDTSVENW	0	0
P0A5H3	NGIEPCIARAKAYAPFADLIW	0	0
P0A5H3	SEKKCGHLGGKVLIPTQQHIR	0	1
P0A5H3	SHWEDQLASEKKCGHLGGKVL	0	0
P0A5H3	YNCSPSFNWKKHLDDATIAKF	0	0
P0A5H3	EAARQFSEAVKAEYPDQMLAY	0	1
P0A5H3	FQKELAAMGFKFQFITLAGFH	0	1
P0A5H3	KKHLDDATIAKFQKELAAMGF	0	1
P0A5H3	___MSVVGTPKSAEQIQQEWD	0	1
P0A5H3	QQEWDTNPRWKDVTRTYSAED	0	1
P0A5L2	AVGGAKNSVLKLMAATLLAEG	0	0
P0A5L2	CVLGPLVGRCKRARVALPGGD	0	0
P0A5L2	LSGEVAVGGAKNSVLKLMAAT	0	0
P0A5L2	SFRVTQYERPKAVNVATLPFP	0	0
P0A5L2	DPAHLQLVLHKLHDAGATVTQ	0	1
P0A5U4	AGIYGKLAWEKTIPNWFFEPD	0	0
P0A5U4	ALELAVAQIEKSYGKGSVMRL	0	0
P0A5U4	AVAQIEKSYGKGSVMRLGDEA	0	0
P0A5U4	FGSPETTTGGKALKFYASVRM	0	0
P0A5U4	GAIVWATPDHKVLTEYGWRAA	0	0
P0A5U4	GDGRDGWVGGKTPINFINVQR	0	0
P0A5U4	KQAEFDILYGKGISREGSLID	0	0

P0A5U4	__MTQTPDREKALELAVAQIE	0	0
P0A5U4	NRTRVKVVKNKCLAEGTRIFD	0	0
P0A5U4	PETTTGGKALKFYASVRMDVR	0	0
P0A5U4	QARLMSQALRKMTGALNNSGT	0	0
P0A5U4	RMDVRRVETLKDGTNAVGNRT	0	0
P0A5U4	SIVNGRRIQSKRQVFEVRISG	0	0
P0A5U4	STVRDYDPTQKRPSIVNGRRI	0	0
P0A5U4	TAIFINQLRDKIGVMFGSPET	0	0
P0A5U4	TNAVGNRTRVKVVKNKCLAEG	0	0
P0A5U4	TYEGEQLGQGKENARNFLVEN	0	0
P0A5U4	VASGDPRGGMKQVLGASRLRR	0	1
P0A5U4	VGNRTRVKVVKNKCLAEGTRI	0	0
P0A5U4	VQALADALDDKFLHDMLAEEL	0	0
P0A5U4	VVVHNCSPPFKQAEFDILYGK	0	0
P0A5U4	YGWRAAGELRKGDRVAQPRRF	0	0
P0A5U4	ADEIEKKIKEKLGIGAVVTDD	0	1
P0A5U4	AEHALDPDYAKKLGVDTDSLL	0	1
P0A5U4	DLCQQAGIYGKLAWEKTIPNW	0	1
P0A5U4	DMGVDQGLIRKSGAWFTYEGE	0	1
P0A5U4	DVADEIEKKIKEKLGIGAVVT	0	1
P0A5U4	EHALDPDYAKKLGVDTDSLLV	0	1
P0A5U4	HRIEDVVDGRKPIHVVAAAKD	0	1
P0A5U4	IEIYGPESSGKTTVALHAVAN	0	1
P0A5U4	NADVADEIEKKIKEKLGIGAV	0	1
P0A5U4	RKPIHVVAAAKDGTLHARPVV	0	1
P0A5Z4	ADVEIDVPAHKVTRNGEQISL	0	0
P0A5Z4	DDYIMKPFKPKELVARVRARL	0	0
P0A5Z4	EFDLLVALARKPRQVFTRDVL	0	0
P0A5Z4	GADDYIMKPFKPKELVARVRA	0	0
P0A5Z4	LESGADDYIMKPFKPKELVAR	0	0
P0A5Z4	SGVPIVMLTAKTDTVDVVLGL	0	0
P0A5Z4	VVLTVRGVGYKAGPP______	0	0
P0A5Z4	VNVHVQRLRAKVEKDPENPTV	0	1
P0CG95	LTLPASVDLEKETVITGRVVD	0	0
P0CG95	_____MCSGPKQGLTLPASVD	0	0
P0CG99	AFKYYTSYTLKTFDGKRYLER	0	0
P0CG99	AQIYAWRKGIKTLYYIRLRQM	0	0
P0CG99	ASKIEIRKEGKIGRVYYPAPY	0	1
P0CG99	ATPTFLNSGKKQRGEPVSCFL	0	0
P0CG99	DIHIPTQDDWKQLKESVQKHG	0	1
P0CG99	DQGLSLTLFFKDTATTRDVNK	0	0
P0CG99	DWKQLKESVQKHGIYNQNLQA	0	0
P0CG99	ERGTHFKGFEKSKYASGEFFD	0	0
P0CG99	FRFPTFLGAFKYYTSYTLKTF	0	0
P0CG99	GTHFKGFEKSKYASGEFFDKY	0	0
P0CG99	HPDIYRFLDTKRENADEKIRI	0	0
P0CG99	HPVASKIEIRKEGKIGRVYYP	0	0
P0CG99	IPTQDDWKQLKESVQKHGIYN	0	0

P0CG99	IQFEKDREAAKQYFLQHVNQN	0	0
P0CG99	KDTATTRDVNKAQIYAWRKGI	0	0
P0CG99	KIAIERGTHFKGFEKSKYASG	0	0
P0CG99	LDTKRENADEKIRIKTLSLGV	0	0
P0CG99	MVDDARIRKTKINAREFFQTL	0	0
P0CG99	NDDLSYAKVGKDISCNLGSLN	0	0
P0CG99	NQSSGVIPIMKLLEDSFSYAN	0	1
P0CG99	NTVFFHSQDEKLDYLIENEYY	0	0
P0CG99	RSINSALQLSKRGGGVALLLS	0	0
P0CG99	SCNLGSLNIAKAMDSPDFAQT	0	0
P0CG99	SEFNDDLSYAKVGKDISCNLG	0	0
P0CG99	SKYASGEFFDKYTDQVWEPKT	0	0
P0CG99	SNIREHGAPIKNIENQSSGVI	0	0
P0CG99	STSSIHPVASKIEIRKEGKIG	0	0
P0CG99	TDQVWEPKTDKVRRLFADADI	0	0
P0CG99	TSYTLKTFDGKRYLERFEDRV	0	0
P0CG99	TVNRSNPIAGKITHSNLCSEI	0	0
P0CG99	VNKAQIYAWRKGIKTLYYIRL	0	0
P0CG99	VPFADVSVTEKYYEMVDDARI	0	0
P0CG99	YDADGKIQFEKDREAAKQYFL	0	0
P0CG99	YEMVDDARIRKTKINAREFFQ	0	1
P0CG99	AMLNLYDADGKIQFEKDREAA	0	1
P0CG99	DKYTDQVWEPKTDKVRRLFAD	0	1
P0CG99	IKSLLDRAYAKKFRFPTFLGA	0	1
P0CG99	KSLLDRAYAKKFRFPTFLGAF	0	1
P0CG99	LAAGDTELAEKLVDEIIDGRF	0	1
P0CG99	PATPTFLNSGKKQRGEPVSCF	0	1
P0CG99	RENADEKIRIKTLSLGVVIPD	0	1
P0CG99	VIPDITFELAKKNEDMYLFSP	0	1
P0CG99	VLDQYSRDFIKSLLDRAYAKK	0	1
P0CG99	VLFHALRASNKIAIERGTHFK	0	1
P0CG99	YQDAYEIGYEKIIDTYAAATQ	0	1
P0CH36	GICHSDIHTVKAEWGVPNYPV	0	0
P0CH36	GLGGLGHVAVKLAKAMGADVT	0	0
P0CH36	LDLGAYLGLLKLDGALVELGL	0	0
P0CH36	LRHWNAGPGKKVAVIGLGGLG	0	0
P0CH36	PLRHWNAGPGKKVAVIGLGGL	0	0
P0CH36	TEVGSEVTKYKVGDRVGVGCF	0	0
P0CH36	VDSCRECDNCKAGLEQYCTGT	0	1
P0CH36	VVTEVGSEVTKYKVGDRVGVG	0	0
P0CH36	YATSDPETFDKLAGSFDLILN	0	0
P0CH36	AATSATEPLTKTTITRRAVGP	0	1
P0CH36	DVTVLSQSLKKMEDGLRLGAS	0	1
P0CH36	GLGHVAVKLAKAMGADVTVLS	0	1
P17670	AGHVNHTIWWKNLSPNGGDKP	0	0
P17670	AIADAFGSFDKFRAQFHAAAT	0	0
P17670	AVAKLEEARAKEDHSAILLNE	0	0
P17670	EDHSAILLNEKNLAFNLAGHV	0	0

P17670	HAFYLQYKNVKVDFAKAFWNV	0	0
P17670	MWEHAFYLQYKNVKVDFAKAF	0	0
P17670	QYKNVKVDFAKAFWNVVNWAD	0	0
P17670	YVKGANDAVAKLEEARAKEDH	0	0
P17670	AALGWDTLGNKLLIFQVYDHQ	0	1
P17670	HHSKHHATYVKGANDAVAKLE	0	1
P17670	SGQINELHHSKHHATYVKGAN	0	1
P17670	WKNLSPNGGDKPTGELAAAIA	0	1
P60176	ADIVVTATGNKDIIMLEHIKA	0	0
P60176	ADLSLADFGRKELRIAEHEMP	0	0
P60176	DKWTKIAESVKGVTEETTTGV	0	0
P60176	DVGKGCAEAMKGQGARVSVTE	0	0
P60176	EPKGVPVFAWKGETLEEYWWA	0	0
P60176	EQMLTWPDPDKPANMILDDGG	0	0
P60176	ERSGATRVNVKPQVDLWTFGD	0	0
P60176	EYDNEVYRLPKHLDEKVARIH	0	0
P60176	HVEALGGHLTKLTKEQAEYLG	0	0
P60176	IIMLEHIKAMKDHAILGNIGH	0	0
P60176	LRTRFETDKDKWTKIAESVKG	0	0
P60176	MLVLRGMQYEKAGVVPPAEED	0	0
P60176	___MTGNLVTKNSLTPDVRNG	0	0
P60176	NKDIIMLEHIKAMKDHAILGN	0	0
P60176	NLLRTRFETDKDKWTKIAESV	0	0
P60176	NRGTDALIGGKKVLICGYGDV	0	1
P60176	PAEEDDPAEWKVFLNLLRTRF	0	0
P60176	RFETDKDKWTKIAESVKGVTE	0	0
P60176	RGTDALIGGKKVLICGYGDVG	0	0
P60176	RREYAEVQPLKGARISGSLHM	0	0
P60176	VGPHGTPDEPKGVPVFAWKGE	0	0
P60176	VLICGYGDVGKGCAEAMKGQG	0	0
P60176	VYRLPKHLDEKVARIHVEALG	0	0
P60176	YLGVDVEGPYKPDHYRY____	0	0
P60176	QTIAQIELWTKNDEYDNEVYR	0	1
P60176	TPDVRNGIDFKIADLSLADFG	0	1
P60796	EGVFVGSGIFKSGAPEHRAAA	0	0
P60796	GNPATGTARVKRGMAEMLKGG	0	0
P60796	IAAVTIPVMAKVRIGHFVEAQ	0	0
P60796	ISEGAAMIRSKGEAGTGDVSN	0	0
P60796	MSEDELFVAAKELQAPYELVA	0	0
P60796	TPADYAHHIDKWNFTVPFVCG	0	0
P60796	APEHRAAAIVKATTFFDDPDV	0	1
P60796	ELVAEVARAGKLPVTLFTAGG	0	1
P63345	AKAVANSLAKKMAEVRGDDAH	0	0
P63345	ARNSKLMETLKEARQQLLALR	0	0
P63345	CSPNIDAASLKKGQTVRLNEA	0	0
P63345	DAEAAQDIYSKYLTEFLPVHA	0	0
P63345	DTVDVFTSGRKMRLTCSPNID	0	1
P63345	EFDGDRSACIKAMIEKVVDRM	0	0

P63345	EVRGDDAHEAKSYFLNIKGPE	0	0
P63345	HEAKSYFLNIKGPELLNKFVG	0	0
P63345	LYREYSLRPPKGVLLYGPPGC	0	0
P63345	RDAVELPFLHKELYREYSLRP	0	0
P63345	RIDSLAARNSKLMETLKEARQ	0	0
P63345	RSACIKAMIEKVVDRMYAEID	0	0
P63345	SPNIDAASLKKGQTVRLNEAL	0	0
P63345	AMIQNVVDRAKKNAIKSVLET	0	1
P63345	EALNDDTRPRKLRPGDSLLVD	0	1
P63345	MIQNVVDRAKKNAIKSVLETG	0	1
P63345	VVDRAKKNAIKSVLETGQPGL	0	1
P63458	ELARRCVLAGKDVIVAGHSVG	0	0
P63458	ELRGVPARAVKSPADLDELAN	0	0
P63458	LAATRGAEMAKACATEPTGMS	0	0
P63458	PPAGTLSGIAKRELRGVPARA	0	0
P63458	TATLLSNRDGKPVTSAAAAMD	0	0
P63458	GAADQIAAWSKAADLDLARLG	0	1
P63458	LEKLAEDPPAKARVRALGVAG	0	1
P63523	AISVPVDHTEKLVTVIELKLL	0	0
P63523	EDEMFIVGRMKDMLIVYGRNH	0	0
P63523	GDNVAEGYWNKPDETRHTFGA	0	0
P63523	GEAMDELDVIKNNVTAAISRS	0	0
P63523	LTGNSPSFRVKDLPSAAYLQY	0	0
P63523	PAGTIGEIWVKGDNVAEGYWN	0	0
P63523	PGSIPTTTSGKIRRAACVEQY	0	0
P63523	TEKLVTVIELKLLGDSAGEAM	0	0
P63523	LYVASRNSGDKPEVVYFEPDK	0	1
P63523	PNFAFELAVRKTTDADIEGLD	0	1
P63523	STGSANRCEPKTGTPLLSYGM	0	1
P63568	FKLSRLTGRTKLVAAHDAFHG	0	0
P63568	GITPDVVTLAKGLGGGLPIGA	0	0
P63568	GSLALTGQPAKQTPFAPLPGD	0	0
P63568	LGIALTAPHAKDAEATARDAG	0	1
P63568	SGAEANEAAFKLSRLTGRTKL	0	1
P63673	AIAQHWMYEGKHVLIIFDDLT	0	0
P63673	AVKVKKPAPKKKK________	0	0
P63673	DEDKLAKEAVKVKKPAPKKKK	0	0
P63673	DEHVEALDEDKLAKEAVKVKK	0	0
P63673	DKLAKEAVKVKKPAPKKKK__	0	0
P63673	EAVKVKKPAPKKKK_______	0	0
P63673	ERGARLVELLKQPQSQPMPVE	0	0
P63673	FENIEEGQQVKRTGEVLSVPV	0	0
P63673	GQRQLIIGDRKTGKTAVCVDT	0	0
P63673	HVLIIFDDLTKQAEAYRAISL	0	0
P63673	KLAKEAVKVKKPAPKKKK___	0	0
P63673	KVKKPAPKKKK__________	0	0
P63673	LHSRLLERCAKLSDDLGGGSL	0	0
P63673	QLIIGDRKTGKTAVCVDTILN	0	0

P63673	QRQNWESGDPKKQVRCVYVAI	0	0
P63673	RQNWESGDPKKQVRCVYVAIG	0	0
P63673	SLTGLPIIETKANDISAYIPT	0	0
P63673	VEALDEDKLAKEAVKVKKPAP	0	0
P63673	VGGAAQIKAMKEVAGSLRLDL	0	0
P63673	VKVKKPAPKKKK_________	0	0
P63673	VSRVGGAAQIKAMKEVAGSLR	0	0
P63673	AAAASESAGFKWLAPYTGSAI	0	1
P63673	AFASDLDAASKAQLERGARLV	0	1
P63673	APSVVHRQGVKEPLQTGIKAI	0	1
P63673	DKLTEVIKNFKKGFAATGGGS	0	1
P63673	EAADKLTEVIKNFKKGFAATG	0	1
P63673	EILTEIRDSQKLTEEAADKLT	0	1
P63673	GVKEPLQTGIKAIDAMTPIGR	0	1
P63673	KLTEVIKNFKKGFAATGGGSV	0	1
P63673	VRCVYVAIGQKGTTIAAVRRT	0	1
P64245	AVTNQWVNSYKRLVQGGEAPT	0	0
P64245	DDPLQLSEVGKSFIAGILEHA	0	0
P64245	DNVMTFRYVIKEVALEEGARA	0	0
P64245	EEGARASFMPKPFGQHPGSAM	0	0
P64245	EHVFDFFLRNKRTEWANYRSH	0	1
P64245	LVRVPMYTPHKTSSRRVEVRS	0	0
P64245	LWFTDVLGFLKSVAIAPAELE	0	0
P64245	VHPEIEFFLLKPGPEDGSVPV	0	0
P64245	PRHVLRRQLTKAGELGFSCYV	0	1
P65161	KQLNKLINVIKIVEQDDEHSV	0	0
P65161	LTVEATGNRGKLEALLRVLEP	0	0
P65161	SRGPRGIGTAK__________	0	0
P65161	THTLSVLVEDKPGVLARVAAL	0	0
P65161	AEDTPLEQITKQLNKLINVIK	0	1
P65161	HSVSRELALIKVQADAGSRSQ	0	1
P65161	_______MSPKTHTLSVLVED	0	1
P65232	ISARLIADLLKTAGADRIVTV	0	0
P65232	LGGVPLAFIHKTRDPRVPNQV	0	0
P65232	MIDALKRGSAKRITAVMPFYP	0	0
P65232	MPFYPYARQDKKHRGREPISA	0	0
P65232	_MSHDWTDNRKNLMLFAGRAH	0	0
P65232	PDSGRVRIAEKWADALGGVPL	0	0
P65232	PFYPYARQDKKHRGREPISAR	0	0
P65232	MEQLIMIDALKRGSAKRITAV	0	1
P65232	VTNTLPIGEDKRFPQLTVLSI	0	1
P65277	EAFEGACAYWKPRTLPAP___	0	0
P65277	GLDDIALTLRKLDEIEAFEGA	0	0
P65277	TDQIIPAVFLKRVTRTGFEDG	0	0
P65277	FGDIFRGNAGKAGLLAAEVAQ	0	1
P65277	VAQDDVELLWKLIEQSPGLEI	0	1
P65573	LVDSLRSDTPKAPTRGAPLCG	0	0
P65573	MGGDAIFDRLKEHLGVGHDET	0	0

P65573	PTLAGLQVARKNDMQAPPTPG	0	0
P65880	ACEFKNQVLVKIYNRKALDPA	0	0
P65880	DKVTERYMGSKKIGTTGRGIG	0	0
P65880	DVLDPEQLTHKVEAACEFKNQ	0	0
P65880	GAQWGDEGKGKATDLLGGRVQ	0	0
P65880	GLQDRGVDTAKLLISADAHLL	0	0
P65880	GRGIGPCYQDKIARIGIRVAD	0	0
P65880	KVTERYMGSKKIGTTGRGIGP	0	0
P65880	LIGAQWGDEGKGKATDLLGGR	0	0
P65880	LLMPYHIAIDKVTERYMGSKK	0	0
P65880	PMTQRDLCRAKPVYEELPGWW	0	0
P65880	THKVEAACEFKNQVLVKIYNR	0	0
P65880	TRIGTVLGILKAYTTRVGSGP	0	0
P65880	VNGITDYFLTKLDVLSSLESV	0	0
P65880	DALLEQAEGFKHRIADTRLLL	0	1
P65880	GAREFDDLPAKARDYVLRLEQ	0	1
P65880	NQVLVKIYNRKALDPAQVVDA	0	1
P66056	APKKKVAGLIKLQIVAGQANP	0	1
P66056	DQVREIAETKKTDLNANDVDA	0	1
P66056	GVAKGSAEPHKTKVAKVTWDQ	0	0
P66056	KLLLKAAGVAKGSAEPHKTKV	0	0
P66056	KTPPAAKLLLKAAGVAKGSAE	0	0
P66056	LNANDVDAAAKIIAGTARSMG	0	0
P66056	_______MAPKKKVAGLIKLQ	0	0
P66056	______MAPKKKVAGLIKLQI	0	0
P66056	_____MAPKKKVAGLIKLQIV	0	0
P66056	QHGVNIMEFCKAYNAATENQR	0	0
P66056	TFTLKTPPAAKLLLKAAGVAK	0	0
P66056	WDQVREIAETKKTDLNANDVD	0	0
P66056	YEDRSFTFTLKTPPAAKLLLK	0	0
P66056	AKGSAEPHKTKVAKVTWDQVR	0	1
P66902	GNAGNITAYWKGYTEYHQLGL	0	0
P66902	KLAQAVMHGAKIIQIDGNFDD	0	0
P66902	NGLKDPDTALKDMPSVSPVPV	0	0
P66902	SKQTGCTIHLKVEGLNPTGSF	0	1
P66902	SVNPVRIEGQKTAAFEIVDVL	0	1
P66902	TSAVEAQQQSKGRFLAASDEE	0	1
P66902	VEGLNPTGSFKDRGMTMAVTD	0	0
P66902	VVCTVTGNGLKDPDTALKDMP	0	0
P66902	ASAASIAGLLKAIDDGWVARG	0	1
P66902	TEYHQLGLIDKLPRMLGTQAA	0	1
P66902	TPLIAATNLSKQTGCTIHLKV	0	1
P66902	VPVDPVAVVEKLGLA______	0	1
P69440	DTDDVILNRMKVYRDETAPLL	0	0
P69440	EEGTKLGVEAKRYLDAGDLVP	0	0
P69440	LFRRNIEEGTKLGVEAKRYLD	0	0
P69440	PLLEYYRDQLKTVDAVGTMDE	0	0
P69440	VLLLGPPGAGKGTQAVKLAEK	0	0

P69440	VSEEVLLERLKGRGRADDTDD	0	0
P69440	KGTQAVKLAEKLGIPQISTGE	0	1
P69440	PGAGKGTQAVKLAEKLGIPQI	0	1
P69440	VFARALRALGK__________	0	1
P71703	AIFASENNTIKIADVAPTNVI	0	0
P71703	ARVPIVGDDIKSQVGATITHR	0	0
P71703	AVTSNLKREFKTKDVHIGPSD	0	0
P71703	FGDVPLNLEYKLEVWDSPNSA	0	0
P71703	FIASDPVWAKKFTDARVPIVG	0	0
P71703	GATITHRVLAKLFEDRGVQLD	0	0
P71703	IDAVRAAKIAKDRGIGGPVIP	0	0
P71703	MLERERLESKKISKTQAVTSN	0	0
P71703	NMLERERLESKKISKTQAVTS	0	0
P71703	PVIPASAYLMKSPPEQLPDDI	0	0
P71703	QRGPTLDGIGKYYADTIELSD	0	1
P71703	RERLESKKISKTQAVTSNLKR	0	1
P71703	RFGPYHVRDVKFVAAFDVDAK	0	0
P71703	SDHVGWLDDRKWAYVRLEGRA	0	0
P71703	SKTQAVTSNLKREFKTKDVHI	0	1
P71703	TSNLKREFKTKDVHIGPSDHV	0	1
P71703	VDVVQALKEAKVDVLVSYLPV	0	1
P71703	VFIASDPVWAKKFTDARVPIV	0	0
P71703	AEPVDVVQALKEAKVDVLVSY	0	1
P71703	FVAAFDVDAKKVGFDLSDAIF	0	1
P71703	GVIIDAVRAAKIAKDRGIGGP	0	1
P71703	YLPVGSEEADKFYAQCAIDAG	0	1
P71724	LIAALSAALLKLPVANWPALG	0	0
P71724	DLRRAYDTAVKLGERTGLVVR	0	1
P71973	AEHGMAARGYKDVRANTVPAF	0	0
P71973	VGLHRPAEFNKSHIPAFLAGE	0	0
P77899	GVLPYLRPDGKTQVTIAYEDN	0	0
P77899	ILEIGYDSSDKGFDGATCGVN	0	0
P77899	_______MSEKGRLFTSESVT	0	0
P77899	RLSRRLTEVRKNGVLPYLRPD	0	0
P77899	TQHAADIDLEKTLDPDIREKV	0	0
P77899	TVRVLVNPTGKFVLGGPMGDA	0	0
P77899	DVELPWEQLDKVDDLKRAI__	0	1
P77899	EKTLDPDIREKVLNTVLDDLA	0	1
P77899	HVVGEVTTSAKEAFADITNTV	0	1
P77899	TETEDPVKIEKAIGEVFDLRP	0	1
P77899	WEQLDKVDDLKRAI_______	0	1
P96382	PGTRMRSDTPKVLHTLAGRSM	0	0
P96382	QRNIENWVQRKRPGSPAAQAS	0	0
P96382	RPGSPAAQASKRASEMACQQP	0	0
P96382	SVFVNYDGTSKRRTTVGSHVR	0	0
P96382	MLSHVLHAIAKLAPQRLIVVL	0	1
P96382	RPGTALGADGKLGAFVEVKNS	0	1
P96382	VKNSTIGTGTKVPHLTYVGDA	0	1

P96825	AGILRDGTFHKMSFENWDAVL	0	0
P96825	ATEDGAANIIKTALDEFGAVH	0	0
P96825	LFGNDGANFDKPPSVQDVAAR	0	0
P96825	LINTLALEGAKYNIHANALAP	0	0
P96825	MSFENWDAVLKVHLYGGYHVL	0	0
P96825	NASVYVVGGGKVQRVALFGND	0	0
P96825	NFGQTNYGAAKLGLVGLINTL	0	0
P96825	QDILPPEVLEKLTPEFVAPVV	0	0
P96825	ADEVVAEIRDKGGRAVANYDS	0	1
P96825	DLSGAKIAGFKL_________	0	1
Q10504	AKTIKGYALGKHFEGRNATHQ	0	0
Q10504	ATTMATVRTFKEVLRDKQIGP	0	0
Q10504	AVDADLMLAYKESEVGQILHE	0	0
Q10504	EGRNATHQMKKLTLEDLKEFR	0	0
Q10504	EVGFNHFFRGKSHPGGGDQVF	0	0
Q10504	FEGRNATHQMKKLTLEDLKEF	0	0
Q10504	FNHYLHDRGIKDTSDQHVWCF	0	0
Q10504	GFVPERRTKSKALTLPGRDIY	0	0
Q10504	HKGQPTVILAKTIKGYALGKH	0	0
Q10504	LGGFVPERRTKSKALTLPGRD	0	0
Q10504	LPGRDIYAPLKKGSGHQEVAT	0	1
Q10504	NLKRGGHDYRKVYAAYRAAVD	0	0
Q10504	NMSDQDIWNLKRGGHDYRKVY	0	0
Q10504	NRDGVAIETEKLRHPDRPAGV	0	0
Q10504	PGRDIYAPLKKGSGHQEVATT	0	0
Q10504	QMKKLTLEDLKEFRDTQRIPV	0	0
Q10504	QPTVILAKTIKGYALGKHFEG	0	0
Q10504	TTPDGDYQTYKANDGGYVRDH	0	0
Q10504	VRTFKEVLRDKQIGPRIVPII	0	0
Q10504	YAAYRAAVDHKGQPTVILAKT	0	0
Q10504	YHAATEQRTNKAQILASGVAM	0	0
Q10504	FFRGAGWNVIKVVWGREWDAL	0	1
Q10530	ALTDDYFIERKLYPNVDFYTG	0	0
Q10530	DDVSEFVRKVKNREAGVKLMG	0	0
Q10530	GGDDSLLGIAKELEEAALTDD	0	0
Q10530	KLMGFGHRVYKNYDPRARIVK	0	0
Q10530	KLPTIAAYAYKKSVGQPFLYP	0	0
Q10530	LPTIAAYAYKKSVGQPFLYPD	0	0
Q10530	QRHTMLHEDLKRFFDGFPRNA	0	0
Q10530	RGYPIDQLAEKSTFIEVCYLL	0	0
Q10530	RKVKNREAGVKLMGFGHRVYK	0	0
Q10530	VELSTIRLLAKLPTIAAYAYK	0	0
Q10530	VKEQADKILAKLGGDDSLLGI	0	0
Q10530	WREMHDEGDSKIGRPRQIYTG	0	0
Q10530	DGIALGPLLAKTGHTTFDVGF	0	1
Q10530	DVGFANTAAAKSSITYIDGDA	0	1
Q10530	KNYDPRARIVKEQADKILAKL	0	1
Q10530	SGDDVSEFVRKVKNREAGVKL	0	1

Q10682	ARVITTGGSQKAVNADRSGLA	0	1
Q50685	AIACKDPEKKKKLMTDIFALA	0	0
Q50685	AYVLEGGSARKLIEAREKAFW	0	0
Q50685	DFDQVMAAVPKILASGLAPDI	0	0
Q50685	DGNVHMAIACKDPEKKKKLMT	0	0
Q50685	EAREKAFWAAKALGADDIIDT	0	0
Q50685	GLLISFDRMNKVLEVDTANQV	0	0
Q50685	GPPQKPAYAAKPATPEEVAQL	0	0
Q50685	GTNAGGMRAVKYGVARHNVLG	0	0
Q50685	GYFLELEDPVKISLMRRIKQS	0	0
Q50685	HDEELTGPPQKPAYAAKPATP	0	0
Q50685	HMAIACKDPEKKKKLMTDIFA	0	0
Q50685	IACKDPEKKKKLMTDIFALAM	0	0
Q50685	ISGEHGVGRAKTGYFLELEDP	0	1
Q50685	MAIACKDPEKKKKLMTDIFAL	0	0
Q50685	PVKISLMRRIKQSFDPAGILN	0	0
Q50685	ATPEEVAQLLKAASENGVPVT	0	1
Q50685	EIIRTGGRMAKVSTGYDLTQL	0	1
Q50685	SARKLIEAREKAFWAAKALGA	0	1
Q50685	TLALVTEVIVKLHPRLDHNAS	0	1
Q7D8W0	GGSSKDRIAVKMIEAAEASGQ	0	0
Q7D8W0	GPEIWADTEGKVTHFVAGIGT	0	0
Q7D8W0	GTITGAGRYLKEVSGGRVRIV	0	0
Q7D8W0	GYKCVFVCPDKVSEDKRNVLI	0	0
Q7D8W0	IEAAEASGQLKPGGTIVEPTS	0	0
Q7D8W0	LALVAQRRGYKCVFVCPDKVS	0	0
Q7D8W0	LPDGGRGYMSKIFNDAWMSSY	0	0
Q7D8W0	RLVRDIDGAWKPDQYANPEGP	0	0
Q7D8W0	SCGMAVVAALKVAEEAGPDAL	0	0
Q7D8W0	VEYLNPGGSSKDRIAVKMIEA	0	0
Q7D8W0	WDALMVVEEGKPVGVITRYDL	0	0
Q7D8W0	FVCPDKVSEDKRNVLIAYGAE	0	1
Q7D8W0	LLSAVFEGRAKLADAVSAHMS	0	1
Q7D8W0	QSTVGDVLRRKSGALPALVHT	0	1
Q7D8W0	VPDGAGTVAAKVEYLNPGGSS	0	1

Table S5. The 10-fold cross-validation prediction results of SVM balance on the training dataset.

Protein Name	Peptide	Class label	Prediction label
A0QNF6	FGEVVDEESQKVVDAIASTPT	1	1
A0QP32	DYFAHWINVGKNADESKLPKV	1	1
A0QP90	AGATRIIGVAKAGLDDAGYRN	1	1
A0QPN2	ANADTVTLWSKLALESARARR	1	1
A0QQ65	NMKYADGTPIKASDFTYAIQR	1	1
A0QQH7	LFDEHGAYLAKTGGEVGVTTG	1	1
A0QQU5	AVEKVTETLLKSAKEVETKEQ	1	1
A0QQU5	VAAGANPLGLKRGIEKAVEKV	1	0
A0QS45	SGEPHKTKVAKVTWDQVREIA	1	1

A0QS98	KALEGDPKWVKSVEELMEAVD	1	1
A0QSE0	KRWRLVEILEKAK________	1	1
A0QSH8	KGTQAEKLSEKLGIPQISTGD	1	1
A0QSL6	SGHVGFKGSRKSTPFAAQLAA	1	1
A0QSU4	KIAQVVEVAAKEPEPSAAIRL	1	1
A0QUV7	GRGVGSAENFKIVEELADSLG	1	1
A0QUX7	QSLAVGATEQKDMSRMTIVVS	1	1
A0QUY3	GQTEAAARVDKAVSEHLATRG	1	0
A0QUY9	RVVADVLRGRKVADGVRMLIV	1	0
A0QUZ0	SFILNLSPFDKGSVLVAGPDF	1	0
A0QV10	ITSIASLETGKRLGPDPRTFN	1	1
A0QVB9	ALVEADGDFDKAVELLRIKGA	1	1
A0QVB9	ANDIETLKAAKTGDTTVEQAI	1	0
A0QVB9	IADLSAKIGEKLELRRAAYFD	1	0
A0QVM0	YSFDVDDDGLKNAYYQHREAY	1	1
A0QW25	DVIDEAATFLKQQEDKGVVVR	1	1
A0QWG8	TFYDDPDVLAKVSRGLGEAMV	1	1
A0QWT3	TFGSETVDPAKIEKAIGEVFD	1	1
A0QWT3	WEQTNKVDDLKSAI_______	1	1
A0QWV9	GAARRLGVSAKAREVRGSDRV	1	1
A0QWW2	SVTDLTAELAKSASVEDINAA	1	1
A0QWX9	KTILQAIELWKIVDRPNLLIK	1	1
A0QWX9	VDTEIDARLEKIGSDEALALR	1	1
A0QX20	ELTDAKNAFRKDIHNYVEQNH	1	0
A0QX81	GGVDVDAVVPKVRPIVDAVAQ	1	1
A0QX93	ATEEEDVLLEKELLADEKERA	1	1
A0QXX7	NSWPDNVSLDKARRLLWPIKQ	1	0
A0QYD4	ARRDDLEVAAKELDIESIVFD	1	1
A0QYS9	DLVARAGVLGKTLSHGIEELG	1	1
A0QZ54	IVYIRTLVTGKSSSASRAIDT	1	1
A0QZA1	SVARTLIDASKSAQMVVVGNR	1	0
A0QZE3	IHIEQGRELEKNNVTIGLVDR	1	1
A0R066	ERKIDVDEWQKKAGAGEITEV	1	1
A0R079	KTSDDIFKLIKDENVEYVDIR	1	1
A0R083	LLAAGLRGVEKGYVLGPQAED	1	1
A0R0B0	DHFFGRDPRTKALVADMSDQE	1	1
A0R0B2	VAAGAVAALDKLAEDPPAKAR	1	1
A0R0B3	AVQTEDKYGVKIPDEDLAGLR	1	0
A0R0B3	TVGDVVAYIQKLEEENPEAAA	1	1
A0R0B4	IRVLEDEFVTKWDLPVRIGGH	1	0
A0R0B5	VELRRLSYLQKMATVLGRRVW	1	1
A0R0W1	RAQVLVPELEKRLDDINAQLD	1	1
A0R0W4	DVADRIAVLNKGRIEQIGSPT	1	1
A0R1B5	AIEVVYGADVKGRGIESAAPL	1	1
A0R1V9	LTAVVGGDLSKVDAKQPDDYF	1	1
A0R1V9	PDDYFTRVTSKDYEGKWRIIF	1	1
A0R1V9	VGGDLSKVDAKQPDDYFTRVT	1	1
A0R1Y7	QSHQKAAAAWKDGVFADEVVP	1	0

A0R218	DVYVSMNMVRKNGLRRGDAVT	1	0
A0R220	NFDDCLELARKLTADFPTIAL	1	1
A0R2G5	NSLSGAVEVVKRDGTGITLNE	1	1
A0R2V7	NAQALADGFVKRDAGLVTGGT	1	1
A0R2W6	IGRQAIEGFYKNMTAATTDIK	1	0
A0R342	HPDIPAAATVKPGSEFRIECK	1	1
A0R3D2	LTANVRTRTGKGASRQARRDG	1	1
A0R3D6	VVVLGPGSIPKTPSGKLRRAN	1	0
A0R4C9	RDFVDAQQFSKLLSERGIAND	1	1
A0R4D0	TGAGIHEVDVKVA________	1	1
A0R4Z5	DEGPRETTLEKMAALQPLVEG	1	1
A0R518	EDLAETVELVKGLNRRIVAEE	1	1
A0R566	MPNSNPVAAWKALKDGNARFV	1	1
A0R5E1	SGFRTLEENQKVEFEVGQSPK	1	1
A0R5M8	FDLDAFEHVAKWNSDEERIEM	1	1
A0R5R5	LSREQATWAQKAIAQEGLTDL	1	0
A0R5R7	EILEDRLGESKIASWTDPKGG	1	1
A0R635	HNETVVLDFPKRVVREPIPDE	1	1
A0R647	_MTNSGALDTKFHALIQDQIR	1	1
A0R652	HHSKHHATYVKGVNDAIAKLE	1	1
A0R652	WKNLSPNGGDKPTGELAAAID	1	1
A0R6E3	PATEAETAQIKDLIAALGG__	1	0
A0R6E3	RAAVVLSGLRKLQGSLAEAAS	1	1
A0R6Q0	IKTADVDAAAKEIEATGGAII	1	1
A0R7F9	VIRKDGGTVDKVDIWGRRRLA	1	1
A0R7G6	NFVAAFDVDAKKVGFDLSEAI	1	1
A4ZHR8	ALGGTLTKLSKDQAEYIGVDV	1	1
EG10025	APFAGTVKEIKVNVGDKVSTG	1	0
EG10207	FADYDEARFQKEGFRVVPPAA	1	1
EG10251	DVKILDPRVGKEFPLPTYATS	1	0
EG10367	SVVDLTVRLEKAATYEQIKAA	1	1
EG10407	QEIFTREEMIKYFTLNAVSKS	1	1
EG10410	LAMQVVVLRDKLEPYFTEGRY	1	1
EG10416	SAEQVDPRDEKVANLEAQLAE	1	1
EG10416	TRERDGILRVKAEMENLRRRT	1	1
EG10489	_______MESKVVVPAQGKKI	1	1
EG10489	WLKVKNPNTGKEIVIKDVIAD	1	1
EG10492	MNDAFWDELLKVRGEVNKVIE	1	1
EG10762	YLRWAESRGFKTEIIEESEGE	1	0
EG10770	DIVEYLVKQIKG_________	1	0
EG10770	KSFRVQELAEKLYSELRAQGI	1	1
EG10770	QLGTKYSEALKASVQGEDGRN	1	0
EG10803	EGKVFLNIGDKFLLDANLGKG	1	1
EG10803	RADKVREIAAKLGRHVAILGD	1	1
EG10876	ARAAIEAAGGKIEE_______	1	1
EG10903	AALELAEQREKPTWLEVDAGK	1	1
EG10903	REKAKKQSRVKAALELAEQRE	1	1
EG10905	YTAAITGAEGKIHRLEDWGRR	1	1

EG10954	TYVTNLNNLIKGTAFEGKSLE	1	1
EG11192	GELILNEKLAKQLVTAANWVK	1	0
EG11223	HSWVEGKLIDKVGQLGKKLHT	1	1
EG11415	TQVINSKTPLKSYPLDIHNVQ	1	1
EG11427	AWNEKFAAYAKAYPQEAAEFT	1	1
EG11427	FDAKAKEFIAKLQANPAKIAS	1	0
EG11518	LQLGKDLDRFKNLVLVHAARY	1	1
EG11615	EYALLSRLMLKAGSPVHREIL	1	0
EG11615	LHFLARIRQKKYTLPVLILTA	1	1
EG12204	__MAKIKGQVKWFNESKGFGF	1	0
EG12411	RKAGIPLALNKLNAVPTTAYP	1	0
EG12503	EGVKYLEENAKKEGVNSTESG	1	1
EG12545	PNYLHKEPVIKAAKNKKHVFC	1	1
EG12669	GEIAADFAQDKKSDFDLTPFR	1	0
EG12712	INSNEELALPKEKLQELHI__	1	1
EG12982	________MQKVVLATGNVGK	1	1
EG13014	LITYGGGSVKKTGVLDQVLDA	1	1
EG13952	AGGIYLFTDEKSALAYLEKHT	1	1
EG13952	AYLEKHTARLKNLGVEEVVAK	1	0
O05598	VVVLGPGTIPKTPSGKLRRAN	1	1
O05814	YSFDIDAAGLKAAYHAHREAY	1	1
O06188	SAVEAAGKLVKIETDIPRGPA	1	0
O06391	IAARMTTAVDKARVRGAGHEV	1	1
O33294	LHSRGFSEDPKALEPAVRKVE	1	1
O33341	EKSLDDLRLFKALVDAASGYA	1	0
O53176	FMRNSIDLYHKQAADTGARIV	1	1
O53204	DKGYDGRALIKDLSFSLPRNG	1	0
O53226	TKTTRLTPGDKAPAFTLPDAD	1	1
O53226	YNVKATGHVAKLRRDLSV___	1	1
O53442	QHVMKGYRAEKYTQVETLVYM	1	1
O53618	GILRPKSGAIKFDEVDITTLQ	1	0
O53665	TALVYLSPDAKPAATGLESTM	1	1
O53665	TQALAPGLAAKGITINAVAPG	1	1
O53871	AEAWSGGYFAKSVVPVRDQNG	1	0
O69687	IGATLSAEQAKWGQKAVEDEG	1	0
O86352	VGLSRGWSLIKSVRLGNAAGA	1	1
P09621	SARDVLAVVSK__________	1	1
P0A508	EWQLRVGAGEKLGFAQNDIEL	1	1
P0A520	AVEKVTETLLKGAKEVETKEQ	1	1
P0A556	PAKEDEEVVRKATTDEPFAAL	1	0
P0A5B7	LPVGADEDDIKATYDKGILTV	1	1
P0A5B7	LTIKAERTEQKDFDGRSEFAY	1	0
P0A5B7	LTVSVAVSEGKPTEKHIQIRS	1	1
P0A5B7	RAELPGVDPDKDVDIMVRDGQ	1	1
P0A5F7	QAHQIVEQAHKVALEASSSGR	1	1
P0A5H3	LDDATIAKFQKELAAMGFKFQ	1	1
P0A5L2	VARITAPDEPKYDADFAAVRQ	1	1
P0A5U4	ENADVADEIEKKIKEKLGIGA	1	1

P0A5Z4	HVQRLRAKVEKDPENPTVVLT	1	1
P0CG95	SGAGIHEVDVKIT________	1	0
P0CG99	IPDITFELAKKNEDMYLFSPY	1	1
P0CH36	ADVTVLSQSLKKMEDGLRLGA	1	1
P17670	SRYAAATSQTKGLIFG_____	1	1
P60176	ALGGHLTKLTKEQAEYLGVDV	1	1
P60796	TFFDDPDVLAKVSRGLGEAMV	1	1
P63458	VAAGRLTALEKLAEDPPAKAR	1	1
P63523	KPEVVYFEPDKLSTGSANRCE	1	0
P63568	GLVRRAEVLGKSLRHGIEALG	1	1
P63673	SQKLTEEAADKLTEVIKNFKK	1	1
P65161	ESLAVGATECKDRSRMTIVVS	1	1
P65232	AHPELAEQVAKELDVHVTSQD	1	1
P65277	ITAATVVLPFKIDDHSAWRLL	1	1
P65573	DVLARLEVDAKEIIGRYPDRR	1	0
P65880	LFDEHGEYLSKTGREFGVTTG	1	1
P66056	SAEPHKTKVAKVTWDQVREIA	1	1
P66902	NFDDCLELARKMAADFPTISL	1	1
P69440	DGYPRSVEQAKALHEMLERRG	1	1
P71703	KFVAAFDVDAKKVGFDLSDAI	1	0
P71724	QAVAAAEVLGKRQPLLIVSSD	1	1
P71973	AIIDDASTFFKQQEERGVVVR	1	1
P77899	TFGTETEDPVKIEKAIGEVFD	1	1
P96382	DGKLGAFVEVKNSTIGTGTKV	1	0
P96825	WAEITDLSGAKIAGFKL____	1	1
Q10504	DHFFGRDPRTKALVENMSDQD	1	0
Q10530	RARIVKEQADKILAKLGGDDS	1	1
Q10682	VVAVGFTFDPKTHIARVITTG	1	1
Q50685	DTVVPRASMPKFLSTARGLAA	1	1
Q7D8W0	GAGELVSAAGKALRDWDALMV	1	1
A0QNF6	ATLHTNRGDIKIALFGNHAPK	0	1
A0QNF6	DEFHPELQFDKPYLLAMANAG	0	0
A0QNF6	KIALFGNHAPKTVNNFVGLAQ	0	1
A0QNF6	NGSQFFITVGKTPHLNRRHTI	0	0
A0QNF6	NNFVGLAQGTKDYSTENASGG	0	1
A0QNF6	TGTGRGGPGYKFEDEFHPELQ	0	1
A0QP32	AGNTVFTNVAKTDDGDVWWEG	0	0
A0QP32	AGTFQKLNPEKQPNSYLALSD	0	0
A0QP32	AHLVEAGTFQKLNPEKQPNSY	0	0
A0QP32	CMGPLDAEDPKLGVEITDSEY	0	1
A0QP32	DKLGDDGFFVKALHSIGAPLE	0	1
A0QP32	EGDPQHLIDWKGNDWTPESGE	0	0
A0QP32	FVGEKLPTGLKDEFDALKHRL	0	0
A0QP32	GDDIAWMRFGKDGRLYATNPE	0	0
A0QP32	GKNADESKLPKVFFVNWFRRG	0	1
A0QP32	GNDWTPESGEKAAHPNSRYCT	0	1
A0QP32	GSEQTAAAEGKVGTVRRDPMA	0	1
A0QP32	GWLAEHMLILKLISPENKAYF	0	1

A0QP32	GYGGNALLGKKCYSLRIASAM	0	0
A0QP32	IAAAFPSACGKTNLAMLQPTI	0	0
A0QP32	IEEWFEFVGEKLPTGLKDEFD	0	0
A0QP32	INVGKNADESKLPKVFFVNWF	0	0
A0QP32	ISAILFGGRRKTTVPLITEAR	0	1
A0QP32	KDVPWPCNDTKYITHFPETRE	0	0
A0QP32	LILKLISPENKAYFIAAAFPS	0	0
A0QP32	LKWAVERIEHKADGKSTPIGI	0	0
A0QP32	MTRMGRAALDKLGDDGFFVKA	0	1
A0QP32	PADVDEALAVKPEEWRAELPL	0	1
A0QP32	PGFGENSRVLKWAVERIEHKA	0	0
A0QP32	SGYGGNALLGKKCYSLRIASA	0	1
A0QP32	SIGAPLEPGQKDVPWPCNDTK	0	0
A0QP32	TGLKDEFDALKHRLSEEG___	0	1
A0QP32	TIPGLDTAPTKHQGLLAWVQE	0	0
A0QP32	TNWSSNPNAMKTIAAGNTVFT	0	0
A0QP32	VERIEHKADGKSTPIGIVPTA	0	0
A0QP90	AGVPFYLRTGKRMSQRFSEIV	0	0
A0QP90	DEAIRLEMTAKEPGSGGRLRP	0	0
A0QP90	FGGTGDLAARKLLPALYLRDR	0	1
A0QP90	HSRTETFVAVKAHIRNWRWAG	0	1
A0QP90	HVNRESVRDEKRKVLEALEPL	0	1
A0QP90	NRESVRDEKRKVLEALEPLTA	0	1
A0QP90	QIFRIDHYLGKESVQQLLVTR	0	1
A0QP90	QRFSEIVVQFKPVPLPMFPGI	0	0
A0QP90	VTESSRVVLEKPIGRDLASAQ	0	1
A0QPN2	APDVAWDSQAKTAVSSRSLNL	0	0
A0QPN2	ARAGGKFSAHKDATSTGAGLN	0	0
A0QPN2	ARARRSAEGAKARARAASKAK	0	1
A0QPN2	ASRMRDLGLAKNEEGPEPQDF	0	0
A0QPN2	CEGDSALGTIKAARDATFQAA	0	0
A0QPN2	DATFQAAFPLKGKPPNVYGFP	0	0
EG10025	DAAPAQAEEKKEAAPAAAPAA	0	0
EG10025	DEVEITEILVKVGDKVEAEQS	0	1
EG10025	DEVEVTEILVKVGDKVEAEQS	0	1
EG10025	DEVEVTEVMVKVGDKVAAEQS	0	0
EG10025	DVQAYVKEAIKRAEAAPAATG	0	1
EG10025	EEAAKRKLDVKITPVVFIMKA	0	0
EG10025	EGAAPAAAPAKQEAAAPAPAA	0	0
EG10025	ELKVNVGDKVKTGSLIMIFEV	0	0
EG10025	ELSRELMTISKKARDGKLTAG	0	0
EG10025	EQSLITVEGDKASMEVPAPFA	0	0
EG10025	EQSLITVEGDKASMEVPSPQA	0	0
EG10025	EVPAPFAGTVKEIKVNVGDKV	0	1
EG10025	EVPAPFAGVVKELKVNVGDKV	0	1
EG10025	EVPSPQAGIVKEIKVSVGDKT	0	0
EG10025	FRKQQNEEAAKRKLDVKITPV	0	0
EG10025	GEAGAAAPAAKQEAAPAAAPA	0	0

EG10025	GGIPGMLPWPKVDFSKFGEIE	0	0
EG10025	IEEVELGRIQKISGANLSRNW	0	1
EG10025	ILREDVQAYVKEAIKRAEAAP	0	0
EG10025	ITEILVKVGDKVEAEQSLITV	0	1
EG10025	KQQNEEAAKRKLDVKITPVVF	0	0
EG10025	KSAMEPVWNGKEFVPRLMLPI	0	0
EG10025	LAREFGVNLAKVKGTGRKGRI	0	0
EG10025	LSEDGQRLTLKKYINIGVAVD	0	0
EG10025	LSRELMTISKKARDGKLTAGE	0	1
EG10025	LVVPVFKDVNKKGIIELSREL	0	0
EG10025	_____MAIEIKVPDIGADEVE	0	0
EG10025	MLPWPKVDFSKFGEIEEVELG	0	0
EG10025	MTISKKARDGKLTAGEMQGGC	0	1
EG10025	NLAKVKGTGRKGRILREDVQA	0	1
EG10025	PAAAPAAAAAKDVNVPDIGSD	0	1
EG10025	QEAAAPAPAAKAEAPAAAPAA	0	1
EG10025	REFGVNLAKVKGTGRKGRILR	0	1
EG10025	SEDGQRLTLKKYINIGVAVDT	0	0
EG10025	SPQAGIVKEIKVSVGDKTQTG	0	0
EG10025	TDITELEAFRKQQNEEAAKRK	0	1
EG10025	TPNGLVVPVFKDVNKKGIIEL	0	0
EG10025	VKEIKVNVGDKVSTGSLIMVF	0	0
EG10025	VKEIKVSVGDKTQTGALIMIF	0	0
EG10025	VKELKVNVGDKVKTGSLIMIF	0	0
EG10025	VKITPVVFIMKAVAAALEQMP	0	1
EG10025	VMIPHVTHFDKTDITELEAFR	0	1
EG10025	VTEILVKVGDKVEAEQSLITV	0	1
EG10025	VTEVMVKVGDKVAAEQSLITV	0	1
EG10025	VVPVFKDVNKKGIIELSRELM	0	1
EG10207	ATVGSCAQIGKNVHLSGGVGI	0	0
EG10207	CAVIVKKVDAKTRGKVGINEL	0	0
EG10207	DGQWVTHQWLKKAVLLSFRIN	0	1
EG10207	ESRYFDKVPMKFADYDEARFQ	0	1
EG10207	GQWVTHQWLKKAVLLSFRIND	0	0
P63458	TATLLSNRDGKPVTSAAAAMD	0	0
P63523	AISVPVDHTEKLVTVIELKLL	0	0
P63523	EDEMFIVGRMKDMLIVYGRNH	0	0
P63523	GDNVAEGYWNKPDETRHTFGA	0	0
P63523	GEAMDELDVIKNNVTAAISRS	0	1
P63523	LTGNSPSFRVKDLPSAAYLQY	0	1
P63523	LYVASRNSGDKPEVVYFEPDK	0	1
P63523	PAGTIGEIWVKGDNVAEGYWN	0	0
P63523	PGSIPTTTSGKIRRAACVEQY	0	0
P63523	PNFAFELAVRKTTDADIEGLD	0	1
P63523	STGSANRCEPKTGTPLLSYGM	0	0
P63523	TEKLVTVIELKLLGDSAGEAM	0	1
P63568	FKLSRLTGRTKLVAAHDAFHG	0	0
P63568	GITPDVVTLAKGLGGGLPIGA	0	0

P63568	GSLALTGQPAKQTPFAPLPGD	0	1
P63568	LGIALTAPHAKDAEATARDAG	0	0
P63568	SGAEANEAAFKLSRLTGRTKL	0	0
P63673	AAAASESAGFKWLAPYTGSAI	0	0
P63673	AFASDLDAASKAQLERGARLV	0	1
P63673	AIAQHWMYEGKHVLIIFDDLT	0	0
P63673	APSVVHRQGVKEPLQTGIKAI	0	0
P63673	AVKVKKPAPKKKK________	0	0
P63673	DEDKLAKEAVKVKKPAPKKKK	0	0
P63673	DEHVEALDEDKLAKEAVKVKK	0	0
P63673	DKLAKEAVKVKKPAPKKKK__	0	1
P63673	DKLTEVIKNFKKGFAATGGGS	0	0
P63673	EAADKLTEVIKNFKKGFAATG	0	1
P63673	EAVKVKKPAPKKKK_______	0	1
P63673	EILTEIRDSQKLTEEAADKLT	0	1
P63673	ERGARLVELLKQPQSQPMPVE	0	0
P63673	FENIEEGQQVKRTGEVLSVPV	0	1
P63673	GQRQLIIGDRKTGKTAVCVDT	0	0
P63673	GVKEPLQTGIKAIDAMTPIGR	0	1
P63673	HVLIIFDDLTKQAEAYRAISL	0	0
P63673	KLAKEAVKVKKPAPKKKK___	0	0
P63673	KLTEVIKNFKKGFAATGGGSV	0	1
P63673	KVKKPAPKKKK__________	0	0
P63673	LHSRLLERCAKLSDDLGGGSL	0	0
P63673	QLIIGDRKTGKTAVCVDTILN	0	0
P63673	QRQNWESGDPKKQVRCVYVAI	0	1
P63673	RQNWESGDPKKQVRCVYVAIG	0	0
P63673	SLTGLPIIETKANDISAYIPT	0	0
P63673	VEALDEDKLAKEAVKVKKPAP	0	1
P63673	VGGAAQIKAMKEVAGSLRLDL	0	0
P63673	VKVKKPAPKKKK_________	0	0
P63673	VRCVYVAIGQKGTTIAAVRRT	0	0
P63673	VSRVGGAAQIKAMKEVAGSLR	0	1
P64245	AVTNQWVNSYKRLVQGGEAPT	0	0
P64245	DDPLQLSEVGKSFIAGILEHA	0	0
P64245	DNVMTFRYVIKEVALEEGARA	0	1
Q50685	ATPEEVAQLLKAASENGVPVT	0	0
Q50685	AYVLEGGSARKLIEAREKAFW	0	0
Q50685	DFDQVMAAVPKILASGLAPDI	0	1
Q50685	DGNVHMAIACKDPEKKKKLMT	0	0
Q50685	EAREKAFWAAKALGADDIIDT	0	1
Q50685	EIIRTGGRMAKVSTGYDLTQL	0	0
Q50685	GLLISFDRMNKVLEVDTANQV	0	0
Q50685	GPPQKPAYAAKPATPEEVAQL	0	0
Q50685	GTNAGGMRAVKYGVARHNVLG	0	0
Q50685	GYFLELEDPVKISLMRRIKQS	0	0
Q50685	HDEELTGPPQKPAYAAKPATP	0	0
Q50685	HMAIACKDPEKKKKLMTDIFA	0	0

Q50685	IACKDPEKKKKLMTDIFALAM	0	0
Q50685	ISGEHGVGRAKTGYFLELEDP	0	1
Q50685	MAIACKDPEKKKKLMTDIFAL	0	0
Q50685	PVKISLMRRIKQSFDPAGILN	0	0
Q50685	SARKLIEAREKAFWAAKALGA	0	0
Q50685	TLALVTEVIVKLHPRLDHNAS	0	0
Q7D8W0	FVCPDKVSEDKRNVLIAYGAE	0	0
Q7D8W0	GGSSKDRIAVKMIEAAEASGQ	0	1
Q7D8W0	GPEIWADTEGKVTHFVAGIGT	0	0
Q7D8W0	GTITGAGRYLKEVSGGRVRIV	0	0
Q7D8W0	GYKCVFVCPDKVSEDKRNVLI	0	0
Q7D8W0	IEAAEASGQLKPGGTIVEPTS	0	0
Q7D8W0	LALVAQRRGYKCVFVCPDKVS	0	0
Q7D8W0	LLSAVFEGRAKLADAVSAHMS	0	0
Q7D8W0	LPDGGRGYMSKIFNDAWMSSY	0	0
Q7D8W0	QSTVGDVLRRKSGALPALVHT	0	1
Q7D8W0	RLVRDIDGAWKPDQYANPEGP	0	0
Q7D8W0	SCGMAVVAALKVAEEAGPDAL	0	0
Q7D8W0	VEYLNPGGSSKDRIAVKMIEA	0	0
Q7D8W0	VPDGAGTVAAKVEYLNPGGSS	0	0
Q7D8W0	WDALMVVEEGKPVGVITRYDL	0	0

Table S6. The prediction results of our method for the test dataset.

Protein Name	Peptide	Class label	Prediction results
EG10032	AAVKSGSELGKQAKDIMDAGK	1	1
EG10032	HAPSGRVYHVKFNPPKVEGKD	1	1
EG10032	KSGSELGKQAKDIMDAGKLVT	1	1
EG10032	RVYHVKFNPPKVEGKDDVTGE	1	1
EG10241	DVVDAEFEEVKDKK_______	1	1
EG10241	VDAEFEEVKDKK_________	1	1
EG10420	ADAIFIEELRKADLYDKVSQA	1	1
EG10518	YRGPGLEEGMKIFQELKQTFG	1	1
EG10599	KATLEDLGQAKRVVINKDTTT	1	1
EG10599	SVAREIELEDKFENMGAQMVK	1	1
EG10599	VGKLIAEAMDKVGKEGVITVE	1	1
EG10758	IQGGGFEPGMKQKATKEPIKN	1	1
EG10789	TPSETAQLNLKKVLGFITDAG	1	1
EG10885	NKFPAIIYGGKEAPLAIELDH	1	1
EG10906	ALETLAQRSGKSELEAFEVAL	1	1
EG10906	ANELSDAAENKGTAVKKREDV	1	1
EG11005	NGCTVRNVYIKEAFDTGVRYD	1	1
EG11073	NLQNYLETELKRDNPVLIMGD	1	1
EG11137	VRDALEAAGLKADSAEVSMIP	1	1
EG11835	DDDEEEEEDEKQEDMMRLLRG	1	1
EG12316	FLAAIAKGEAKSPLLTPEKAI	1	1
EG13261	DLTAADGQTRKTFQEILAALG	1	1

EG13261	NRSEDAEAVLKTIPLQDQDTR	1	1
EG13433	CDMILVCNNRKGAVSVLDNLS	1	1
EG13723	GYGTLADVPEKVDMVDVFRNS	1	1
EG14074	AELEPQRNTIKTQNYQTRDPQ	1	1
EG20151	HSTKAFDASKKLTPEQAEQIK	1	1
EG20151	SRAAVAFAPGKPLEIVEIDVA	1	1
EG50010	STEEGKARVAKSAAGNYVFNE	1	1
EG10032	DVTGEELTTRKDDQEETVRKR	0	1
EG10032	EAEAGNTKYAKVDGTKPVAEV	0	0
EG10032	IILLGAPGAGKGTQAQFIMEK	0	0
EG10032	KGTQAQFIMEK__________	0	0
EG10032	KQAKDIMDAGKLVTDELVIAL	0	0
EG10032	MTAPLIGYYSKEAEAGNTKYA	0	1
EG10032	NTKYAKVDGTKPVAEVRADLE	0	0
EG10032	PRTIPQADAMKEAGINVDYVL	0	0
EG10032	PVAEVRADLEKILG_______	0	0
EG10032	RKDDQEETVRKRLVEYHQMTA	0	0
EG10032	STGDMLRAAVKSGSELGKQAK	0	0
EG10032	VKFNPPKVEGKDDVTGEELTT	0	0
EG10032	VTDELVIALVKERIAQEDCRN	0	0
EG10032	YSKEAEAGNTKYAKVDGTKPV	0	0
EG10241	ADNGDAWVEVKGQKMAPPQIS	0	0
EG10241	AGADASANNAKDDDVVDAEFE	0	0
EG10241	AKDKNSGKEQKITIKASSGLN	0	0
EG10241	ALETALKGEDKAAIEAKMQEL	0	0
EG10241	AMQRLKEAAEKAKIELSSAQQ	0	0
EG10241	APPQISAEVLKKMKKTAEDYL	0	0
EG10241	DADGILHVSAKDKNSGKEQKI	0	0
EG10241	DAEANAEADRKFEELVQTRNQ	0	0
EG10241	DAEFEEVKDKK__________	0	0
EG10241	DAGRIAGLEVKRIINEPTAAA	0	0
EG10241	DATGPKHMNIKVTRAKLESLV	0	0
EG10241	DGETLVGQPAKRQAVTNPQNT	0	0
EG10241	DGILHVSAKDKNSGKEQKITI	0	0
EG10241	DLVNRSIEPLKVALQDAGLSV	0	0
EG10241	EAGDKLPADDKTAIESALTAL	0	1
EG10241	GDAWVEVKGQKMAPPQISAEV	0	0
EG10241	GGQTRMPMVQKKVAEFFGKEP	0	0
EG10241	GQTRMPMVQKKVAEFFGKEP_	0	0
EG10241	HVSAKDKNSGKEQKITIKASS	0	0
EG10241	IIEIDEVDGEKTFEVLATNGD	0	0
EG10241	ISAEVLKKMKKTAEDYLGEPV	0	0
EG10241	KGEDKAAIEAKMQELAQVSQK	0	0
EG10241	KHMNIKVTRAKLESLVEDLVN	0	0
EG10241	KMQELAQVSQKLMEIAQQQHA	0	0
EG10241	LIAKNTTIPTKHSQVFSTAED	0	0
EG10241	LINYLVEEFKKDQGIDLRNDP	0	0
EG10241	MGGVMTTLIAKNTTIPTKHSQ	0	0

EG10241	________MGKIIGIDLGTTN	0	0
EG10241	NSGKEQKITIKASSGLNEDEI	0	0
EG10241	PPQISAEVLKKMKKTAEDYLG	0	0
EG10241	PYITADATGPKHMNIKVTRAK	0	0
EG10241	QGDHLLHSTRKQVEEAGDKLP	0	0
EG10241	QGERKRAADNKSLGQFNLDGI	0	0
EG10241	QISAEVLKKMKKTAEDYLGEP	0	0
EG10241	QRLKEAAEKAKIELSSAQQTD	0	1
EG10241	RLINYLVEEFKKDQGIDLRND	0	1
EG10241	RNDPLAMQRLKEAAEKAKIEL	0	1
EG10241	SALTALETALKGEDKAAIEAK	0	1
EG10241	SSGLNEDEIQKMVRDAEANAE	0	0
EG10241	TAAALAYGLDKGTGNRTIAVY	0	0
EG10241	TNPQNTLFAIKRLIGRRFQDE	0	0
EG10241	TRKQVEEAGDKLPADDKTAIE	0	0
EG10241	VAEFFGKEPRKDVNPDEAVAI	0	0
EG10241	VQGGVLTGDVKDVLLLDVTPL	0	0
EG10241	VQKKVAEFFGKEPRKDVNPDE	0	1
EG10241	VQRDVSIMPFKIIAADNGDAW	0	0
EG10241	VTIHVLQGERKRAADNKSLGQ	0	1
EG10241	YFNDAQRQATKDAGRIAGLEV	0	0
EG10420	AGENDPEAKRKIIGRVFVEVF	0	0
EG10420	ALAGENDPEAKRKIIGRVFVE	0	0
EG10420	ARIREQVGDDKVILGLSGGVD	0	0
EG10420	ASATGKAHVIKSHHNVGGLPK	0	0
EG10420	CPFAIMANEEKRFYGVQFHPE	0	0
EG10420	CQCEALWTPAKIIDDAVARIR	0	0
EG10420	DEEALKLEDVKWLAQGTIYPD	0	0
EG10420	EELRKADLYDKVSQAFTVFLP	0	0
EG10420	EMKMGLVEPLKELFKDEVRKI	0	0
EG10420	FVEVFDEEALKLEDVKWLAQG	0	1
EG10420	GIEDALTADGKPLLDVWMSHG	0	0
EG10420	GLGVRVLGEVKKEYCDLLRRA	0	0
EG10420	GLVEPLKELFKDEVRKIGLEL	0	0
EG10420	HNVGGLPKEMKMGLVEPLKEL	0	0
EG10420	ISRVVYDISGKPPATIEWE__	0	0
EG10420	KSHHNVGGLPKEMKMGLVEPL	0	0
EG10420	LGVRVLGEVKKEYCDLLRRAD	0	0
EG10420	LKELFKDEVRKIGLELGLPYD	0	0
EG10420	LLDVWMSHGDKVTAIPSDFIT	0	0
EG10420	____MTENIHKHRILILDFGS	0	0
EG10420	RSVGVMGDGRKYDWVVSLRAV	0	0
EG10420	TAMLLHRAIGKNLTCVFVDNG	0	0
EG10420	VIESAASATGKAHVIKSHHNV	0	0
EG10518	AKLEPFLKQMKAIDDLVKGFE	0	0
EG10518	AKTGAVINVKKPQFVSPGQMG	0	0
EG10518	CDGPSALPLAKLEPFLKQMKA	0	0
EG10518	EEGMKIFQELKQTFGVKIITD	0	0

EG10518	FQELKQTFGVKIITDVHEPSQ	0	0
EG10518	GQMGNIVDKFKEGGNEKVILC	0	0
EG10518	ICEHYVTVTQKLGIPYVFKAS	0	0
EG10518	IEAHPDPEHAKCDGPSALPLA	0	1
EG10518	IPYVFKASFDKANRSSIHSYR	0	0
EG10518	KQMKAIDDLVKGFEELDTSK_	0	1
EG10518	LPLAKLEPFLKQMKAIDDLVK	0	0
EG10518	MAKTGAVINVKKPQFVSPGQM	0	0
EG10518	__________MKQKVVSIGDIN	0	0
EG10518	________MKQKVVSIGDINVA	0	0
EG10518	RQTDLVEAMAKTGAVINVKKP	0	0
EG10518	SPGQMGNIVDKFKEGGNEKVI	0	1
EG10518	TQKLGIPYVFKASFDKANRSS	0	0
EG10518	VDKFKEGGNEKVILCDRGANF	0	0
EG10518	_VDMLGFSIMKKVSGNSPVIF	0	0
EG10518	VKGFEELDTSK___________	0	0
EG10518	VVDMLGFSIMKKVSGNSPVIF	0	0
EG10599	AATEVEMKEKKARVEDALHAT	0	0
EG10599	ADAVKVTLGPKGRNVVLDKSF	0	0
EG10599	AKLAGGVAVIKVGAATEVEMK	0	0
EG10599	DKSFGAPTITKDGVSVAREIE	0	0
EG10599	DREKLQERVAKLAGGVAVIKV	0	0
EG10599	DVKFGNDARVKMLRGVNVLAD	0	0
EG10599	EEATSDYDREKLQERVAKLAG	0	0
EG10599	EEPSVVANTVKGGDGNYGYNA	0	0
EG10599	EMLPVLEAVAKAGKPLLIIAE	0	0
EG10599	ESPFILLADKKISNIREMLPV	0	0
EG10599	GAATEVEMKEKKARVEDALHA	0	0
EG10599	GAQMVKEVASKANDAAGDGTT	0	1
EG10599	GGVALIRVASKLADLRGQNED	0	1
EG10599	GPKGRNVVLDKSFGAPTITKD	0	0
EG10599	GQNEDQNVGIKVALRAMEAPL	0	1
EG10599	ISANSDETVGKLIAEAMDKVG	0	1
EG10599	ISEEIGMELEKATLEDLGQAK	0	1
EG10599	KALSVPCSDSKAIAQVGTISA	0	0
EG10599	KAVTAAVEELKALSVPCSDSK	0	0
EG10599	KFENMGAQMVKEVASKANDAA	0	0
EG10599	KVGAATEVEMKEKKARVEDAL	0	0
EG10599	LAQAIITEGLKAVAAGMNPMD	0	0
EG10599	LESPFILLADKKISNIREMLP	0	0
EG10599	LGQAKRVVINKDTTTIIDGVG	0	0
EG10599	LIAEAMDKVGKEGVITVEDGT	0	0
EG10599	LVVNTMRGIVKVAAVKAPGFG	0	0
EG10599	________MAAKDVKFGNDARV	0	0
EG10599	____MAAKDVKFGNDARVKML	0	0
EG10599	MIDMGILDPTKVTRSALQYAA	0	0
EG10599	MRGIVKVAAVKAPGFGDRRKA	0	1
EG10599	NPMDLKRGIDKAVTAAVEELK	0	0

EG10599	PVLEAVAKAGKPLLIIAEDVE	0	0
EG10599	RGVNVLADAVKVTLGPKGRNV	0	0
EG10599	RGYLSPYFINKPETGAVELES	0	1
EG10599	TTECMVTDLPKNDAADLGAAG	0	0
EG10599	VAAGMNPMDLKRGIDKAVTAA	0	1
EG10599	VKAPGFGDRRKAMLQDIATLT	0	1
EG10758	EVVDGMDVVDKIKGVATGRSG	0	0
EG10758	FEPGMKQKATKEPIKNEANNG	0	0
EG10758	FHTNHGDIVIKTFDDKAPETV	0	0
EG10758	GDIVIKTFDDKAPETVKNFLD	0	0
EG10758	GGGFEPGMKQKATKEPIKNEA	0	1
EG10758	GRSGMHQDVPKEDVIIESVTV	0	0
EG10758	MKQKATKEPIKNEANNGLKNT	0	0
EG10758	PIKNEANNGLKNTRGTLAMAR	0	0
EG10758	TFDDKAPETVKNFLDYCREGF	0	0
EG10758	VDGMDVVDKIKGVATGRSGMH	0	0
EG10789	AAATIARHLAKEVDFFSIGTN	0	0
EG10789	AKASAQLETIKTKAGETFGEE	0	0
EG10789	ALEELDDEYLKERAADVRDIG	0	0
EG10789	ASAQLETIKTKAGETFGEEKE	0	0
EG10789	ASEKAELAKLKDLPAITLDGH	0	0
EG10789	DKELPYMNFPKEENPFLGWRA	0	0
EG10789	ELEQEIIALIKDKHMTADAAA	0	0
EG10789	EQEIIALIKDKHMTADAAAHE	0	0
EG10789	ERAADVRDIGKRLLRNILGLK	0	0
EG10789	EVERFLSGRAKASAQLETIKT	0	0
EG10789	GIAFGKALLLKEDEIVIDRKK	0	0
EG10789	IIRNTNFEDAKVLAEQALAQP	0	0
EG10789	ILASPGIAFGKALLLKEDEIV	0	0
EG10789	ISVEEVRALRKEIEIYKQELR	0	0
EG10789	IVRTMDIGGDKELPYMNFPKE	0	0
EG10789	IYKQELRDEGKAFDESIEIGV	0	1
EG10789	_KEDEIVIDRKKISADQVDQE	0	1
EG10789	KRLLRNILGLKIIDLSAIQDE	0	1
EG10789	LKEDEIVIDRKKISADQVDQE	0	1
EG10789	MSAISIPRIKKIIRNTNFEDA	0	0
EG10789	MTLVNKFIEEKTIC_______	0	0
EG10789	PMSPSVLNLIKQVIDASHAEG	0	0
EG10789	PSETAQLNLKKVLGFITDAGG	0	1
EG10789	PTEEEQFAAYKAVAEACGSQA	0	0
EG10789	QVASEKAELAKLKDLPAITLD	0	0
EG10789	QVIDASHAEGKWTGMCGELAG	0	1
EG10789	RAILRASAFGKLRIMFPMIIS	0	0
EG10789	RALRKEIEIYKQELRDEGKAF	0	1
EG10789	RAVQEQVASEKAELAKLKDLP	0	0
EG10789	SMSAISIPRIKKIIRNTNFED	0	0
EG10789	TKAGETFGEEKEAIFEGHIML	0	0
EG10789	TTDELMTLVNKFIEEKTIC__	0	0

EG10789	VGTGSVTSQVKNDDYLILDAV	0	0
EG10789	YVNPTNEVIDKMRAVQEQVAS	0	0
EG10885	APLAIELDHDKVMNMQAKAEF	0	0
EG10885	DHDKVMNMQAKAEFYSEVLTI	0	0
EG10885	GASRRLRAANKFPAIIYGGKE	0	0
EG10885	INAEVRKEQGKGASRRLRAAN	0	0
EG10885	IVVDGKEIKVKAQDVQRHPYK	0	0
EG10885	KAQDVQRHPYKPKLQHIDFVR	0	0
EG10885	LTIVVDGKEIKVKAQDVQRHP	0	0
EG10885	_MFTINAEVRKEQGKGASRRL	0	1
EG10885	QDVQRHPYKPKLQHIDFVRA_	0	0
EG10885	SEVLTIVVDGKEIKVKAQDVQ	0	0
EG10906	AAENKGTAVKKREDVHRMAEA	0	0
EG10906	AMRWIVEAARKRGDKSMALRL	0	0
EG10906	DAAENKGTAVKKREDVHRMAE	0	0
EG10906	DPKFGSELLAKFVNILMVDGK	0	0
EG10906	EDVHRMAEANKAFAHYRWLSL	0	0
EG10906	FVNILMVDGKKSTAESIVYSA	0	0
EG10906	IGQRKILPDPKFGSELLAKFV	0	1
EG10906	IVEAARKRGDKSMALRLANEL	0	1
EG10906	KFVNILMVDGKKSTAESIVYS	0	0
EG10906	LENVRPTVEVKSRRVGGSTYQ	0	0
EG10906	MPRRRVIGQRKILPDPKFGSE	0	0
EG10906	SFSHQAGASSKQPALGYLN__	0	0
EG11005	AEQIYIPVLIKKREQEKGLDR	0	0
EG11005	AFDTGVRYDFKGNFDLEGLER	0	0
EG11005	ANLKAMYSIAKKYDIPVVMDS	0	0
EG11005	ARFAENAYFIKQREAEYKDWT	0	1
EG11005	DFIIEAFKHVKENAANIKGLT	0	0
EG11005	EPKVLRHFTAKLKEV______	0	0
EG11005	_EQIYIPVLIKKREQEKGLDR	0	0
EG11005	FLLGRDPKTGKQLPCPAELLR	0	0
EG11005	GGHAAFVDAGKLLPHIPADQF	0	0
EG11005	GGQPVSLANLKAMYSIAKKYD	0	0
EG11005	IGSFLLGRDPKTGKQLPCPAE	0	0
EG11005	IKGLTFTYEPKVLRHFTAKLK	0	0
EG11005	KHVKENAANIKGLTFTYEPKV	0	1
EG11005	KVLRHFTAKLKEV________	0	1
EG11005	KYADMLAMSAKKDAMVPMGGL	0	1
EG11005	______MENFKHLPEPFRIRV	0	0
EG11005	MVPMGGLLCMKDDSFFDVYTE	0	0
EG11005	NLKAMYSIAKKYDIPVVMDSA	0	0
EG11005	PAQALACELYKVAGIRAVEIG	0	0
EG11005	PFRIRVIEPVKRTTRAYREEA	0	1
EG11005	PVLIKKREQEKGLDRSKMVAF	0	1
EG11005	REQEKGLDRSKMVAFSNYFFD	0	1
EG11005	RSYYALAESVKNIFGYQYTIP	0	0
EG11005	THMDFIIEAFKHVKENAANIK	0	0

EG11005	TIEQITRETYKYADMLAMSAK	0	0
EG11005	TRAYREEAIIKSGMNPFLLDS	0	0
EG11005	YADMLAMSAKKDAMVPMGGLL	0	0
EG11005	YFIKQREAEYKDWTIEQITRE	0	0
EG11073	DDMFPLEEVAKLGYNVFYHGQ	0	0
EG11073	DRFSWFDYRSKGFDDNRGLRI	0	0
EG11073	ENRKRWLRTGKCSFLPEEREW	0	0
EG11073	GIDYEIRSMEKPSDHAPVWAT	0	0
EG11073	KGHYGVALLTKETPIAVRRGF	0	0
EG11073	LDIGIGEENRKRWLRTGKCSF	0	0
EG11073	LGYNVFYHGQKGHYGVALLTK	0	1
EG11073	__________MKFVSFNINGLR	0	0
EG11073	PQGESRDHPIKFPAKAQFYQN	0	0
EG11073	QPDVIGLQETKVHDDMFPLEE	0	0
EG11073	RPHQLEAIVEKHQPDVIGLQE	0	1
EG11073	SRDHPIKFPAKAQFYQNLQNY	0	0
EG11137	ANPRLRAAVDKALSNNMTRDT	0	0
EG11137	DGSVAYLFSKKGVISFEKGDE	0	0
EG11137	DVYTAWEEMGKVRDALEAAGL	0	0
EG11137	FSKKGVISFEKGDEDTIMEAA	0	1
EG11137	HSKWANTRHRKAAQDAKRGKI	0	1
EG11137	KADMDAETAPKLMRLIDMLED	0	0
EG11137	KIIRELVTAAKLGGGDPDANP	0	1
EG11137	______MAGHSKWANTRHRKAA	0	0
EG11137	QDAKRGKIFTKIIRELVTAAK	0	1
EG11137	RKAAQDAKRGKIFTKIIRELV	0	1
EG11137	SAEVSMIPSTKADMDAETAPK	0	1
EG11137	TDGSVAYLFSKKGVISFEKGD	0	0
EG11137	TRHRKAAQDAKRGKIFTKIIR	0	0
EG11137	TVAEVRHAFSKCGGNLGTDGS	0	0
EG11835	AAGGNTTSGSKGQNAPKDPRI	0	0
EG11835	DQEARDRKRQKKRRGHAPGSR	0	0
EG11835	EELDQEARDRKRQKKRRGHAP	0	0
EG11835	GVTEKVTKQHKPKSEKPMLSP	0	0
EG11835	IPLGVTEKVTKQHKPKSEKPM	0	0
EG11835	KLDRIDELMQKLGLSYDDDEE	0	0
EG11835	KTPIPLGVTEKVTKQHKPKSE	0	0
EG11835	__________MKPSSSNSRSKG	0	0
EG11835	MKPSSSNSRSKGHAKARRKTR	0	0
EG11835	NAPKDPRIGSKTPIPLGVTEK	0	0
EG11835	QEARDRKRQKKRRGHAPGSRA	0	0
EG11835	RSKGHAKARRKTREELDQEAR	0	0
EG11835	SAEEQSWVDAKLDRIDELMQK	0	0
EG11835	SSNSRSKGHAKARRKTREELD	0	0
EG11835	TEKVTKQHKPKSEKPMLSPQA	0	1
EG11835	TSGSKGQNAPKDPRIGSKTPI	0	0
EG11835	VTKQHKPKSEKPMLSPQAELE	0	0
EG12316	AERAAEGIAPKPLDANQMAAL	0	0

EG12316	AERSAAGCTIKLNKEPIIEYL	0	1
EG12316	AIKQGLLTVEKKGKKNIFSGR	0	1
EG12316	ARPLSAVQGEKIDEVFIGSCM	0	0
EG12316	AVIDIDLADIKEPILCAPNDP	0	0
EG12316	DDAKLAPIAAKALSHTLLMFD	0	0
EG12316	DIPLHALAMLKNAREGIEPDQ	0	1
EG12316	DMPESVLVRFKGKMQPGITLR	0	0
EG12316	DNFYDVEEKAKAGNEYAKQVM	0	1
EG12316	EAKSPLLTPEKAIELLGTMQG	0	1
EG12316	EKAKAGNEYAKQVMQSWADAE	0	0
EG12316	ELAAVAALIGKLPTPEEYQTY	0	0
EG12316	ESDRGFSLAQKMVGRACGVKG	0	0
EG12316	EYLNSNIVLLKWMIAEGYGDR	0	0
EG12316	EYQTYVAQVDKTAVDTYRYLN	0	0
EG12316	GDVVGTGSSRKSATNSVLWFM	0	0
EG12316	GIRPGAYCEPKMTSVGSQDTT	0	0
EG12316	GLLTVEKKGKKNIFSGRILEI	0	0
EG12316	HPLIDALDDAKLAPIAAKALS	0	0
EG12316	IKQGLLTVEKKGKKNIFSGRI	0	0
EG12316	ILEIEGLPDLKVEQAFELTDA	0	0
EG12316	KRGGGLCLGGKIAPIFFNTME	0	0
EG12316	LAEKLTVTVFKVTGETNTDDL	0	0
EG12316	LPHSDVFRQAKDVAESDRGFS	0	0
EG12316	LVHAIPLYAIKQGLLTVEKKG	0	0
EG12316	MFDNFYDVEEKAKAGNEYAKQ	0	0
EG12316	MGDDIPHVPNKRGGGLCLGGK	0	0
EG12316	MGDVIDVYPYKGEVRNHETGE	0	0
EG12316	____MLEEYRKHVAERAAEGI	0	1
EG12316	NFNQLSQYTEKADGVIFQTAV	0	0
EG12316	NQMAALVELLKNPPAGEEEFL	0	1
EG12316	PDQPGVVGPIKQIEALQQKGF	0	1
EG12316	PESVLVRFKGKMQPGITLRDL	0	0
EG12316	PIKQIEALQQKGFPLAYVGDV	0	0
EG12316	PLIIGRGLTTKAREALGLPHS	0	0
EG12316	PPGVDEAAYVKAGFLAAIAKG	0	0
EG12316	QGLLTVEKKGKKNIFSGRILE	0	0
EG12316	QKMVGRACGVKGIRPGAYCEP	0	0
EG12316	QSFCHTAAYPKPVDVNTHHTL	0	0
EG12316	RAAGKLLDAHKGQLPTRLWVA	0	0
EG12316	SAAGCTIKLNKEPIIEYLNSN	0	0
EG12316	TEEGYYSVFGKSGARIEIPGC	0	1
EG12316	TGELLATFELKTDVLIDEVRA	0	0
EG12316	TLERRIQGMEKWLANPELLEA	0	0
EG12316	TNIGHFRAAGKLLDAHKGQLP	0	1
EG12316	TTGPMTRDELKDLACLGFSAD	0	0
EG12316	VKAGFLAAIAKGEAKSPLLTP	0	0
EG12316	WFLNRPALAEKLTVTVFKVTG	0	0
EG13261	ALELLFGHLRKDLTAADGQTR	0	0

EG13261	ALGTGDALASKYRRQLYALLY	0	0
EG13261	AQYNGQFILAKLDCDAEQMIA	0	0
EG13261	DKVLPREEELKAQQAMQLMQE	0	0
EG13261	PEEAIRALLDKVLPREEELKA	0	0
EG13261	QGLVAQIELLKQAADTPEIQQ	0	0
EG13261	SNYTDALPLLKDAWQLSNQNG	0	0
EG13433	AALSGMEEGGKLAQEAGWLMA	0	0
EG13433	DPRPQAEIRAKDMSVFSSLIR	0	1
EG13433	FIDGMHEAGMKTTGKHFPGHG	0	0
EG13433	GERSYHADPQKALAIASRFID	0	1
EG13433	GHGAVTADSHKETPCDPRPQA	0	0
EG13433	KAERVTRLYHKGSFSRQELMD	0	0
EG13433	MHEAGMKTTGKHFPGHGAVTA	0	0
EG13433	NQLHERWQEEKAGH_______	0	0
EG13433	RPASGSPYWLKTVLRQELGFD	0	0
EG13433	RQELMDSARWKAISTRLNQLH	0	0
EG13433	SVFSSLIRENKLDAIMPAHVI	0	0
EG13433	VSVLDNLSPIKAERVTRLYHK	0	0
EG13723	AIEIPRLGLAK__________	0	0
EG13723	HTIALVGASDKPDRPSYRVMK	0	0
EG13723	KPDRPSYRVMKYLLDQGYHVI	0	1
EG13723	KVAGKTLLGQKGYGTLADVPE	0	1
EG13723	_________MKETDIAGILTS	0	1
EG13723	QGYHVIPVSPKVAGKTLLGQK	0	0
EG13723	VAQEAIAIGAKTLWMQLGVIN	0	0
EG13723	VIPVSPKVAGKTLLGQKGYGT	0	0
EG14074	ARIEKMGVTIKCNNEVGNTLT	0	0
EG14074	AVCARVCPTEKLCQSGCTRAG	0	0
EG14074	DVAMDVASTLKVLGCQAVTCV	0	0
EG14074	EELDEFPASEKEFTSARELGV	0	0
EG14074	EGDKTVVYAVKTGKEAAEAIH	0	0
EG14074	FAAGDIVEGDKTVVYAVKTGK	0	0
EG14074	FIRSIYFRNFKGAAETIRENN	0	0
EG14074	GFTPVAVEGNKVTFKHVRLSG	0	1
EG14074	IYQPGTKTLGKVAIIGAGPAG	0	1
EG14074	KTVVYAVKTGKEAAEAIHHYL	0	1
EG14074	LSGELTMAADKIILAVGQHAR	0	0
EG14074	NQGYDVTIYEKEAHPGGWLRN	0	0
EG14074	QACPAQTDPGKFIRSIYFRNF	0	0
EG14074	TGMEIYQPGTKTLGKVAIIGA	0	0
EG14074	TPAFTSLLAIKEASRCLLCHD	0	0
EG14074	VAVEGNKVTFKHVRLSGELTM	0	1
EG14074	VGNTLTLEQLKAENRAVLVTV	0	0
EG14074	VLDAEIARIEKMGVTIKCNNE	0	0
EG20151	AAGNYVFNERKMLDASHVVVF	0	0
EG20151	AAILDAEFGLKEKGYTSLVVV	0	0
EG20151	ADGRFATPEAKAANDKGRKFF	0	0
EG20151	AKTAMDDVWLKLVVDQEDADG	0	0

EG20151	ATPEAKAANDKGRKFFADMHR	0	0
EG20151	DASHVVVFCAKTAMDDVWLKL	0	0
EG20151	DLHDDAEWMAKQVYLNVGNFL	0	0
EG20151	EAKAANDKGRKFFADMHRKDL	0	0
EG20151	GRKFFADMHRKDLHDDAEWMA	0	0
EG20151	HFIVASTEEGKARVAKSAAGN	0	0
EG20151	ILDAEFGLKEKGYTSLVVVPV	0	0
EG20151	ISVALKRHSTKAFDASKKLTP	0	0
EG20151	KLTPEQAEQIKTLLQYSPSST	0	0
EG20151	__MDIISVALKRHSTKAFDAS	0	0
EG20151	RHSTKAFDASKKLTPEQAEQI	0	0
EG20151	SVEDFNATLPKSRLPQNITLT	0	0
EG50010	APPKKGEVLIKVTHTGVCHTD	0	0
EG50010	CGECEFCRSGKTNLCVAVRET	0	0
EG50010	EIVEIDVAPPKKGEVLIKVTH	0	1
EG50010	GRIIAIDTNPKKFDLARRFGA	0	0
EG50010	INPNDYDKPIKDVLLDINKWG	0	1
EG50010	IVEIDVAPPKKGEVLIKVTHT	0	0
EG50010	LAVVQGARQAKAGRIIAIDTN	0	1
EG50010	LCVAVRETQGKGLMPDGTTRF	0	0
EG50010	_________MKSRAAVAFAPG	0	0
EG50010	NDAFDLMHEGKSIRTVIRY__	0	0
EG50010	PFQLVTGRVWKGSAFGGVKGR	0	0
EG50010	PIKDVLLDINKWGIDHTFECI	0	0
EG50010	QLPGMVEDAMKGDIDLEPFVT	0	0
EG50010	RIIAIDTNPKKFDLARRFGAT	0	0
EG50010	TDCINPNDYDKPIKDVLLDIN	0	0
EG50010	TGIGAVHNTAKVQPGDSVAVF	0	0
EG50010	VEVGEGVTSVKPGDHVIPLYT	0	0
EG50010	VWKGSAFGGVKGRSQLPGMVE	0	0
EG50010	YTVVAEVSLAKINPEANHEHV	0	0

Table S7. The prediction results of PUL-PUP for the test dataset.

Protein Name	Peptide	Class label	Prediction results
EG10032	AAVKSGSELGKQAKDIMDAGK	1	1
EG10032	HAPSGRVYHVKFNPPKVEGKD	1	0
EG10032	KSGSELGKQAKDIMDAGKLVT	1	1
EG10032	RVYHVKFNPPKVEGKDDVTGE	1	1
EG10241	DVVDAEFEEVKDKK_______	1	1
EG10241	VDAEFEEVKDKK_________	1	0
EG10420	ADAIFIEELRKADLYDKVSQA	1	0
EG10518	YRGPGLEEGMKIFQELKQTFG	1	1
EG10599	KATLEDLGQAKRVVINKDTTT	1	0
EG10599	SVAREIELEDKFENMGAQMVK	1	0
EG10599	VGKLIAEAMDKVGKEGVITVE	1	1
EG10758	IQGGGFEPGMKQKATKEPIKN	1	1
EG10789	TPSETAQLNLKKVLGFITDAG	1	1

EG10885	NKFPAIIYGGKEAPLAIELDH	1	1
EG10906	ALETLAQRSGKSELEAFEVAL	1	1
EG10906	ANELSDAAENKGTAVKKREDV	1	1
EG11005	NGCTVRNVYIKEAFDTGVRYD	1	1
EG11073	NLQNYLETELKRDNPVLIMGD	1	0
EG11137	VRDALEAAGLKADSAEVSMIP	1	1
EG11835	DDDEEEEEDEKQEDMMRLLRG	1	1
EG12316	FLAAIAKGEAKSPLLTPEKAI	1	1
EG13261	DLTAADGQTRKTFQEILAALG	1	1
EG13261	NRSEDAEAVLKTIPLQDQDTR	1	1
EG13433	CDMILVCNNRKGAVSVLDNLS	1	0
EG13723	GYGTLADVPEKVDMVDVFRNS	1	0
EG14074	AELEPQRNTIKTQNYQTRDPQ	1	0
EG20151	HSTKAFDASKKLTPEQAEQIK	1	1
EG20151	SRAAVAFAPGKPLEIVEIDVA	1	1
EG50010	STEEGKARVAKSAAGNYVFNE	1	1
EG10032	DVTGEELTTRKDDQEETVRKR	0	1
EG10032	EAEAGNTKYAKVDGTKPVAEV	0	0
EG10032	IILLGAPGAGKGTQAQFIMEK	0	0
EG10032	KGTQAQFIMEK__________	0	0
EG10032	KQAKDIMDAGKLVTDELVIAL	0	0
EG10032	MTAPLIGYYSKEAEAGNTKYA	0	1
EG10032	NTKYAKVDGTKPVAEVRADLE	0	0
EG10032	PRTIPQADAMKEAGINVDYVL	0	0
EG10032	PVAEVRADLEKILG_______	0	0
EG10032	RKDDQEETVRKRLVEYHQMTA	0	0
EG10032	STGDMLRAAVKSGSELGKQAK	0	0
EG10032	VKFNPPKVEGKDDVTGEELTT	0	0
EG10032	VTDELVIALVKERIAQEDCRN	0	0
EG10032	YSKEAEAGNTKYAKVDGTKPV	0	0
EG10241	ADNGDAWVEVKGQKMAPPQIS	0	0
EG10241	AGADASANNAKDDDVVDAEFE	0	0
EG10241	AKDKNSGKEQKITIKASSGLN	0	0
EG10241	ALETALKGEDKAAIEAKMQEL	0	1
EG10241	AMQRLKEAAEKAKIELSSAQQ	0	0
EG10241	APPQISAEVLKKMKKTAEDYL	0	0
EG10241	DADGILHVSAKDKNSGKEQKI	0	0
EG10241	DAEANAEADRKFEELVQTRNQ	0	0
EG10241	DAEFEEVKDKK__________	0	0
EG10241	DAGRIAGLEVKRIINEPTAAA	0	0
EG10241	DATGPKHMNIKVTRAKLESLV	0	0
EG10241	DGETLVGQPAKRQAVTNPQNT	0	0
EG10241	DGILHVSAKDKNSGKEQKITI	0	0
EG10241	DLVNRSIEPLKVALQDAGLSV	0	0
EG10241	EAGDKLPADDKTAIESALTAL	0	1
EG10241	GDAWVEVKGQKMAPPQISAEV	0	0
EG10241	GGQTRMPMVQKKVAEFFGKEP	0	0
EG10241	GQTRMPMVQKKVAEFFGKEP_	0	0

EG10241	HVSAKDKNSGKEQKITIKASS	0	0
EG10241	IIEIDEVDGEKTFEVLATNGD	0	0
EG10241	ISAEVLKKMKKTAEDYLGEPV	0	0
EG10241	KGEDKAAIEAKMQELAQVSQK	0	0
EG10241	KHMNIKVTRAKLESLVEDLVN	0	0
EG10241	KMQELAQVSQKLMEIAQQQHA	0	0
EG10241	LIAKNTTIPTKHSQVFSTAED	0	0
EG10241	LINYLVEEFKKDQGIDLRNDP	0	0
EG10241	MGGVMTTLIAKNTTIPTKHSQ	0	0
EG10241	________MGKIIGIDLGTTN	0	0
EG10241	NSGKEQKITIKASSGLNEDEI	0	0
EG10241	PPQISAEVLKKMKKTAEDYLG	0	0
EG10241	PYITADATGPKHMNIKVTRAK	0	0
EG10241	QGDHLLHSTRKQVEEAGDKLP	0	0
EG10241	QGERKRAADNKSLGQFNLDGI	0	0
EG10241	QISAEVLKKMKKTAEDYLGEP	0	0
EG10241	QRLKEAAEKAKIELSSAQQTD	0	1
EG10241	RLINYLVEEFKKDQGIDLRND	0	1
EG10241	RNDPLAMQRLKEAAEKAKIEL	0	1
EG10241	SALTALETALKGEDKAAIEAK	0	1
EG10241	SSGLNEDEIQKMVRDAEANAE	0	0
EG10241	TAAALAYGLDKGTGNRTIAVY	0	0
EG10241	TNPQNTLFAIKRLIGRRFQDE	0	0
EG10241	TRKQVEEAGDKLPADDKTAIE	0	0
EG10241	VAEFFGKEPRKDVNPDEAVAI	0	0
EG10241	VQGGVLTGDVKDVLLLDVTPL	0	0
EG10241	VQKKVAEFFGKEPRKDVNPDE	0	1
EG10241	VQRDVSIMPFKIIAADNGDAW	0	0
EG10241	VTIHVLQGERKRAADNKSLGQ	0	1
EG10241	YFNDAQRQATKDAGRIAGLEV	0	1
EG10420	AGENDPEAKRKIIGRVFVEVF	0	0
EG10420	ALAGENDPEAKRKIIGRVFVE	0	0
EG10420	ARIREQVGDDKVILGLSGGVD	0	0
EG10420	ASATGKAHVIKSHHNVGGLPK	0	0
EG10420	CPFAIMANEEKRFYGVQFHPE	0	0
EG10420	CQCEALWTPAKIIDDAVARIR	0	0
EG10420	DEEALKLEDVKWLAQGTIYPD	0	0
EG10420	EELRKADLYDKVSQAFTVFLP	0	0
EG10420	EMKMGLVEPLKELFKDEVRKI	0	0
EG10420	FVEVFDEEALKLEDVKWLAQG	0	1
EG10420	GIEDALTADGKPLLDVWMSHG	0	1
EG10420	GLGVRVLGEVKKEYCDLLRRA	0	0
EG10420	GLVEPLKELFKDEVRKIGLEL	0	1
EG10420	HNVGGLPKEMKMGLVEPLKEL	0	0
EG10420	ISRVVYDISGKPPATIEWE__	0	0
EG10420	KSHHNVGGLPKEMKMGLVEPL	0	0
EG10420	LGVRVLGEVKKEYCDLLRRAD	0	0
EG10420	LKELFKDEVRKIGLELGLPYD	0	0

EG10420	LLDVWMSHGDKVTAIPSDFIT	0	0
EG10420	____MTENIHKHRILILDFGS	0	0
EG10420	RSVGVMGDGRKYDWVVSLRAV	0	0
EG10420	TAMLLHRAIGKNLTCVFVDNG	0	0
EG10420	VIESAASATGKAHVIKSHHNV	0	0
EG10518	AKLEPFLKQMKAIDDLVKGFE	0	1
EG10518	AKTGAVINVKKPQFVSPGQMG	0	1
EG10518	CDGPSALPLAKLEPFLKQMKA	0	1
EG10518	EEGMKIFQELKQTFGVKIITD	0	0
EG10518	FQELKQTFGVKIITDVHEPSQ	0	0
EG10518	GQMGNIVDKFKEGGNEKVILC	0	0
EG10518	ICEHYVTVTQKLGIPYVFKAS	0	0
EG10518	IEAHPDPEHAKCDGPSALPLA	0	1
EG10518	IPYVFKASFDKANRSSIHSYR	0	0
EG10518	KQMKAIDDLVKGFEELDTSK_	0	1
EG10518	LPLAKLEPFLKQMKAIDDLVK	0	1
EG10518	MAKTGAVINVKKPQFVSPGQM	0	0
EG10518	__________MKQKVVSIGDIN	0	0
EG10518	________MKQKVVSIGDINVA	0	0
EG10518	RQTDLVEAMAKTGAVINVKKP	0	0
EG10518	SPGQMGNIVDKFKEGGNEKVI	0	1
EG10518	TQKLGIPYVFKASFDKANRSS	0	0
EG10518	VDKFKEGGNEKVILCDRGANF	0	0
EG10518	_VDMLGFSIMKKVSGNSPVIF	0	0
EG10518	VKGFEELDTSK__________	0	0
EG10518	VVDMLGFSIMKKVSGNSPVIF	0	1
EG10599	AATEVEMKEKKARVEDALHAT	0	1
EG10599	ADAVKVTLGPKGRNVVLDKSF	0	1
EG10599	AKLAGGVAVIKVGAATEVEMK	0	1
EG10599	DKSFGAPTITKDGVSVAREIE	0	0
EG10599	DREKLQERVAKLAGGVAVIKV	0	0
EG10599	DVKFGNDARVKMLRGVNVLAD	0	0
EG10599	EEATSDYDREKLQERVAKLAG	0	0
EG10599	EEPSVVANTVKGGDGNYGYNA	0	0
EG10599	EMLPVLEAVAKAGKPLLIIAE	0	0
EG10599	ESPFILLADKKISNIREMLPV	0	0
EG10599	GAATEVEMKEKKARVEDALHA	0	0
EG10599	GAQMVKEVASKANDAAGDGTT	0	1
EG10599	GGVALIRVASKLADLRGQNED	0	1
EG10599	GPKGRNVVLDKSFGAPTITKD	0	0
EG10599	GQNEDQNVGIKVALRAMEAPL	0	1
EG10599	ISANSDETVGKLIAEAMDKVG	0	1
EG10599	ISEEIGMELEKATLEDLGQAK	0	1
EG10599	KALSVPCSDSKAIAQVGTISA	0	1
EG10599	KAVTAAVEELKALSVPCSDSK	0	1
EG10599	KFENMGAQMVKEVASKANDAA	0	0
EG10599	KVGAATEVEMKEKKARVEDAL	0	1
EG10599	LAQAIITEGLKAVAAGMNPMD	0	1

EG10599	LESPFILLADKKISNIREMLP	0	1
EG10599	LGQAKRVVINKDTTTIIDGVG	0	1
EG10599	LIAEAMDKVGKEGVITVEDGT	0	0
EG10599	LVVNTMRGIVKVAAVKAPGFG	0	0
EG10599	_______MAAKDVKFGNDARV	0	0
EG10599	____MAAKDVKFGNDARVKML	0	0
EG10599	MIDMGILDPTKVTRSALQYAA	0	0
EG10599	MRGIVKVAAVKAPGFGDRRKA	0	1
EG10599	NPMDLKRGIDKAVTAAVEELK	0	0
EG10599	PVLEAVAKAGKPLLIIAEDVE	0	0
EG10599	RGVNVLADAVKVTLGPKGRNV	0	0
EG10599	RGYLSPYFINKPETGAVELES	0	1
EG10599	TTECMVTDLPKNDAADLGAAG	0	1
EG10599	VAAGMNPMDLKRGIDKAVTAA	0	1
EG10599	VKAPGFGDRRKAMLQDIATLT	0	1
EG10758	EVVDGMDVVDKIKGVATGRSG	0	0
EG10758	FEPGMKQKATKEPIKNEANNG	0	0
EG10758	FHTNHGDIVIKTFDDKAPETV	0	0
EG10758	GDIVIKTFDDKAPETVKNFLD	0	0
EG10758	GGGFEPGMKQKATKEPIKNEA	0	1
EG10758	GRSGMHQDVPKEDVIIESVTV	0	0
EG10758	MKQKATKEPIKNEANNGLKNT	0	0
EG10758	PIKNEANNGLKNTRGTLAMAR	0	0
EG10758	TFDDKAPETVKNFLDYCREGF	0	0
EG10758	VDGMDVVDKIKGVATGRSGMH	0	0
EG10789	AAATIARHLAKEVDFFSIGTN	0	1
EG10789	AKASAQLETIKTKAGETFGEE	0	1
EG10789	ALEELDDEYLKERAADVRDIG	0	1
EG10789	ASAQLETIKTKAGETFGEEKE	0	0
EG10789	ASEKAELAKLKDLPAITLDGH	0	1
EG10789	DKELPYMNFPKEENPFLGWRA	0	0
EG10789	ELEQEIIALIKDKHMTADAAA	0	0
EG10789	EQEIIALIKDKHMTADAAAHE	0	0
EG10789	ERAADVRDIGKRLLRNILGLK	0	0
EG10789	EVERFLSGRAKASAQLETIKT	0	0
EG10789	GIAFGKALLLKEDEIVIDRKK	0	0
EG10789	IIRNTNFEDAKVLAEQALAQP	0	0
EG10789	ILASPGIAFGKALLLKEDEIV	0	0
EG10789	ISVEEVRALRKEIEIYKQELR	0	0
EG10789	IVRTMDIGGDKELPYMNFPKE	0	0
EG10789	IYKQELRDEGKAFDESIEIGV	0	1
EG10789	_KEDEIVIDRKKISADQVDQE	0	1
EG10789	KRLLRNILGLKIIDLSAIQDE	0	1
EG10789	LKEDEIVIDRKKISADQVDQE	0	1
EG10789	MSAISIPRIKKIIRNTNFEDA	0	0
EG10789	MTLVNKFIEEKTIC_______	0	1
EG10789	PMSPSVLNLIKQVIDASHAEG	0	0
EG10789	PSETAQLNLKKVLGFITDAGG	0	1

EG10789	PTEEEQFAAYKAVAEACGSQA	0	0
EG10789	QVASEKAELAKLKDLPAITLD	0	0
EG10789	QVIDASHAEGKWTGMCGELAG	0	1
EG10789	RAILRASAFGKLRIMFPMIIS	0	0
EG10789	RALRKEIEIYKQELRDEGKAF	0	1
EG10789	RAVQEQVASEKAELAKLKDLP	0	1
EG10789	SMSAISIPRIKKIIRNTNFED	0	1
EG10789	TKAGETFGEEKEAIFEGHIML	0	0
EG10789	TTDELMTLVNKFIEEKTIC__	0	0
EG10789	VGTGSVTSQVKNDDYLILDAV	0	0
EG10789	YVNPTNEVIDKMRAVQEQVAS	0	0
EG10885	APLAIELDHDKVMNMQAKAEF	0	0
EG10885	DHDKVMNMQAKAEFYSEVLTI	0	0
EG10885	GASRRLRAANKFPAIIYGGKE	0	1
EG10885	INAEVRKEQGKGASRRLRAAN	0	1
EG10885	IVVDGKEIKVKAQDVQRHPYK	0	1
EG10885	KAQDVQRHPYKPKLQHIDFVR	0	0
EG10885	LTIVVDGKEIKVKAQDVQRHP	0	1
EG10885	_MFTINAEVRKEQGKGASRRL	0	1
EG10885	QDVQRHPYKPKLQHIDFVRA_	0	0
EG10885	SEVLTIVVDGKEIKVKAQDVQ	0	1
EG10906	AAENKGTAVKKREDVHRMAEA	0	0
EG10906	AMRWIVEAARKRGDKSMALRL	0	0
EG10906	DAAENKGTAVKKREDVHRMAE	0	0
EG10906	DPKFGSELLAKFVNILMVDGK	0	0
EG10906	EDVHRMAEANKAFAHYRWLSL	0	0
EG10906	FVNILMVDGKKSTAESIVYSA	0	0
EG10906	IGQRKILPDPKFGSELLAKFV	0	1
EG10906	IVEAARKRGDKSMALRLANEL	0	1
EG10906	KFVNILMVDGKKSTAESIVYS	0	0
EG10906	LENVRPTVEVKSRRVGGSTYQ	0	0
EG10906	MPRRRVIGQRKILPDPKFGSE	0	0
EG10906	SFSHQAGASSKQPALGYLN__	0	0
EG11005	AEQIYIPVLIKKREQEKGLDR	0	0
EG11005	AFDTGVRYDFKGNFDLEGLER	0	0
EG11005	ANLKAMYSIAKKYDIPVVMDS	0	0
EG11005	ARFAENAYFIKQREAEYKDWT	0	1
EG11005	DFIIEAFKHVKENAANIKGLT	0	0
EG11005	EPKVLRHFTAKLKEV______	0	0
EG11005	_EQIYIPVLIKKREQEKGLDR	0	0
EG11005	FLLGRDPKTGKQLPCPAELLR	0	0
EG11005	GGHAAFVDAGKLLPHIPADQF	0	0
EG11005	GGQPVSLANLKAMYSIAKKYD	0	0
EG11005	IGSFLLGRDPKTGKQLPCPAE	0	0
EG11005	IKGLTFTYEPKVLRHFTAKLK	0	0
EG11005	KHVKENAANIKGLTFTYEPKV	0	1
EG11005	KVLRHFTAKLKEV________	0	1
EG11005	KYADMLAMSAKKDAMVPMGGL	0	1

EG11005	______MENFKHLPEPFRIRV	0	0
EG11005	MVPMGGLLCMKDDSFFDVYTE	0	0
EG11005	NLKAMYSIAKKYDIPVVMDSA	0	0
EG11005	PAQALACELYKVAGIRAVEIG	0	0
EG11005	PFRIRVIEPVKRTTRAYREEA	0	1
EG11005	PVLIKKREQEKGLDRSKMVAF	0	1
EG11005	REQEKGLDRSKMVAFSNYFFD	0	1
EG11005	RSYYALAESVKNIFGYQYTIP	0	0
EG11005	THMDFIIEAFKHVKENAANIK	0	1
EG11005	TIEQITRETYKYADMLAMSAK	0	1
EG11005	TRAYREEAIIKSGMNPFLLDS	0	0
EG11005	YADMLAMSAKKDAMVPMGGLL	0	0
EG11005	YFIKQREAEYKDWTIEQITRE	0	0
EG11073	DDMFPLEEVAKLGYNVFYHGQ	0	0
EG11073	DRFSWFDYRSKGFDDNRGLRI	0	0
EG11073	ENRKRWLRTGKCSFLPEEREW	0	0
EG11073	GIDYEIRSMEKPSDHAPVWAT	0	0
EG11073	KGHYGVALLTKETPIAVRRGF	0	0
EG11073	LDIGIGEENRKRWLRTGKCSF	0	0
EG11073	LGYNVFYHGQKGHYGVALLTK	0	1
EG11073	_________MKFVSFNINGLR	0	0
EG11073	PQGESRDHPIKFPAKAQFYQN	0	0
EG11073	QPDVIGLQETKVHDDMFPLEE	0	0
EG11073	RPHQLEAIVEKHQPDVIGLQE	0	1
EG11073	SRDHPIKFPAKAQFYQNLQNY	0	0
EG11137	ANPRLRAAVDKALSNNMTRDT	0	0
EG11137	DGSVAYLFSKKGVISFEKGDE	0	0
EG11137	DVYTAWEEMGKVRDALEAAGL	0	0
EG11137	FSKKGVISFEKGDEDTIMEAA	0	1
EG11137	HSKWANTRHRKAAQDAKRGKI	0	1
EG11137	KADMDAETAPKLMRLIDMLED	0	0
EG11137	KIIRELVTAAKLGGGDPDANP	0	1
EG11137	_____MAGHSKWANTRHRKAA	0	0
EG11137	QDAKRGKIFTKIIRELVTAAK	0	1
EG11137	RKAAQDAKRGKIFTKIIRELV	0	1
EG11137	SAEVSMIPSTKADMDAETAPK	0	1
EG11137	TDGSVAYLFSKKGVISFEKGD	0	0
EG11137	TRHRKAAQDAKRGKIFTKIIR	0	0
EG11137	TVAEVRHAFSKCGGNLGTDGS	0	0
EG11835	AAGGNTTSGSKGQNAPKDPRI	0	0
EG11835	DQEARDRKRQKKRRGHAPGSR	0	0
EG11835	EELDQEARDRKRQKKRRGHAP	0	0
EG11835	GVTEKVTKQHKPKSEKPMLSP	0	1
EG11835	IPLGVTEKVTKQHKPKSEKPM	0	1
EG11835	KLDRIDELMQKLGLSYDDDEE	0	0
EG11835	KTPIPLGVTEKVTKQHKPKSE	0	1
EG11835	_________MKPSSSNSRSKG	0	1
EG11835	MKPSSSNSRSKGHAKARRKTR	0	0

EG11835	NAPKDPRIGSKTPIPLGVTEK	0	0
EG11835	QEARDRKRQKKRRGHAPGSRA	0	0
EG11835	RSKGHAKARRKTREELDQEAR	0	0
EG11835	SAEEQSWVDAKLDRIDELMQK	0	0
EG11835	SSNSRSKGHAKARRKTREELD	0	0
EG11835	TEKVTKQHKPKSEKPMLSPQA	0	1
EG11835	TSGSKGQNAPKDPRIGSKTPI	0	0
EG11835	VTKQHKPKSEKPMLSPQAELE	0	0
EG12316	AERAAEGIAPKPLDANQMAAL	0	0
EG12316	AERSAAGCTIKLNKEPIIEYL	0	1
EG12316	AIKQGLLTVEKKGKKNIFSGR	0	1
EG12316	ARPLSAVQGEKIDEVFIGSCM	0	0
EG12316	AVIDIDLADIKEPILCAPNDP	0	0
EG12316	DDAKLAPIAAKALSHTLLMFD	0	0
EG12316	DIPLHALAMLKNAREGIEPDQ	0	1
EG12316	DMPESVLVRFKGKMQPGITLR	0	0
EG12316	DNFYDVEEKAKAGNEYAKQVM	0	1
EG12316	EAKSPLLTPEKAIELLGTMQG	0	1
EG12316	EKAKAGNEYAKQVMQSWADAE	0	0
EG12316	ELAAVAALIGKLPTPEEYQTY	0	0
EG12316	ESDRGFSLAQKMVGRACGVKG	0	0
EG12316	EYLNSNIVLLKWMIAEGYGDR	0	0
EG12316	EYQTYVAQVDKTAVDTYRYLN	0	1
EG12316	GDVVGTGSSRKSATNSVLWFM	0	0
EG12316	GIRPGAYCEPKMTSVGSQDTT	0	0
EG12316	GLLTVEKKGKKNIFSGRILEI	0	0
EG12316	HPLIDALDDAKLAPIAAKALS	0	0
EG12316	IKQGLLTVEKKGKKNIFSGRI	0	0
EG12316	ILEIEGLPDLKVEQAFELTDA	0	0
EG12316	KRGGGLCLGGKIAPIFFNTME	0	0
EG12316	LAEKLTVTVFKVTGETNTDDL	0	1
EG12316	LPHSDVFRQAKDVAESDRGFS	0	1
EG12316	LVHAIPLYAIKQGLLTVEKKG	0	0
EG12316	MFDNFYDVEEKAKAGNEYAKQ	0	0
EG12316	MGDDIPHVPNKRGGGLCLGGK	0	0
EG12316	MGDVIDVYPYKGEVRNHETGE	0	0
EG12316	____MLEEYRKHVAERAAEGI	0	1
EG12316	NFNQLSQYTEKADGVIFQTAV	0	0
EG12316	NQMAALVELLKNPPAGEEEFL	0	1
EG12316	PDQPGVVGPIKQIEALQQKGF	0	1
EG12316	PESVLVRFKGKMQPGITLRDL	0	0
EG12316	PIKQIEALQQKGFPLAYVGDV	0	0
EG12316	PLIIGRGLTTKAREALGLPHS	0	0
EG12316	PPGVDEAAYVKAGFLAAIAKG	0	0
EG12316	QGLLTVEKKGKKNIFSGRILE	0	0
EG12316	QKMVGRACGVKGIRPGAYCEP	0	0
EG12316	QSFCHTAAYPKPVDVNTHHTL	0	0
EG12316	RAAGKLLDAHKGQLPTRLWVA	0	0

EG12316	SAAGCTIKLNKEPIIEYLNSN	0	0
EG12316	TEEGYYSVFGKSGARIEIPGC	0	1
EG12316	TGELLATFELKTDVLIDEVRA	0	0
EG12316	TLERRIQGMEKWLANPELLEA	0	0
EG12316	TNIGHFRAAGKLLDAHKGQLP	0	1
EG12316	TTGPMTRDELKDLACLGFSAD	0	0
EG12316	VKAGFLAAIAKGEAKSPLLTP	0	1
EG12316	WFLNRPALAEKLTVTVFKVTG	0	0
EG13261	ALELLFGHLRKDLTAADGQTR	0	1
EG13261	ALGTGDALASKYRRQLYALLY	0	1
EG13261	AQYNGQFILAKLDCDAEQMIA	0	0
EG13261	DKVLPREEELKAQQAMQLMQE	0	0
EG13261	PEEAIRALLDKVLPREEELKA	0	0
EG13261	QGLVAQIELLKQAADTPEIQQ	0	0
EG13261	SNYTDALPLLKDAWQLSNQNG	0	0
EG13433	AALSGMEEGGKLAQEAGWLMA	0	0
EG13433	DPRPQAEIRAKDMSVFSSLIR	0	1
EG13433	FIDGMHEAGMKTTGKHFPGHG	0	0
EG13433	GERSYHADPQKALAIASRFID	0	1
EG13433	GHGAVTADSHKETPCDPRPQA	0	0
EG13433	KAERVTRLYHKGSFSRQELMD	0	0
EG13433	MHEAGMKTTGKHFPGHGAVTA	0	0
EG13433	NQLHERWQEEKAGH_______	0	0
EG13433	RPASGSPYWLKTVLRQELGFD	0	0
EG13433	RQELMDSARWKAISTRLNQLH	0	0
EG13433	SVFSSLIRENKLDAIMPAHVI	0	0
EG13433	VSVLDNLSPIKAERVTRLYHK	0	0
EG13723	AIEIPRLGLAK__________	0	0
EG13723	HTIALVGASDKPDRPSYRVMK	0	0
EG13723	KPDRPSYRVMKYLLDQGYHVI	0	1
EG13723	KVAGKTLLGQKGYGTLADVPE	0	1
EG13723	_________MKETDIAGILTS	0	1
EG13723	QGYHVIPVSPKVAGKTLLGQK	0	1
EG13723	VAQEAIAIGAKTLWMQLGVIN	0	1
EG13723	VIPVSPKVAGKTLLGQKGYGT	0	0
EG14074	ARIEKMGVTIKCNNEVGNTLT	0	0
EG14074	AVCARVCPTEKLCQSGCTRAG	0	0
EG14074	DVAMDVASTLKVLGCQAVTCV	0	0
EG14074	EELDEFPASEKEFTSARELGV	0	0
EG14074	EGDKTVVYAVKTGKEAAEAIH	0	0
EG14074	FAAGDIVEGDKTVVYAVKTGK	0	0
EG14074	FIRSIYFRNFKGAAETIRENN	0	0
EG14074	GFTPVAVEGNKVTFKHVRLSG	0	1
EG14074	IYQPGTKTLGKVAIIGAGPAG	0	1
EG14074	KTVVYAVKTGKEAAEAIHHYL	0	1
EG14074	LSGELTMAADKIILAVGQHAR	0	0
EG14074	NQGYDVTIYEKEAHPGGWLRN	0	0
EG14074	QACPAQTDPGKFIRSIYFRNF	0	0

EG14074	TGMEIYQPGTKTLGKVAIIGA	0	0
EG14074	TPAFTSLLAIKEASRCLLCHD	0	0
EG14074	VAVEGNKVTFKHVRLSGELTM	0	1
EG14074	VGNTLTLEQLKAENRAVLVTV	0	0
EG14074	VLDAEIARIEKMGVTIKCNNE	0	0
EG20151	AAGNYVFNERKMLDASHVVVF	0	0
EG20151	AAILDAEFGLKEKGYTSLVVV	0	0
EG20151	ADGRFATPEAKAANDKGRKFF	0	0
EG20151	AKTAMDDVWLKLVVDQEDADG	0	0
EG20151	ATPEAKAANDKGRKFFADMHR	0	1
EG20151	DASHVVVFCAKTAMDDVWLKL	0	1
EG20151	DLHDDAEWMAKQVYLNVGNFL	0	0
EG20151	EAKAANDKGRKFFADMHRKDL	0	0
EG20151	GRKFFADMHRKDLHDDAEWMA	0	0
EG20151	HFIVASTEEGKARVAKSAAGN	0	1
EG20151	ILDAEFGLKEKGYTSLVVVPV	0	0
EG20151	ISVALKRHSTKAFDASKKLTP	0	0
EG20151	KLTPEQAEQIKTLLQYSPSST	0	0
EG20151	__MDIISVALKRHSTKAFDAS	0	0
EG20151	RHSTKAFDASKKLTPEQAEQI	0	0
EG20151	SVEDFNATLPKSRLPQNITLT	0	0
EG50010	APPKKGEVLIKVTHTGVCHTD	0	0
EG50010	CGECEFCRSGKTNLCVAVRET	0	0
EG50010	EIVEIDVAPPKKGEVLIKVTH	0	1
EG50010	GRIIAIDTNPKKFDLARRFGA	0	0
EG50010	INPNDYDKPIKDVLLDINKWG	0	1
EG50010	IVEIDVAPPKKGEVLIKVTHT	0	0
EG50010	LAVVQGARQAKAGRIIAIDTN	0	1
EG50010	LCVAVRETQGKGLMPDGTTRF	0	0
EG50010	_________MKSRAAVAFAPG	0	0
EG50010	NDAFDLMHEGKSIRTVIRY__	0	0
EG50010	PFQLVTGRVWKGSAFGGVKGR	0	0
EG50010	PIKDVLLDINKWGIDHTFECI	0	0
EG50010	QLPGMVEDAMKGDIDLEPFVT	0	0
EG50010	RIIAIDTNPKKFDLARRFGAT	0	0
EG50010	TDCINPNDYDKPIKDVLLDIN	0	0
EG50010	TGIGAVHNTAKVQPGDSVAVF	0	0
EG50010	VEVGEGVTSVKPGDHVIPLYT	0	0
EG50010	VWKGSAFGGVKGRSQLPGMVE	0	0
EG50010	YTVVAEVSLAKINPEANHEHV	0	0

Table S8. The prediction results of PSoL for the test dataset.

Protein Name	Peptide	Class label	Prediction label
EG10032	AAVKSGSELGKQAKDIMDAGK	1	0
EG10032	HAPSGRVYHVKFNPPKVEGKD	1	1
EG10032	KSGSELGKQAKDIMDAGKLVT	1	0
EG10032	RVYHVKFNPPKVEGKDDVTGE	1	1

EG10241	DVVDAEFEEVKDKK_______	1	1
EG10241	VDAEFEEVKDKK_________	1	0
EG10420	ADAIFIEELRKADLYDKVSQA	1	0
EG10518	YRGPGLEEGMKIFQELKQTFG	1	1
EG10599	KATLEDLGQAKRVVINKDTTT	1	1
EG10599	SVAREIELEDKFENMGAQMVK	1	0
EG10599	VGKLIAEAMDKVGKEGVITVE	1	1
EG10758	IQGGGFEPGMKQKATKEPIKN	1	0
EG10789	TPSETAQLNLKKVLGFITDAG	1	1
EG10885	NKFPAIIYGGKEAPLAIELDH	1	0
EG10906	ALETLAQRSGKSELEAFEVAL	1	0
EG10906	ANELSDAAENKGTAVKKREDV	1	1
EG11005	NGCTVRNVYIKEAFDTGVRYD	1	0
EG11073	NLQNYLETELKRDNPVLIMGD	1	1
EG11137	VRDALEAAGLKADSAEVSMIP	1	1
EG11835	DDDEEEEEDEKQEDMMRLLRG	1	0
EG12316	FLAAIAKGEAKSPLLTPEKAI	1	0
EG13261	DLTAADGQTRKTFQEILAALG	1	0
EG13261	NRSEDAEAVLKTIPLQDQDTR	1	1
EG13433	CDMILVCNNRKGAVSVLDNLS	1	1
EG13723	GYGTLADVPEKVDMVDVFRNS	1	1
EG14074	AELEPQRNTIKTQNYQTRDPQ	1	0
EG20151	HSTKAFDASKKLTPEQAEQIK	1	1
EG20151	SRAAVAFAPGKPLEIVEIDVA	1	0
EG50010	STEEGKARVAKSAAGNYVFNE	1	1
EG10032	DVTGEELTTRKDDQEETVRKR	0	0
EG10032	EAEAGNTKYAKVDGTKPVAEV	0	0
EG10032	IILLGAPGAGKGTQAQFIMEK	0	0
EG10032	KGTQAQFIMEK__________	0	0
EG10032	KQAKDIMDAGKLVTDELVIAL	0	0
EG10032	MTAPLIGYYSKEAEAGNTKYA	0	0
EG10032	NTKYAKVDGTKPVAEVRADLE	0	1
EG10032	PRTIPQADAMKEAGINVDYVL	0	0
EG10032	PVAEVRADLEKILG_______	0	1
EG10032	RKDDQEETVRKRLVEYHQMTA	0	0
EG10032	STGDMLRAAVKSGSELGKQAK	0	0
EG10032	VKFNPPKVEGKDDVTGEELTT	0	1
EG10032	VTDELVIALVKERIAQEDCRN	0	1
EG10032	YSKEAEAGNTKYAKVDGTKPV	0	0
EG10241	ADNGDAWVEVKGQKMAPPQIS	0	0
EG10241	AGADASANNAKDDDVVDAEFE	0	1
EG10241	AKDKNSGKEQKITIKASSGLN	0	0
EG10241	ALETALKGEDKAAIEAKMQEL	0	0
EG10241	AMQRLKEAAEKAKIELSSAQQ	0	1
EG10241	APPQISAEVLKKMKKTAEDYL	0	1
EG10241	DADGILHVSAKDKNSGKEQKI	0	1
EG10241	DAEANAEADRKFEELVQTRNQ	0	1
EG10241	DAEFEEVKDKK__________	0	1

EG10241	DAGRIAGLEVKRIINEPTAAA	0	1
EG10241	DATGPKHMNIKVTRAKLESLV	0	0
EG10241	DGETLVGQPAKRQAVTNPQNT	0	0
EG10241	DGILHVSAKDKNSGKEQKITI	0	0
EG10241	DLVNRSIEPLKVALQDAGLSV	0	0
EG10241	EAGDKLPADDKTAIESALTAL	0	1
EG10241	GDAWVEVKGQKMAPPQISAEV	0	0
EG10241	GGQTRMPMVQKKVAEFFGKEP	0	0
EG10241	GQTRMPMVQKKVAEFFGKEP_	0	0
EG10241	HVSAKDKNSGKEQKITIKASS	0	0
EG10241	IIEIDEVDGEKTFEVLATNGD	0	0
EG10241	ISAEVLKKMKKTAEDYLGEPV	0	0
EG10241	KGEDKAAIEAKMQELAQVSQK	0	0
EG10241	KHMNIKVTRAKLESLVEDLVN	0	0
EG10241	KMQELAQVSQKLMEIAQQQHA	0	0
EG10241	LIAKNTTIPTKHSQVFSTAED	0	1
EG10241	LINYLVEEFKKDQGIDLRNDP	0	1
EG10241	MGGVMTTLIAKNTTIPTKHSQ	0	1
EG10241	________MGKIIGIDLGTTN	0	1
EG10241	NSGKEQKITIKASSGLNEDEI	0	0
EG10241	PPQISAEVLKKMKKTAEDYLG	0	0
EG10241	PYITADATGPKHMNIKVTRAK	0	0
EG10241	QGDHLLHSTRKQVEEAGDKLP	0	0
EG10241	QGERKRAADNKSLGQFNLDGI	0	1
EG10241	QISAEVLKKMKKTAEDYLGEP	0	0
EG10241	QRLKEAAEKAKIELSSAQQTD	0	1
EG10241	RLINYLVEEFKKDQGIDLRND	0	1
EG10241	RNDPLAMQRLKEAAEKAKIEL	0	0
EG10241	SALTALETALKGEDKAAIEAK	0	1
EG10241	SSGLNEDEIQKMVRDAEANAE	0	0
EG10241	TAAALAYGLDKGTGNRTIAVY	0	1
EG10241	TNPQNTLFAIKRLIGRRFQDE	0	0
EG10241	TRKQVEEAGDKLPADDKTAIE	0	0
EG10241	VAEFFGKEPRKDVNPDEAVAI	0	1
EG10241	VQGGVLTGDVKDVLLLDVTPL	0	0
EG10241	VQKKVAEFFGKEPRKDVNPDE	0	1
EG10241	VQRDVSIMPFKIIAADNGDAW	0	0
EG10241	VTIHVLQGERKRAADNKSLGQ	0	1
EG10241	YFNDAQRQATKDAGRIAGLEV	0	1
EG10420	AGENDPEAKRKIIGRVFVEVF	0	0
EG10420	ALAGENDPEAKRKIIGRVFVE	0	0
EG10420	ARIREQVGDDKVILGLSGGVD	0	1
EG10420	ASATGKAHVIKSHHNVGGLPK	0	0
EG10420	CPFAIMANEEKRFYGVQFHPE	0	0
EG10420	CQCEALWTPAKIIDDAVARIR	0	0
EG10420	DEEALKLEDVKWLAQGTIYPD	0	0
EG10420	EELRKADLYDKVSQAFTVFLP	0	1
EG10420	EMKMGLVEPLKELFKDEVRKI	0	1

EG10420	FVEVFDEEALKLEDVKWLAQG	0	1
EG10420	GIEDALTADGKPLLDVWMSHG	0	0
EG10420	GLGVRVLGEVKKEYCDLLRRA	0	0
EG10420	GLVEPLKELFKDEVRKIGLEL	0	0
EG10420	HNVGGLPKEMKMGLVEPLKEL	0	0
EG10420	ISRVVYDISGKPPATIEWE__	0	0
EG10420	KSHHNVGGLPKEMKMGLVEPL	0	0
EG10420	LGVRVLGEVKKEYCDLLRRAD	0	0
EG10420	LKELFKDEVRKIGLELGLPYD	0	0
EG10420	LLDVWMSHGDKVTAIPSDFIT	0	0
EG10420	____MTENIHKHRILILDFGS	0	0
EG10420	RSVGVMGDGRKYDWVVSLRAV	0	0
EG10420	TAMLLHRAIGKNLTCVFVDNG	0	0
EG10420	VIESAASATGKAHVIKSHHNV	0	0
EG10518	AKLEPFLKQMKAIDDLVKGFE	0	1
EG10518	AKTGAVINVKKPQFVSPGQMG	0	1
EG10518	CDGPSALPLAKLEPFLKQMKA	0	0
EG10518	EEGMKIFQELKQTFGVKIITD	0	1
EG10518	FQELKQTFGVKIITDVHEPSQ	0	0
EG10518	GQMGNIVDKFKEGGNEKVILC	0	0
EG10518	ICEHYVTVTQKLGIPYVFKAS	0	0
EG10518	IEAHPDPEHAKCDGPSALPLA	0	0
EG10518	IPYVFKASFDKANRSSIHSYR	0	0
EG10518	KQMKAIDDLVKGFEELDTSK_	0	0
EG10518	LPLAKLEPFLKQMKAIDDLVK	0	1
EG10518	MAKTGAVINVKKPQFVSPGQM	0	0
EG10518	_________MKQKVVSIGDIN	0	0
EG10518	_______MKQKVVSIGDINVA	0	0
EG10518	RQTDLVEAMAKTGAVINVKKP	0	1
EG10518	SPGQMGNIVDKFKEGGNEKVI	0	0
EG10518	TQKLGIPYVFKASFDKANRSS	0	1
EG10518	VDKFKEGGNEKVILCDRGANF	0	0
EG10518	_VDMLGFSIMKKVSGNSPVIF	0	0
EG10518	VKGFEELDTSK__________	0	1
EG10518	VVDMLGFSIMKKVSGNSPVIF	0	0
EG10599	AATEVEMKEKKARVEDALHAT	0	0
EG10599	ADAVKVTLGPKGRNVVLDKSF	0	1
EG10599	AKLAGGVAVIKVGAATEVEMK	0	1
EG10599	DKSFGAPTITKDGVSVAREIE	0	0
EG10599	DREKLQERVAKLAGGVAVIKV	0	0
EG10599	DVKFGNDARVKMLRGVNVLAD	0	0
EG10599	EEATSDYDREKLQERVAKLAG	0	0
EG10599	EEPSVVANTVKGGDGNYGYNA	0	0
EG10599	EMLPVLEAVAKAGKPLLIIAE	0	0
EG10599	ESPFILLADKKISNIREMLPV	0	0
EG10599	GAATEVEMKEKKARVEDALHA	0	0
EG10599	GAQMVKEVASKANDAAGDGTT	0	0
EG10599	GGVALIRVASKLADLRGQNED	0	1

EG10599	GPKGRNVVLDKSFGAPTITKD	0	1
EG10599	GQNEDQNVGIKVALRAMEAPL	0	0
EG10599	ISANSDETVGKLIAEAMDKVG	0	0
EG10599	ISEEIGMELEKATLEDLGQAK	0	0
EG10599	KALSVPCSDSKAIAQVGTISA	0	1
EG10599	KAVTAAVEELKALSVPCSDSK	0	0
EG10599	KFENMGAQMVKEVASKANDAA	0	0
EG10599	KVGAATEVEMKEKKARVEDAL	0	0
EG10599	LAQAIITEGLKAVAAGMNPMD	0	0
EG10599	LESPFILLADKKISNIREMLP	0	0
EG10599	LGQAKRVVINKDTTTIIDGVG	0	1
EG10599	LIAEAMDKVGKEGVITVEDGT	0	1
EG10599	LVVNTMRGIVKVAAVKAPGFG	0	1
EG10599	_______MAAKDVKFGNDARV	0	0
EG10599	____MAAKDVKFGNDARVKML	0	0
EG10599	MIDMGILDPTKVTRSALQYAA	0	0
EG10599	MRGIVKVAAVKAPGFGDRRKA	0	1
EG10599	NPMDLKRGIDKAVTAAVEELK	0	0
EG10599	PVLEAVAKAGKPLLIIAEDVE	0	1
EG10599	RGVNVLADAVKVTLGPKGRNV	0	0
EG10599	RGYLSPYFINKPETGAVELES	0	1
EG10599	TTECMVTDLPKNDAADLGAAG	0	1
EG10599	VAAGMNPMDLKRGIDKAVTAA	0	0
EG10599	VKAPGFGDRRKAMLQDIATLT	0	0
EG10758	EVVDGMDVVDKIKGVATGRSG	0	1
EG10758	FEPGMKQKATKEPIKNEANNG	0	0
EG10758	FHTNHGDIVIKTFDDKAPETV	0	0
EG10758	GDIVIKTFDDKAPETVKNFLD	0	0
EG10758	GGGFEPGMKQKATKEPIKNEA	0	0
EG10758	GRSGMHQDVPKEDVIIESVTV	0	0
EG10758	MKQKATKEPIKNEANNGLKNT	0	1
EG10758	PIKNEANNGLKNTRGTLAMAR	0	0
EG10758	TFDDKAPETVKNFLDYCREGF	0	0
EG10758	VDGMDVVDKIKGVATGRSGMH	0	0
EG10789	AAATIARHLAKEVDFFSIGTN	0	1
EG10789	AKASAQLETIKTKAGETFGEE	0	1
EG10789	ALEELDDEYLKERAADVRDIG	0	1
EG10789	ASAQLETIKTKAGETFGEEKE	0	1
EG10789	ASEKAELAKLKDLPAITLDGH	0	0
EG10789	DKELPYMNFPKEENPFLGWRA	0	0
EG10789	ELEQEIIALIKDKHMTADAAA	0	0
EG10789	EQEIIALIKDKHMTADAAAHE	0	1
EG10789	ERAADVRDIGKRLLRNILGLK	0	1
EG10789	EVERFLSGRAKASAQLETIKT	0	1
EG10789	GIAFGKALLLKEDEIVIDRKK	0	0
EG10789	IIRNTNFEDAKVLAEQALAQP	0	0
EG10789	ILASPGIAFGKALLLKEDEIV	0	0
EG10789	ISVEEVRALRKEIEIYKQELR	0	1

EG10789	IVRTMDIGGDKELPYMNFPKE	0	0
EG10789	IYKQELRDEGKAFDESIEIGV	0	1
EG10789	_KEDEIVIDRKKISADQVDQE	0	0
EG10789	KRLLRNILGLKIIDLSAIQDE	0	0
EG10789	LKEDEIVIDRKKISADQVDQE	0	0
EG10789	MSAISIPRIKKIIRNTNFEDA	0	0
EG10789	MTLVNKFIEEKTIC_______	0	0
EG10789	PMSPSVLNLIKQVIDASHAEG	0	0
EG10789	PSETAQLNLKKVLGFITDAGG	0	1
EG10789	PTEEEQFAAYKAVAEACGSQA	0	0
EG10789	QVASEKAELAKLKDLPAITLD	0	0
EG10789	QVIDASHAEGKWTGMCGELAG	0	0
EG10789	RAILRASAFGKLRIMFPMIIS	0	0
EG10789	RALRKEIEIYKQELRDEGKAF	0	1
EG10789	RAVQEQVASEKAELAKLKDLP	0	0
EG10789	SMSAISIPRIKKIIRNTNFED	0	0
EG10789	TKAGETFGEEKEAIFEGHIML	0	1
EG10789	TTDELMTLVNKFIEEKTIC__	0	0
EG10789	VGTGSVTSQVKNDDYLILDAV	0	0
EG10789	YVNPTNEVIDKMRAVQEQVAS	0	0
EG10885	APLAIELDHDKVMNMQAKAEF	0	0
EG10885	DHDKVMNMQAKAEFYSEVLTI	0	1
EG10885	GASRRLRAANKFPAIIYGGKE	0	1
EG10885	INAEVRKEQGKGASRRLRAAN	0	1
EG10885	IVVDGKEIKVKAQDVQRHPYK	0	0
EG10885	KAQDVQRHPYKPKLQHIDFVR	0	0
EG10885	LTIVVDGKEIKVKAQDVQRHP	0	1
EG10885	_MFTINAEVRKEQGKGASRRL	0	0
EG10885	QDVQRHPYKPKLQHIDFVRA_	0	0
EG10885	SEVLTIVVDGKEIKVKAQDVQ	0	0
EG10906	AAENKGTAVKKREDVHRMAEA	0	0
EG10906	AMRWIVEAARKRGDKSMALRL	0	0
EG10906	DAAENKGTAVKKREDVHRMAE	0	0
EG10906	DPKFGSELLAKFVNILMVDGK	0	1
EG10906	EDVHRMAEANKAFAHYRWLSL	0	0
EG10906	FVNILMVDGKKSTAESIVYSA	0	0
EG10906	IGQRKILPDPKFGSELLAKFV	0	0
EG10906	IVEAARKRGDKSMALRLANEL	0	0
EG10906	KFVNILMVDGKKSTAESIVYS	0	0
EG10906	LENVRPTVEVKSRRVGGSTYQ	0	1
EG10906	MPRRRVIGQRKILPDPKFGSE	0	0
EG10906	SFSHQAGASSKQPALGYLN__	0	0
EG11005	AEQIYIPVLIKKREQEKGLDR	0	0
EG11005	AFDTGVRYDFKGNFDLEGLER	0	1
EG11005	ANLKAMYSIAKKYDIPVVMDS	0	0
EG11005	ARFAENAYFIKQREAEYKDWT	0	0
EG11005	DFIIEAFKHVKENAANIKGLT	0	0
EG11005	EPKVLRHFTAKLKEV______	0	1

EG11005	_EQIYIPVLIKKREQEKGLDR	0	1
EG11005	FLLGRDPKTGKQLPCPAELLR	0	1
EG11005	GGHAAFVDAGKLLPHIPADQF	0	1
EG11005	GGQPVSLANLKAMYSIAKKYD	0	1
EG11005	IGSFLLGRDPKTGKQLPCPAE	0	0
EG11005	IKGLTFTYEPKVLRHFTAKLK	0	0
EG11005	KHVKENAANIKGLTFTYEPKV	0	0
EG11005	KVLRHFTAKLKEV________	0	1
EG11005	KYADMLAMSAKKDAMVPMGGL	0	0
EG11005	______MENFKHLPEPFRIRV	0	0
EG11005	MVPMGGLLCMKDDSFFDVYTE	0	0
EG11005	NLKAMYSIAKKYDIPVVMDSA	0	0
EG11005	PAQALACELYKVAGIRAVEIG	0	0
EG11005	PFRIRVIEPVKRTTRAYREEA	0	0
EG11005	PVLIKKREQEKGLDRSKMVAF	0	1
EG11005	REQEKGLDRSKMVAFSNYFFD	0	1
EG11005	RSYYALAESVKNIFGYQYTIP	0	0
EG11005	THMDFIIEAFKHVKENAANIK	0	0
EG11005	TIEQITRETYKYADMLAMSAK	0	0
EG11005	TRAYREEAIIKSGMNPFLLDS	0	0
EG11005	YADMLAMSAKKDAMVPMGGLL	0	0
EG11005	YFIKQREAEYKDWTIEQITRE	0	1
EG11073	DDMFPLEEVAKLGYNVFYHGQ	0	0
EG11073	DRFSWFDYRSKGFDDNRGLRI	0	0
EG11073	ENRKRWLRTGKCSFLPEEREW	0	0
EG11073	GIDYEIRSMEKPSDHAPVWAT	0	1
EG11073	KGHYGVALLTKETPIAVRRGF	0	0
EG11073	LDIGIGEENRKRWLRTGKCSF	0	0
EG11073	LGYNVFYHGQKGHYGVALLTK	0	0
EG11073	_________MKFVSFNINGLR	0	0
EG11073	PQGESRDHPIKFPAKAQFYQN	0	0
EG11073	QPDVIGLQETKVHDDMFPLEE	0	0
EG11073	RPHQLEAIVEKHQPDVIGLQE	0	0
EG11073	SRDHPIKFPAKAQFYQNLQNY	0	0
EG11137	ANPRLRAAVDKALSNNMTRDT	0	1
EG11137	DGSVAYLFSKKGVISFEKGDE	0	0
EG11137	DVYTAWEEMGKVRDALEAAGL	0	1
EG11137	FSKKGVISFEKGDEDTIMEAA	0	0
EG11137	HSKWANTRHRKAAQDAKRGKI	0	1
EG11137	KADMDAETAPKLMRLIDMLED	0	0
EG11137	KIIRELVTAAKLGGGDPDANP	0	0
EG11137	_____MAGHSKWANTRHRKAA	0	0
EG11137	QDAKRGKIFTKIIRELVTAAK	0	0
EG11137	RKAAQDAKRGKIFTKIIRELV	0	0
EG11137	SAEVSMIPSTKADMDAETAPK	0	0
EG11137	TDGSVAYLFSKKGVISFEKGD	0	0
EG11137	TRHRKAAQDAKRGKIFTKIIR	0	0
EG11137	TVAEVRHAFSKCGGNLGTDGS	0	1

EG11835	AAGGNTTSGSKGQNAPKDPRI	0	1
EG11835	DQEARDRKRQKKRRGHAPGSR	0	0
EG11835	EELDQEARDRKRQKKRRGHAP	0	0
EG11835	GVTEKVTKQHKPKSEKPMLSP	0	0
EG11835	IPLGVTEKVTKQHKPKSEKPM	0	0
EG11835	KLDRIDELMQKLGLSYDDDEE	0	1
EG11835	KTPIPLGVTEKVTKQHKPKSE	0	0
EG11835	__________MKPSSSNSRSKG	0	0
EG11835	MKPSSSNSRSKGHAKARRKTR	0	0
EG11835	NAPKDPRIGSKTPIPLGVTEK	0	0
EG11835	QEARDRKRQKKRRGHAPGSRA	0	0
EG11835	RSKGHAKARRKTREELDQEAR	0	0
EG11835	SAEEQSWVDAKLDRIDELMQK	0	0
EG11835	SSNSRSKGHAKARRKTREELD	0	0
EG11835	TEKVTKQHKPKSEKPMLSPQA	0	1
EG11835	TSGSKGQNAPKDPRIGSKTPI	0	0
EG11835	VTKQHKPKSEKPMLSPQAELE	0	0
EG12316	AERAAEGIAPKPLDANQMAAL	0	0
EG12316	AERSAAGCTIKLNKEPIIEYL	0	1
EG12316	AIKQGLLTVEKKGKKNIFSGR	0	0
EG12316	ARPLSAVQGEKIDEVFIGSCM	0	0
EG12316	AVIDIDLADIKEPILCAPNDP	0	0
EG12316	DDAKLAPIAAKALSHTLLMFD	0	0
EG12316	DIPLHALAMLKNAREGIEPDQ	0	0
EG12316	DMPESVLVRFKGKMQPGITLR	0	0
EG12316	DNFYDVEEKAKAGNEYAKQVM	0	1
EG12316	EAKSPLLTPEKAIELLGTMQG	0	1
EG12316	EKAKAGNEYAKQVMQSWADAE	0	0
EG12316	ELAAVAALIGKLPTPEEYQTY	0	0
EG12316	ESDRGFSLAQKMVGRACGVKG	0	0
EG12316	EYLNSNIVLLKWMIAEGYGDR	0	0
EG12316	EYQTYVAQVDKTAVDTYRYLN	0	0
EG12316	GDVVGTGSSRKSATNSVLWFM	0	1
EG12316	GIRPGAYCEPKMTSVGSQDTT	0	0
EG12316	GLLTVEKKGKKNIFSGRILEI	0	0
EG12316	HPLIDALDDAKLAPIAAKALS	0	0
EG12316	IKQGLLTVEKKGKKNIFSGRI	0	0
EG12316	ILEIEGLPDLKVEQAFELTDA	0	1
EG12316	KRGGGLCLGGKIAPIFFNTME	0	0
EG12316	LAEKLTVTVFKVTGETNTDDL	0	1
EG12316	LPHSDVFRQAKDVAESDRGFS	0	0
EG12316	LVHAIPLYAIKQGLLTVEKKG	0	0
EG12316	MFDNFYDVEEKAKAGNEYAKQ	0	1
EG12316	MGDDIPHVPNKRGGGLCLGGK	0	0
EG12316	MGDVIDVYPYKGEVRNHETGE	0	0
EG12316	____MLEEYRKHVAERAAEGI	0	0
EG12316	NFNQLSQYTEKADGVIFQTAV	0	0
EG12316	NQMAALVELLKNPPAGEEEFL	0	0

EG12316	PDQPGVVGPIKQIEALQQKGF	0	0
EG12316	PESVLVRFKGKMQPGITLRDL	0	0
EG12316	PIKQIEALQQKGFPLAYVGDV	0	0
EG12316	PLIIGRGLTTKAREALGLPHS	0	0
EG12316	PPGVDEAAYVKAGFLAAIAKG	0	0
EG12316	QGLLTVEKKGKKNIFSGRILE	0	0
EG12316	QKMVGRACGVKGIRPGAYCEP	0	0
EG12316	QSFCHTAAYPKPVDVNTHHTL	0	0
EG12316	RAAGKLLDAHKGQLPTRLWVA	0	0
EG12316	SAAGCTIKLNKEPIIEYLNSN	0	1
EG12316	TEEGYYSVFGKSGARIEIPGC	0	0
EG12316	TGELLATFELKTDVLIDEVRA	0	0
EG12316	TLERRIQGMEKWLANPELLEA	0	0
EG12316	TNIGHFRAAGKLLDAHKGQLP	0	0
EG12316	TTGPMTRDELKDLACLGFSAD	0	0
EG12316	VKAGFLAAIAKGEAKSPLLTP	0	1
EG12316	WFLNRPALAEKLTVTVFKVTG	0	0
EG13261	ALELLFGHLRKDLTAADGQTR	0	0
EG13261	ALGTGDALASKYRRQLYALLY	0	0
EG13261	AQYNGQFILAKLDCDAEQMIA	0	1
EG13261	DKVLPREEELKAQQAMQLMQE	0	0
EG13261	PEEAIRALLDKVLPREEELKA	0	1
EG13261	QGLVAQIELLKQAADTPEIQQ	0	0
EG13261	SNYTDALPLLKDAWQLSNQNG	0	0
EG13433	AALSGMEEGGKLAQEAGWLMA	0	0
EG13433	DPRPQAEIRAKDMSVFSSLIR	0	0
EG13433	FIDGMHEAGMKTTGKHFPGHG	0	0
EG13433	GERSYHADPQKALAIASRFID	0	0
EG13433	GHGAVTADSHKETPCDPRPQA	0	0
EG13433	KAERVTRLYHKGSFSRQELMD	0	0
EG13433	MHEAGMKTTGKHFPGHGAVTA	0	0
EG13433	NQLHERWQEEKAGH_______	0	0
EG13433	RPASGSPYWLKTVLRQELGFD	0	1
EG13433	RQELMDSARWKAISTRLNQLH	0	0
EG13433	SVFSSLIRENKLDAIMPAHVI	0	0
EG13433	VSVLDNLSPIKAERVTRLYHK	0	0
EG13723	AIEIPRLGLAK__________	0	0
EG13723	HTIALVGASDKPDRPSYRVMK	0	0
EG13723	KPDRPSYRVMKYLLDQGYHVI	0	0
EG13723	KVAGKTLLGQKGYGTLADVPE	0	0
EG13723	_________MKETDIAGILTS	0	0
EG13723	QGYHVIPVSPKVAGKTLLGQK	0	0
EG13723	VAQEAIAIGAKTLWMQLGVIN	0	0
EG13723	VIPVSPKVAGKTLLGQKGYGT	0	0
EG14074	ARIEKMGVTIKCNNEVGNTLT	0	0
EG14074	AVCARVCPTEKLCQSGCTRAG	0	0
EG14074	DVAMDVASTLKVLGCQAVTCV	0	0
EG14074	EELDEFPASEKEFTSARELGV	0	0

EG14074	EGDKTVVYAVKTGKEAAEAIH	0	1
EG14074	FAAGDIVEGDKTVVYAVKTGK	0	0
EG14074	FIRSIYFRNFKGAAETIRENN	0	0
EG14074	GFTPVAVEGNKVTFKHVRLSG	0	0
EG14074	IYQPGTKTLGKVAIIGAGPAG	0	0
EG14074	KTVVYAVKTGKEAAEAIHHYL	0	0
EG14074	LSGELTMAADKIILAVGQHAR	0	0
EG14074	NQGYDVTIYEKEAHPGGWLRN	0	0
EG14074	QACPAQTDPGKFIRSIYFRNF	0	0
EG14074	TGMEIYQPGTKTLGKVAIIGA	0	0
EG14074	TPAFTSLLAIKEASRCLLCHD	0	0
EG14074	VAVEGNKVTFKHVRLSGELTM	0	0
EG14074	VGNTLTLEQLKAENRAVLVTV	0	0
EG14074	VLDAEIARIEKMGVTIKCNNE	0	0
EG20151	AAGNYVFNERKMLDASHVVVF	0	1
EG20151	AAILDAEFGLKEKGYTSLVVV	0	0
EG20151	ADGRFATPEAKAANDKGRKFF	0	1
EG20151	AKTAMDDVWLKLVVDQEDADG	0	1
EG20151	ATPEAKAANDKGRKFFADMHR	0	0
EG20151	DASHVVVFCAKTAMDDVWLKL	0	0
EG20151	DLHDDAEWMAKQVYLNVGNFL	0	1
EG20151	EAKAANDKGRKFFADMHRKDL	0	0
EG20151	GRKFFADMHRKDLHDDAEWMA	0	1
EG20151	HFIVASTEEGKARVAKSAAGN	0	0
EG20151	ILDAEFGLKEKGYTSLVVVPV	0	0
EG20151	ISVALKRHSTKAFDASKKLTP	0	0
EG20151	KLTPEQAEQIKTLLQYSPSST	0	0
EG20151	__MDIISVALKRHSTKAFDAS	0	1
EG20151	RHSTKAFDASKKLTPEQAEQI	0	1
EG20151	SVEDFNATLPKSRLPQNITLT	0	0
EG50010	APPKKGEVLIKVTHTGVCHTD	0	0
EG50010	CGECEFCRSGKTNLCVAVRET	0	0
EG50010	EIVEIDVAPPKKGEVLIKVTH	0	1
EG50010	GRIIAIDTNPKKFDLARRFGA	0	0
EG50010	INPNDYDKPIKDVLLDINKWG	0	0
EG50010	IVEIDVAPPKKGEVLIKVTHT	0	1
EG50010	LAVVQGARQAKAGRIIAIDTN	0	1
EG50010	LCVAVRETQGKGLMPDGTTRF	0	0
EG50010	_________MKSRAAVAFAPG	0	0
EG50010	NDAFDLMHEGKSIRTVIRY__	0	1
EG50010	PFQLVTGRVWKGSAFGGVKGR	0	0
EG50010	PIKDVLLDINKWGIDHTFECI	0	0
EG50010	QLPGMVEDAMKGDIDLEPFVT	0	0
EG50010	RIIAIDTNPKKFDLARRFGAT	0	0
EG50010	TDCINPNDYDKPIKDVLLDIN	0	0
EG50010	TGIGAVHNTAKVQPGDSVAVF	0	0
EG50010	VEVGEGVTSVKPGDHVIPLYT	0	0
EG50010	VWKGSAFGGVKGRSQLPGMVE	0	0

EG50010	YTVVAEVSLAKINPEANHEHV	0	0

Table S9. The prediction results of SVM_balance for the test dataset.

Protein Name	Peptide	Class label	Prediction result
EG10032	AAVKSGSELGKQAKDIMDAGK	1	0
EG10032	HAPSGRVYHVKFNPPKVEGKD	1	1
EG10032	KSGSELGKQAKDIMDAGKLVT	1	1
EG10032	RVYHVKFNPPKVEGKDDVTGE	1	1
EG10241	DVVDAEFEEVKDKK_______	1	1
EG10241	VDAEFEEVKDKK_________	1	1
EG10420	ADAIFIEELRKADLYDKVSQA	1	0
EG10518	YRGPGLEEGMKIFQELKQTFG	1	0
EG10599	KATLEDLGQAKRVVINKDTTT	1	0
EG10599	SVAREIELEDKFENMGAQMVK	1	1
EG10599	VGKLIAEAMDKVGKEGVITVE	1	1
EG10758	IQGGGFEPGMKQKATKEPIKN	1	1
EG10789	TPSETAQLNLKKVLGFITDAG	1	0
EG10885	NKFPAIIYGGKEAPLAIELDH	1	0
EG10906	ALETLAQRSGKSELEAFEVAL	1	1
EG10906	ANELSDAAENKGTAVKKREDV	1	1
EG11005	NGCTVRNVYIKEAFDTGVRYD	1	1
EG11073	NLQNYLETELKRDNPVLIMGD	1	0
EG11137	VRDALEAAGLKADSAEVSMIP	1	1
EG11835	DDDEEEEEDEKQEDMMRLLRG	1	0
EG12316	FLAAIAKGEAKSPLLTPEKAI	1	1
EG13261	DLTAADGQTRKTFQEILAALG	1	1
EG13261	NRSEDAEAVLKTIPLQDQDTR	1	0
EG13433	CDMILVCNNRKGAVSVLDNLS	1	0
EG13723	GYGTLADVPEKVDMVDVFRNS	1	0
EG14074	AELEPQRNTIKTQNYQTRDPQ	1	1
EG20151	HSTKAFDASKKLTPEQAEQIK	1	1
EG20151	SRAAVAFAPGKPLEIVEIDVA	1	1
EG50010	STEEGKARVAKSAAGNYVFNE	1	1
EG10518	AKLEPFLKQMKAIDDLVKGFE	0	0
EG10518	AKTGAVINVKKPQFVSPGQMG	0	0
EG10518	CDGPSALPLAKLEPFLKQMKA	0	0
EG10518	EEGMKIFQELKQTFGVKIITD	0	1
EG10518	FQELKQTFGVKIITDVHEPSQ	0	0
EG10518	GQMGNIVDKFKEGGNEKVILC	0	0
EG10518	ICEHYVTVTQKLGIPYVFKAS	0	1
EG10599	AKLAGGVAVIKVGAATEVEMK	0	1
EG10599	DREKLQERVAKLAGGVAVIKV	0	1
EG10599	DVKFGNDARVKMLRGVNVLAD	0	0
EG10599	EEATSDYDREKLQERVAKLAG	0	1
EG10599	EEPSVVANTVKGGDGNYGYNA	0	0
EG10599	EMLPVLEAVAKAGKPLLIIAE	0	0

EG10599	ESPFILLADKKISNIREMLPV	0	1
EG10789	DKELPYMNFPKEENPFLGWRA	0	0
EG10789	ELEQEIIALIKDKHMTADAAA	0	0
EG10789	EQEIIALIKDKHMTADAAAHE	0	1
EG10789	ERAADVRDIGKRLLRNILGLK	0	0
EG10789	EVERFLSGRAKASAQLETIKT	0	1
EG10789	GIAFGKALLLKEDEIVIDRKK	0	0
EG11005	MVPMGGLLCMKDDSFFDVYTE	0	0
EG11005	NLKAMYSIAKKYDIPVVMDSA	0	0
EG11005	PAQALACELYKVAGIRAVEIG	0	0
EG11005	PFRIRVIEPVKRTTRAYREEA	0	0
EG13433	DPRPQAEIRAKDMSVFSSLIR	0	1
EG13433	FIDGMHEAGMKTTGKHFPGHG	0	0
EG50010	VEVGEGVTSVKPGDHVIPLYT	0	0
EG50010	VWKGSAFGGVKGRSQLPGMVE	0	0
EG50010	YTVVAEVSLAKINPEANHEHV	0	1
